# Self-Healing Polymeric Soft Actuators

**DOI:** 10.1021/acs.chemrev.2c00418

**Published:** 2022-12-21

**Authors:** Sebastian Bonardd, Mridula Nandi, José Ignacio Hernández García, Binoy Maiti, Alex Abramov, David Díaz Díaz

**Affiliations:** † Departamento de Química Orgánica, Universidad de La Laguna, Avenida Astrofísico Francisco Sánchez, La Laguna 38206, Tenerife Spain; ‡ Instituto Universitario de Bio-Orgánica Antonio González, Universidad de La Laguna, Avenida Astrofísico Francisco Sánchez, La Laguna 38206, Tenerife Spain; § Department of Chemical and Biomolecular Engineering, University of Delaware, Newark, Delaware 19716, United States; ∥ School of Chemistry & Biochemistry, Georgia Institute of Technology, 901 Atlantic Drive NW, Atlanta, Georgia 30332, United States; ⊥ Institute of Organic Chemistry, University of Regensburg, Universitätstrasse 31, Regensburg 93053, Germany

## Abstract

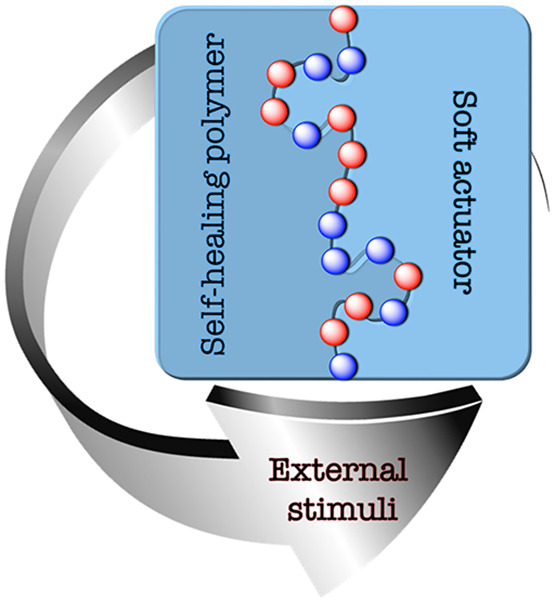

Natural evolution has provided multicellular organisms with sophisticated functionalities and repair mechanisms for surviving and preserve their functions after an injury and/or infection. In this context, biological systems have inspired material scientists over decades to design and fabricate both self-healing polymeric materials and soft actuators with remarkable performance. The latter are capable of modifying their shape in response to environmental changes, such as temperature, pH, light, electrical/magnetic field, chemical additives, etc. In this review, we focus on the fusion of both types of materials, affording new systems with the potential to revolutionize almost every aspect of our modern life, from healthcare to environmental remediation and energy. The integration of stimuli-triggered self-healing properties into polymeric soft actuators endow environmental friendliness, cost-saving, enhanced safety, and lifespan of functional materials. We discuss the details of the most remarkable examples of self-healing soft actuators that display a macroscopic movement under specific stimuli. The discussion includes key experimental data, potential limitations, and mechanistic insights. Finally, we include a general table providing at first glance information about the nature of the external stimuli, conditions for self-healing and actuation, key information about the driving forces behind both phenomena, and the most important features of the achieved movement.

## 1 Introduction

### 1.1 Self-Healing Soft Materials

Over millions of years of evolution, multicellular organisms have developed different repair mechanisms to survive in a specific ecosystem and preserve their functions after an injury and/or infection.^1−4^ The large biological diversity in the animal and plant kingdoms has resulted in astoundingly diverse types of healing processes found in nature.^2,5−7^ In general, the biological complexity of living systems is matched by complexity in the healing mechanism. Although plants and animals share some key aspects of healing processes, their differences in need and biology generate also unique pathways, such as clotting in animals and damage containment (i.e., process of discarding infected/damaged tissue) in plants.^1^ Some of the most appealing examples of biological healing include, just to mention a few, the muscle contraction and extension through the transformation of chemical to mechanical energy,^8−10^ adaptive camouflage through muscle control,^11^ both innate and adaptative immune response of animals and plants,^12^ the regeneration of nervous systems by maintaining the homeostatic environment surrounding the nerves,^13,14^ the healing of hard^11,15^ and soft tissues^8,16,17^ in both vertebrate and invertebrates through a complex set of cellular signals and humoral responses, the release of special secretion cells or vascular networks to release healing molecular fluid when an injury breaks the cell walls of some plants,^1^ and the self-healing process of the human skin via an inflammatory response of cells below the dermis by increasing collagen production.^18^

What seems to be clear is that the self-healing processes found in living systems involves a complex cascade of physical events and chemical reactions, however, the exact chemistries of which are far from understood in many cases even though scientists continue to seek inspiration in biological systems to design new and more reliable self-healing materials. In materials, the presence of local regions with lower performance than that of the surrounding areas is referred as damage.^19^ Thus, the challenge in the design of self-healing materials is centered in the fabrication of new systems, in most cases composites, with an autonomous or externally stimulated damage healing ability in order to extend their useful life in a given application. Therefore, the incorporation of, for instance, self-healing agents in a material would likely modify its properties. Hence, it is of utmost importance to monitor those changes in order to assess the performance of the new material, which ideally should be at least equal to that of the unmodified system. From a practical point of view, we should consider that in most cases the ability of self-healing materials to recuperate, autonomously^20^ or externally assisted, their initial properties is mainly affected by the selection of the healing agents. As a matter of fact, a large variety of self-healing agents have been extensively studied to meet the highly demanding requirements of smart materials for high-tech applications.

**Figure 1:**
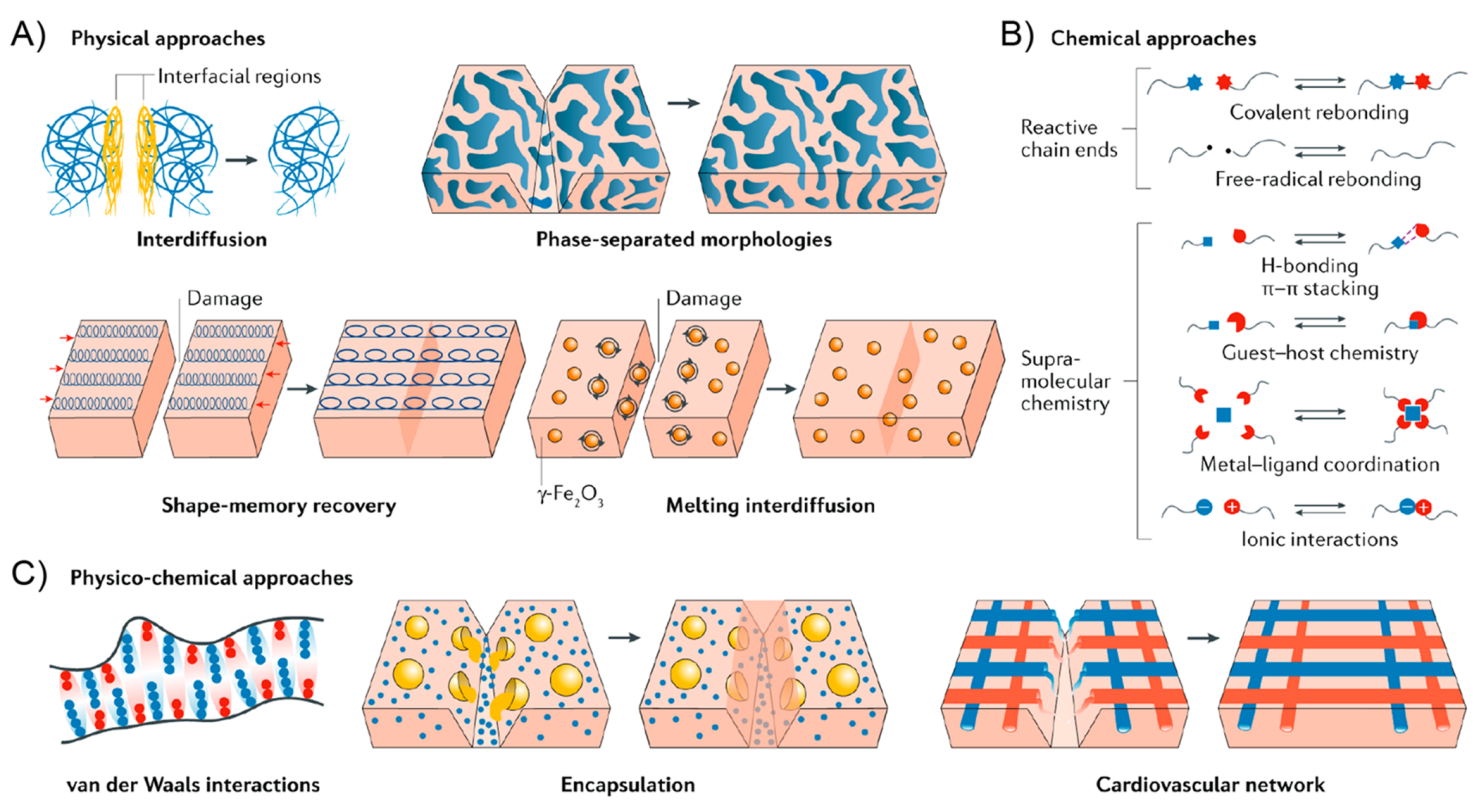
Self-healing mechanisms. (A) Physical processes to realize self-healing include interdiffusion of polymer chains, the introduction of phase-separated morphologies, shape-memory effects, and the introduction of active nanoparticles into a polymer matrix. (B) Chemical processes to facilitate self-healing involve either introducing reactive chain ends or supramolecular chemistries. (C) Physical and chemical processes can be combined to realize self-healing. Self-healing is achieved by incorporating enhanced van der Waals interactions, or encapsulating nanocapsules or microcapsules containing reactive liquids to heal a wound, or by mimicking cardiovascular architectures composed of hollow fibers filled with reactive chemicals to heal a polymer matrix. Reproduced with permission from ref (21). Copyright 2020 Nature Publishing Group.

Within this context, the principal strategies for the synthesis of self-healable polymers involve either physical or chemical occurrences at the molecular level, although an overlap between the two approaches results are evident when looking at the self-healing phenomenon as a whole.^21,22^ As shown in Figure 1, the most common examples of physical self-healing processes involve interchain diffusion,^23^ phase-separated morphologies,^24,25^ shape-memory effects,^26,27^ and the incorporation of superparamagnetic nanoparticles.^28^ On the other hand, chemical self-healing processes comprise mainly the integration of covalent,^29−31^ free-radical,^32,33^ or supramolecular^25,34−36^ dynamic bonds into the material.^37^ As mentioned, the combination of physical and chemical events constitutes also a versatile strategy for fine-tuning the healing process of the materials with multiscale complexity such as interdigitated copolymer morphologies^20^ and cardiovascular networks.^38^

Based on the publications and patents reported during the last decades on this vast field, intrinsic (i.e., without external input), capsule-based, and vascular methods constitute probably the main approaches used in order to impart self-healing capability to different materials. In the case of intrinsic self-healing materials, such as the pioneering epoxy systems,^31^ repair is achieved through the inherent reversibility of bonding in the polymer matrix phase. Furthermore, among a significant number of reversible reactions,^39−41^ the thermal Diels–Alder (DA) reaction for cross-linking linear polymers^42−45^ and disulfide bond formation,^46^ together with the usage of host–guest interactions^47^ have been widely explored for the fabrication of self-healing composites capable of multiple healing.^48^ Furthermore, a promising chemical strategy combining reversible covalent linkages through imine bond formation with noncovalent interactions through hydrogen bonds has been also reported.^49^ Alternatively, microcapsule-based self-healing materials were first proposed by White et al.^20^ and nowadays are highly extended due to their ease of applicability and their potential for mass production. These microcapsules, embedded in the polymer matrix, contain the specific healing agent that will be delivered to the damaged zone upon physical rupture of the capsule.^50,51^ In particular, capsule-based self-healing coatings have been studied by many research groups during the past few years due to the increased importance of preserving the potential of protection of the underlying substrate.^52−60^ Finally, similar to blood vessels, vascular self-healing materials incorporate healing agents into a polymer matrix through well-defined microchannels, an idea originally proposed by Toohey et al.^61^ Vascular self-healing materials have been extensively reported during the past decade due to their healing versatility and the large scale of damage that can be healed with this method.^62−72^ With all this in mind, the possibility of providing self-healing capability not only to static materials but also to stimuli-responsive dynamic systems (i.e., actuators) has been a focus of attention in both academia and industry.

### 1.2 Polymeric Actuators

Polymer actuators constitute a very important class of materials capable of modifying their shape in response to changes in the environment, such as temperature, pH, light, electrical/magnetic field, and/or chemical additives, among others.^73−75^ A key challenge in this field is to develop robust actuators with programmable motion and high strain density.^76−81^ Such materials would be easy to produce, mold, cut, and three-dimensional (3D) print while generating large macroscopic actuation at relatively low energy input. Similar to self-healing materials, many polymeric actuators resemble biological soft actuators that display a diversity of motion.^82^ Indeed, both plants and animals possess complex actuation systems controlled mainly by two types of actuators, muscle cells for animals^83^ and turgor-driven cells for plants,^84^ that transform chemical energy into mechanical output. The muscle cells contract or stiffen when activated, while the turgor cells undergo volume expansion or shrinkage by autonomous or voluntary excitation. Although these actuators are controlled by the nervous systems or signaling molecules, there are some examples in plants where the motion depends on the passive deformation of hygroscopic materials at different humidity conditions.^85^ Along this line, another sophisticated natural process is the opening of a pinecone to release seeds at low humidity conditions.^86^ These systems consist of two macroscopic layers of tissues with different degrees of hygroscopic swelling, the scales of pinecone bend outward as the outer layer shrinks greater under decreasing humidity. The swelling ability of the two layers is controlled at the cellular level through a specific arrangement of cellulose microfibrils. It is very important to realize that cell-level actuators are shaped, assembled, and structures well-arranged in a hierarchical manner, which govern their macroscopic actuation efficiency.^85^ From the mechanical standpoint, the actuation is controlled by the stress and strain distribution at various structural levels of well-organized components within the corresponding supporting matrices.

Within this scenario, many researchers are continually looking for inspiration in biological systems to create new generations of automaton soft actuators (also referred as soft robotics) with high degrees of freedom and mechanical properties closer to natural actuators compared to conventional rigid actuators (e.g., electric motors). The advantages of lightweight soft actuators include, among others, flexibility, safety in interaction with humans (e.g., healthcare applications), availability of a wide range of materials and mechanical properties, and use of various stimuli for the actuation. Due to their softness and compliance, soft actuators are widely used in different applications such as packing, food processing, microfabrication, robotics, lab-on-a-chip systems, etc.^74,87,88^ Since the pioneering work reporting an aircraft electric actuator in 1944,^89^ more than 150 000 scientific papers have been dedicated to the study of actuators, which proves the great interest aroused by these materials.

Most soft actuators are made of synthetic polymers mainly due to their great tunability, as they can be soft and hard depending on their chemical and physical structure.^90,91^ Furthermore, the large variety of different physical and chemical inputs enables a broad range of strain, stress, and conformations compared to traditional ceramic or metallic based actuators.^92^ In terms of the fabrication techniques,^77^ they usually begin from the liquid form such as melts or solutions, followed by solidification through cooling, solvent evaporation, and/or curing (i.e., cross-linking). In general, one-dimensional (1D), two-dimensional (2D), and 3D structures can be fabricated by extrusion, casting, and various molding techniques, with a range of structural characteristics and features from nano to macro levels. Moreover, more complex shapes such as hollow inner structures can be achieved in one single step by 3D printing.

**Figure 2:**
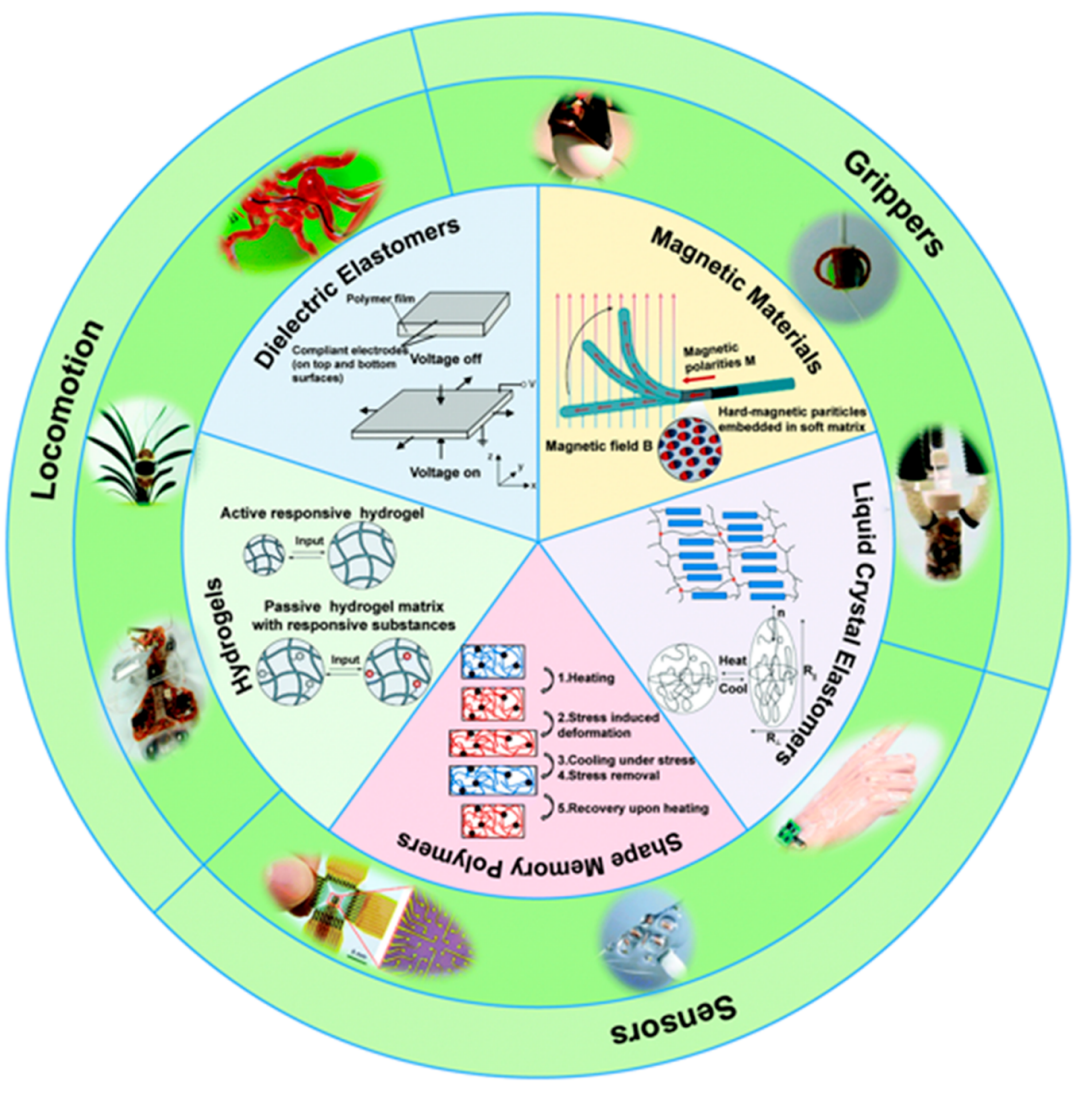
General overview of typical stimuli-responsive materials and their applications for soft robotics. Reproduced with permission from ref (93). Copyright 2020 Royal Society of Chemistry.

Exploring the literature over the last decades, there are a large number of examples of soft actuators^93^ based on shape-memory polymers^94−97^ and electro-/magneto-active^1,98−104^ polymers, dielectric elastomers,^76,105−109^ liquid crystal elastomers,^110−113^ and hydrogels,^114−118^ among others^119^ (Figure 2). In this sense, electric field, magnetic field, light, and temperature are the most common stimuli employed for the actuation.^120,121^

### 1.3 Scope of This Review

**Figure 3:**
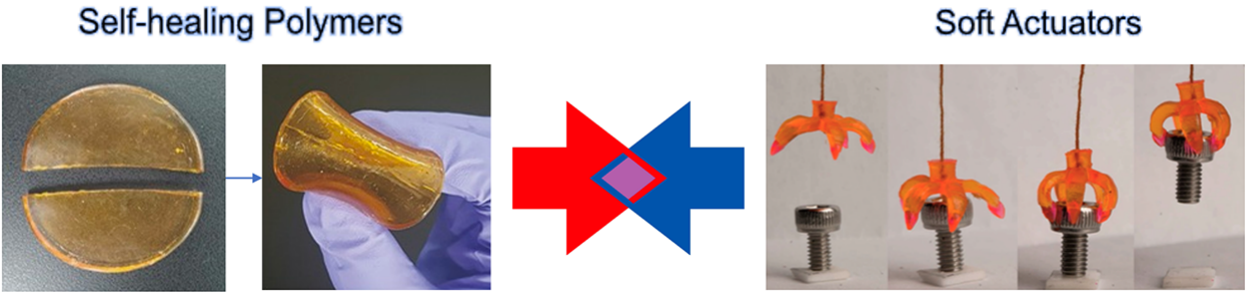
Scope of this review: Merging self-healing polymers with soft actuators. (left) Adapted with permission from ref (123). Copyright 2018 John Wiley and Sons. (right) Adapted with permission from ref (124). Copyright 2016 Authors, Springer Nature.

Despite all OF the advantages of soft materials, they are more prone to damage, especially in dynamic and arbitrary circumstances. Thus, their common limited lifespan may lead to unsustainable future applications.^122^ Thus, in an attempt to increase the reliability of soft actuators for practical applications, it would be extremely useful to provide polymeric soft actuators with self-repairing capabilities to continue operating with unchanged performance after a damage or breakdown. Thus, with this review we focus on a marriage of convenience between the best of the two worlds, self-healing polymers and soft actuators (Figure 3), which should also endow the final materials with environmental friendliness, cost-saving, enhanced safety, and life prolonging by maintaining structure and functionality after a damage. In order to better define the scope of this review, we have concentrated our efforts toward the identification of self-healing soft actuators that display a macroscopic movement under certain environmental conditions. Thus, those examples of self-healing polymers without visual movement or soft actuators without self-healing capacity are out of the scope of this review. Representative literature for those examples is distributed in the previous introductory sections. In addition, typical swelling/deswelling behavior of hydrogels (e.g., used for drug delivery applications) was not considered within the macroscopic actuation. Regarding the use of external stimuli associated with the functionality of the materials, a large part of the review is centered in photo, magnetic, thermal, and electric actuators, those being examples clearly the most abundant in the literature. A few additional instances dealing with pH-, mechanical-, and redox-based actuation are also included in the last section. In order to provide a temporal context to the field, we have provided a chronological discussion of the development of the different materials within each section based on the external stimuli employed. We present a discussion of the major achievements by discussing the key experimental details that are critical for a given system, potential limitations, and mechanistic insights for both the self-healing and the actuation processes. Finally, we have compiled the contributions in a general table, providing at first glance the nature of the external stimuli, conditions for self-healing and actuation, key information about the driving forces for both phenomena, and the most important features of the achieved movement. We believe this table will be very useful for all researchers interested in soft actuators with self-healing capacity.

## 2 Self-Healing Polymeric Actuators

### 2.1 Self-Healing Photo-Actuators

**Figure 4:**
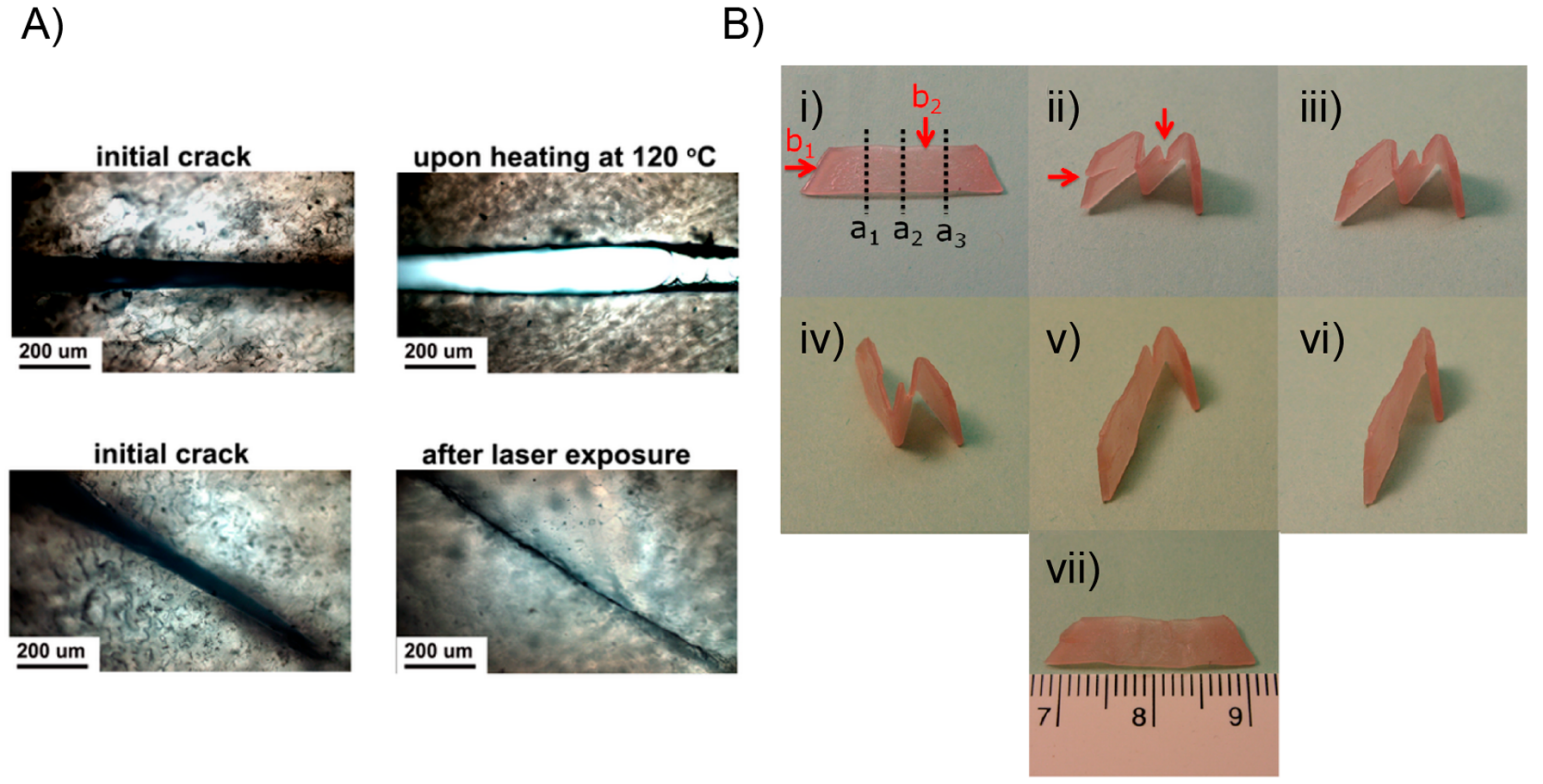
(A) Difference between bulk heating and light-induced local heating on the healing performance. (B) Sequentially triggering the optical healing and the light-controlled shape recovery process for a film of cross-linked PEO/AuNP. (i) Original film with the permanent shape; (ii) temporary shape obtained by folding the film along the lines a_1_, a_2_, a_3_ at 80 °C followed by cooling to room temperature, then two cuts were made as indicated by red arrows (b_1_ and b_2_ in photo (i); (iii) the b_1_ cut was healed by exposing the crack to laser (12 W/cm^2^) for 5 s; (iv) the first unbending after 10 s laser scanning along the fold a_1_ at a power of 6 W/cm^2^, followed by the second unbending under the same condition along the fold a_2_; (v) the other cut b_2_ remained in the film of an intermediate temporary shape; (vi) the cut b_2_ was optically healed under the same condition as for the cut b_1_; (vii) the third light-triggered unbending along the fold a_3_ completed the permanent shape recovery. Adapted with permission from ref (125). Copyright 2013 American Chemical Society.

In 2013, Zhang and Zhao reported the first polymer-based nanocomposite exhibiting both shape-memory and self-healing properties triggered by light stimuli which, in addition, can be activated independently and sequentially in the same material.^125^ This material, under light irradiation, was able to recover its initial shape in a fast-manner after suffering folds or unidirectional stretches, as well as heal from cut-through cracks. Experimentally, the design of the material involved the inclusion of small amounts of Au nanoparticles (AuNPs) (0.003 wt % (wt %)) into chemically cross-linked poly(ethylene oxide) (PEO) matrices, presenting crystalline domains capable of performing reversible melting phenomena. To achieve a good nanoparticle dispersion, AuNPs with an average diameter of 10 nm were decorated superficially with PEO brushes and added into a mixture of APS, *N*,*N*,*N*′,*N*′-tetramethylethylenediamine (TMEDA), and a linear PEO derivative with both ends modified with acrylate structures. Films of reticulated PEO/AuNP were prepared by exposing the above mixture to a curing process performed at room temperature and 60 °C, consecutively, for a total time of 4 days. The authors devised the inclusion of AuNPs motivated by the photothermal effect that arises from the surface plasmon resonance phenomenon that these entities experience under light irradiation.^126−129^ Thereby, during light irradiation, local heat is generated at the surroundings of AuNPs, which is later dissipated through the polymer matrix allowing the tuning of the local temperature with respect to the melting temperature of the semicrystalline reticulated material. Therefore, by inducing melting and recrystallization processes inside the material, both shape recovery and healing phenomena can be activated. In terms of the light-controlled shape-memory property, the achieved material was able to completely recover from a 400% stretch deformation. The initial material was stretched unidirectionally at 80 °C (temperature (*T*) > melting temperature (*T*_m_)) and rapidly cooled at room temperature to fix the temporary shape. Then, using a laser source (530 nm, 7 W/cm^2^) and irradiating different areas of the material, it was possible to return it to its initial dimensions. Moreover, authors were able to demonstrate the on–off behavior of this process, along with its dependence on the laser intensity which turns out to be directly related to the local temperature generated. Regarding the self-healing capability triggered by light, the material showed an outstanding ability to recover from a cut-through crack created with a razor blade (Figure 4A). Surprisingly, by exposing the damaged area to a laser (13 W/cm^2^) during 3 s at room temperature, a single piece was obtained which was able to stand >14000 times its own weight, ensuring a correct and efficient healing property. As a complement to the above, tensile tests were carried out, showing that healed samples gained about 62% of its original tensile strength. Authors explained the healing process through a mechanism where polymer chains present in the melted portion of the material can re-entangle between each other and then fix their positions during the recrystallization of the material. However, based on the above, the authors found an unexpected result when they tried to carry out the healing process exposing the bulk material to temperatures above *T*_m_, finding that the obtained samples broke easily in the damaged area, and therefore, indicating the absence of healing capacity. This allowed them to corroborate the importance of the localized heat generated around AuNPs under light irradiation to achieve a successful self-healing property. The above because when the whole sample is heated, the thermal expansion experienced by the bulk material results in an expansion outward that prevents the contact between the fractured surfaces hindering the polymer chain interdiffusion.^130^ Additionally, it is worth noting that neither shape-memory nor self-healing properties activated by light were observed in materials without presence of AuNPs. Finally, the authors corroborated the independence of both phenomena by inducing, in an alternate manner, the healing of cuts and reshaping of folds exerted on a poly(ethylene glycol) (PEG)/AuNP film (Figure 4B). In this sense, shape-memory and self-healing processes were individually and sequentially triggered within the same material depending on where the light source was focused. Furthermore, light irradiation would activate both processes if the crack is positioned in a bent region. The excellent results reported by this work served as a basis for the beginning of the development of new photoactuators also exhibiting light-driven self-healing ability. Regarding the above, the authors left open the possibility of complementing their results by evaluating the effect of nanoparticles’ size, morphology, and nature on the photothermal effect and, therefore, on the actuation and healing properties. In addition, the substitution of photothermal agents based on noble metals with other less expensive materials would be attractive from an economic point of view.

**Figure 5:**
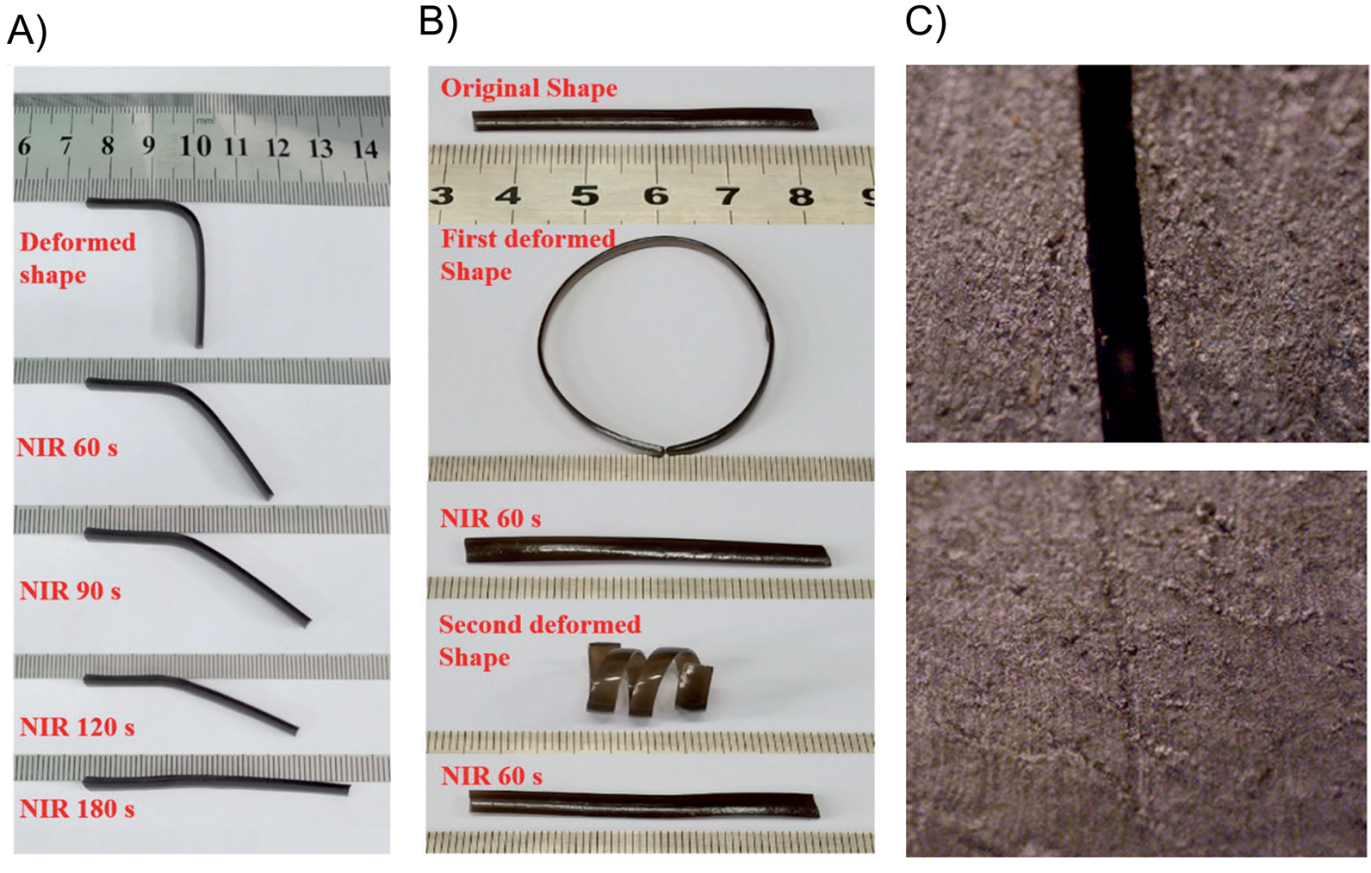
(A,B) Photos showing the shape recovery behavior of the PVA–PDAP hydrogel under NIR irradiation (0.75 W/cm^2^). (C) Optical microscopy photos of the cut surface before (up) and after (bottom) NIR irradiation (output power: 0.75 W/cm^2^, irradiation time: 30 s). Adapted with permission from ref (131). Copyright 2017 John Wiley and Sons.

The self-healable photoactuator field had to wait around four years for its development to continue, where, in 2017, Yang and co-workers prepared a hydrogel based on poly(vinyl alcohol) (PVA) and polydopamine particles (PDAPs), which, under near-infrared light (NIR), exhibited ultrafast shape-memory and self-healing properties.^131^ The rapid light-responsiveness showed by this material was attributed to the photothermal effect induced by PDAPs.^132,133^ In addition, this material stood out by its excellent mechanical properties and biocompatibility, having outstanding potential in biomedical applications such as tissue engineering, artificial skin and arthrodial cartilage. By following an easy and straightforward protocol, authors were able to synthesize microsized PDAPs through the oxidative polymerization of dopamine.^134^ Then, the as-prepared spherical PDAPs with an average diameter size of 330 nm were included and well-dispersed into PVA solutions. Lastly, PVA–PDAPs hydrogels from above mixtures were obtained after several freezing/thawing cycles. Two different processes contributed to the physical cross-linking within the PVA–PDAP hydrogels; the first one, reversible in character, was assigned to PVA crystalline domains, while the second was attributed to hydrogen bonding occurring between PVA and PDAPs which act as a fixed phase. Indeed, thanks to the existence of hydrogen bonds between both parts, an adequate dispersion of PDAPs was achieved inside hydrogels, affording a notable enhancement of the mechanical properties of the final material. Regarding the above, and compared to a neat PVA hydrogel, authors demonstrated that the addition of a small amount of PDAP particles (2 wt %) allowed an abrupt increase of the Young’s modulus (from 0.47 to 0.83 MPa), the elongation at break (from 322% to 452%), and the tensile strength (by about 4.3 times) of the material. Therefore, PDAP entities not only allow the light-driven shape-memory and self-healing processes but also contribute to the stability of the cross-linked network. Due to the cross-linked nature of PVA–PDAP hydrogels, these materials exhibited shape-memory response. First, the authors tested this property without light stimuli by stretching a sample up to 100% elongation deformation. Then, they perform freezing/thawing cycles, maintaining the strain to induce the PVA crystallization and form a new crystalline phase that fixed the shape of the sample (corroborated by differential scanning calorimetry (DSC) and X-ray diffraction (XRD) measurements). The elongated material exhibited a complete shape recover ratio of around ≈100% when it was exposed to temperatures above the *T*_m_ of PVA (50–60 °C). The above encouraged to authors to test the shape-memory property of the material but now under NIR irradiation, where PDAP particles exhibit photothermal effect. Surprisingly, authors found that samples even with a low content of PDAP (0.5 wt %) and under a very low NIR light source (808 nm, 0.25 W/cm^2^) showed a notable photothermal effect evidenced by an ultrafast temperature rise. In this sense, samples containing a 2 wt % of PDAP reached temperatures as high as 140 °C in around 20 s during their irradiation with a NIR source of 1.5 W/cm^2^ power intensity, corroborating that under these conditions the *T*_m_ of PVA is easily surpassed. Therefore, the NIR-activated shape-memory property of PVA–PDAP hydrogels was successfully corroborated by showing that samples fully recover their initial shapes after being deformed by simple bending (Figure 5A) or also molded into more complex structures such as circles and spirals (Figure 5B). The above experiments were carried out by irradiating samples with a NIR-light source with an intensity of 0.75 W/cm^2^ during 60 s, allowing the shape recovery process to be considered as fast. In addition, authors also demonstrated that the recovery ratio increases with increasing the NIR intensity from 0.25 to 1.5 W/cm^2^ and the on–off nature of the process because the process was completely stopped once the irradiation was halted. The mechanism involved in the shape-memory process is attributed to the melting-crystallization phenomena occurring within the material during NIR irradiation. The ability of PDAPs to convert NIR light into heat, and its subsequent dissipation along the matrix, allows the sample temperature to be rapidly increased above its *T*_m_. Thus, after achieving the fusion of the crystal lattice, the hydrogel recovers its initial shape by releasing the excess energy stored in the form of structural tension. This mechanism was corroborated by the authors by performing XRD measurements to samples before and after being irradiated, where after 10 s of irradiation (0.75 W/cm^2^) samples did not show peaks associated with PVA crystalline phases. The above demonstrates that the shape-memory ability showed by these hydrogels is due to the melting of crystalline PVA phases. On the other hand, these materials also showed self-healing capacity attributed to the existence of hydrogen bonds between PVA and PDAPs. This property was studied by cutting a sample in two pieces and bringing them together to then being irradiated during 30 s using a NIR source (0.75 W/cm^2^). The healing process, qualitatively, was successfully observed by optical microscopy (Figure 5C), however, in order to study this process from a more quantitative perspective, the authors evaluated the self-healing property of samples by means of tensile tests. Healing efficiencies were calculated from the ratio of strength at the break of healed and original samples. Regarding the above, after being irradiated for 30 s, PVA–PDAP hydrogels containing 2 wt % of PDAPs revealed a healing efficiency of 94%. This value is notably higher than the one calculated for a sample healed at 37 °C during 36 h (24%). From the above, it corroborated the importance of the NIR light conversion into heat by PDAP, which would allow the local rise of temperature around the damaged region, promoting the thermally activated PVA chains motions and the reformation of hydrogen bonds. In addition, authors also checked the healing efficiency of samples after multiple damage-healing cycles, showing that materials can be repeatedly healed exhibiting healing capacity values above 85% even after fourth cycles. Finally, aiming to introduce this type of systems into biomedical fields, biocompatibility assays were performed in terms of cytotoxicity evaluation, where no significant difference were found between bare PVA, PVA–PDAP hydrogels, and the control experiment, indicating that PVA–PDAP does not inhibit the cell growth.

**Figure 6:**
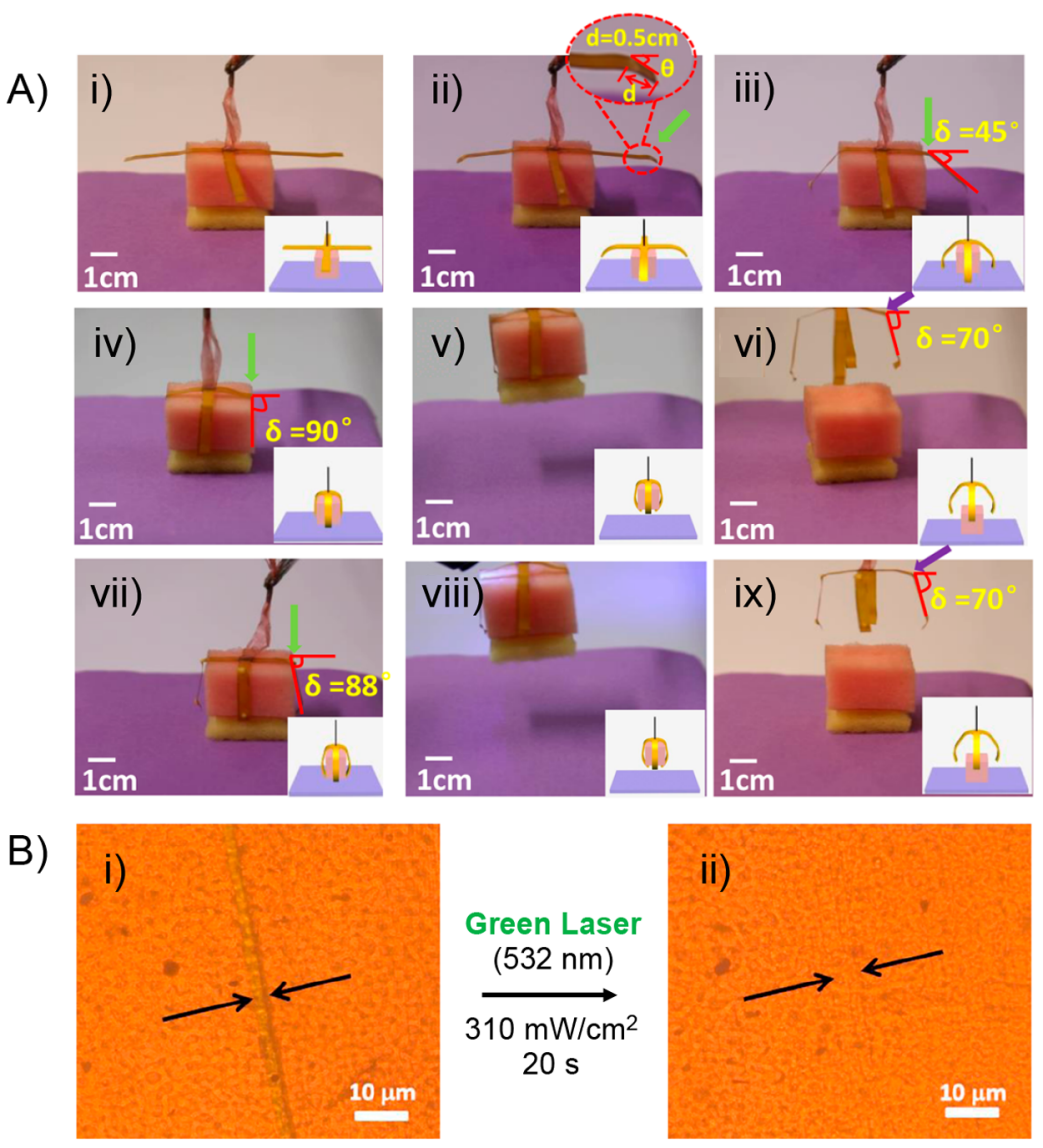
(A) Photographs showing the cyclic process of grabbing and releasing an object by the optically actuated manipulator arms. (i) Manipulator arms crossed at the center were held vertically with their center tied with a rope. (ii–iv) Finger bends with a knuckle induced by the green light. (v) The object grabbed by the finger is lifted. (vi) Fingers releases the object induced by UV light. (vii–ix) The above process is repeated. (B) Optical microscopic images of a scratched sample before (i) and after (ii) the exposure to a green laser. Adapted with permission from ref (135). Copyright 2018 American Chemical Society.

A year later, Si and collaborators achieved the preparation of a photoresponsive supramolecular polymer-based system exhibiting both light-induced actuation and self-healing ability.^135^ This work can be considered as the first report in which the above-mentioned photoactuation property is not ascribed to photothermal effect but to the well-known light-triggered trans–cis isomerization of azo structures.^136^ Due to the above, the obtained material can be considered a “truly” light-stimulated system showing a controllable and reversible shape-memory property, along with a fast self-healing capability. Furthermore, on the basis of the excellent optical-actuation performance shown, the authors were able to successively grab and release an object using this material in an “arm” configuration, expanding the scope of work into the field of advanced microrobotics. First, authors carried out the synthesis of a poly(acrylic acid) (PAA) graft copolymer bearing 2-ureido-4[1*H*]-pyrimidinone (UPy) units as pendant groups (PAA-u), which then was dissolved together with 3,3′,5,5-azobenzenetetracarboxylic acid (t-Azo). This mixture was successively heated, cooled at room temperature, and stirred overnight before being deposited, through the dip-coating technique over a glass substrate. The above system was irradiated under ultraviolet (UV) light during 48 h, after which a PAA-u/t-Azo film was peeled off from the substrate. The structural stability of this material was attributed to the presence of multiple hydrogen bonds serving as cross-linking points within the polymer matrix. In this regard, the system can be considered a supramolecular assembly. The authors successfully corroborate the presence of this type of interaction by means of Fourier transform infrared spectroscopy (FTIR) and DSC analysis, while the higher mechanical strength exhibited by PAA-u/t-Azo, compared to PAA and PAA-u films, served also as indirect evidence of its cross-linked structure. Nonetheless, authors declared that hydrogen bonds not only are responsible of the structural stability of the material, but also allow stabilization of the azo *Z*-isomers formed during the irradiation process.^137^ It has been reported that the photoactuation property of azo-polymers depends largely on the ratio of *E*/*Z* isomers (usually dominated by the *E*-isomer).^12,138,139^ In this sense, the increase of the *Z*-isomer population inside the material has been adopted as a viable strategy to improve its light-actuation property. The above was the primary motivation to conduct the materiaĺs formation under UV light, inducing the *E* to *Z* isomerization process of t-Azo entities, which then, thanks to H-bonds, some of them would find a suitable environment to preserve their *Z* conformation. The success of the strategy was demonstrated by ultraviolet–visible (UV–vis) spectroscopy by tracking the changes of the band assigned to π–π transitions of t-Azo moieties, from which a 25% of *Z*-isomers in the final material was calculated. This value was 10% higher than the one obtained for the pristine t-Azo. The efficient stabilization of *Z*-isomers in the PAA-u/t-Azo assembly was also demonstrated by their notable half-life values (120 h in dark), being longer than in many previous studies.^140−148^ Surprisingly, the authors demonstrated the high reversibility of the photoisomerization process by carrying out up to 100 irradiation cycles alternating green and UV light, without observing deterioration of the property. Regarding the above, the *Z*-to-*E* isomerization induced by green light irradiation allowed reduction from 25% to 12% the amount of *Z*-isomerin 60 s, whereas the *E*-to-*Z* isomerization using UV light achieved an increase up to 27% in around 240 s. After ascertaining the *E*/*Z* isomerization in the PAA-u/t-Azo film, its light-driven actuation property was evaluated. To achieve this, a strip of film was exposed to a green laser, successfully achieving a controlled photoinduced bending-like motion. This optically conducted bending allowed the deformation of the material into different shapes that remained stable after the cessation of irradiation. The magnitude of the bending deformation was highly dependent on the irradiation time, while the rate of the process was efficiently modulated by varying the intensity of the light source. The bent film did not recover its initial form even after 480 h, ascribing this result to the high stability of the *E*-isomer within the assembly matrix. Then, using ultraviolet light, the initial shape of the strip was recovered by inducing bending deformations in the opposite directions to those achieved with green light. The photobending property displayed by the films was tested over 100 consecutive cycles, revealing excellent reversibility as well as demonstrating the robustness of the photoactuator. The observed deformation would be induced by intrinsic stretching and contraction forces arising from the isomerization of t-Azo units. Based on the above, when the material is exposed to light irradiation, those azo entities present on the irradiated surface are more available to carry out light absorption than the ones present on the back surface. Considering this, during the irradiation process, t-Azo units in the illuminated surface should exhibit a faster and higher degree of isomerization than those at the back, thereby the unequal *Z*-to-*E* isomerization rate along the material would generate stretching forces causing the material’s deformation. The same explanation can be applied to the shape recovery achieved under UV light irradiation, where the *E*-to-*Z* isomerization process would generate contraction forces along the material. Interestingly, the authors devised a method to measure these stretching and contraction driving forces exerted by the material. The stretching driving forces, emerged under green light irradiation, fall between the range of 3.2 × 10^–6^ to 1.7 × 10^–5^ N, where the greatest values were obtained under higher irradiation intensities. Similarly, the contraction forces achieved by UV irradiation exhibited values between 1.6 × 10^–6^ and 1.1 × 10^–5^ N. Motivated by the above results, the authors successfully attempted to fabricate a photoactuated manipulator arm using two strips of PAA-u/t-Azo film disposed into a cross shape, simulating a “hand” configuration (Figure 6A). Then, each strip (“finger”) was consecutively irradiated with a green laser stimulating its bending process. The bent configuration was able to grab an object which afterward was lifted and maintained for a period of 480 h. After that, using a UV light source, the release of the object was induced by recovering the initial shape of the “fingers”. The authors demonstrated the reversibility of the process by performing 40 successively grab–release cycles alternating between green and UV irradiation. Additionally, PAA-u/t-Azo films exhibited an outstanding light triggered self-healing property, which was developed during the bending process activated under green laser irradiation. Before activating the bending motion, the strip was damaged with a scratch using a razor blade. Initially, the self-healing process was monitored by optical microscopy in which after 20 s of green light irradiation (310 mW/cm^–2^) the scratch disappeared completely (Figure 6B). However, a more in-depth investigation regarding the healing ability of the material was carried out by performing stress–strain tests for the original sample and its healed counterpart. These experiments revealed that the healing property increases with the light exposition time, recovering up to 98% of the tensile strength after 20 s of irradiation (25.5 and 25 MPa for the original and healed sample, respectively). The light-induced self-healing process was further investigated by inducing more severe damage to the sample, that is, cutting it in two. In this case, the initially bisected sample recovers around 94–97% of its original tensile strength after being irradiated with green light. In addition, the authors demonstrated that the healing ability can be notably improved by irradiating the sample with higher intensities or longer times. The excellent and rapid self-healing property exhibited by PAA-u/t-Azo has been attributed to the multiple hydrogen bonds, reversible in nature, existing within the structure. During irradiation, besides inducing the bending of the material, the local temperature of the illuminated area increases to around 72 °C according to the obtained high-resolution infrarred image. On the other hand, by means of FTIR spectra recorded at different temperatures, the authors demonstrated that hydrogen bonds in the sample are weakened as temperature increases while they are efficiently reformed during the cooling process. Therefore, the proposed light-induced self-healing mechanism for this material would be associated with the local heating of the damaged zone due to irradiation, prompting the temporal dissociation of H-bonds that later could be reformed by connecting areas separated by the fissure. In addition, the light-induced bending actuation could also be involved by facilitating the contact between the damaged areas. It is important to mention that most of the supramolecular polymer assemblies exhibit thermally induced self-healing properties, activated after being heated above their glass transition temperature (*T*_g_). In those cases, the healing mechanism mainly consists of the interpenetration and re-entanglement of polymeric chains facilitated by their increased mobility. However, because the temperature achieved in PAA-u/t-Azo (65–80 °C) did not surpass its *T*_g_ value (106 °C), the mechanism would be mostly attributed to H-bond reformation. Lastly, the self-healing performance revealed by PAA-u/t-Azo films was notably superior to most of the previously reported polymer-based systems in terms of healing efficiency, temperature requirements, and healing time.

Within the field of self-healing photoactuators, the material designed by Si et al. is undoubtedly one of the best exponents but also unique in terms of design. In reference to the above, the lack of reports related to the development of self-healing photoactuators, whose actuation is achieved by the isomerization of molecular entities is not a coincidence. Indeed, the development and understanding of these types of systems is usually more challenging in terms of synthetic nd characterization procedures. Due to the above, the current literature reveals that this field has been widely dominated by materials whose actuating motions and self-healing capabilities are triggered by the photothermal effect. This could be attributed to the greater simplicity with which these types of systems can be prepared and studied. Notwithstanding the above, the use and implication of other types of isomerizable structures, aiming to replace the already widely studied azo compounds, should be the next step in the area.

**Figure 7:**
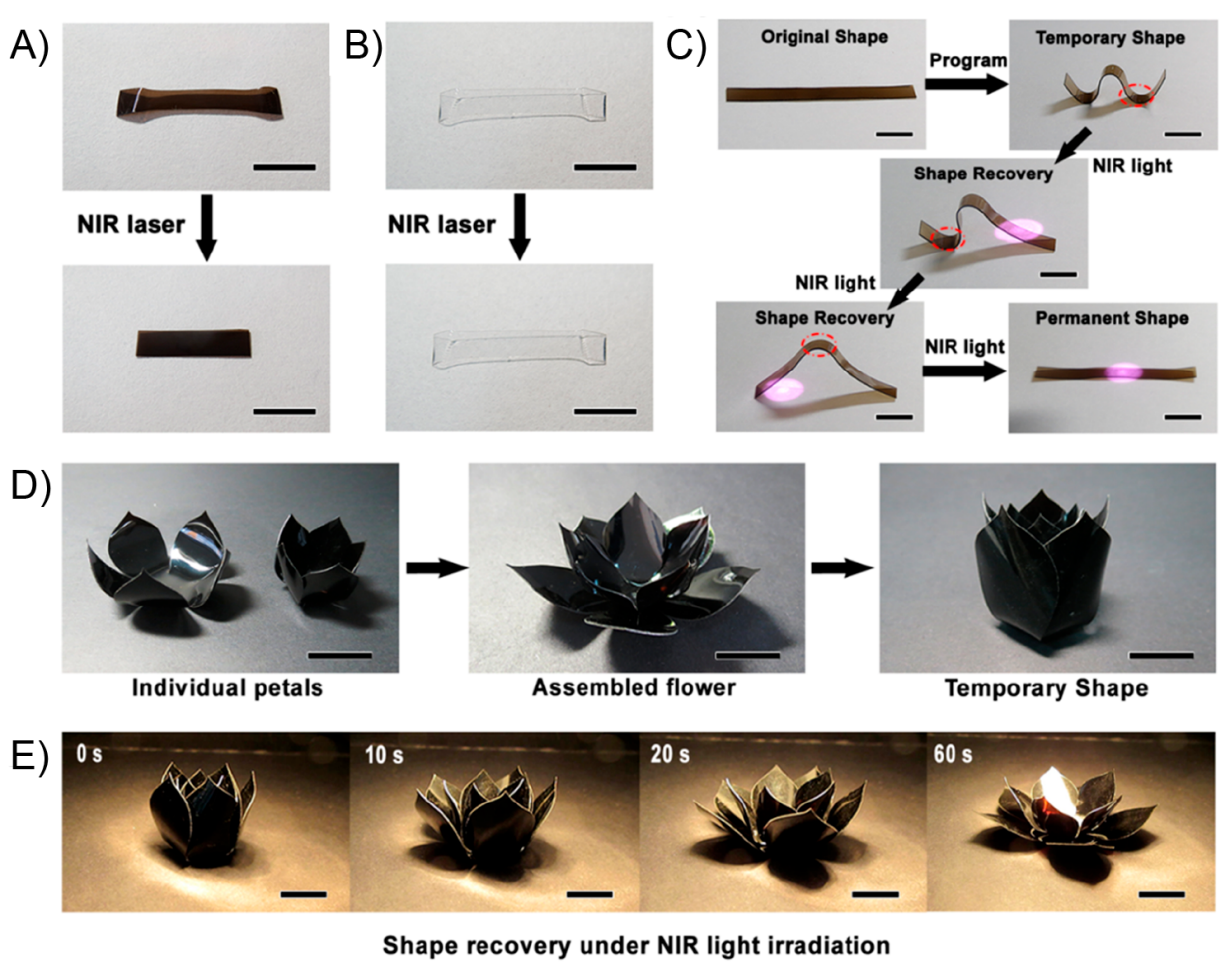
(A) NIR-light induced shape recovery of the PVA/PAA-GO_3%_ and (B) PVA film. (C) Sequential shape recovery of the W-shaped PVA/PAA-GO_3%_ film induced by NIR light irradiation. (D) Fabrication process of the PVA/PAA-GO_3%_ flower that can bloom under NIR light irradiation. (E) Time-sequence images of the PVA/PAA-GO_3%_ flower that is blooming under NIR light irradiation. The scale bars in all panels are 1 cm. Adapted with permission from ref (149). Copyright 2019 American Chemical Society.

Regarding the preparation of novel photothermal actuators, at the beginning of 2019, by dispersing poly(acrylic acid)-grafted graphene oxide (PAA-GO) into PVA matrix, Li and collaborators carried out the preparation of a robust polymer network stabilized by multiple hydrogen bonds, exhibiting an excellent NIR-light-induced shape-memory property, attributed to the photothermal effect caused by the presence of GO.^149^ In addition, due to the reversible nature of hydrogen bonding,^150^ PVA/PAA-GO films displayed an outstanding self-healing property assisted by water, which also endowed this material with the ability to repair the fatigue shape-memory function. Based on the obtained results, the authors aimed this material to be potentially used as actuators in biomedical devices and flexible electronics, among others. In the first place, the authors carried out the preparation of PAA-GO thought redox-initiated graft polymerization of PAA onto GO nanosheets in aqueous media. The success of the polymerization was corroborated by means of Raman spectroscopy, while the content of PAA in PAA-GO (≈ 28%) was determined by thermogravimetric analysis (TGA). Then, PVA/PAA-GO_x%_ films containing 1, 2, 3, and 4 wt % of PAA-GO were prepared through the solvent-casting technique from PVA/PAA-GO aqueous mixtures. Uniform films were obtained thanks to the improved dispersibility exhibited by PAA-GO. FTIR spectra recorded at different temperatures allow demonstration that hydrogen bonds between carbonyls of PAA and hydroxyl groups from PVA would be the main type of interactions taking place within the polymer matrix. Stress–strain curves measured for PVA/PAA-GO_x%_ samples revealed a similar behavior to pristine PVA, however, the yield stress and fracture stress values were dramatically increased after the addition of PAA-GO, while the opposite trend was observed for the elongation at break values. Overall, the presence of PAA-GO in PVA matrix significantly increases the mechanical strength of samples, permitting them to be repeatedly deformed without evidence of crack formation. Therefore, the thermally induced shape-memory of these samples was first evaluated by deforming a flat film strip at temperatures well above its glass transition temperature (*T*_g_ + 20 °C), followed by cooling to room temperature in order to fix the new temporary shape. Because the *T*_g_ of the material is considerably higher than room temperature, samples were able to maintain their temporary shape at ambient conditions. Then, when the deformed strip was heated again above *T*_g_, a complete recovering of the original shape was afforded in 30 s, corroborating the thermally induced shape-memory property. The shape recovery ratio (*R*_r,t_) of pristine PVA films decreases from 97.5 ± 0.4% to 80.2 ± 0.3% after 15 consecutive folding/recovery cycles. Surprisingly, after being exposed to the same mechanical stress, *R*_r,t_ values for PVA/PAA-GO_x%_ containing 1, 2, 3, and 4% went from 98.2 ± 0.2, 98.6 ± 0.3, 99.6 ± 0.4, and 99.7 ± 0.2% to 87.4 ± 0.4, 88.6 ± 0.2, 90.6 ± 0.4, and 90.7 ± 0.3%, respectively. Authors argued that the greater drop of the *R*_r,t_ for the PVA film should be ascribed to a higher disentangling and glissade of polymer chains during bent/unbent cycles. Conversely, thanks to the formation of hydrogen bonds between PAA-GO and PVA, the disentanglement and/or slippering of polymer chains would be diminishing, avoiding the abrupt decrease of *R*_r,t_ values. Afterward, the NIR-triggered shape-memory ability of PVA/PAA-GO_3%_ sample was evaluated, first, by elongating a strip under heating and then fixing this temporary shape by cooling to room temperature. Figure 7A shows that this sample was able to completely recover its original shape in 35 s of NIR-light irradiation (808 nm, 1.3 W/cm^–2^) thanks to the outstanding photothermal property provided by GO, which allowed the sample to increase its temperature up to 75.4 °C (*T*_g_ + 21.4 °C) in only 5 s. It is worth noting that no shape-memory property was observed for a pristine PVA film after being exposed to the same experimental protocol (Figure 7B). Moreover, an initially flat strip sample, deformed into a W-shape, was able to recover its original shape by sequentially irradiating different areas of the film with NIR-light (Figure 7C). The authors went further by testing the shape-memory property on samples arranged in more complicated structures, in this case, a petal-shaped PVA/PAA-GO_3%_ film (Figure 7D). This sample, initially configured as an open flower, was deformed into a bud at 74 °C and fixed after cooling at room temperature. Then, after 1 min of NIR irradiation (47 mW cm^–1^), the sample emulated the bloom motion (Figure 7E). PVA/PAA-GO_3%_ samples also exhibited the ability to heal in environments with high relative humidity (RH). A film strip was cut into two pieces, after which several drops of water were deposited on the damaged surfaces. Then, the two pieces were brought together to start the healing process. The authors observed an optimal adhesion between the parts at 5 min of initiated the contact and an evident heal of the cut after 5 h. However, the scar of the damaged remained visible under SEM observation. Stress–strain curves of the healed sample showed a similar value for the yield stress regarding the original sample (71.4 MPa), while lower values for the fracture stress (61.2 MPa) and elongation at break (152.4%) were obtained after the healing process. However, to demonstrate the efficiency of the healing, an additional sample, purely consisting of PVA and GO, were exposed to the same damage and healing protocol, giving as a result a sample that easily fractures in the affected zone during bending. The above result corroborated the importance of PAA in the self-healing ability displayed by these materials. Because the healing property was activated by the presence of water, authors argued that one of the main functions of PAA was to increase the water absorption (swelling ability) of the material. In this sense, after water adsorption, it is expected that hydrogen bonds within the matrix would be weakened, resulting in a less restricted environment for the polymer chains mobility, which, after inducing the contact between the fractured fragments, the re-entanglement and reformation of interactions between PVA and PAA-GO would allow the healing of the material. More importantly, in this work, authors demonstrate that the self-healing ability is not only limited to healing mechanical damage but also healing the fatigued shape-memory function. Regarding the above, when a piece of PVA/PAA-GO_3%_ was exposed to 12 folding/recovery cycles its *R*_r,t_ value was calculated as 92.9 ± 0.4%, however, after being healed for 6 h under 90% RH conditions, this value notably increased up to 99.5 ± 0.3%. Repeating the above experiment, but now after 60 folding/recovery cycles, the *R*_r,t_ value jumped from 92.6 ± 0.2 to 99.8 ± 0.3%. Authors explained that a possible mechanism for the healing process of the fatigued shape-memory function could be, again, addressed to the absorbed water thanks to the hygroscopic nature of PAA, where water can act as plasticizer enhancing the chains mobility inside the matrix, facilitating the transition from a high-energy stretched configuration to a more relaxed coiled state. Author also evidenced that the crystallinity degree of PVA in PVA/PAA-GO after being exposed to 90% RH conditions was lower than in a PVA film, serving as additional argument to support the idea of a higher chain mobility in PVA/PAA-GO.

**Figure 8:**
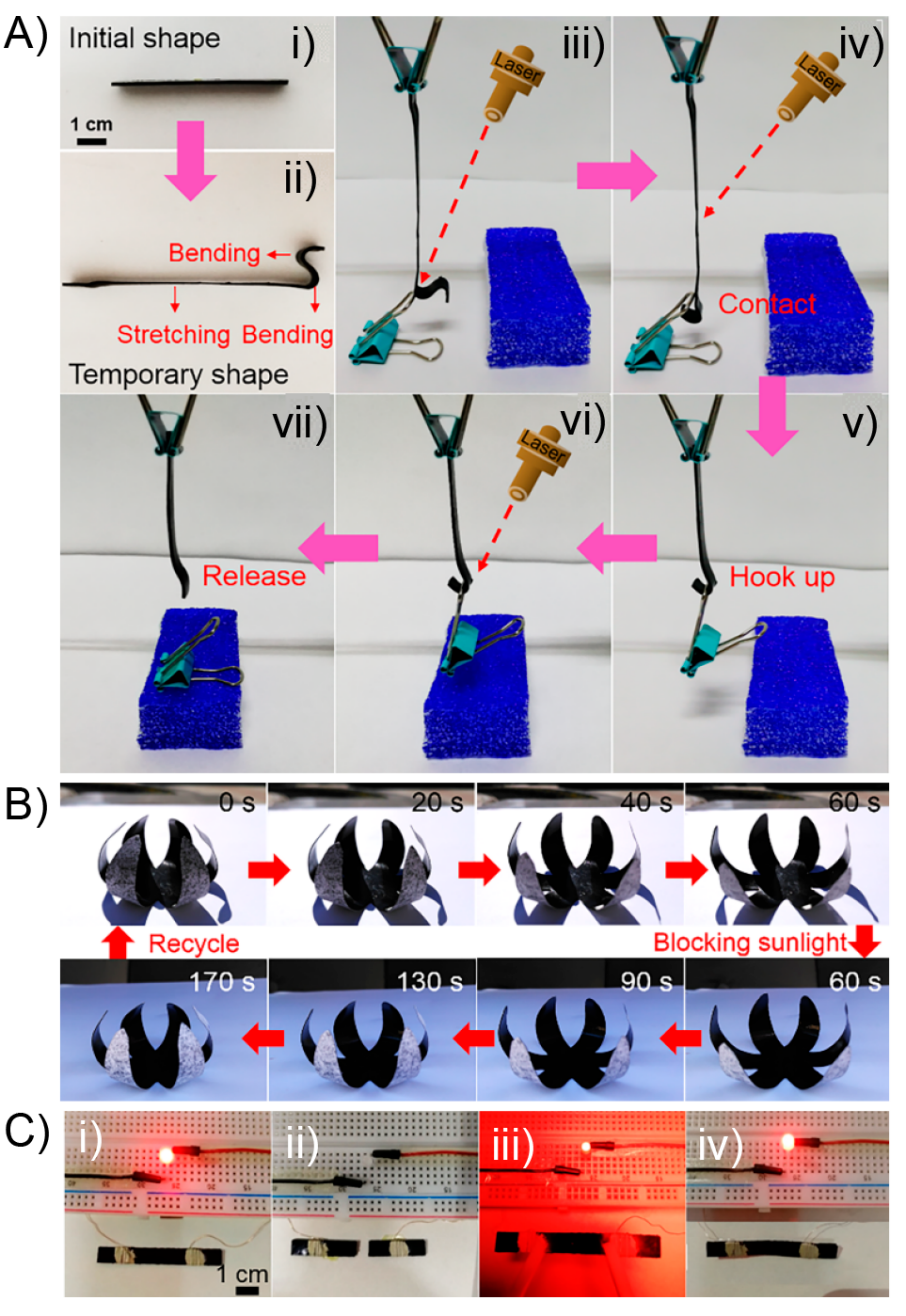
(A) Digital images of a remotely controlled light-actuated hook showing its (i) initial shape, (ii) temporary shape, and (iii–vii) the composite hook contacting the binder clip, hooking it up, and releasing it. (B) Cyclic shape changes of a bionic flower made of bilayer composites controlled by natural sunlight. (C) Digital photos of the circuit constructed by MWCNTs/CPPC sheet and a light-emitting-diode (LED) lamp at different states: (i) the LED is lighted at the initial state, (ii) the composite sheet is cut into two parts extinguishing the LED, (iii) the two parts of the sheet are connected under IR light, and (iv) after the healing process of the sheet the LED is lighted up again. Adapted with permission from ref (151). Copyright 2020 Elsevier.

A few months later, Cui and co-workers developed a light-actuated nanocomposite exhibiting a fast light-induced self-healing property.^151^ This material, comprised of multiwalled carbon nanotubes (MWCNTs) dispersed inside chlorinated poly(propylene carbonate) (CPPC), exhibited an outstanding shape-memory property activated by IR light, simulated sunlight, and also by natural sunlight, giving way to a fast and strong response that can be remotely and sequentially activated. Experimentally, MWCNTs/CPPC nanocomposites were fabricated by melt blending and molded into sheets through a hot-press process conducted at 140 °C and 10 MPa. Following the same above protocol, the authors also prepared a sample consisting of pure CPPC for comparison purposes. In the case of nanocomposites, four different amounts of MWCNTs were used (1, 2, 3, and 4 wt %). SEM analysis showed a uniform distribution of MWCNTs within the polymer matrix, even when through conductivity measurements a percolation threshold around 1.5 wt % was estimated. In addition, the mechanical properties of obtained samples showed that both the Young’s modulus and the tensile strength values increased with the MWCNTs load, revealing that these entities would help to reinforce the internal network of the material. Prior to studying the light-actuated properties, the authors analyzed the thermally induced shape-memory function of pure CPPC. They found that this polymer exhibited an excellent shape-memory property when heated at 60 °C, above its glass transition temperature (*T*_g_ = 36.2 °C), achieving full recovery from stretched or bent initial configurations. Then, aiming to obtain a more quantitative information about this property, through DMA measurements the shape fixing ratio (*R*_f_) and the shape recovery ratio (*R*_r_) were calculated. It is important to keep in mind that *R*_f_ is related to the crystalline phase of the material, whereas *R*_r_ corresponds to the amorphous portion. Values of 100% and 85.5% were calculated for *R*_f_ and *R*_r_, respectively, corroborating that CPPC is an excellent thermally driven shape-memory material. Due to the above, because MWCNTs exhibit a remarkably photothermal effect,^152,153^ the inclusion of these entities into CPPC resulted in an attractive strategy to search for synergic effects during the fabrication of light-responsive materials. Indeed, under IR irradiation (150 mW/cm^2^), the authors evidenced a much more rapid increment of the surface temperature in nanocomposites than in pure CPPC. In this sense, while pure CPPC reached a temperature of 41.7 °C after 60 s of IR illumination, the nanocomposite containing the lowest amount of MWCNTs (1 wt %) was able to reach temperatures above the *T*_g_ of the system (46.0 °C) in only 10 s. Later, to test the light-triggered shape-memory function of these materials, a flat sheet of MWCNTs/CPPC (1 wt %) was heated up to 60 °C, deformed (stretched, bended, or twisted), and finally cooled to room temperature in order to fix the new temporary shape. As an example of the above, a sheet deformed into a “U” shape was able to rapidly recover its original flat shape (*R*_r_ = 100%) after 40 s of IR irradiation (150 mW/cm^2^), whereas the same experiment performed on a pure CPPC strip required about 160 s of illumination. Aiming to demonstrate the potential application of these MWCNTs/CPPC nanocomposites in high-tech fields, the authors prepared a light-actuated hook that can be remotely controlled (Figure 8A). The hook’s temporary shape was achieved from a flat MWCNTs/CPPC (1 wt %) sheet, which was subjected to a stretching process (200% strain) followed by two bending deformations at one of its ends. Then, by sequentially irradiating different zones of the sample, its original shape could be recovered, and during this process, the hook was able to grab an object, lift it, and place it in another position of higher altitude. Surprisingly, the contraction force exerted by the sample with 200% strain was 2.6 ± 0.2 N, meaning that a MWCNTs/CPPC (1 wt %) sheet can lift 550 times its own weight. Another critical fact resolved by the authors of this work was the dependence of the shape recovery time on the thickness of the sample, going from 40 to 7 s of IR light irradiation when the thickness falls from 1.0 mm to 0.2 mm. The same phenomenon was observed using simulated sunlight of low density (87 mW/cm^2^), achieving a rapid photoactuation property in samples having low thickness, thereby opening the possibility of using the abundant solar energy to trigger shape changes. Motivated by the sunlight-induced shape-memory property of these materials and taking as inspiration the heliophile flowers, the authors prepared an artificial flower by assembling petals fabricated from MWCNTs/CPPC films. The closed state was chosen to be the temporary form of the flower, which was achieved by heating the system up to 60 °C and rapidly cooling it to room temperature. Then, under IR light irradiation, the petals gradually open into a fully bloom state within 70 s. Additionally, by decreasing from 1.0 to 0.2 mm the thickness of petals, the bloom state was reached under natural sunlight in only 39 s. However, unlike a real heliophilous flower, this system could not perform reversible opening and closing movements. To emulate this situation, the authors assembled a new flower but using petals fabricated from a bilayered material comprised of a MWCNTs/CPPC film hot-pressed onto paper (Figure 8B). The above idea emerged from the much lower thermal expansion coefficient that paper has compared to CPPC;^154^ thereby, because the paper layer is more likely to remain bent, a new force driving the shape change from the open state to the close state will remain once the light source is removed. Thus, under IR or natural sunlight illumination, the MWCNTs/CPPC layer will tend to reach its stretched configuration, creating a driving force that dominates the process, however, when the light source is turned off, the driving force caused by the paper will induce the petals bent again. In this way, by alternating light and dark conditions, the system was able to achieve these photoactuated motions in a cyclic manner. On the other hand, MWCNTs/CPPC nanocomposites also exhibited a fast and efficient light-induced self-healing capability, ascribed to the excellent photothermal effect displayed by MWCNTs, allowing the temperature of the system to increase rapidly above its *T*_g_. Regarding the above, due to the greater mobility of the polymer matrix at the rubbery state, the proposed self-healing mechanism would involve the interdiffusion and re-entanglement of the polymer chains along the damaged area. The authors evaluated the efficiency of the healing process, first, by comparing the mechanical properties between the original and the healed samples obtained after 3 min of IR irradiation, demonstrating that healed nanocomposites maintain robust mechanical properties in terms of Young’s modulus, tensile strength, and elongation at break. As a proof of this, a healed sample was able to sustain a weight of 1000 g. In a complementary way, the authors studied the efficiency of the healing process through the evolution of the conductive properties of the material under light irradiation (Figure 8C). After being cut into two halves, the resistance of the sample showed an abrupt increase ascribed to the disruption of the inner MWCNTs network. However, after inducing the contact between both pieces, the resistance value started to decrease rapidly after 5 s of IR light irradiation. This was ascribed to the reconstruction of the MWCNTs conductive network promoted by the interdiffusion and re-entanglement of CPPC polymer chains. Interestingly, the resistance value gradually decreases up to reach a value close to the measured resistance before the damage. Moreover, the robustness of the healing process was successfully demonstrated by consecutive cutting–healing cycles. Conversely, when visible light was used instead of IR irradiation, the healing process was not achieved completely, possibly related to the greater penetration of IR light. This is a relevant factor to be solved if we want to improve the performance of the new generation of self-healable actuators, searching to maximize their functions by using conditions provided by nature.

**Figure 9:**
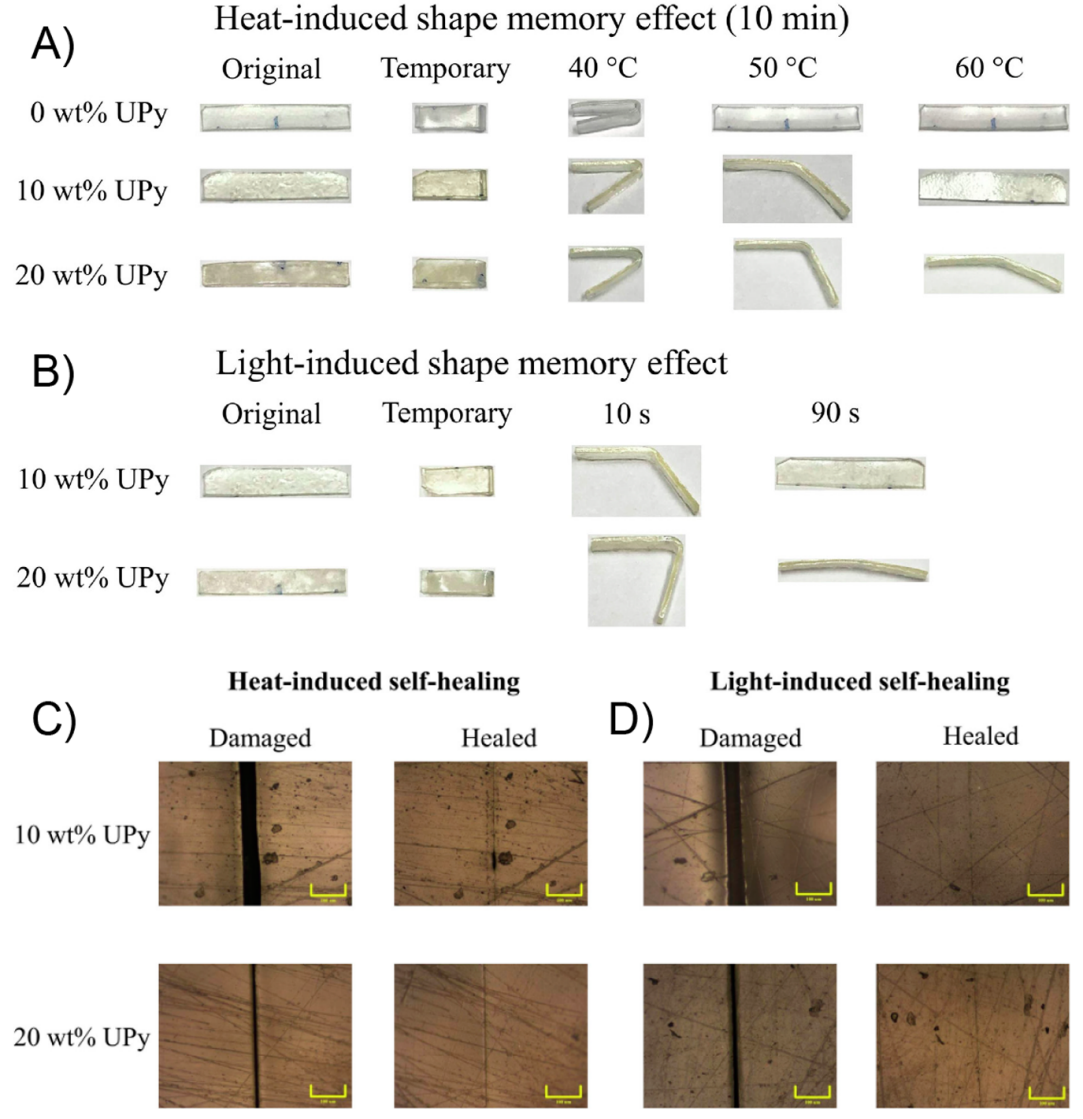
Heat-induced (A) and light-induced (B) shape-memory behaviors of PEG-PCL-UPy polymers with different UPy contents. Optical micrographs showing scratches of PEG-PCL-UPy polymers before and after healing by heat (C) and light (D) (scale bar = 100 μm). Adapted with permission from ref (155). Copyright 2020 Elsevier.

In the same year, Yan and co-workers prepared a polymer-based network standing out by its high toughness and displaying both light-triggered shape-memory and self-healing properties.^155^ Motivated by the outstanding mechanical properties and the fast and efficient self-healing ability, the authors successfully tested this material as a strain sensor, demonstrating its potential application in wearable electronic devices. By polymerizing methacrylic derivatives of PEG, poly(ε-caprolactone) (PCL), and UPy, a supramolecular network constructed by covalent and transient cross-links was achieved. Based on thermal and mechanical characterizations, the authors concluded that samples containing 10–20% UPy content were able to display efficient shape-memory and healing properties. The material revealed an excellent mechanical performance characterized by tensile stress and toughness values of 7.2 MPa and 25.2 MJ/m^3^, respectively, attributed to its semicrystalline nature provided by the presence of both PEG and PCL segments and the existence of multiple hydrogen bonding interactions provided by UPy units. On the other hand, DSC measurements showed *T*_m_ values in the range of 40–47 °C, corroborating the presence of crystalline domains within the matrix. The shape-memory property shown by these materials was attributed by the authors to reversible melting-crystallization processes, which can be triggered either by external heating (Figure 9A) or by light irradiation (Figure 9B). The latter based on the well-known ability of UPy units to transform ultraviolet light into heat.^156,157^ Regarding the above, the photothermal property of samples was studied under UV irradiation (365 nm) by varying the intensity of the light source (200–500 mW/cm^2^), concluding that after 2 min under UV-light irradiation at 500 mW/cm^–2^ the sample reaches a temperature of ≈63 °C, which is higher than the melting temperature of the network and, thereby, enough to activate the dynamic process of UPy dimers. Thus, the shape-memory property was first evaluated by folding samples at 70 °C (above *T*_m_), followed by a cooling process. All networks were able to maintain their temporary shapes for long periods, characterized by excellent shape fixing ratio values (≈100%). Then, the shape recovery process of bent samples was carried out by using a laser beam as irradiation source (365 nm, 500 mW/cm^2^). After 10 s of irradiation, samples containing 10 and 20% of UPy content reached shape recovery values of 71% and 47%, respectively, while after 90 s these values increased up to 99% and 92%. The authors attributed the good performance showed by these systems to the photoinduced heat generated in the irradiated area that allows, simultaneously, the melt of crystalline domains and the cleavage of hydrogen bonds existing between UPy units. Due to the above, the strained sample was able to release the stored strain energy by inducing the apparition of restoring forces that triggers the shape recovering process. Regarding the self-healing ability displayed by these systems, after scratching the sample’s surface with a razor blade, the authors evaluated this property by using external heat (10 min at 70 °C) (Figure 9C) and UV light irradiation (365 nm, 500 mW/cm^–2^, 1 min) (Figure 9D). Interestingly, damaged samples were able to fully close the cracks under both conditions, however, showing evident advantages in terms of time when light was used. The healing efficiency was also evaluated by tensile test, where samples healed under light irradiation showed better restoring of their mechanical properties than those healed under heat. Particularly, the light-healed network having 20% of UPy content reached 86% of the original toughness. In this sense, when the healing process is carried out by temperature the entire sample is affected, inducing the healing of the damaged area but also prompting the disruption between UPy units in undamaged areas. As authors argued, the healing process is assisted by shape-memory motions that help bring together interfaces along the damage area, facilitating the encounter and reformation of UPy hydrogen bonds.

**Figure 10:**
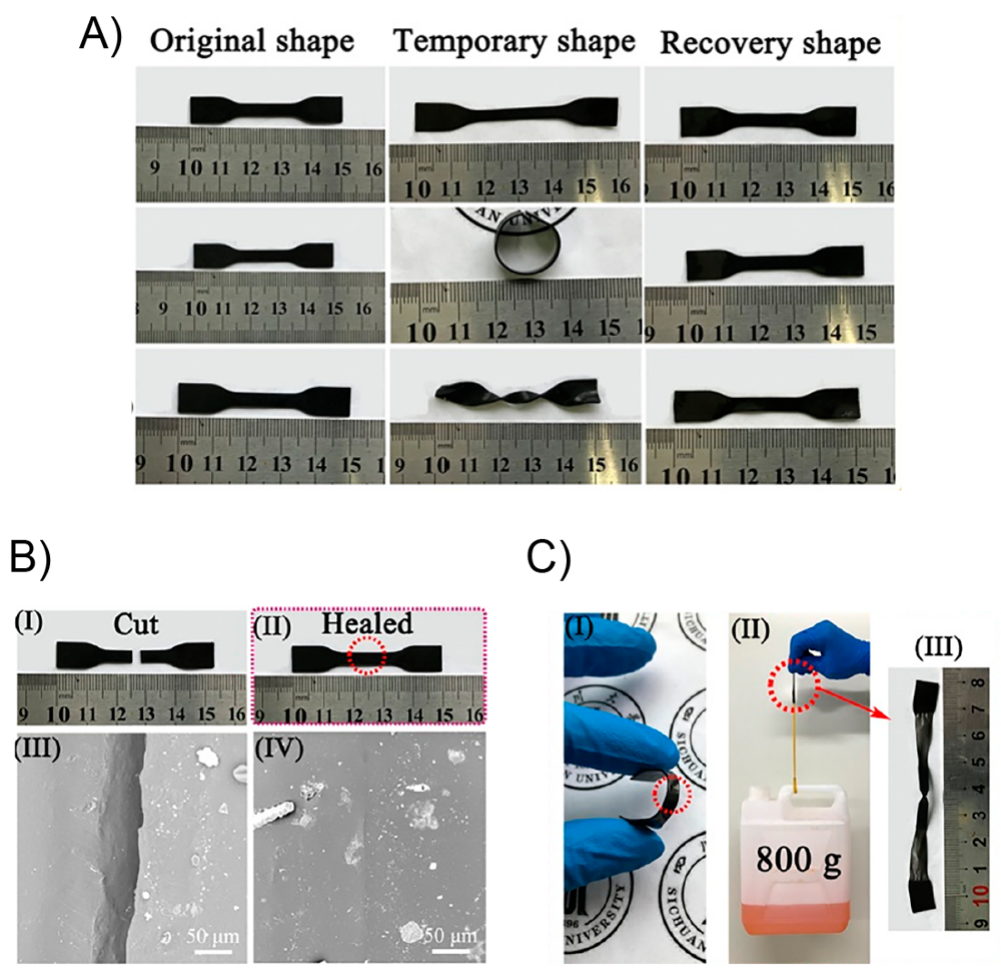
(A) Digital photographs of dPTD–mfGO nanocomposites strips showing their initial, temporary, and recovery shapes achieved during the shape-memory process under VIS–NIR irradiation (400–1100 nm). (B) Digital images (I–II) and SEM micrographs (III–IV) of a healed dPTD-mfGO sample. (C) Digital photographs showing the self-healing behavior of the healed dPTD–mfGO2 with different shapes: (I) bending, (II) bearing a weight of 800 g, and (III) after stretching. Adapted with permission from ref (158). Copyright 2020 Elsevier.

The design of polymeric systems featuring efficient flame retardancy function has become an innovative topic as they are able to reduce fire risks and economic losses in practical applications. Motivated by the above and by the absence of reports on self-healing actuators showing flame retardancy properties, almost parallel to Yan et al.,^155^ Du et al. devised the preparation of a new polymer-based nanocomposite showing shape-memory and self-healing, both activated by VIS–NIR light (400–1100 nm), achieving an outstanding flame retardant capacity.^158^ This material was achieved by dispersing multifunctionalized GO entities within a polymer matrix consisting of a polyurethane (PU) containing reversible diselenide bonds. The light-triggered shape-memory property was ascribed to the photothermal effect displayed by these graphene oxide derivatives under VIS–NIR irradiation, while the self-healing ability, also activated by light, was attributed to a combination of the above photothermal effect and the VIS–NIR reversible formation of Se–Se bonds. First, the authors carried out the synthesis of a multifunctionalized GO (mfGO) through the covalent incorporation of nitrogen, phosphorus, and silicon-based molecular units. The N, P, and Si containing structures were poly(ethylenimine) (PEI), 9,10-dihydro-9-oxa-10-phosphaphenanthrene (DOPO), and isocyanatopropyltriethoxysilane (IPTS), respectively, which were selected based on past reports suggesting the adequate fire safety properties exhibited by materials containing these types of substrates.^159−161^ The preparation of mfGO was corroborated in terms of FT-IR, Raman, TGA, XRD, and scanning electron microscopy–energy dispersive X-ray spectroscopy (SEM-EDS), showing a correct chemical structure, along with important features related to chemical composition and morphological properties. For instance, XRD analyses showed a peak at 2θ = 9.77° (002 reflection) and a *d*-spacing value of 9.05 Å for GO. In contrast, the 2θ peak was shifted down to 7.42°, and the interlayer spacing was increased to 12.01 Å in the case of mfGO. This increase of the *d*-spacing was attributed to the intercalation of PEI, DOPO, and IPTS units between the GO sheets. Moreover, in contrast to pristine GO, mfGO displayed a higher thermal stability, supporting its potential application as flame retardant. On the other hand, following well-known solution polymerization protocols, the authors conducted the preparation of a PU from poly(hexylene-adipate) diol (PHA), polytetramethylene ether glycol (PTMG), and 2,4-toluene diisocyanate (TDI). To the above solution, they incorporated mfGO achieving a 2 wt % dispersion followed by the addition of di(1-hydroxyethylene) diselenide as chain extender. Then, the reaction mixture was poured on a Teflon mold and cured at 60 °C for 48 h, obtaining the final nanocomposite coded as dPTD–mfGO (dPTB refers to the polyurethane copolymer containing 1,4-butanediol (BDO)). Similarly, the above process was repeated but using 1,4-butanediol instead of di(1-hydroxyethylene) diselenide, generating a nanocomposite coded as dPTB–mfGO, which was used for comparison purposes. Surprisingly, the authors evidenced a notably better dispersion for mfGO than pristine GO in the polymer matrix, showing no evidence of agglomeration phenomena. This observation, ascribed to an enhancement of compatibility, was attributed to hydrogen bond interactions occurring between PU chains and PEI grafts attached to the surface of mfGO. Following the same argument, the so-obtained dPTD–mfGO nanocomposite exhibited better mechanical properties than pure dPTD PU, demonstrated by an increase of 23% and 16% for the tensile strength and elongation at break values (dPTD refers to the polyurethane copolymer containing di(1-hydroxyethylene)diselenide). The authors explained this result based on a more effective load transfer from the polymer to mfGO sheets under external mechanical stress. In terms of *d*-spacing, the values of mfGO containing PTB and PTD increased to 12.35 and 12.26 Å, respectively. Such an increase compared to the pristine mfGO (12.01 Å) was ascribed to the strong hydrogen-bonding interactions between mfGO sheets and PU chains. In addition, the DMA analysis showed that both dPTD–mfGO and dPTB–mfGO nanocomposites exhibited higher Young’s modulus values than their counterparts matrices (dPTD and dPTB), suggesting a higher rigidity and toughness of the polymer matrix in the presence of mfGO. The presence of crystalline regions in nanocomposites was confirmed by means of DSC and XRD measurements. All PU and nanocomposites films showed relatively similar melting behavior, characterized by two endothermic processes around ≈24 °C and ≈38 °C assigned to the melting of PHA and PTMG, respectively. However, the enthalpic contribution of the former was notably higher than the one associated with PTMG segments. Thus, the crystalline structure in these samples was mainly related to PHA. Furthermore, measurements revealed that nanocomposites containing mfGO showed an increment in their crystallinity, suggesting that these entities can act as nucleation sites, reinforcing the presence of crystalline domains. The latter found direct relation with the adequate shape fixity exhibited by these samples during shape-memory process. As a first approach to study the shape-memory process, a simple bending test was conducted to evaluate parameters such as the *R*_f_, *R*_r_, and recovery times under VIS–NIR irradiation and also external heating. Regarding *R*_f_, while neat polyurethanes dPTD and dPTB exhibited values of 88.3% and 91.5%, respectively, their counterparts containing mf-GO showed increased values around 95.2% and 96.7%. The same trend was observed for *R*_r_ where, again, dPTD–mfGO and dPTB–mfGO nanocomposites showed values of 93.1% and 95.5%, respectively, being higher than those measured for pure polyurethanes. The higher *R*_f_ values were related to the increased crystallinity degree and the presence of hydrogen bonding within the polyurethane structure, both attributed to the presence of mfGO. On the other hand, the increment on the *R*_r_ values after the incorporation of mfGO would be ascribed to an enhancement of the physically cross-linking process between the attached polymer chains over mfGO and the PU matrix. The authors also prepared two additional samples consisting of dPTD and dPTB polyurethanes containing nonfunctionalized GO. Both samples displayed *R*_f_ and *R*_r_ values even lower than neat dPTD and dPTB, accusing the importance of achieving a good filler dispersion. Another important parameter measured for all samples was the recovery time, defined as the time required to reach the maximum *R*_r_ value. This parameter was evaluated under two modalities: VIS–NIR irradiation and external heat. Recovery times were notably faster under light irradiation than under heating; however, during light experiments, only those samples containing mfGO were able to carry out the recovery process, demonstrating the importance of mfGO. The authors also performed an additional experiment using pristine GO as filler in dPTD and dPTB, obtaining longer recovery times in comparison to dPTD–mfGO and dPTB–mfGO, which, according to authors, this result could be related to the low dispersibility showed by unmodified GO. Notwithstanding the above, the need of mfGO or GO to achieve the shape-memory property under light irradiation was attributed to the well-known photothermal effect displayed by these entities, especially under NIR conditions. The authors also evaluated the repeatability of the shape-memory process, performing consecutive cycles of deformation. Results showed that, while pristine dPTD and dPTB exhibited a noticeable diminishing in their *R*_f_ and *R*_r_ values, after three cycles, nanocomposites containing mfGO kept both values above 90%. Later, focusing on dPTD–mfGO, the authors demonstrated the ability of this system to recover its initial shape from different type of deformations (Figure 10A). In this sense, dPTD–mfGO samples were initially heated at 45 °C (above its melting temperature) and reshaped into different configurations such as circles and spirals. Subsequently, the system was allowed to cool down to fix the new temporary shape. All dPTD–mfGO samples showing different temporary shapes were able to recover their initial shapes in notably short times under VIS-NIR light. For example, the dPTD–mfGO sample that was deformed into a spiral shape recovery its original configuration within 10 s. The actuation mechanism proposed by authors was based on melting-crystallization phenomena triggered by the photothermal effect. Regarding the above, temporary shapes were successfully achieved after deforming samples heated above their melting temperatures. Then, thanks to the nucleation effect showed by the mfGO entities during PU crystallization, the system maintained the initially induced shape. Then, during light irradiation, the mfGO fillers allowed the transformation of VIS–NIR light into thermal energy, which was transferred through the polymeric matrix inducing the melting of crystalline domains, releasing the stored strain energy, and, thus, triggering the shape recovery process. On the other hand, the authors also evaluated the light-activated self-healing property of these materials by cutting samples into two pieces, brought into contact and irradiating them for 3 min with a VIS–NIR source (400–1100 nm, 25 mW/cm^2^), after which a one-single piece of material showing no evidence for scars under SEM visualization was obtained (Figure 10B). To achieve this quantitatively, tensile experiments were performed on original and healed samples, reporting the healing efficiency as the ratio of the maximum tensile strength or elongation at break values of the healed and original samples. Evidently, pure dPTB PU showed a poor healing efficiency, characterized by healing efficiencies of 14% and 1% for maximum tensile strength and elongation at break, respectively. Contrary to the above, dPTD revealed a better healing property with healing efficiency values of 31% and 41%. Authors ascribed this to the light-triggered exchange reaction of diselenide bonds present in the polymer backbone.^162,163^ Surprisingly, the healing efficiencies of dPT–mfGO (39% and 70%) and dPTD–mfGO (80% and 96%) nanocomposites were notably increased after the incorporation of mfGO, demonstrating the importance of the photothermal effect on the healing process. In this sense, by using an infrared digital camera, the authors were able to visualize the rising of the temperature for each sample under VIS–NIR irradiation. Samples containing mfGO achieved temperatures above 45 °C in only 8 s, reaching temperatures as high as ≈55 °C in around 16 s. Conversely, none of the pure PU samples reached temperatures above 30 °C in the same time scale. Therefore, it was clear that nanocomposite samples in short times of irradiation were able to surpass the melting temperature of PU segments, favoring the mobility, diffusion, and re-entanglement of polymer chains across the damage area, promoting the healing process. Notwithstanding the above, it must be pointed out that the healing efficiency of dPTD–mfGO was considerably higher than dPTB–mfGO even when both have a similar photothermal response. Regarding the above, the authors attributed this result to a synergistic effect achieved between the photothermal effect provided by mfGO and diselenide exchange reactions present in dPTD–mfGO. Both light-activated processes would complement each other, allowing a better healing process under VIS–NIR irradiation where, simultaneously, the melt of crystalline domains and the activation of diselenide linkages would promote the interfacial diffusion and re-entanglement of polymer chains across the damaged area. Surprisingly, the healed dPTD–mfGO sample was able to maintain a weight of 800 g and also resist strong bending and stretching deformations without showing signals of fracture at the joint position (Figure 10C). Finally, these photoactuators exhibiting light-triggered self-healing property were successfully tested as flame retardant materials, demonstrating that the multifunctionalization of GO and its incorporation into PU matrices allow the enhancing of the thermal stability and the flame retardancy property.

**Figure 11:**
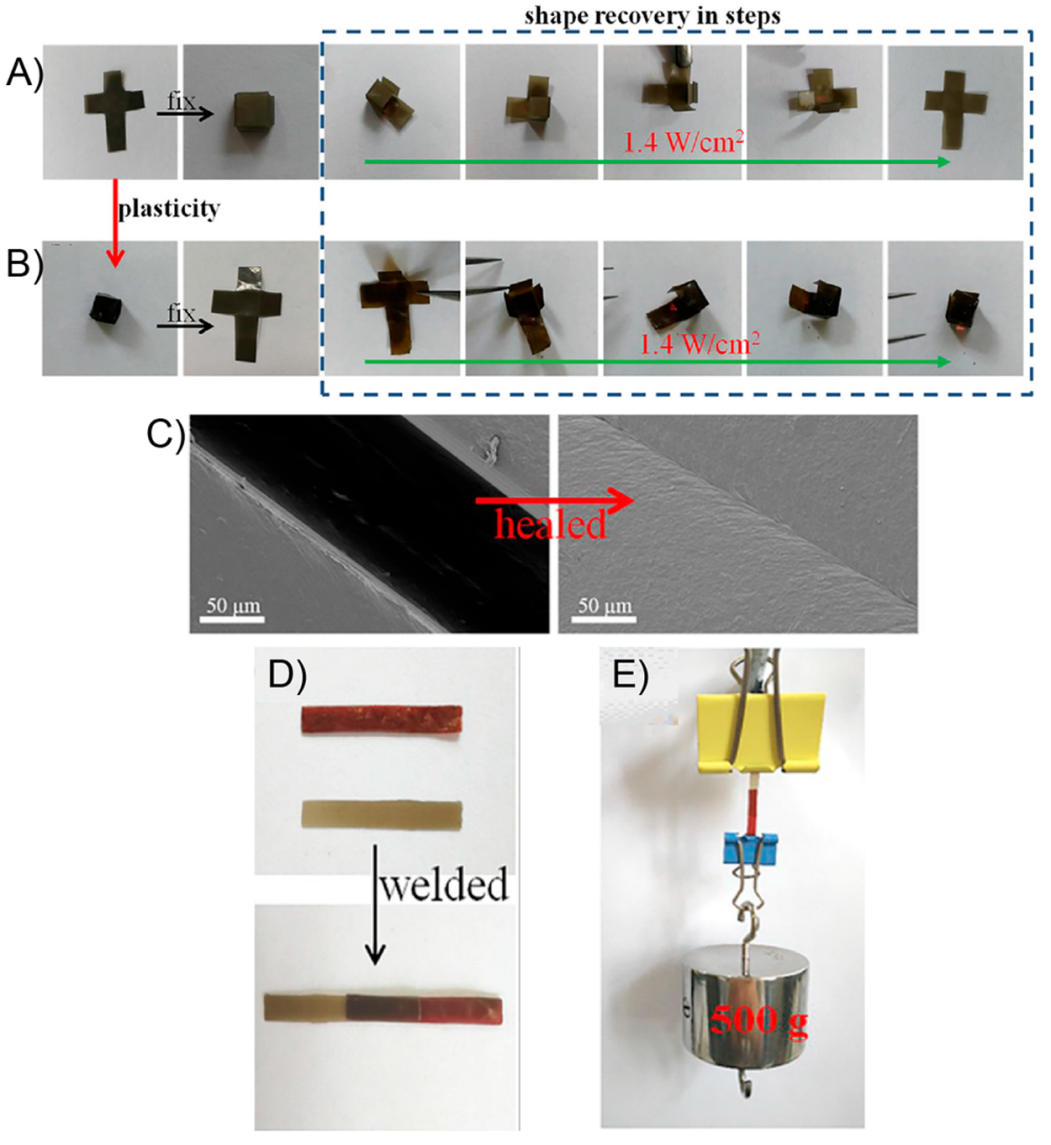
(A) Light-induced shape-memory behavior of original PU/GO network. (B) Light-induced shape-memory behavior of PU/GO network after light-induced plasticity. (C) SEM images of light-driven self-healing process. (D) Optical image of the photo welding process between two pieces of PU/GO. (E) Optical image showing the welding strength. Adapted with permission from ref (164). Copyright 2020 Elsevier.

By the end of 2019, Bai and co-workers also introduced a light-responsive thermoset consisting of a cross-linked PU-based matrix containing graphene oxide (GO) as filler.^164^ The achieved thermoset polymer, thanks to the outstanding photothermal effect of GO, displayed both NIR light-induced shape-memory and self-healing capabilities. The material, under remote NIR irradiation, was able to be repeatedly reshaped into new configurations showing no alteration of its shape-memory function, also maintaining good mechanical properties. Due to the above features, authors proposed this system to be potentially used as light-controlled actuator, self-healing coatings, and as an optical welding material. Authors conducted the manufacturing of the PU/GO thermosetting nanocomposite by first grafting PCL onto GO for then inducing the network formation using 4,4-methylenediphenyl diisocyanate (MDI) as the cross-linking agent. This in situ polymerization protocol allowed obtaining well-dispersed GO nanosheets within the polymer matrix as was demonstrated by transmission electron microscopy (TEM) analysis. The above, plus the well-known high photothermal conversion efficiency of GO, allowed this material to be endowed with remarkable shape-memory and self-healing properties induced by NIR light. Authors demonstrated that a 0.1 wt % of GO was enough to obtain a light-responsive material showing good mechanical properties, because at higher compositions, samples became brittle. Because the actuation property of the thermoset is related to the photothermal effect exhibited by GO, the authors first studied its shape-memory capability activated purely by temperature. The *T*_m_ values measured for samples was around 68 °C, serving as the switching temperature for the shape-memory process. By thermomechanical analysis (TMA), authors demonstrated that samples exhibited excellent shape-memory properties showing a shape recovery ratio of 95.4% after being incubated at 90 °C. Interestingly, authors also studied the solid-state plasticity displayed by these materials using dynamic mechanical analysis (DMA), where samples were subjected to a 10% tensile strain, which was completely relaxed after being heated at 130 °C. Taking the above results as reference, authors were able to study both light-induced shape-memory and light-induced plasticity phenomena. However, because both processes are activated at different temperatures, two NIR irradiation power densities of 1.4 and 2.5 W/cm^2^ were used to reach 90 and 130 °C, respectively. A bending recovery test was used to evaluate the two processes. First, the initial sample was folded in half under 1.4 W/cm^2^ light irradiation, achieving the temporary shape after being equilibrated at room temperature during 3 min. This folded sample was again irradiated under 1.4 W/cm^2^ NIR light, recovering its initial shape within seconds. On the other hand, regarding the plasticity process, the sample was again bent in half but now irradiated with a power density of 2.5 W/cm^2^ during 30 min, in order to relax the bending force. It must be mentioned that authors did not evidence any shape recovery process for this sample when it was irradiated with a 1.4 W/cm^2^ power source, demonstrating a successful light-induced reconfiguration due to plasticization. This stable reshaped sample maintained a remarkable shape-memory property, recovering its initial bent shape after being stretched under 1.4 W/cm^2^ irradiation. Authors confirmed the robustness of the method by conducting several consecutive cycles of the above process, also reconfiguring the sample’s shape repeatedly between straight and bent formats. Thereby, under NIR light irradiation, the permanent shape of samples was easily reconfigured through light-induced plasticity at the same time that no relevant weakening of the shape-memory property was observed, displaying shape recovery ratio values of around 95.4%, being notably similar to those obtained by thermal treatment. However, one of the strong points of this work was the application of both light-induced properties to induce the formation of more complex structures. In this regard, Figure 11A shows a planar cross-shape sample that was deformed into a cube after being irradiated and equilibrated at room temperature to then, under NIR irradiation (1.4 W/cm^2^), returned step by step into its original planar shape. Notwithstanding the above, when the cube structure was irradiated with the 2.5 W/cm^2^ power source, the opposite process was achieved, where the planar cross-shape sample recovered its cubical form under 1.4 W/cm^2^ power source NIR irradiation (Figure 11B). Furthermore, because light-triggered plasticity is directly related to polymer chain motions and carbamate exchange reactions,^165,166^ authors found that these materials also displayed light-induced self-healing capability. This property was successfully demonstrated by means of optical microscopy (OM) and SEM images, where razor blade cracks were completely healed after 30 min of 2.5 W/cm^2^ NIR light irradiation (Figure 11C). Aiming to quantitatively evaluate the healing process, both original and healed samples were subjected to tensile strength measurements. These experiments revealed that original and healed materials reported values of about 16.6 and 14.2 MPa, respectively, indicating that around 85% of the lost strength was recovered thanks to the light-driven self-healing process. Authors state that a possible explanation for the observed healing phenomenon would be ascribed to the photothermal mechanism activated by NIR light in which the diffusion of polymer chains across the wounded interface is prompted. Thereby, after diffusion and entanglement processes, along with the occurrence of carbamate exchange reactions, polymer chains are able to rearrange and heal the crack. Finally, motivated by the outstanding light-triggered properties displayed by this class of nanocomposite, authors evaluated its performance as light actuators, self-healing coatings, and optical welding materials, demonstrating its broad versatility. Regarding the light actuator applications, nanocomposités strips were able to lift up around 2000 times its own weight under 1.4 W/cm^2^ NIR light irradiation. On the other hand, corrosion tests were carried out on metal pieces coated with this material. The deposited coating was intentionally damaged, and after inducing its light-triggered healing, the sample did not show clues of corrosion. This was not the case for samples where the healing process was not activated, in which clear signs of corrosion were visible. Lastly, two portions of nanocomposite were welded under 2.5 W/cm^2^ NIR light irradiation for 30 min (Figure 11D), after which a burden 25000 times heavier than sample’s weight was successfully bearing (Figure 11E). In summary, the strategy developed by the authors of this work allowed the easy preparation of a highly versatile light-activated system that shows remote actuation control, the possibility of being used as a self-healing coating and the construction of strong optically welded materials.

**Figure 12:**
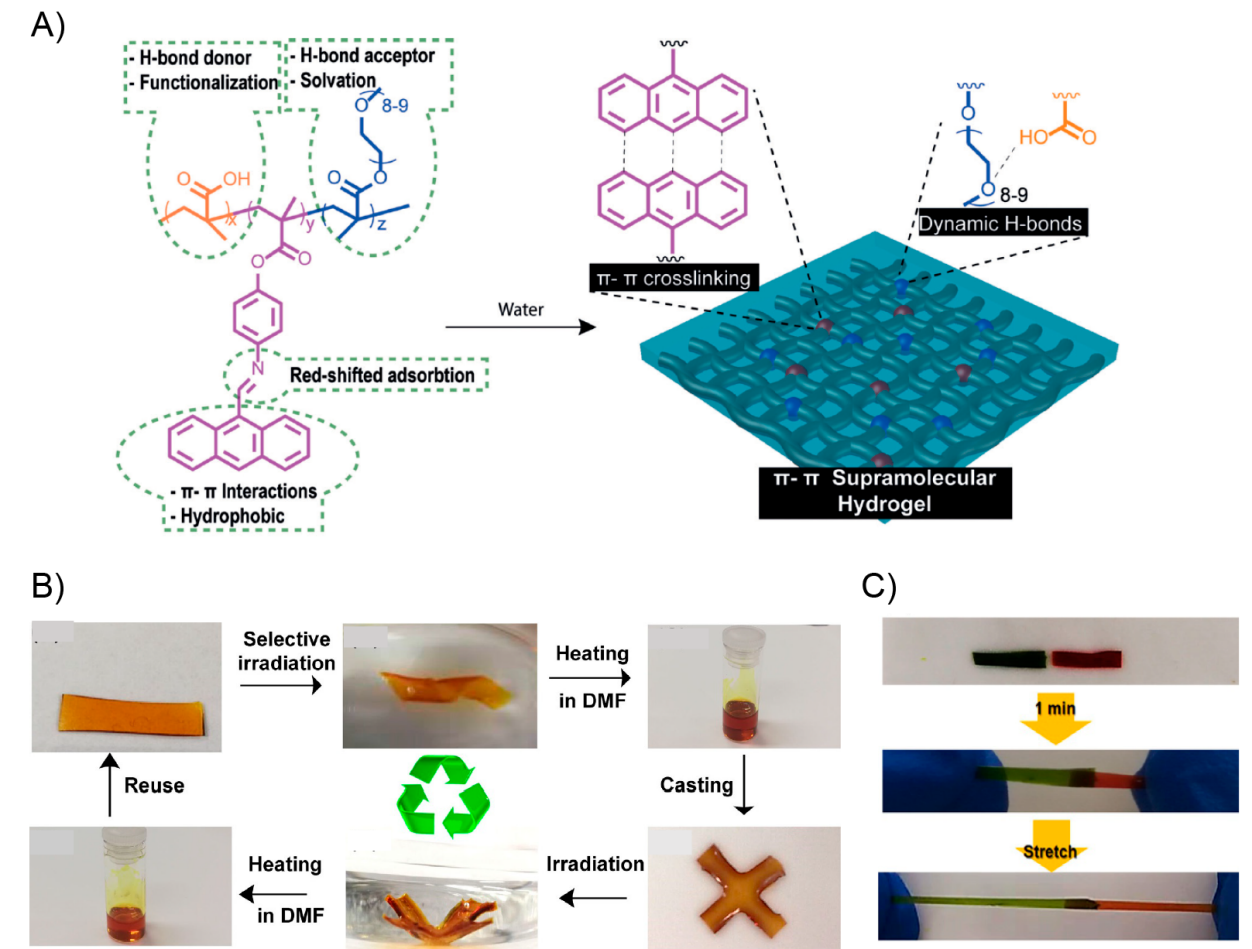
(A) Chemical structure of supramolecular hydrogel and different interaction of each functional groups. (B) Photographs showing the recycle and reprogrammable process of hydrogel photographs showing qualitative evidence of self-healing; after healing for 1 min, the gels could be stretched to 100%. (C) Photographs showing qualitative evidence of self-healing; after healing for 1 min, the gels could be stretched to 100%. Adapted with permission from ref (167). Copyright 2020 John Wiley and Sons.

Light-responsive shape-memory hydrogels are typically made with covalent cross-linkers and cannot be recycled. With this idea in mind, in 2020, Jiang et al. developed a double-cross-linked supramolecular light-responsive hydrogel actuator which is self-healable and recyclable.^167^ The hydrogel was synthesized by covalent integration of the anthracene derivative into an engineered copolymer of methacrylic acid (MAA) and oligo ethylene glycol methyl ether methacrylate (OEGMA) (i.e., poly(MAA-*co*-OEGMA)) (Figure 12A). In addition, with hydrogen bonding between MAA and OEGMA,^168^ anthracene moiety also plays a significant role by providing strong π–π interactions, thus forming a supramolecular network in water which significantly enhances the mechanical strength of hydrogel/film. Besides the π–π interactions, anthracene moiety undergoes dimerization upon visible light irradiation (420–530 nm, 10 mW/cm^2^),^169^ resulting in higher mechanical strength demonstrated by the increase in Young’s modulus (11-fold) and tensile strength (20-fold) values compared to the original gel before radiation. The higher stiffness gained by the material after irradiation was also related to the decrease in its swelling property. Regarding the photoactuation displayed by this material, under visible light exposure, the water-swollen hydrogel was able to bend 12° toward the light source side after 1 h irradiation, replicating similar motions previously reported for other hydrogel-like systems.^170,171^ Interestingly, in the dry state, the photoinduced bending movement was also observed at the irradiated side but showing higher bending angles in remarkably short times (28° within 20 s). The mechanism through which the photoactuation is achieved would be related to a light-induced gradient cross-linking density across the material. In this sense, the exposed surface of the hydrogel preferentially absorbs the incoming light increasing locally the chemical cross-linking density owing to a much lower swelling ratio than the areas away from the light sources. In this way, the strain variations generated within the inner structure of the material could be translated as macroscopic bending movements. The bent film returned to the initial state within 60 min when heated to 90 °C because of the thermally reversible anthracene dimerization. Thus, the presence of dynamic bond makes the designed structures to be recycled and reprogrammed into different 3D objects (Figure 12B). The self-healing function exhibited by the system was mainly assigned to noncovalent interactions present under constant equilibria within the material. The authors argued that at 35 °C, the hydrogel is in an elastomeric state facilitating the dynamic dissociation and reformation of hydrogen bonds and π–π interactions. Thereby, as is shown in Figure 12C, when two hydrogel pieces were attached at 35 °C, in only 1 min, a single-one system was obtained. This system stood out by its outstanding integrity and stability, as demonstrated after being stretched up to elongation of 100%. The healing function was also studied quantitatively using tensile testing experiments, measuring the recovery of the fracture stress value. Regarding the above, after 3 min of healing, a 68% recovery of the original fracture stress was calculated for the healed sample. Another relevant merit of this work that should be mentioned was the ingenious way in which the authors corroborated the participation of both hydrogen bonds and π–π interactions in the healing mechanism.

**Figure 13:**
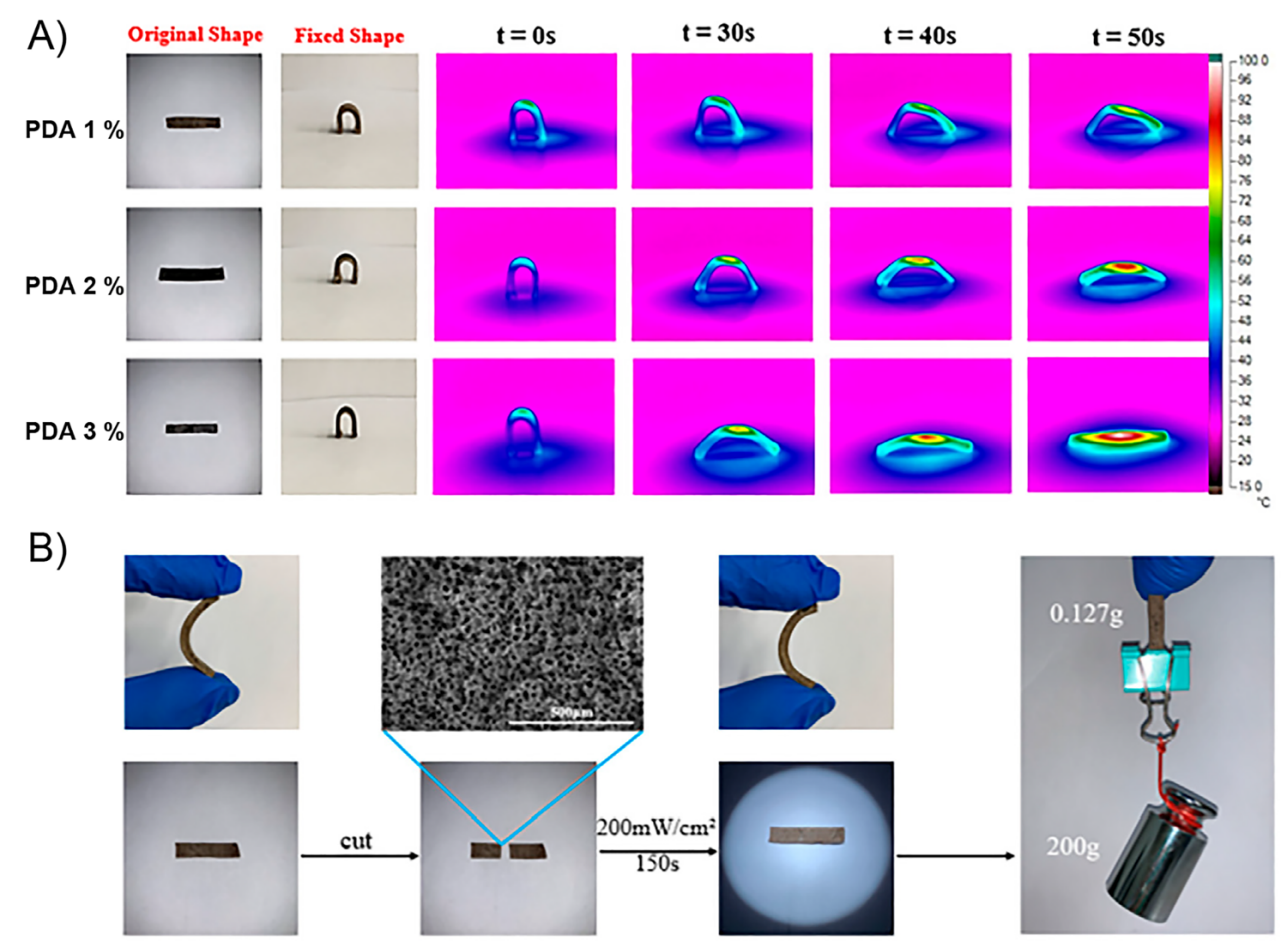
(A) IR thermal images of light-response shape recovery processes at a light intensity of 200 mW/cm^2^. (B) Self-healing performance of PCL/TPU/PDA (3 wt %) composite. Adapted with permission from ref (172). Copyright 2020 Elsevier.

In May 2020, by dispersing PDA particles within microporous polymer blends comprised of PCL and thermoplastic polyurethane (TPU), Chen and collaborators were able to fabricate polymer-based nanocomposites displaying excellent light-induced shape-memory and also self-healing ability.^172^ Both light-triggered properties were ascribed to the photothermal effect induced by the presence of PDA, along with the semicrystalline nature of the polymer matrix. The obtained materials revealed an outstanding shape recovery ratio and a fast self-healing ability, where surface cracks were healed in 150 s of light irradiation. Due to the inherent biocompatibility of the employed components, the authors proposed this type of material to be useful in the biomedical field, such as artificial muscles and soft robotics. Following an easy and straightforward solution blending protocol, authors achieved the preparation of PCL/TPU mixtures at different ratios, with TPU entities acting as net points within the polymer network, while PCL is responsible for the formation of thermally reversible semicrystalline segments. The authors evaluated the shape-memory property based on the *R*_f_ and *R*_r_ parameters. Thereby, achieving a material with a well-balance crystalline–amorphous structure was mandatory. In this sense, through XRD and DSC analysis, the authors determined that blends having 50% of PCL afford the highest crystallinity degree, presenting a melting temperature around 55 °C. Using DMA tests, first, the authors assessed the shape-memory property of these materials activated by temperature recording consecutive thermomechanical cycles. From the above results, and based on the obtained *R*_f_ and *R*_r_ values, authors demonstrated that PCL/TPU mixtures having 50% of PCL content were the most suitable to further evaluate their light-triggered shape-memory function. Therefore, to PCL/TPU 50% mixtures, different amounts of PDA particles (1, 2, and 3 wt %) were added. These particles displayed spherical shapes and diameter sizes between 400 and 500 nm. SEM images revealed that PDA particles were well-dispersed and maintain a uniform morphology once they were included in the polymer matrix. Moreover, by UV–vis spectroscopy, it was demonstrated that after the inclusion of PDA particles, materials showed higher absorbances than neat PCL/TPU blends in the range between 400–1000 nm, corroborating the efficient light absorption by the PDA dispersed phase. By aiming to investigate the photothermal effect, authors exposed PCL/TPU and PCL/TPU/DPA samples to a visible light irradiation source (Xe lamp) at a fixed intensity of 200 mW/cm^2^. The experiment revealed that, while samples without PDA particles experienced a small change in their temperature, those samples containing PDA reached temperatures above 60 °C within the first 20 s of irradiation, showing a higher heating rate ascribed to the photothermal effect. In addition, both the temperature and heating rate were higher in those materials containing greater amount of PDA. Considering the above, authors went forward by evaluating the light-induced shape-memory property of these samples using the same light source. To achieve this, PCL/TPU/PDA strips containing different amounts of PDA (1, 2, and 3 wt %) were folded and fixed into a “U” shape before starting the irradiation step. Then, the whole process was followed using an IR thermal imaging camera recording the temperature changes experienced by the irradiated samples (Figure 13A). The authors observed that samples bearing higher amounts of PDA reached higher temperatures and recovered their initial shapes more rapidly, which would be directly related to a more efficient process in which light is converted into heat. It is worth noting that samples without PDA did not show morphology changes under illumination. To carry out a more quantitative analysis of the shape-memory property, the authors plotted evolution through time of *R*_r_ values, showing that while PCL/TPU did not display any shape-memory function, all samples containing PDA particles were able to recover completely their initial shapes (*R*_r_ = 100%). Interestingly, the sample with higher PDA content (3 wt %), started to recover at 10 s of irradiation and achieved a *R*_r_ value of 100% after only 50 s. This shape-memory property was also tested on samples initially molded into more complex configurations where, for example, a sample strip fixed into a spiral shape recovered completely its initial flat configuration after being irradiated during 150 s (200 mW/cm^2^). Moreover, authors demonstrated the direct relation between the speed of light-triggered motions with the light source intensity because the same spiral-to-planar transition took place in 30 s, when the light intensity was raised to 500 mW/cm^2^. Surprisingly, the authors found that these samples were also able to self-heal under visible light irradiation as was demonstrated by cutting a PCL/TPU/PDA (3 wt %) strip into two separate pieces, which, after being put back together and irradiated at a light intensity of 200 mW/cm^2^ during 150 s, the damaged sample was able to recover its initial flexibility and even lift an object 1575 times heavier than its own weight without showing glimpses of deformation (Figure 13B). SEM images revealed that the cracks caused by the damage were still present after 30 s of irradiation, however, at 150 s they were almost imperceptible. The self-healing efficiency was evaluated from a more quantitative perspective using strain–stress measurements. Thereby, because the tensile strength of the original sample was 1.63 MPa, the healing efficiency was around 78.5%, considering that the healed sample displayed a tensile strength about 1.28 MPa. Authors attribute this fast-healing process to the microporous structure showed by samples, which was corroborated by preparing a nonporous PCL/TPU sample by direct melting blending followed by a hot-pressing step. This sample was cut into two pieces and spliced together in an oven at 70 °C for 150 s. The resulting healed sample showed tensile strength and elongation at break values far less than the original sample, supporting the hypothesis about the importance of the microporous structure involved in the self-healing mechanism. Based on the above, the authors proposed the following self-healing mechanism. When two separated parts are spliced together and irradiated with visible light, the income light is transformed into heat thanks to the photothermal effect displayed by PDA particles. This heat allows a rapid increase in the sample temperature above the melting temperature of PCL. The melted PCL chains present in the crack wet this region and start to diffuse, interpenetrate, and re-entangle with TPU chains.^173,174^ Then, once the light source is removed, a rapid cooling process below the melting temperature of PCL is reached thanks to the microporous structure, where PCL chains are recrystallized, finishing the healing process. Considering the fast and efficient performance of both light-driven properties, the authors visualize the use of this intelligent self-healable photoactuator as a suitable material for biomedical soft robotics applications, potentially useful for the elaboration of microdevices or artificial muscles. The foundation of this idea comes from the expected biocompatibility and biodegradability of the system since PCL/TPU blends have already been studied for biomedical applications.^175^ Therefore, the effect of the PDA inclusion on both properties must be assessed prior to moving toward biomedical applications.

On the same day that Chen’s work was accepted, Dong and collaborators reported the preparation of a NIR-light photoresponsive material after including polyaniline (PANI) nanofibers into an epoxy resin.^176^ The fabricated material stood out by exhibiting simultaneously shape-memory and self-healing properties.^177−180^ The good performance displayed by these materials was ascribed to the outstanding photothermal effect displayed by PANI, allowing the system temperature to increase rapidly in short irradiation times. PANI nanofibers were synthesized by interfacial polymerization.^181^ To achieve this, the authors carried out the process dissolving aniline and ammonium persulfate (APS) in dichloromethane and mixing this solution with an equal amount of HCl (1 M), triggering the polymerization. The obtained fibers, in powder format, were subjected to a careful washing and drying process before its use. SEM analysis revealed a uniform size and an adequate length-to-diameter ratio, while by FTIR spectroscopy the expected chemical structure was corroborated. The UV–vis spectrum recorded for PANI fibers showed three absorption bands centered at 340, 450, and 848 nm, the first being ascribed to π–π* transitions occurring in benzenoid rings while the other two to polaron transitions.^181,182^ The polaron transition at 848 nm would be responsible for the NIR-absorption properties displayed by these nanostructures. Based on the above, the authors successfully confirmed the photothermal properties of these entities by irradiating nanofibers suspensions in furanidine with NIR-light (808 nm) at different power densities. Results showed a notably increase of the temperature was achieved during the irradiation process, reaching higher temperatures in less time at higher power densities. After concluding the characterization, different amounts of PANI nanofibers were added into epoxy-based formulations. Before the curing process, part of the above mixture was trespassed into a Teflon mold, while the rest was deposited onto X70 steel plates using the spin-coating technique. Samples prepared on Teflon molds and those deposited onto steel were labeled as P_*x*_ and EP_*x*_, being *x* a reference for the amount of PANI fibers used (*x* = 0, 1, 3, 5, and 7 for 0, 10, 30, 50, and 70 mg per gram of epoxy resin, respectively). Regarding the samples obtained from molds, DSC measurements showed that the inclusion of nanofibers gradually raises the *T*_g_ of the epoxy system, going from 60 °C for P_0_ to 71 °C for P_7_. Based on these values, the temperature chosen to perform the deformation of these samples was 80 °C. Therefore, all samples were heated at 80 °C during 3 min, reshaped into an L configuration and cooled rapidly to room temperature, fixing the new shape. Then, each sample was irradiated using a NIR-light source (808 nm) at 2 W/cm^2^. With exception of the pure epoxy resin, all nanocomposites were able to recover their initial configurations under irradiation; however, their responses were highly dependent on the nanofibers content. For example, P_1_ required more than 55 s of irradiation to recover its initial shape, while P_5_ achieved it in only 15 s. The slowest shape-memory property displayed by P_1_ would be attributed to the less efficient photothermal conversion ascribed to its low nanofiber content. On the other hand, contrary to the expected, the shape-memory response of the sample having the highest fiber composition (P_7_) was not the fastest. The authors attributed this result probably to a more restricted environment for chain motions due to the numerous PANI fibers. Overall, the mechanism involved in the photoactuation process was intimately related to the photothermal properties of these nanostructures, where through the absorption of NIR photons and their subsequent transformation into heat by means of electron–phonon couplings, the sample rapidly reached temperatures above *T*_g_, triggering its shape recovery led by restoring forces emerged through polymer chains entanglements. On the other hand, the self-healing property displayed by these materials was evaluated after scratching the surface of samples and irradiating them using a 808 nm NIR-light source with a power density of 2 W/cm^2^ for 1 min. Optical images showed that all samples, with the exception of the P_0_, exhibited self-healing behavior under light irradiation. The healing efficiencies of samples P_1_, P_3_, and P_5_ were 60.58, 88.41, and 95.76%, respectively, showing that the healing process was gradually enhanced by increasing the amount of PANI. However, P_7_ did not show a better self-healing ability than P_5_, probably for the same reason given above for its also lower shape-memory function. The healing mechanism explained for these samples was also based on the photothermal effect provided by nanofibers. After the absorption of NIR light, the converted heat is transferred to the epoxy matrix, prompting its temperature rise. Due to the above, the higher mobility acquired by polymer chains around the damaged area allow their relaxation into more stable conformations defined by irreversible net points and polymer chain entanglements. Thereby, the crack closure would be a thermally activated plasticity-driven process. Because P_5_ was the sample with the better self-healing property, this sample was subjected to multiple scratching and irradiation processes on the same area using the same irradiation conditions. After the first damage, results showed a crack width diminishing, going from 254.57 um to 10.79 um during irradiation, equivalent to a 95.76% healing efficiency. Then, after three consecutive cycles, the healing efficiency remained as high as 95%, successfully demonstrating the repeatability of the process. Finally, these nanocomposites were evaluated as self-healing protecting coatings of steel electrodes against corrosion phenomena. Samples EP_*x*_ were immersed into 3.5 wt % NaCl solutions, and then the corrosion potential and corrosion density currents were measured. For the epoxy coating without PANI nanofibers (EP_0_), the corrosion potential shifted negatively while the measured current increased through time, probing that this sample did not offer a good protection. On the other hand, for samples containing nanofibers, the effects of corrosion were diminished to some extent, especially for EP_5_, where the corrosion potential and the measured current density were minimally altered at increasing immersion times. Then, using linear sweep voltammetry, the corrosion resistance of scratched and healed samples was tested. It was observed for EP_0_ that, after the damage, the measured current rapidly increased, and after 1 min of NIR irradiation, the current value was even greater than the initial. Conversely, samples coated with nanocomposites displayed a notorious diminishing of the observed current after being irradiated, indicating that the scratched area was successfully healed and thus the metal surface isolated from the corrosive medium.

**Figure 14:**
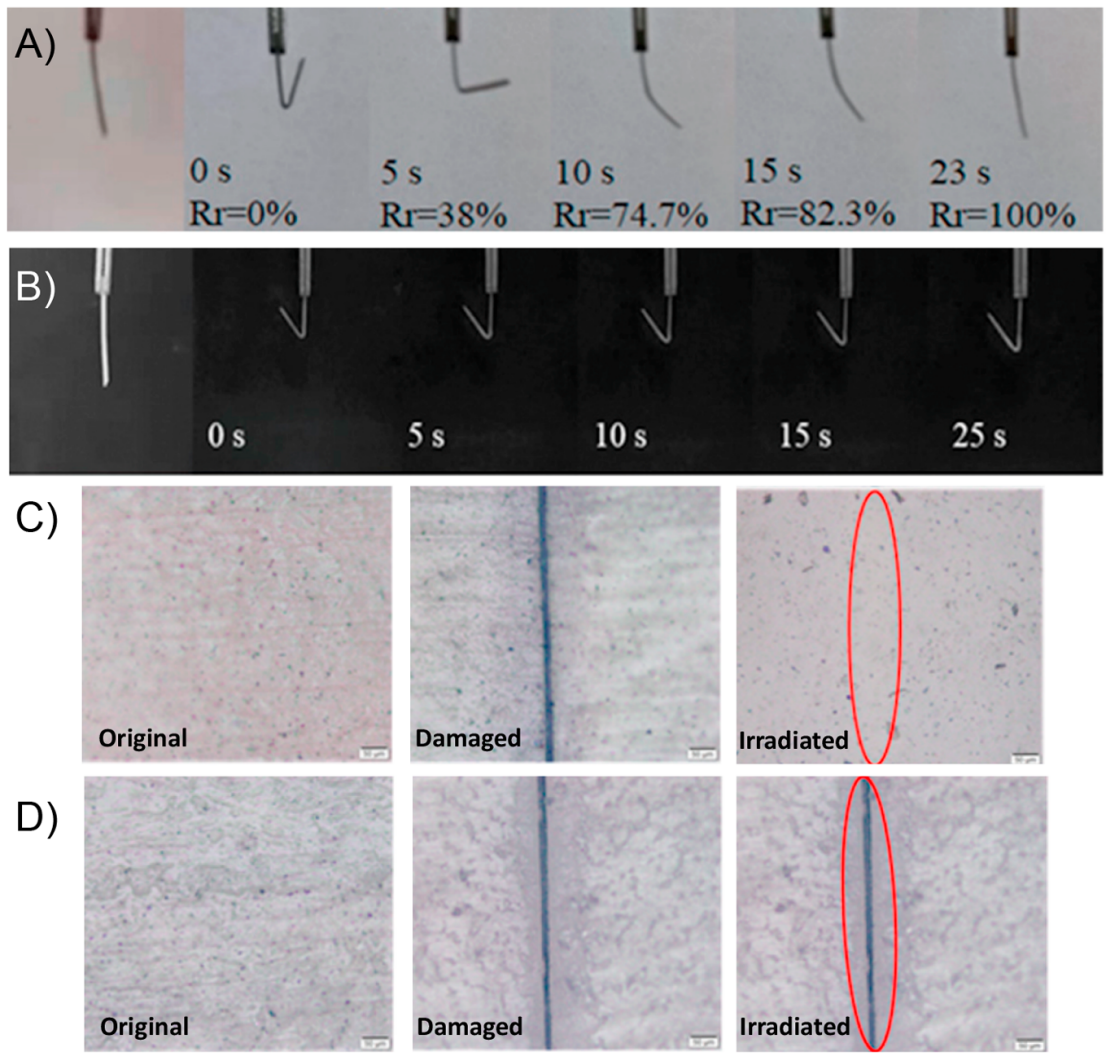
Digital images of the evaluated photoinduced shape-memory process of PU with (A) and without copper sulfide nanoparticles (B). Optical microscope images of the PU-based nanocomposite (C) and pristine PU film (D), showing their original, damaged, and irradiated state. Adapted with permission from ref (183). Copyright 2020 Elsevier.

Two months later, Wang and his co-workers developed a PU-based ultraefficient photoactuator self-healing composite containing copper sulfide nanoparticles (CuSNPs).^183^ The light-induced shape-memory property was investigated by shape fixation followed by shape recovery. Initially, a straight strip was bent mechanically at 70 °C and further cooled to fix the shape. The shape of the nanocomposite having CuSNPs was gradually recovered to the original configuration when it was irradiated by NIR light (Figure 14A). In contrast, the composite without nanoparticles failed to recover under similar conditions (Figure 14B). Here, CuSNPs act as a photothermal agent.^184,185^ Upon exposure to the laser light, nanoparticles absorb the light energy, converting it into heat, which is later irradiated across the structure, inducing the bending of the strip. Further, when the irradiation time increases, the local temperature of the matrix increases above the soft segment *T*_g_, and subsequently, the strain energy release drives the shape-recovery function. The composite film’s photoactuation and self-healing behaviors largely depend on the amounts of photothermal agent present in the film.^26^ On the other hand, the phototriggered self-healing behavior of the nanocomposite film was investigated under NIR irradiation. The crack present in the composite film disappeared after 1 min of laser irradiation followed by cooling (Figure 14C). It must be mentioned that in absence of CuSNPs, PU films did not show self-healing ability (Figure 14D). The healed sample showed almost complete recovery of the mechanical properties compared to that of the original. The self-healing mechanism was explained considering three steps: (1) localized thermal shrinkage near the healing area during NIR irradiation, (2) liquefaction of polymer matrix and polymer chain interdiffusion from both sides of the fracture, and (3) solidification of the polymer chains after light cessation. Despite the interesting results of this work, it should be noted that there are also a few composite films doped with AuNPs^186^ or graphene^187^ with a more efficient self-healing property than that of the material in this report. However, the lower cost of copper-based materials is a parameter to be considered and that could outweigh the lower performance shown.

**Figure 15:**
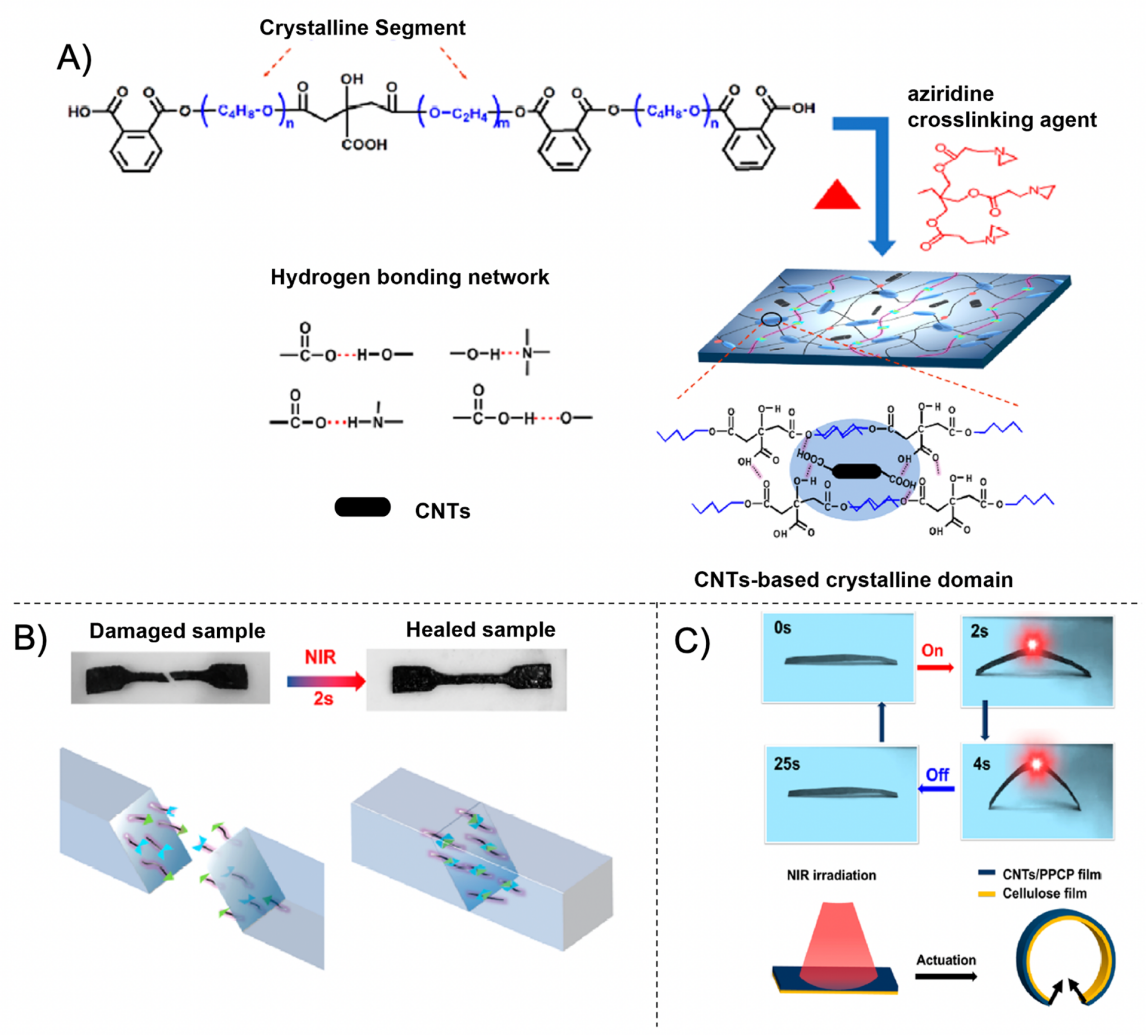
(A) Chemical structure of supramolecular cross-linked elastomer. (B) NIR-accelerated superfast self-healing process. (C) Photographs of a strip-shaped actuator reversibly bending when the NIR light is switched on and off (up) along with a diagram of a bilayer actuation (bending) on exposure to NIR (down). Adapted with permission from ref (188). Copyright 2020 American Chemical Society.

Later, in the same year, Zhang and his co-workers reported a near-infrared photoresponsive soft actuator based on the synergistic effects arising from a crystalline physical cross-linked network supporting by hydrogen bonding interactions.^188^ The macromolecular system (named PCCP) was made by following a one-pot condensation polymerization of PEG, polytetramethylene ether glycol (PTMG), citric acid (CA), and phthalic anhydride (PA) (Figure 15A). Then, bilayered actuators were made using a NIR-photoactive PPCP layer containing dispersed carbon nanotubes (CNTs) joined with an extra NIR-inactive cellulose layer. The actuator showed a wide range of properties, including fast and reversible NIR-driven actuation behavior with a bending angle over 90° in 1.6 s, strong mechanical strength (12.52 MPa), excellent self-healing speed (2 s), and reasonable self-healing efficiency during both mechanical (87.68%) and actuating (99.50%) performance (Figure 15B). The crystalline physical cross-linked network originates from PEG and polytetramethylene ether glycol (PTMG) segments, together with hydrogen bonding interactions taking place between carboxyl and hydroxyl groups, are synergistically responsible for the excellent self-healing and reconfiguration properties shown by the material as well as its good mechanical strength. On exposure to NIR light (808 nm, 0.5 W/cm^2^), the CNTs-based crystalline domains in the photoactive elastomer layer absorb optical energy and transfer heat through the CNT’s thermal conductive network, resulting in a bending motion toward the cellulose side.^189,190^ Moreover, the reversibility of the photoactuation function was successfully corroborated by light on/off cycles (Figure 15C). This actuator is reconfigurable by applying temperature followed by light irradiation, but it cannot be recycled.

**Figure 16:**
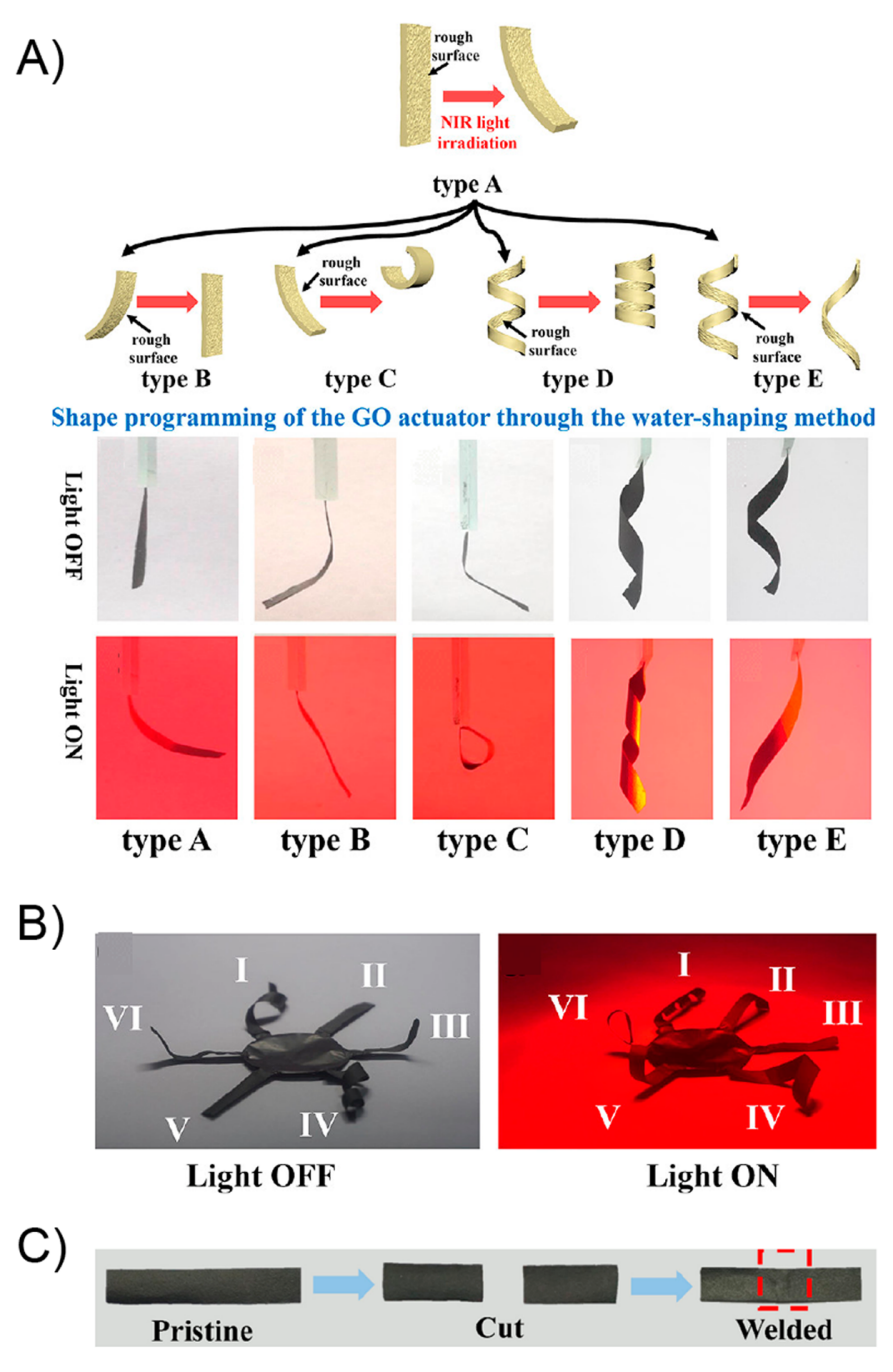
(A) Digital photographs of the initial states of actuators of types A, B, C, D, and E, through water shaping. (B) Digital photograph of the octopus actuator manufactured through water welding method. (C) Photographs of the self-healing procedure of GO film. Adapted and modified with permission from ref (191). Copyright 2020 American Chemical Society.

Nowadays, it is a great challenge to achieve different complex shape morphing light-driven self-healing actuators using conventional fabrication methods, and by the end of 2020, Weng et al. successfully achieved this goal by developing a facile strategy to obtain programmable GO-based light-driven actuators with the advantages of multiple shape designs, programmability, and self-healing function.^191^ The GO film with asymmetric surface morphology was made by directly casting the GO suspension solution on the rough poly(dimethylsiloxane) (PDMS) template. The programming of the initial shapes of the GO actuator were done using a water-shaping method described as follows: the GO films were fixed into predesigned strained shapes and exposed to water mist, whereby the inner stress could be effectively dissipated, allowing the slide of GO sheets against each other inside the strained films, fulfilling the reprogramming process. Different types of NIR-responsive actuators were fabricated using the above-mentioned method (Figure 16A), which exhibited bending, unbending, twisting, and untwisting light-driven motions. The interplay between the photothermal effect and the asymmetric morphology on the opposite surfaces resulted in the bending motion upon exposure to NIR light irradiation. It is essential to mention that GO actuators can be further programmed in various other shapes, e.g., octopus and tendril-shape actuators, through the complementary use of the water-shaping and water-welding methods. The self-healing behavior of the GO actuators films was demonstrated by reconnecting the two cut portions of the film (Figure 16B). The separated GO films can be easily welded by adding water droplets to the damaged area and then evaporating for 30 min. In terms of tensile strength and Young’s modulus values, the healed film shows almost the same mechanical strength compared to the original film. The high healing efficiency showed by this system was supported by previously reported results.^192,193^ The healing mechanism was explained by reversible hydrogen bonding of graphene oxide. GO is a moisture-sensitive material and has a high affinity with water, thereby, when water droplets were added to the damage site, the rebuilding of hydrogen bonds and the reorganization of GO sheets within the damaged part of the film take place, inducing the formation of a dense and uniform joint at the fracture position.^193,194^

**Figure 17:**
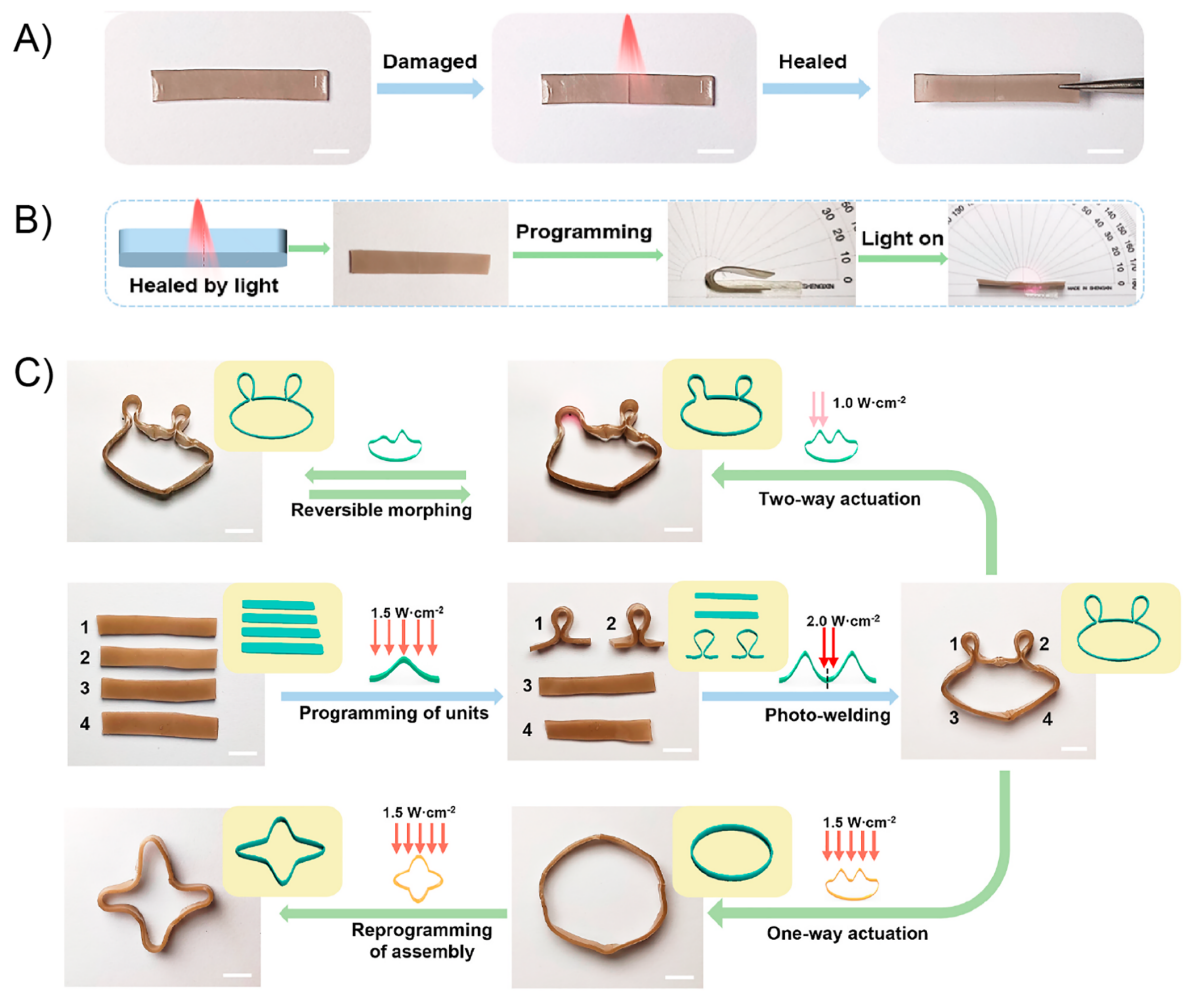
(A) Photographs showing light-driven self-healing behavior of AgNWs/EVA nanocomposites (scale bar = 6 mm). (B) Scheme of the light-triggered healing process and images of one-way shape-memory process of the healed sample. (C) Schematic illustration and photographs showing the confection of a 3D-assembly executing a series of combinational light-driven motion tasks referred to reversible shape transformation, reconfiguration, and reprograming (scale bar = 1 cm). Adapted and modified with permission from ref (195). Copyright 2020 American Chemical Society.

By aiming to contribute to the new era of soft robotics and its related high-tech fields, at the beginning of 2021, Liu and collaborators designed a new, facile, and cost-effective strategy to develop a material exhibiting both light-driven shape-memory and self-healing properties, accompanied by a fast 3D-assembly ability based on the photowelding phenomenon.^195^ More importantly, as a novelty, the authors also evaluated the photoactuation property in air and underwater, expanding the study of polymeric actuators having the ability to rapidly adapt to changing environments.^196^ To achieve this, the authors dispersed silver nanowires (AgNWs) within a semicrystalline poly(ethylene-*co*-vinyl acetate) (EVA) matrix. The obtained AgNWs (around 10 μm of length and 50 nm of diameter) were suspended in ethanol and added to hot toluene in which, later, EVA and dicumyl peroxide (DCP) were dissolved. The mixture was evaporated by allowing the obtainment of a solid membrane which was subjected to consecutive hot-pressing steps. Using the above protocol three samples with increasing amounts of AgNWs were prepared: 0.5, 1.0, and 3.0 wt % AgNWs/EVA. SEM analysis showed homogeneous distributions of AgNWs within the polymer matrix, confirming the effectiveness of the preparation method. UV–vis–NIR spectra of all samples were recorded, showing that, while pristine EVA revealed a weak absorption property between 400 and 900 nm, all nanocomposites, even 0.5 wt % AgNWs/EVA, exhibited a strong absorption over the whole region. In this sense, it was feasible to expect a remarkably photothermal effect using UV, visible, or near-infrared (NIR) light; however, the authors focused efforts on using near-infrared irradiation. In this regard, samples were irradiated with a NIR laser at 808 nm with an intensity of 1.5 W/cm^2^, and their temperature increases were registered by using a hand-held infrared camera. After 70 s of irradiation, while pure EVA did not show a measurable increase of temperature, samples 0.5, 1.0, and 3.0 wt % AgNWs/EVA reached values of 136, 150, and 221 °C, respectively, corroborating the outstanding photothermal effect displayed by AgNWs.^197,198^ In addition, the authors also studied the effect of light intensity on the temperature rise in these materials, achieving significantly higher temperatures by slightly increasing the laser power. Once corroborated the successful light-to-heat conversion of these systems, their thermal characterization was evaluated in terms of DSC. For all samples, results showed the presence of melting phenomena represented by a broad and asymmetrical peak that begins to appear around room temperature (*T*_m,low_) and whose maximum is located around ≈78 °C (*T*_m,high_). This broad temperature range is traduced as heterogeneity in terms of crystalline domains having dissimilar melting temperatures (*T*_m_), which is the base for the actuation motions observed in these nanocomposites. Regarding the above, by controlling the time and intensity of NIR irradiation, the temperature of the sample can be modulated to be below or above *T*_m,high_. To study the shape-memory function, samples were first molded into “U” configurations after being heated to temperatures above *T*_m,high_ by NIR irradiation. This new configuration remains stable after cooling the sample to room temperature due to the “freezing” of polymer chain motions. Then, bent samples were again irradiated, searching for conditions in which their temperatures fall within the range *T*_m,low_–*T*_m,high_. Thereby, all domains with *T*_m_ values above the sample temperature would act as rigid segments giving structural stability to the system, while those having lower values would serve as actuation domains, in which, due to the melting of crystals, the irradiated area dilates and, as a consequence, the “U” configuration is expanded. Conversely, when the light source is turned off, the recrystallization of chains promotes the shrinking of the sample and the recovering of the “U” shape. Therefore, these nanocomposites displayed a reversible light-triggered morphing behavior ascribed to melting/recrystallization phenomena activated by the photothermal effect of AgNWs. Importantly, all AgNWs/EVA samples achieved maximum bent angles of 180° and exhibited excellent actuation speed, even for those samples with low AgNWs loadings or irradiated with low-intensity light sources (808 nm, 1.6 W/cm^2^). Particularly, upon NIR irradiation (808 nm, 2.3 W/cm^2^), the sample 3.0 wt % AgNWs/EVA required around 6 s to increase its bending angle from 0° to 27.5° and 15 s to turn back to its initial state when the light was turned off. Moreover, no significant diminishing of bending angle values was observed after several on/off light cycles, demonstrating the robustness of the actuator performance. Surprisingly, the photoactuation property was also achieved underwater, where slight differences in terms of performance would be ascribed to the water absorption by the sample. Using a 10 W/cm^2^ NIR source (808 nm), the bending angle of 3.0 wt % AgNWs/EVA increased from 0° to 25° in 4 s. Interestingly, when the light was turned off, the angle returned to its initial value in only 5 s, notably faster than in dried state. This was attributed to a more efficient cooling process provided by the aqueous environment. Furthermore, underwater, the authors evaluated the repeatability of the actuation property by exposing samples to consecutive cycles of light/dark conditions, demonstrating the high stability of their photomorphing behavior. The outstanding photothermal effect displayed by AgNWs not only allowed these materials to exhibit light-driven shape-memory motions but also endows them with the ability to self-repair. Regarding the above, one of the strong points of this work was verifying the actuation property of healed samples because most of the works referred to self-healable actuators that evaluated both properties independently. In this sense, authors tested the phototriggered self-healing ability by cutting a piece of sample into two halves, joining them together, and irradiating the damaged zone for 1 h using a NIR laser source (808 nm, 1.5 W/cm^2^). As a result, a single piece of material was obtained with no visible scars under the naked eye evaluation (Figure 17A). Afterward, the healed sample was bent into a “U” configuration following above-mentioned protocols, and after being exposed to NIR illumination, the sample was able to recover completely its initial shape (Figure 17B). The authors proposed a healing mechanism consisting of the merging of two complementary processes activated by the photothermal effect. The first one is referred to the re-entanglements of EVA chains promoted by their increased mobility, while the second would be ascribed to the recross-linking process between monomeric units due to the thermal decomposition of some remaining DPC entities within the material. The hypothesis about the DPC-mediated recross-linking process was successfully corroborated by checking, theoretically^199^ and experimentally (i.e., using DMA), the increase of the cross-linking density inside the material. However, after multiple healing-damage cycles, the healing property of these materials exhibited a diminishing in its performance. The above was attributed to the consumption of remnant DPC molecules after consecutive irradiation steps, forcing the healing process to rely mainly on the re-entanglements of polymer chains. In addition to the above, the fall of the healing efficiency could also be due to the carbonization of EVA fragments due to much light exposure. From our perspective, one of the possible limitations arisen from this work would be related to the participation of DPC species in the healing process. The above since an essential fraction of these entities could be decomposed in the hot-pressing step during sample preparation. Therefore, the amount of remnant DPC within the final material would not be necessarily a constant parameter, directly affecting the reproducibility of the self-healing results. Notwithstanding the above, the excellent self-healing ability showed by these materials allows them to be easily and rapidly assembled into complex 3D configurations. These assembled configurations, exhibiting light-triggered self-healing ability and reversible photoactuation motions, can be considered as soft robots capable of being reconfigured locally and remotely using light irradiation (Figure 17C).

**Figure 18:**
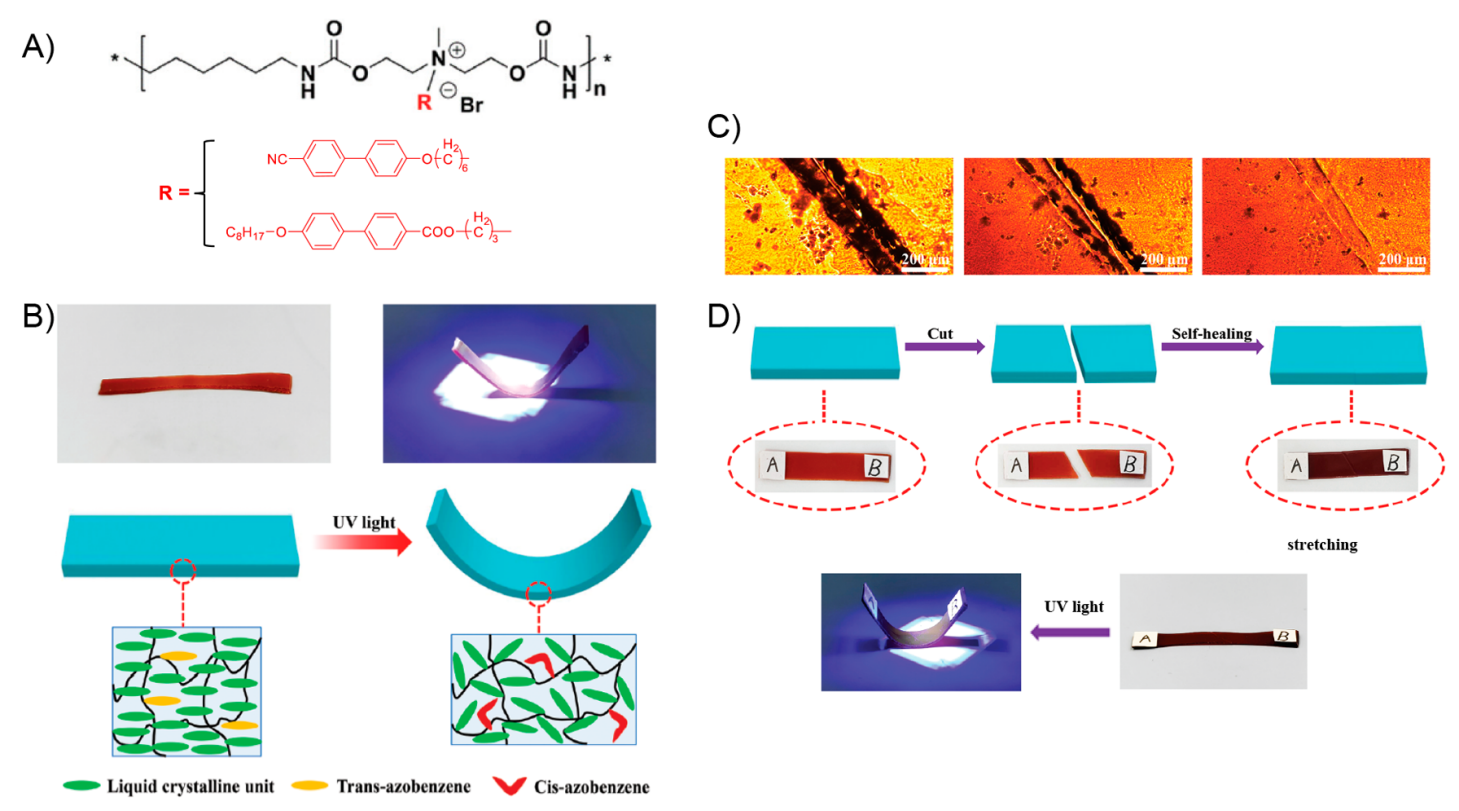
(A) Chemical structure of liquid crystalline PU having crystalline group in the pendent chain. (B) Bending movement of the film before and after irradiation with UV light and schematic illustration of the possible mechanism of light-responsive behavior of the actuator film. (C) UV light driven self-healing process visualized using polarized optical microscopy (POM). (D) Photograph of reconnected film after the self-healing process followed by its stretching and subsequent photobending motion. Adapted with permission from ref (200). Copyright 2021 Royal Society of Chemistry.

Shen and co-workers introduced again the use of azo-containing polymers in the developing of a liquid crystalline PU film that revealed a fast light-responsive actuation property along with self-healing capability.^200^ This system was prepared by quaternization between an ordinary PU and molecular structures typically used in the elaboration of polymers displaying liquid crystalline-like behavior (Figure 18A).^201−203^ The photoinduced actuation behavior was observed under exposure to UV light (365 nm, 50 mW cm^–2^) at room temperature (Figure 18B). After exposure to the UV light, the film was bent toward the light source within 5 s and back to the initial stage (7 s) after the UV light was turned off. The well-known reversible photoisomerization of azobenzene units allows inducing of changes in the internal structure of the material, triggered by the rearrangement of these photoactive units.^204−206^ In this way, the actuation motions performed by the system would be ascribed to changes in the phase of mesogenic units going from a homogeneous arrangement to a disordered state, thereby, during this transition, a local volume contraction of the film occurred. On the other hand, the self-healing behavior of the film was investigated by monitoring the changes in crack status by optical microscopy at 100 °C. After 10 h of thermal treatment, the crack was healed properly (Figure 18C). Furthermore, the self-healing capability was explored by overlapping the two pieces of cut film at 100 °C for 10 h. After the self-healing process, the film was reconnected and exhibited a particular tensile strength with a maximum strain above 200%. Aiming to test the robustness of the material, the healed film was stretched to an elongation ratio of 80% and then exposed to UV irradiation, showing the retention of the photoactuation function (Figure 18D). The self-healing property was explained by the higher mobility that polymer chains acquire at high temperatures, where the diffusion and re-entanglement processes along the damage area are favored. In addition, hydrogen bonding between amide bonds and the electrostatic interaction between ionic species may also drive the self-healing process.

**Figure 19:**
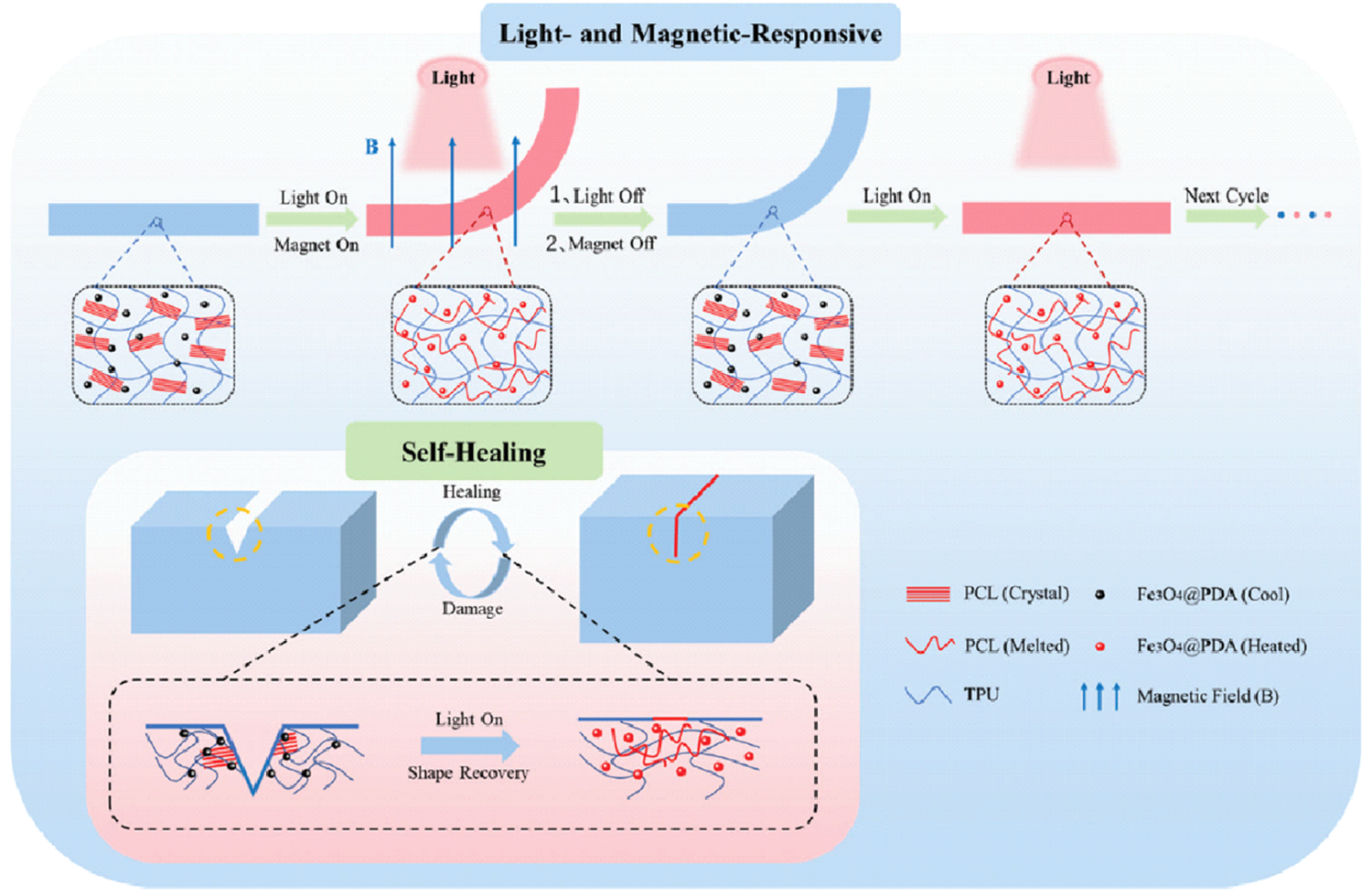
Schematic presentation light- and magnetic-responsive actuation and shape-memory assisted self-healing. Adapted with permission from ref (210). Copyright 2021 Royal Society of Chemistry.

In March 2021, around one year after their previous publication, Chen et al. developed a similar light-responsive shape-memory material by using, again, PCL, TPU, and PDA as photothermal agents. As mentioned before, polydopamine (PDA) has gained much attention and has been extensively used for treatment of cancer and shape-memory materials because of its efficient light-into-heat conversion.^207−209^ However, in this opportunity, they decided to go further with this system, endowing it with a magnetic response function replacing PDA by Fe_3_O_4_@PDA core–shell nanostructures (Figure 19).^210^ In this regard, PDA was coated on the surface of Fe_3_O_4_ magnetic nanostructures through self-polymerization, gaining better miscibility with the polymer matrix. Thereby, Fe_3_O_4_@PDA NPs were successfully embedded into a PCL/TPU mixture (10 wt % content), endowing the final material with a magnetic responsive behavior and the ability to convert efficiently light into heat.^211^ Experimentally, it was observed that under visible light (light intensity 0.2 W/cm^2^), PCL/TPU/Fe_3_O_4_@PDA nanocomposites fixed in temporary U-shaped film recover very fast within 30 s. In addition, both photothermal heating and magnetic-responsive actuation were also explored in a cantilever experiment. Initially, the shape of the cantilever was fixed by applying light, followed by the magnetic field. Then, removing the magnet and further illumination of light on the bent cantilever will help to recover to its initial flat position. Moreover, as was expected, this nanocomposite exhibits excellent self-healing ability under light irradiation. After the exposure of the light (0.2 W cm^–2^) for 60 s, the cracked surface of the prestretched specimen disappeared completely after 120 s. Upon absorption of light energy, the photoexcitation of the Fe_3_O_4_@PDA NPs took place, leading to heat generation, which helps the polymer chain mobility near the crack area. The self-healing efficiency was further quantitatively investigated by stress–strain curves. After healing 3 times, self-healing efficiency was calculated to be 88.3%, confirming the excellent reversibility of the process and, thereby, the robustness of the material.

**Figure 20:**
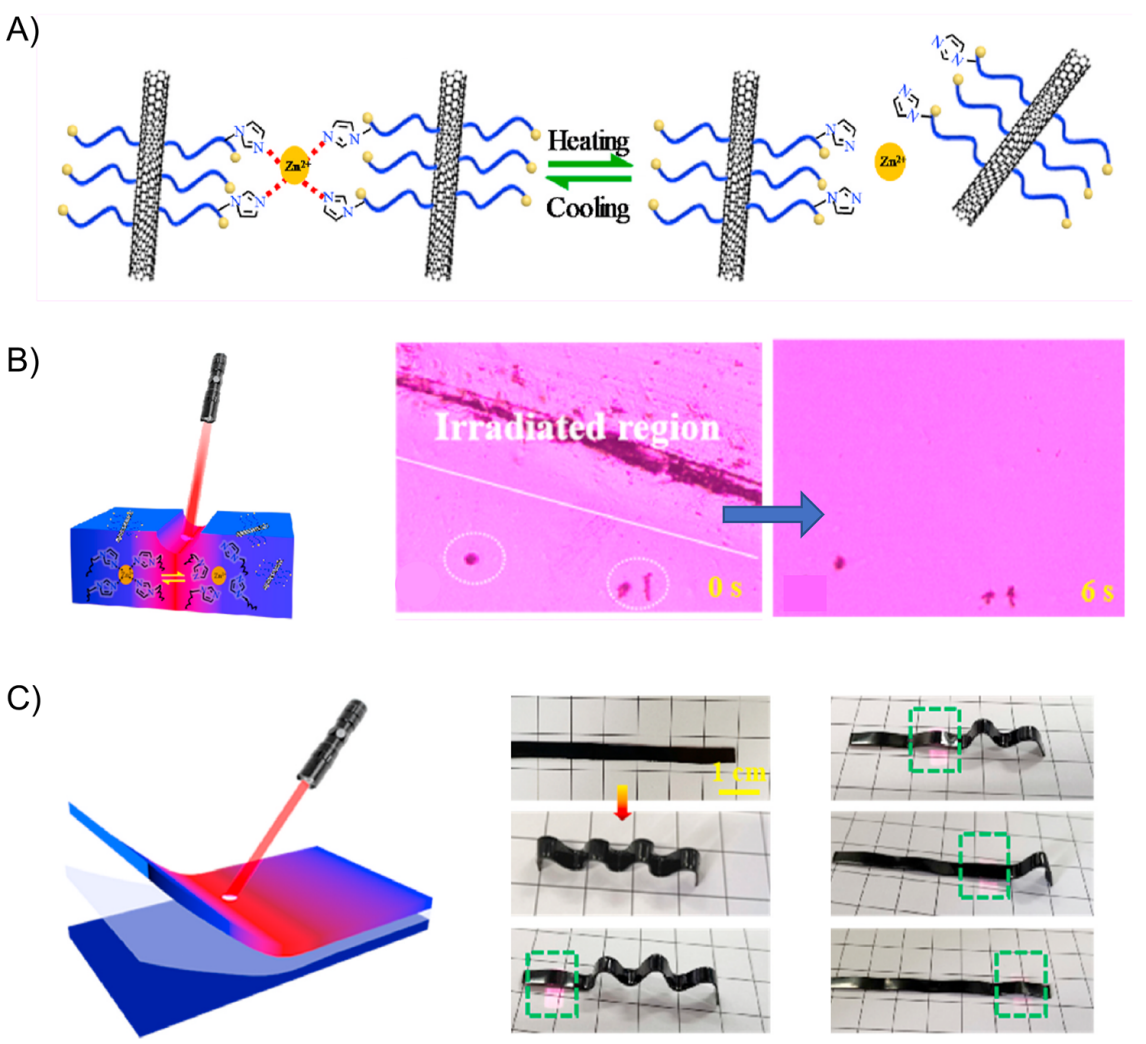
(A) Schematic diagram of the association and disassociation process of Zn^2+^/vinyl imidazole (VI) metal–ligand bonds in response to stimulus. (B) NIR light driven self-healing process followed by POM and (C) targeted shape-morphing process in waved shape of the CNTs*x*-*g*-CP*y*/Zn sample. Adapted with permission from ref (212). Copyright 2021 Elsevier.

A month later, Wang and co-workers designed and devolved a new thermoplastic CNTs-based shape-memory polymer with thermal and NIR light-induced actuation.^212^ The supramolecular cross-linked nature of the CNTs-*graft*-poly(tetrahydrofurfuryl methacrylate-*co*-lauryl acrylate-*co*-1-vinyl imidazole) nanocomposite (labeled as CNTs*x*-*g*-CP*y)* was successfully made via reversible addition–fragmentation chain-transfer (RAFT) polymerization followed by metal–ligand interactions generated after the inclusion of zinc ions (Figure 20A). The metal–ligand interaction between Zn^2+^ and imidazole moiety was introduced into CNTs*x*-*g*-CP*y* samples to build a dynamic supramolecular cross-linked network, which provides an excellent and rapid multiple shape-memory function along with a fast self-healing property, both activated under NIR light irradiation or external heat. The composite having CNTs content of 1.1 wt % showed maximum stress of 1.68 MPa and an elongation at break of 450%. The self-healing process was investigated under an optical microscope by monitoring the crack of a fractured specimen after indirectly and accurately heating with NIR light (Figure 20B). It was observed that the crack completely healed after 6 s of light exposure. Furthermore, the light-induced shape-memory actuation behavior was investigated by shape fixation followed by shape recovery (Figure 20C). Initially, a CNT_1.1_-*g*-CP_3_/Zn sample having four arches and a waved temporary shape was fabricated. Then, the light was exposed separately on each arc one by one, resulting in the recovery of the initial flat shape. The average shape recovery ratio of waved shape film was 93.6%. It is worth mentioning that, upon exposure to NIR light, the photothermal conversion of CNTs can effectively trigger the association and dissociation process of the dynamic metallosupramolecular bond between vinyl imidazole and Zn^2+^, leading to an excellent rapid multiple shape-memory and precise self-healing efficiency.

**Figure 21:**
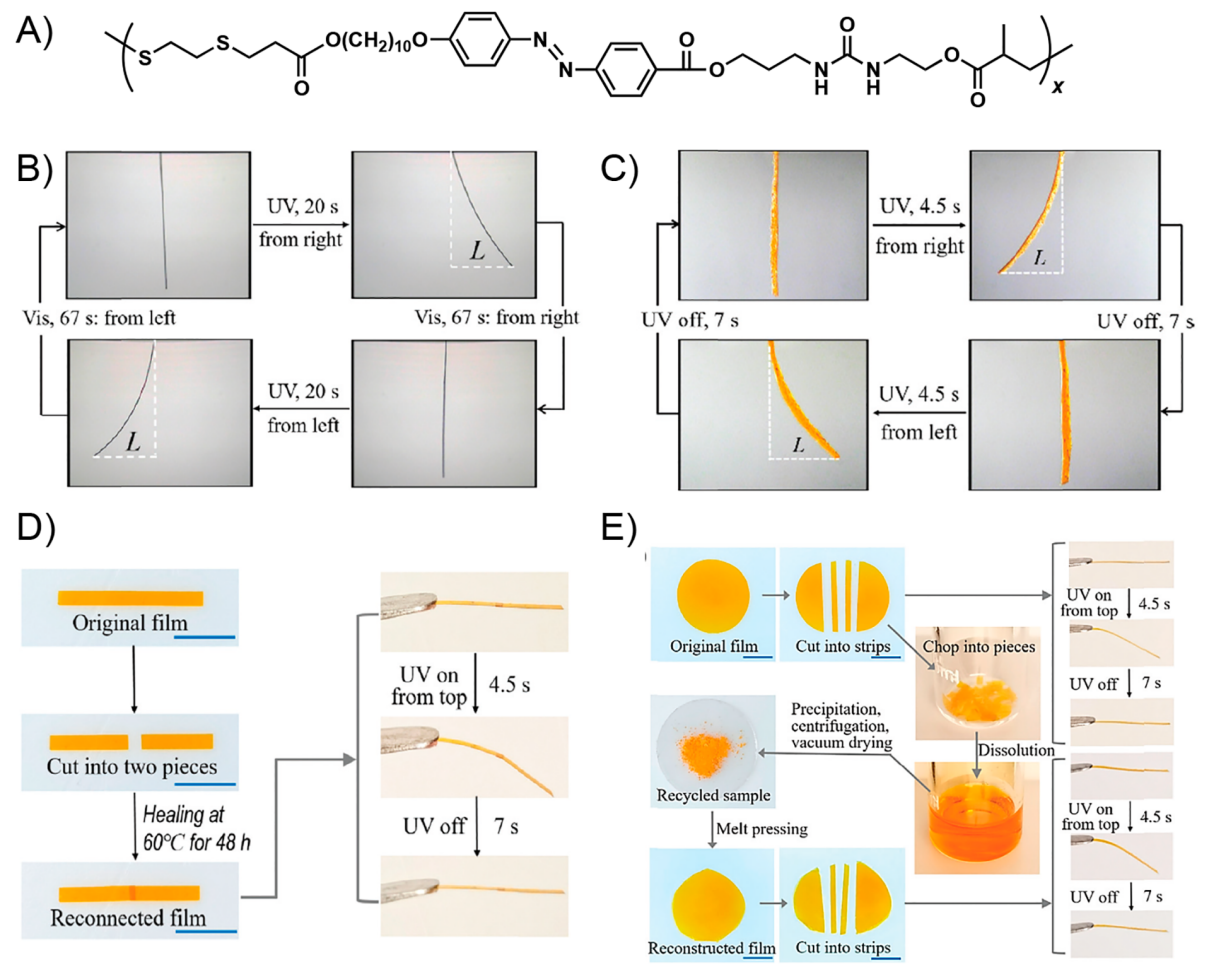
Chemical structure of polymer (PEU-10) prepared via Michael addition reaction. Photographs of a PEU-10 fiber (B) and PEU-10 film (C) displaying photoinduced bending and unbending motions. (D) Self-healing process of a photodeformable PEU-10 film and its photoactuation function after being healed. (E) Reprocesability and recyclability of a PEU-10 film showing no loss of photoactuation function. Adapted with permission from ref (213). Copyright 2021 Royal Society of Chemistry.

To date, the last report assigned to a self-healable polymeric photoactuator is attributed to Wang and collaborators.^213^ By preparing a liquid crystalline poly(ester-urea) bearing azobenzene moieties in the main-chain (PEU-10) (Figure 21A), they recently developed a light-driven actuator showing efficient room temperature self-healing ability. This work is in line with the few previous ones reported that demonstrated effectively that azo-containing polymers are a class of smart soft materials capable of efficiently converting light into mechanical energy, which can be traduced in photoinduced macroscopic motions under specific conditions.^214,215^ PEU-10 was synthesized from an ester and urea unit-containing azo monomers with acrylate/methacrylate end-groups and 1,2-ethanedithiol via Michael addition reaction. Then, PEU was prepared in two different formats: uniaxially oriented fibers and films with homeotropic azo mesongen alignment. PEU-10 showed reversible photoinduced bending and unbending motions because of the trans–cis isomerization of azo units and, consequently, by their alignment.^216^ In this sense, supported by previous reports, the authors argued that the motions observed in the material would be related to contraction and expansion forces triggered on the surface of the sample (top layer) by irradiating it with different types of lights or also by withdrawing the light stimuli, depending the case.^136,217−219^ They also found that the rates of bending and unbending movements could be notably enhanced by incrementing the temperature of the surrounding media.^220,221^ Thereby, in the case of PEU-10 fibers, upon UV light exposure (365 nm), the sample’s surfaces bend toward the light source in 20 s. Then, the irradiation with visible light (>510 nm) on the bending site triggers the azo entities cis-to-trans isomerization, promoting the unbending of the fiber in around 67 s (Figure 21B). On the other hand, by turning on/off a UV light source, PEU-10 films also delivered a reversible photoactuation function but showing opposite behavior to fibers in terms of the direction of the bending/unbending motion. Thus, upon UV irradiation, the PEU-10 film bent away from the light source in 4.5 s, while after turning off the UV light, the film restored its initial shape automatically in only 7 s (Figure 21C). The faster movements exhibited by the films were attributed to the greater rigidity achieved in the material when it is prepared in this type of format. Based on the above, and regardless of the format utilized, PEU-10 was able to achieve photoinduced motions at room temperature and in a reversible manner. Indeed, the robustness of both types of samples was successfully demonstrated by showing that the time required to achieve the bending motion as well as the angle value, remained almost constant over 100 consecutive bending/unbending cycles. Later, the self-healability of a PEU-10 film was studied by overlapping two pieces of a previously bisected film at 60 °C for 48 h (Figure 21D). Using the original sample as reference, the recovery values for the yield strength and elongation at break of the reconnected sample were 87% and 64%, respectively. Moreover, the healed film was able to lift a counter-weight 14570 times heavier than its own weight. Additionally, the healed film maintained unaltered its photoactuation function as is shown in Figure 21B. Surprisingly, the film was reprocessed and recycled at room temperature following conventional protocols, showing no remarkable differences in terms of actuation motions when compared to the original sample (Figure 21E). These types of polymers having multifunctional main-chain azo crystalline units are promising candidates for fabricating various self-healable photoactuators with desired 3D shapes and with different actuation behavior at room temperature.

Very recently, Zhang et al. developed a new strategy to fabricate dynamic cross-linked polyurea (DCPU) and DCPU/PDA composites via in situ photoinitiated copolymerization using UV light.^222^ The nanocomposites were obtained as films (dimensions = 80 × 60 × 1.0 mm^3^) with a good distribution of DPA in the DCPU matrix as observed by SEM imaging. These materials showed high thermomechanical properties, with a tensile strength of 6.74 ± 1 MPa, an elongation at breaking of 334 ± 20%, and a toughness of 14.96 ± 2 MJ m^–3^. These values resulted much higher than those showed by pure DCPU. By working with different concentrations of DPA, the authors demonstrated that the swelling degree decreased and the cross-linking density increased with increasing the PDA content. The thermal behaviors of DCPU and DCPU/PDA nanocomposites were evaluated by DSC, which showed a *T*_g_ of 52.3 °C for DCPU, while the *T*_g_ of the DCPU/PDA nanocomposites increased notably from 53.8 to 57.7 °C as the PDA content increased from 0.5 to 1.5 wt %. Such behavior can be attributed to the restricted motion of polymer chains and strong interactions between PDA and the polymer matrix. It is worth mentioning that PDA was chosen in this study as a multifunctional nanofiller due to the presence of various functional groups (especially amino and hydroxyl groups), which causes the formation of intermolecular hydrogen bonds and strong interfacial interactions with the −NH and −C=O groups of DCPU, making the two polymers highly compatible. However, the mechanical behavior showed that increasing the PDA content restricts the chain mobility of the polymer and limits its stretchability, which was in good agreement with the stress-relaxation tests. Additionally, the nanocomposite films also displayed a remarkable photothermal response, NIR-induced shape-memory actuation behavior and self-healing capability (upon 3 h of NIR irradiation). Specifically, DCPU/PDA with 1.0 wt % of PDA could be remolded multiple times under 10 MPa at 90 °C for 30 min. It should be emphasized that the recycled composite exhibited no remarkable fatigue in ultimate tensile strength, toughness, or elongation at break, even after the third cycle, indicating an excellent performance for such soft actuators. The excellent multicycled self-healing ability of the nanocomposite was ascribed to the combined effect of the photothermal effect of PDA and dynamic urea bonds. Furthermore, the photothermal actuation behavior was explored under NIR light irradiation (λ = 808 nm, 0.5 W cm^–2^). Initially, the spiral shape of the film (50 × 5 × 1 mm^3^) was fixed using a metal cylinder by consecutive heating, deformation, and cooling. Under NIR irradiation, the surface temperatures of a DCPU/PDA film rapidly rose to 155.2 °C in 30 s. After exposure to the NIR light, the spiral shape film started to show actuation behavior within 5 s and ultimately spread out within 60 s of NIR irradiation.

### 2.2 Self-Healing Magnetic Actuators

**Figure 22:**
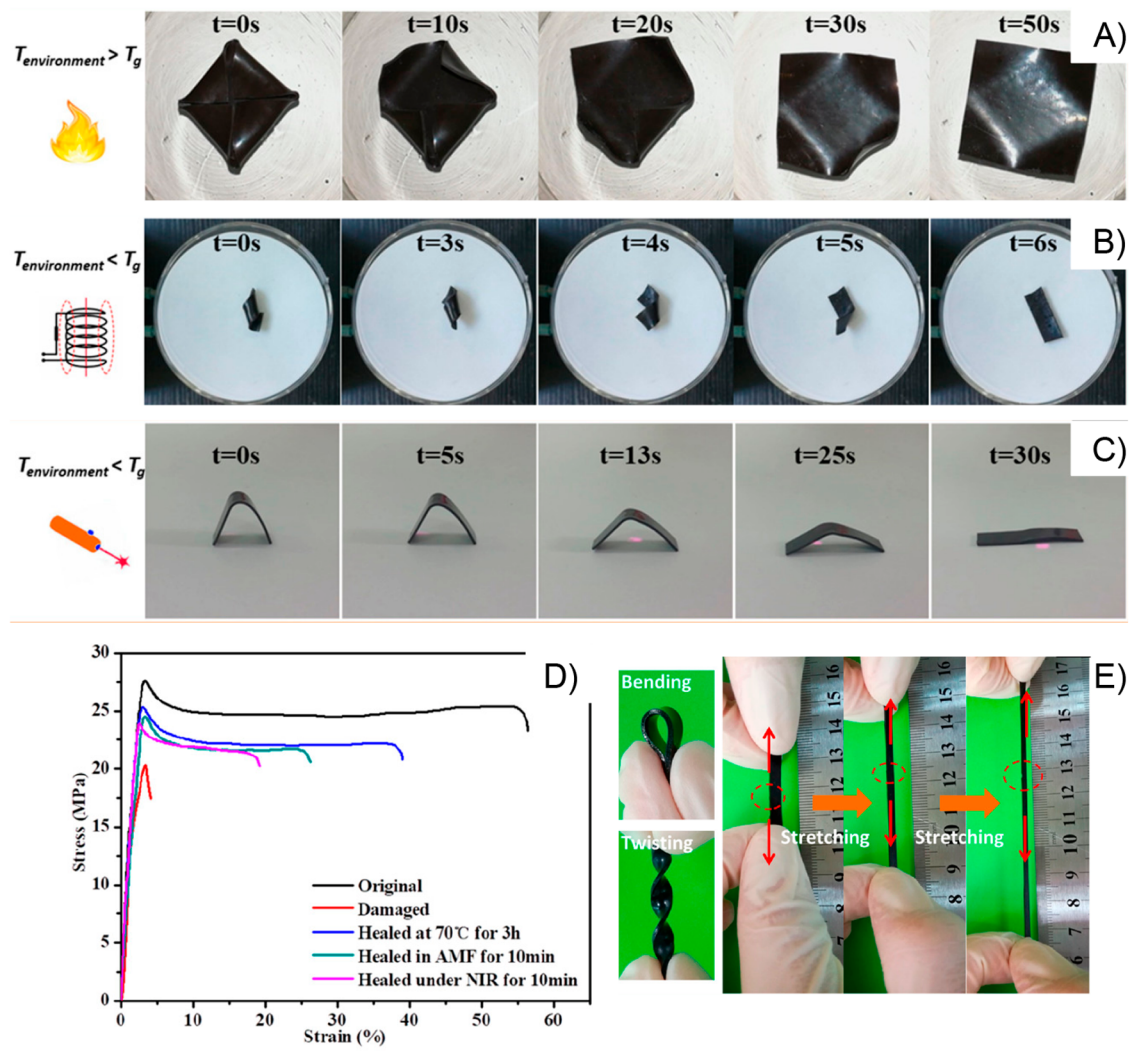
Digital photographs of shape-memory behavior of P7E3F: (A) folded shapes in thermostat at 70 °C, (B) spiral shapes in an alternating magnetic field (45 kHz), and (C) folded shapes under NIR light. (D) Stress–strain curves of original, damage and healed P7E3F samples. (E) Photographs of the self-healing behavior with different shapes after healing. Adapted and modified with permission from ref (223). Copyright 2018 American Chemical Society.

In 2018, Huang and co-workers reported a series of novel self-healing thermoplastic vulcanizates (TPVs), which showed excellent thermal/magnetic/light-triggered shape-memory assisted self-healing behavior.^223^ The damage on polylactide (PLA)/epoxidized natural rubber (ENR)/Fe_3_O_4_ TPVs was healed via events that are improved by synergic effects. In the first place, the shape-memory effect displayed by the material favors the physical contact between damaged surfaces, in addition, the interdiffusion of ENR chains induced by desorption–absorption processes of ENR-Fe_3_O_4_ bonded to rubber that allows the healing of the ENR phase and, by last, the rearranging and re-entanglement of PLA segments that also would promote the repair of TPVs. Experimentally, the PLA/ENR/Fe_3_O_4_ TPVs were prepared at 150 °C with a PLA/ENR weight ratio changing from 90/10 wt % to 50/50 wt %. In terms of nomenclature, and for the sake of clarity, a sample labeled P7E3F1 would consist of a 70/30 PLA/ENR weight ratio with a loading amount of Fe_3_O_4_ of 10 phr (parts per 100 parts of (PLA + ENR)). The shape-memory property of all samples was confirmed and quantitatively evaluated by stress-controlled DMA at 70 °C (above *T*_g_ of PLA). In this sense, the analysis of the sample P7E3F1 showed that most of the fixed strain was recovered, indicating the excellent shape-memory behavior of PLA/ENR/Fe_3_O_4_ TPVs with *R*_f_ and *R*_r_ values of ∼100% and of ∼98%, respectively. Then, to visually observe its shape-memory behavior, a folded specimen was heated to 70 °C, finding a complete recovery to its permanent shape within 50 s (Figure 22A). As a ferromagnetic material, Fe_3_O_4_ could endow PLA/ENR/Fe_3_O_4_ TPV with excellent magnetically sensitive shape-memory effect because of its magnetocaloric effect under an alternating magnetic field (AMF).^28^ To test this, P7E3F1 was put into an AMF with a magnetic field strength of 29.7 kA/m and a frequency of 45 kHz, achieving the complete recovery of its initial shape within 6 s (Figure 22B). Additionally, the photothermal effect displayed by Fe_3_O_4_ made it possible to activate the shape-memory function under NIR light.^224^ Under NIR irradiation, the surface temperature of samples rapidly increased to 86 °C, triggering the shape recovery within 30 s (Figure 22C). As a comparison, P7E3F0 could not be heated by AMF/NIR and no shape recovery was observed, demonstrating the importance of including these nanostructures in the material. It is well-reported that polymer-based actuators having interpenetrating networks as inner structure can display self-healing function assisted by its shape-memory effect.^225−228^ Regarding the above, ENR with high epoxidation level had also exhibited self-healing ability because of its interdiffusion and self-adhesion.^229,230^ Therefore, PLA/ENR/Fe_3_O_4_ TPVs with continuous structure are also supposed to exhibit shape-memory assisted self-healing function. To achieve perfect shape recovery, the specimens were stretched with 50% strain at 70 °C and then damaged by a homemade blade-device. Stress–strain measurements were carried out for P7E3F1samples before the damage, just damaged and healed at different conditions. Tensile strength and strain at break values of 28 MPa and 57%, respectively, were calculated for the undamaged P7E3F1 sample, while just after the damaged both parameters decreased to 20 MPa and 4%. Then, after being healed at 70 °C for 3 h, P7E3F1 displayed a healing efficiency of 69% characterized by the partial recovery of its tensile strength and strain at break values (25 MPa and 39%, respectively). Surprisingly, as is shown in Figure 22D, damaged samples were also capable of healing after 10 min of exposition under AMF and NIR-light irradiation, reaching tensile strength values of 25 and 24 MPa, respectively. These remote/noncontact stimuli allow the healing of the sample with less energy, in shorter times, and avoiding degradation of the nondamaged regions. An obvious crack could be observed on the surface of damaged specimen. After heating at 70 °C for 3 h, the damaged surfaces of the prestretched specimens were forced to achieve physical contact, resulting in the disappearance of the crack. However, the local cracks could still be observed obviously in the unstretched specimens because the stored strain energy in the plastic zone is insufficient to achieve surface contact perfectly confirming that physical contact of the damaged surfaces, induced by shape-memory motions, was a precondition for a successful self-healing process. Experiments showed that the healing efficiency of the prestretched specimens was higher than the unstretched specimens, as the healing efficiency of the stretched P7E3F1 was 69%, whereas the unstretched P7E3F1 was only 54%. These results confirmed the key role played by the shape-memory function on the healing process. When TPVs were heated above *T*_g_, the shape recovery of the prestretched specimens drove the release of stored strain energy in the damaged zone, resulting in the closure of the crack. However, P9E1F1 and P8E2F1 exhibited relatively weak shape-memory effect with shape-recovery ratios of 80 and 84%, respectively, being insufficient to induce the surface contact between damaged regions. Thus, P9E1F1 and P8E2F1 showed inferior self-healing efficiencies, allowing to state that the self-healing capability of these TPVs was directly related to their rubber content. Similarly, the shape-memory effect and segment mobility were too weak to achieve the healing of neat PLA. Conversely, the low self-healing efficiencies of P6E4F1 and P5E5F1 were ascribed to their high cross-link density. As the cross-link density of rubber played a vital role in its self-healing effect, the authors added different amounts (0.5, 1, 1.5 phr) of DCP to samples, aiming to evaluate the effect on their self-healing performance. The specimens were cut into two sections and put close to each other to achieve spatial contact, then heated at 70 °C for 3 h. Healed samples did not fracture at the joint position even under strong twisting, bending, or stretching (Figure 22E). With increasing DCP content, the pure ENR showed a declining healing result due to its high cross-link density.^47^ As the authors demonstrated, P7E3F1 with lower DCP contents exhibited a remarkable healing effect in terms of tensile strength. In contrast, the increment of the DCP content could endow samples with higher tensile strength but sacrifice their self-healing capacity. For example, when the DCP content increased from 0 to 1.5 phr, the healing efficiency of the ENR/Fe_3_O_4_ vulcanizates dropped from 90 to 59%, respectively. The neat ENR and ENR/Fe_3_O_4_ compounds were masticated with same degree in a two-roll mill and then immersed in toluene to evaluate the effect of Fe_3_O_4_ in the self- healing behavior. Neat ENR was first swollen and then almost dissolved within 3 days. However, ENR/Fe_3_O_4_ specimen was just swollen after immersing in toluene for 3 days, suggesting that Fe_3_O_4_ particles can act as strong net-points to absorb ENR chains and form bound rubber to resist the dissolution of ENR.^231^ Meanwhile, it could be seen from the comparison between P7E3F0 and P7E3F1that a fraction of DCP could be adsorbed and shielded by Fe_3_O_4_, reducing the content of covalent cross-link density. Thereby, the incorporation of Fe_3_O_4_ could, simultaneously, form bound rubber and decline covalent cross-link density; thus, PLA/ENR/Fe3O4 TPVs showed better self-healing efficiency than PLA/ENR TPVs. Fe_3_O_4_-containing vulcanizates with low cross-link density exhibited strong peel strength, which was ascribed to interdiffusion of ENR chains and could result in a better self-healing efficiency. In this sense, the corresponding P7E3F1-0.5DCP achieved a high healing efficiency of 83% considered as an outstanding result for TPV-based materials. An interesting result was observed by authors after demonstrating the better healing efficiency of P7E3F1-0.5DCP against P7E3F1-0DCP. They attributed this result to the possible migration of Fe_3_O_4_ entities into PLA phases, dragging with them ENR chains chemically attached to their surfaces by DCP-induced grafting processes. The above would open the possibility of inducing physical interlacement and interlock between chains of both polymers enhancing the healing process. However, at higher amounts of DCP, it would be promoted a higher cross-linking degree within the polymer network reducing the mobility of chains, affecting the healing function. The mechanism of the self-healing was explained as follows: (1) When the TPVs were heated above *T*_g_, the deformation caused by the damage would recover to its initial shape driving by its shape-memory property, allowing the physical contact between both damaged surfaces. (2) Then, the interdiffusion of ENR chains triggered at high temperature would promote the self-adhesion of ENR phases, also supported by the desorption–absorption effect of the bound rubber. (3) Additionally, due to the incorporation of DCP, PLA chains were covalently added to ENR chains and entangled with ENR driven by the migrated Fe_3_O_4_. Thereby, PLA structures present at the damaged join surfaces are able to rearrange and re-entangle with ENR chains, resulting in a complete closure of the cracked interface.

**Figure 23:**
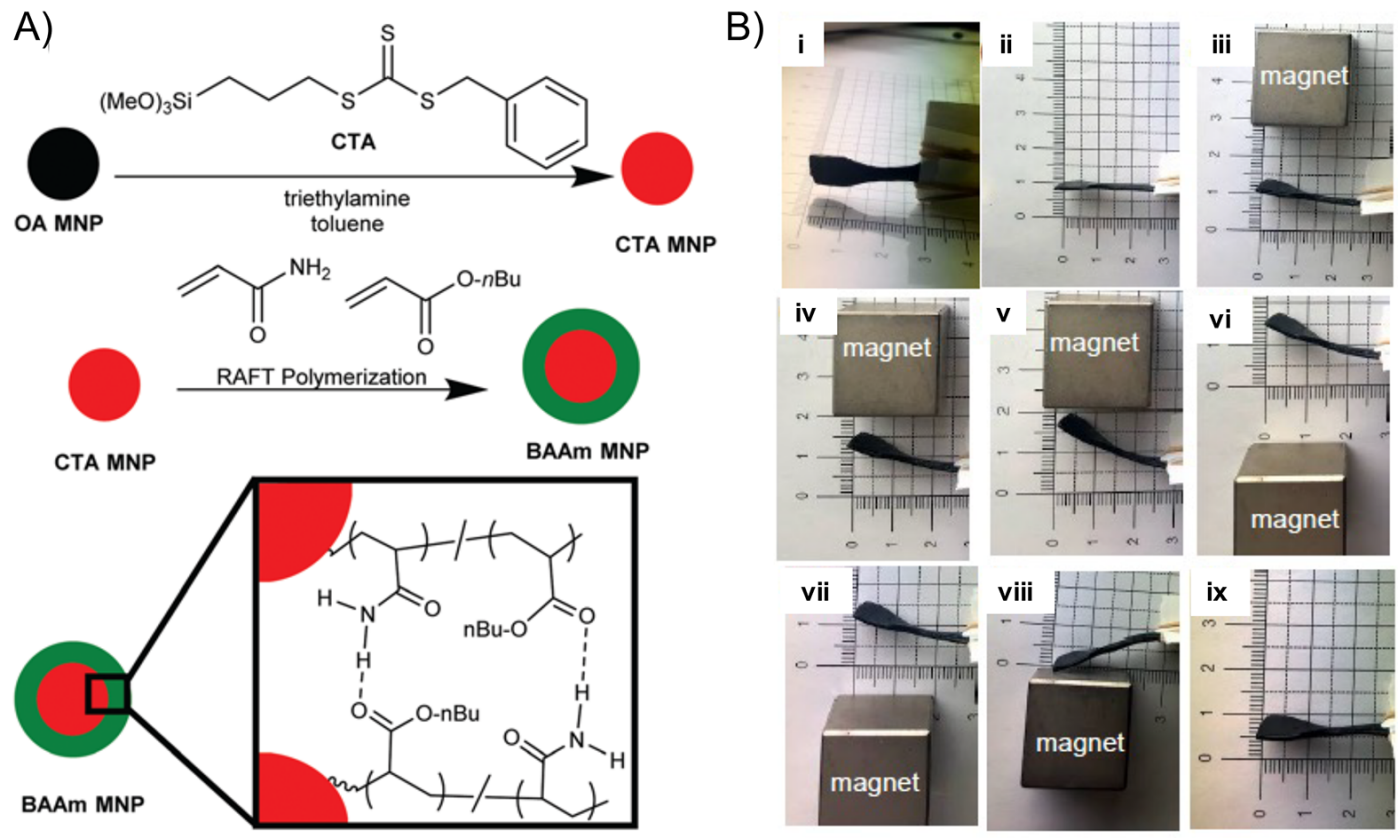
(A) Design and synthesis of magnetic nanoparticles grafted with acrylamide (Am) and *n*-butyl acrylate (BA) copolymers. (top) Functionalization of oleic acid (OA) MNPs with a chain-transfer agent (CTA) to yield CTA MNPs. (center) Synthesis of BAAm copolymer functionalized MNPs. (bottom) Hydrogen bonding between Am and BA are shown as representative examples. (B) Digital photographs showing the actuation of BAAm-MNP-75 sample using 2.54 cm^3^ neodymium magnet (54 kg pull force). Grid in background is for measuring distance. A dog-bone sample was affixed in position in (i) a parallel view and (ii) perpendicular view. (iii) Magnet was placed on the 3 cm mark and (iv) moved to the 2 cm mark where actuation starts and (v) finishes in less than 30 s. (vi) Magnet was placed on the opposite side and (vii) moved closer by 0.5 cm (viii), then by 1 cm where actuation occurred. (ix) Finally, the magnet was removed from area and the sample resumed to its original position. Adapted and modified with permission from ref (232). Copyright 2020 Royal Society of Chemistry.

In 2020, Guan and co-workers reported the fabrication and characterization of self-healing magnetic nanocomposites prepared from commercially available monomers and Fe_3_O_4_ magnetic nanostructures.^232^ These multifunctional systems displayed Young’s modulus around 70 MPa and over 500% extensibility, allowing consideration as robust materials with high mechanical strength. The obtained nanocomposites also stood out by their self-healing function, reaching values of healing efficiency of 46%, referred to the recovery of extensibility. Importantly, after the healing process, samples retained their magnetic actuation property. Experimentally, Fe_3_O_4_-based magnetic nanoparticles (MNPs) with a narrow size distribution (21.5 ± 1.9 nm) were synthesized and superficially functionalized with a radical chain-transfer agent (CTA), allowing the copolymerization of acrylamide (Am) and *n*-butyl acrylate (BA) monomers from MNPs surfaces through the RAFT process. Thereby, by using the graft-from approach, they were able to achieve high weight percent and homogeneous dispersion of MNPs inside polymer matrices.^233^ As indicated above, the authors employed an inexpensive commodity monomer, Am, characterized by its ability to participate in the formation of dynamic and reversible hydrogen bonds, endowing the material with a self-healing function (Figure 23A). Moreover, the copolymerization of Am with BA units was carried out, aiming achievement of a macromolecular system with enhanced polymer chain dynamics, favoring the occurrence of a spontaneous healing process under ambient conditions. A range of nanocomposites with different content of polymer were prepared, referred to as BAAm-MNP-XX, with XX being related to the amount of polymer estimated from TGA measurements. Therefore, BAAm-MNP-XX (XX = 75, 81, 85, and 86) nanocomposites were successfully prepared and characterized. Homogeneous nanoparticles distributions were corroborated by TEM analysis, attributed to the graft-from approach used during the fabrication of samples, allowing to overcome typical phase separation issues usually observed between materials with no or low. Self-healing capabilities were tested by first inducing a cut on the sample, followed by bringing the interfaces of damaged parts back into contact, and finally allowing healing for the desired duration. Then, the process was evaluated, quantitatively, by calculating the self-healing efficiencies by uniaxial mechanical testing and comparing the results to the ones obtained for original samples. Results shown that, at the same temperature, those materials having higher polymer content achieved, in general, higher healing efficiencies in shorter times, assigning the healing function of these materials to the polymer portion. In this sense, while BAAm-MNP-85 sample recovered 41% of the extensibility after being healed at ambient conditions (30 °C) for 2 h, to achieve similar values with BAAm-MNP-33 it was needed to extend the healing process up to 5 h, after which a 46% of its extensibility was recovered. Because the healing process turns out to be dependent on the polymer content of materials, the authors argued that the healing mechanism would be related to hydrogen-bonding reformation between polymer chains present at the material’s interface across the damage. Finally, the magnetic-actuation function was successfully tested by exposing the samples to a neodymium magnet with a 54 kg pull force (Figure 23B). One of the ends of dog-bone-shaped samples was fixed, while the magnet was approached to the other free end. The actuation motions start to be present when the magnet was 2 cm away. Samples revealed a versatile actuation function, achieving diverse orientation by changing the position of the magnet. Then, when the magnet was retired, samples recovered their initial orientations. This experiment showed that the strategy presented in this work allowed the obtainment of self-healable polymer-based nanocomposites displaying rapid and reversible magnetic-induced actuation motions.

**Figure 24:**
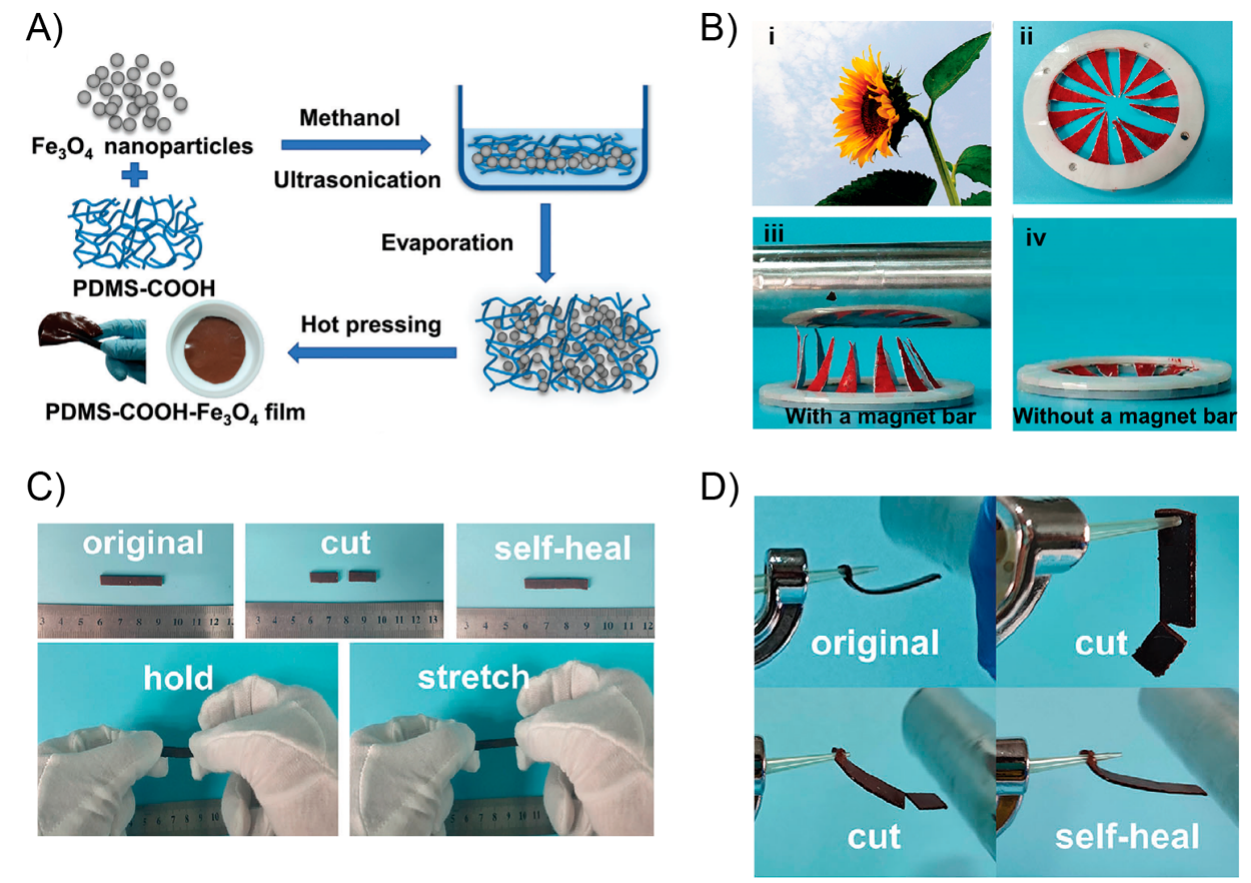
(A) The fabrication process of PDMS-COOH-Fe_3_O_4_ sample. Schematic diagram of the structure of PDMS-COOH and the preparation of PDMS-COOH-Fe_3_O_4_. (B) (i) the photograph of the sunflowers; (ii) the photograph of a bionic sunflower made by the composite film; (iii,iv) the actuation of the bionic sunflower with or without a magnet bar. (C) Digital photos showing a complete self-healing process of this film under an ambient condition. (D) self-healing display of the polymer film (30 × 6 × 0.9 mm^3^) under an ambient condition during the magnetic actuation. Adapted with permission from ref (234). Copyright 2022 John Wiley and Sons.

Very recently, Li and co-workers reported about a room-temperature self-healing magnetic nanocomposite, which was obtained using a simple, efficient, and environmentally friendly strategy.^234^ The strategy designed by the authors consisted in the dispersion of Fe_3_O_4_ nanoparticles into a poly(dimethylsiloxane) soft matrix chemically modified with COOH functional groups (PDMS-COOH). This PDMS derivate was obtained by following previous reported protocols.^235,236^ Fe_3_O_4_ nanoparticles were incorporated, aiming to endow the material with a magnetic-driven actuation function. According to authors, after carrying out a deep optimization process, the optimal content of magnetic nanofiller was determined to be 15 wt %.^237^ On the other hand, the system managed to show an excellent self-healing efficiency (62.2% based on mechanical recovery of fracture strength) at 25 °C for 30 min. Furthermore, as was expected, this healable nanocomposite stood out by showing an excellent and healable magnetic actuation property. The synthetic protocol for the materiaĺs preparation started by achieving a proper dispersion of Fe_3_O_4_ nanoparticles, through mixing and ultrasonication, in a methanolic PDMS dissolution. The obtained dispersion was then poured into polytetrafluoroethylene (PTFE) molds and aerated at room temperature for 24 h and then heated at 120 °C in an oven for 12 h. Finally, the PDMS-COOH-Fe_3_O_4_ sample film was achieved by hot-pressing (Figure 24A). The SEM analysis performed for PDMS-COOH-Fe_3_O_4_ (15%) revealed no apparent particles aggregation, whereas its thermal characterization (by DSC and TGA) showed a *T*_g_ value of 0.5 °C and a thermal decomposition process starting at 220 °C, allowing it to be considered as a thermally stable material for a wide spectrum of applications. On the other hand, the obtained showed adequate mechanical properties based on the obtained values for tensile strength and tensile strain of 0.44 MPa and 400%, respectively. Prior to the study of the actuation function, the magnetic property of PDMS-COOH-Fe_3_O_4_ (15%) was analyzed. This sample, compared to pure Fe_3_O_4_ nanoparticles, showed a decrease of the magnetic property, due to the nonmagnetic nature of the polymer matrix. In addition, the material exhibited a superparamagnetic behavior. Then, a sample strip was actuated toward different orientation in the presence of a magnet by changing the distance and position of a magnet. Additionally, trying to replicate nature-based movements the authors prepared a flower-shaped material using PDMS-COOH-Fe_3_O_4_ (15%) successfully achieving opening and closing motions in the presence and absence of a magnetic bar, respectively (Figure 24B). On the other hand, the authors tested the self-healing property of this material by cutting a film into two pieces using a blade. Both pieces were put in close contact under ambient conditions to complete the healing process. Surprisingly, after 2 min, the healed sample was able to be stretched again without showing visible damage (Figure 24C). In addition, by comparing the tensile stress–strain curves between the original and healed samples, the healing efficiency parameter was calculated. These experiments demonstrate that at higher times, more efficient was the healing process. In this sense, healed samples were stretched with 62.2%, 80%, and 97.7% self-healing efficiencies after being healed for 30 min, 1 h, and 8 h, respectively. Finally, the authors designed an experiment in which they successfully demonstrated the self-healing capacity of the material during the magnetic actuation process (Figure 24D).

### 2.3 Self-Healing Actuators Combining Light and Magnetic Stimuli

**Figure 25:**
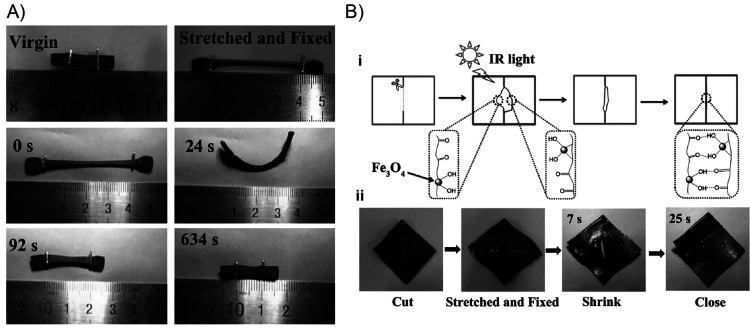
(A) Shape-memory effect of MD_50_-F_5_ elastomers. (B) Illustration (i) and actual images (ii) of shape-memory assisted self-healing process. Adapted with permission from ref (238). Copyright 2022 John Wiley and Sons.

In 2016, Li and co-workers reported a shape-memory magnetic elastomer with high mechanical strength consisting of a conventional polymer and Fe_3_O_4_ nanoparticles.^238^ The elastomeric nanocomposite exhibited superparamagnetism and superior self-healing performance at elevated temperatures. The tensile strength of the cured elastomer can be up to 12.0 MPa and almost equal to that of the original sample. These materials can be remotely controlled to enable their shape-memory function and actuation behavior. They can be used in many applications, from wireless controllers to actuators, biomedical devices, and other remote memory systems. Magnetic elastomer was synthesized from 2-methoxyethyl acrylate (MEA), *N*,*N*-dimethylacrylamide (DMAA), Fe_3_O_4_, and trace amounts of *N*,*N*,*N*′,*N*′-tetramethyldiamine (TEMED) as a catalyst. The sample codes for the obtained nanocomposites were defined as MD_*x*_-F_*n*_, *x* referring to the molar fraction of DMAA and *n* the mass fraction of Fe_3_O_4_ nanoparticles respectively. To illustrate the shape-memory effect, DMA scans of cross-sectional samples were performed, revealing that while the *T*_g_ of neat MD_50_ was 22.2 °C, the *T*_g_ measured for MD50-F5 was decreased to 18.7 °C,^239^ which could be attributed to a hindering of the cross-linking process through which the polymeric network is built, due to the presence of nanoparticles during in situ polymerization. By varying the molar ratio of DMAA, the *T*_g_ values of the elastomers were reduced from 64.2 °C (MD70-F5) to 0.5 °C (MD30-F5). The results indicated that the *T*_g_ of the magnetic elastomers could be easily adjusted by changing the proportion of monomers in the formulations. Additionally, tan δ curves recorded for MD50-F5 displayed a sharp peak at 42.3 °C, which would imply that the MD50-F5 could exhibit an excellent memory performance.^240^ Typical DMA experiments were performed to assess the shape-memory function of these materials quantitatively. First, MD50-F5 elastomer was stretched to 300% at 50 °C and then cooled to room temperature. After 30 min, the sample was retired from the clamps, and the fixing strain was obtained. Surprisingly, the result showed that the shape fixing ratio of the elastomers was almost 100%. Furthermore, when re-exposed to an IR lamp (220 W), the shape rapidly recovered up to 50% in 92 s and almost completely recovered (97%) in 634 s (Figure 25A). The authors designed a simple experiment to confirm the shape-memory-assisted self-healing performance (Figure 25B). The sample was cut in the center, then stretched at 50 °C, and the strain was fixed by cooling the damaged sample at room temperature. Later, when the above sample was exposed to IR light, the crack shrank rapidly inducing the contact between the fractured surfaces. It should be noted that the fissure was absolutely closed within 30 s. In addition, by prolonging the healing time, the samples could be fully restored. The fast rate of crack closure ensured that the fracture surfaces in contact were “fresh”, promoting better healing efficiency. From a mechanistic point of view, there should be an abundant presence of multiple hydrogen bonds between the magnetic nanoparticles and the functional groups present in the polymer chains.^241,242^ Moreover, as authors argued, under the actions of entropic interactions between the particles and the polymer chains, the nanoparticles would be able to migrate to the fracture surfaces. Thus, allowing the reformation of the hydrogen-bonding network when the fracture surfaces get in contact. It should be expected that the diffusion of nanoparticles within the polymer toward the damaged surface improved the density of hydrogen bonds and enhanced the self-healing performance.

**Figure 26:**
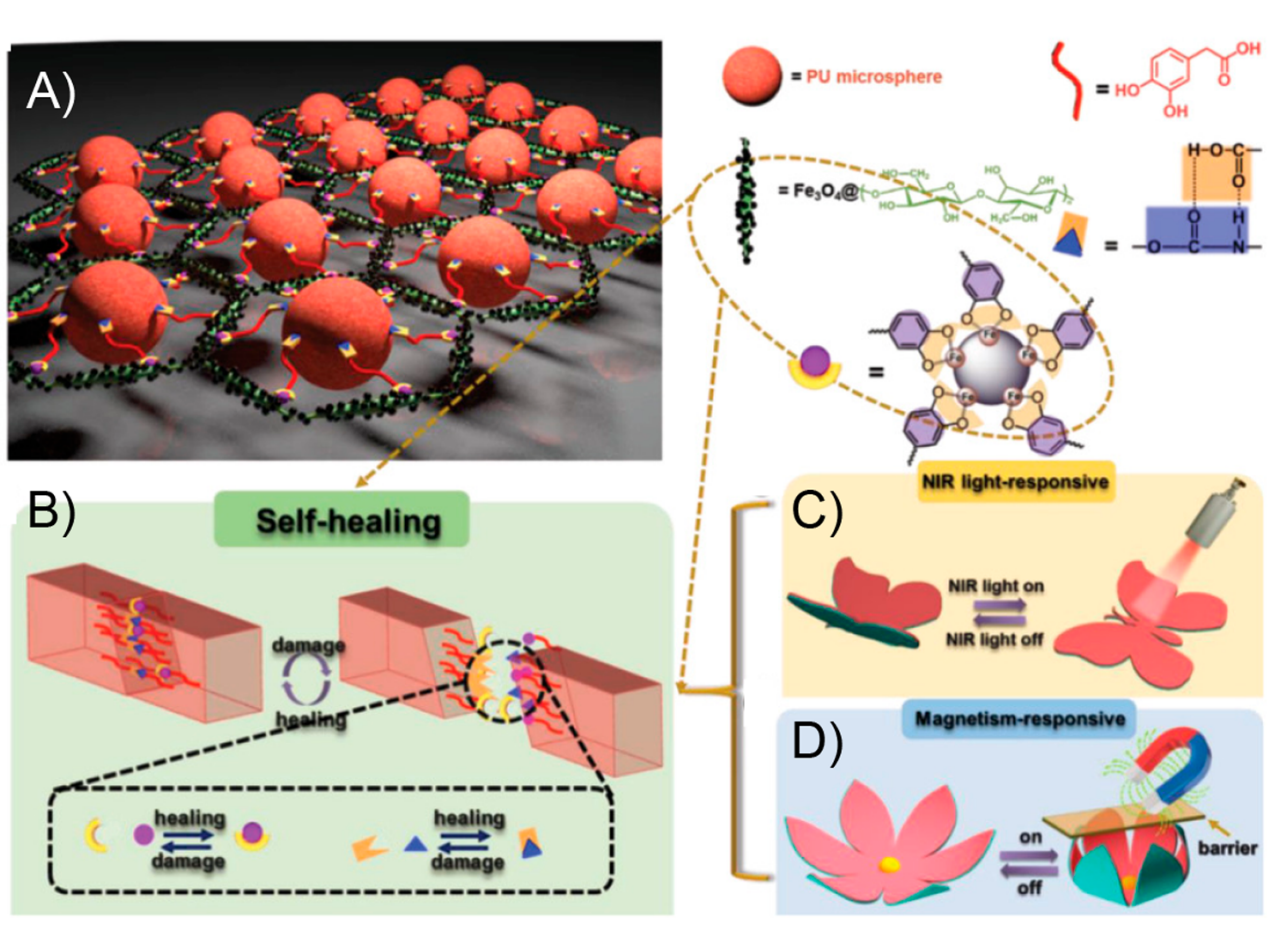
Design of the hierarchically structured supramolecular elastomers. (A) Schematic illustration for hierarchical structural design and interfacial supramolecular cross-linking in PU matrix. (B) The metal coordination cross-linking network design of self-healable supramolecular elastomer. Schematic diagrams of NIR light (C) and magnetic (D) responsive actuating. Adapted with permission from ref (243). Copyright 2019 John Wiley and Sons.

By the end of 2019, Wang and co-workers proposed a novel strategy for the preparation of a multistimuli responsive self-healable soft material achieving unprecedent photothermal conversion and excellent self-healing function (92.2%), allowing consideration as a new benchmark material in the field of soft robotics and with great applicability in the design of new biomimetic actuators.^243^ The strategy consisted in the superficially decoration of cellulose nanocrystals with Fe_3_O_4_ magnetic nanoparticles. This nanohybrid system exhibited excellent water-dispersion facilitating its inclusion into a dispersion of a waterborne PU, promoting the construction of a 3D interconnected network in polymer matrix via the latex self-assembly method.^244−246^ Then, by the addition of 3,4-dihydroxyphenylacetic acid (DOPAC), an interfacial supramolecular structure cross-linked through metal–ligand interactions between Fe_3_O_4_ entities and catechol groups of DOPAC was formed within the PU matrix (Figure 26A). This material, due to the incorporation of Fe_3_O_4_ nanostructures, was endowed with a magnetic-driven actuation function (Figure 26D). Moreover, due to the well-known photothermal property of these nanostructures and their inclusion into a well-ordered and stable 3D-supramolecular structure, the material set a record in terms of photothermal efficiency (79.1%), showing also excellent thermal conductivity (1.92 W/(m K)).^247−250^ This has been attributed to a better and more efficient heat transmission throughout the sample, precisely due to the high molecular order achieved by the supramolecular assembly. In fact, the above properties allow understanding of the good actuation property exhibited by the material under NIR irradiation (Figure 26C), where a strip-shaped sample reached its maximum bend state after only 0.44 s. In this sense, after the Fe_3_O_4_-mediated light-to-heat conversion process, rapid transmission of this heat would take place throughout the sample, allowing its temperature to increase remarkably quickly, triggering the actuation motions. The authors demonstrate the direct relation existing between the light intensity and the actuation function, achieving larges forces under higher light intensities (4.5 × 10^–4^, 7.7 × 10^–4^, and 1.0 × 10^–3^ N for intensities of 0.6, 0.8, and 1.2 W, respectively). In addition, the light-actuation function showed reliability because during 5 consecutive actuation cycles, the motions of the material did not show damping phenomena. On the other hand, this material also exhibited an effective response to magnetic stimuli, achieving, in about 0.36 s, an outstanding magnetic-driven actuation function. Aiming to contribute to the soft robotic field, the authors reshaped this material into different types of configurations, ranging from simple bent samples to more complex ones such as butterflies, hands, and even a small-scale “athlete” that replicated pull-up movements. All of these configurations were able to conduct, in a fast and efficient fashion, reversible multistimuli-induced actuation motions. This material also stood out by its ability to self-repair under ambient conditions and in short times (around 15 s) by just bringing into contact damaged portions. The excellent self-healing function was supported by the high healing efficiency value (92.2%) calculated from stress–strain experiments. In addition, after 20 cycles of consecutive damage/healing, this value remained as high as 79.7%. Outstandingly, the authors demonstrated the versatility and robustness of the healing capability by successfully conducting the process under harsh conditions (e.g., in water and at subzero temperatures). The healing mechanism proposed by authors would be mostly based on the DOPAC-Fe_3_O_4_ metal–ligand interactions arisen from the formation of iron-catechol complexes but also supported by multiple hydrogen-bonding interactions (Figure 26B). The authors state that this work could be considered as the first report about the design of a hierarchical structured self-healing actuator exhibiting high mechanical strength, repeatable and excellent self-healing property, and a fast and multiresponsive actuation motions under ambient conditions and complicated surroundings.

### 2.4 Self-Healing Thermal-Actuators

**Figure 27:**
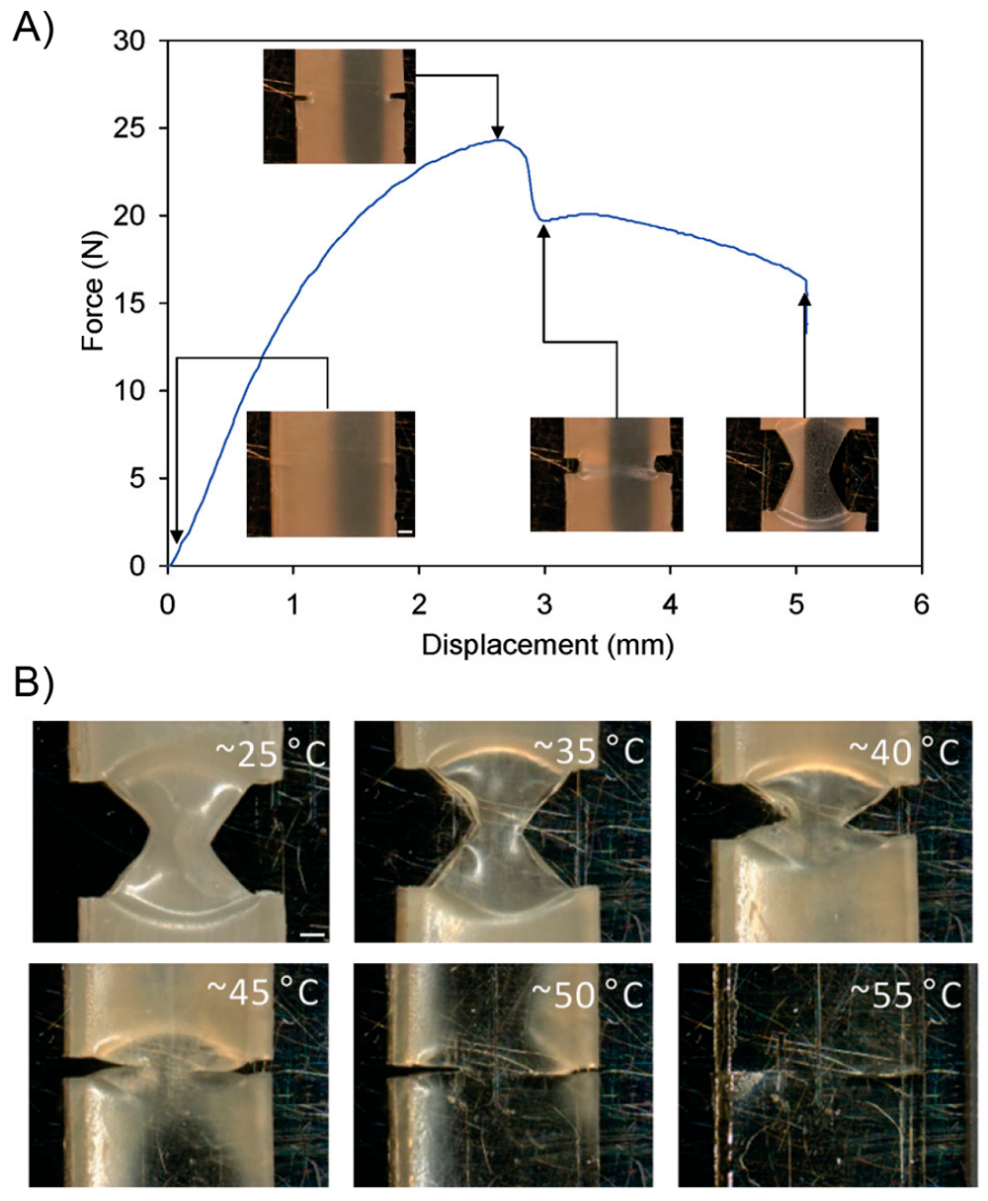
(A) Force vs displacement curve of a notched sample (i-PCL50:n-PCL50) showing micrographs of deformation and crack growth clamped in the Linkam tensite stage. (B) Snapshots of crack closure and crack rebonding when the sample was unclamped from the Linkam tensile stage and heated to the temperatures shown above (scale bar = 500 μm). Adapted with permission from ref (225). Copyright 2011 American Chemical Society.

In 2011, Erika and co-workers reported the preparation of a novel material showing simultaneously shape-memory function and self-healing capability.^225^ This system was achieved by conducting the chemical reticulation of a PCL diacrylate derivative (n-PCL) via UV-triggered thiol–ene polymerization using a tetrathiol as cross-linking agent.^229^ In addition, to the previous formulation, unmodified PCL (i-PCL) was added in order to provide the final material with self-healing properties, taking advantage of its well-known thermoplastic behavior.^251^ In this sense, n-PCL would be responsible for the structural stability of the thermoset, while i-PCL would be in charge of the healing process. Additionally, the authors afforded the preparation of different samples by varying the proportions between n-PCL and i-PCL. All tested samples showed an excellent shape-memory function based on the reversible plasticity shape-memory (RPSM) phenomenon typically observed in this type of semicrystalline polymer systems.^252^ Samples were deformed with high levels of strain into a temporal shape below the critical temperature of the system (melting temperature, *T*_m_). Then, when the material is heated above this temperature (≈55 °C), the system is allowed to release the stored stress energy triggering the movements that will lead the recovery of its initial shape. The shape-memory property of the different samples was evaluated in terms of the *R*_f_ and the *R*_r_ values showing to be highly dependent on the n-PCL/i-PCL proportion. In this regard, samples showed *R*_f_ values in the range 74.1–80.8%, while the calculated values for *R*_r_ were between 69.1 and 92.7%, indicating an opposite behavior for *R*_f_ and *R*_r_ as the content of n-PCL increases and, therefore, a direct relation with the cross-linking density of the material. As mentioned before, the obtained material was able to self-repair under thermal stimuli by achieving the closure of a crack generated after induced mechanical damage (Figure 27A,B). Damaged samples were healed by heating at 80 °C and holding this temperature for 10 min, achieving the complete closure of the crack. Then, the healed samples were subjected to tensile testing experiments to evaluate their healing efficiencies. If the sample under evaluation was not fractured during the test, it was healed in a second cycle and tested again. This was carried out up to three cycles. Samples with compositions greater than 25 wt % of i-PCL achieved a complete healing process (crack closure), showing healing efficiency values greater than 90% after the first healing cycle, and maintaining this performance during the following two cycles. Moreover, samples with higher content of i-PCL displayed higher healing efficiency. Conversely, samples prepared with 20 wt % i-PCL or lower were unable to complete the healing process after the first cycle. Authors explain the healing mechanism based on five consecutive processes. In a first step, at temperatures above *T*_m_, polymer chains in the surfaces of the crack start to rearrange (1) and, consequently, the surfaces start to approach each other (2). In a third stage, called “surface wetting” (3), they explain that after approaching, the surfaces along the fissure come into contact, allowing starting of the interchain diffusion process (4) seeking to reach a state of equilibrium where the diffusion process is completed. Once this state is achieved, the randomization and re-entanglement (5) of i-PCL chains leads to the strengthening of the network, which is finally fixed after cooling down the system to room temperature. One of the limitations of this work mentioned by the authors was the low rubber modulus displayed by these materials when heated above *T*_m_, needing a better mechanical support for practical applications.

**Figure 28:**
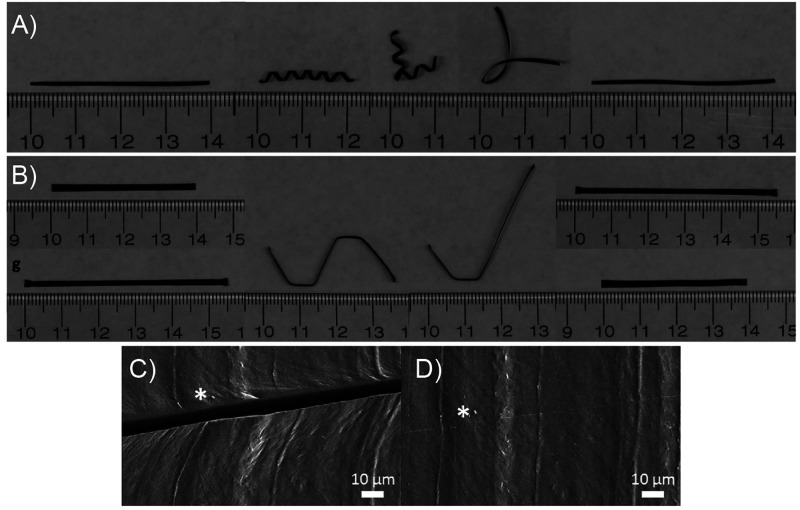
Digital photographs of the first (A) and second (B) multiple-shape-memory cycles performed on a SWNTs/Nafion_H+_ nanocomposite strip. SEM images of a film damaged with razor cuts (C) and after the healing process (D). Adapted with permission from ref (255). Copyright 2012 American Chemical Society.

A year after, based on the multishape-memory effect, understood as the capability of memorizing three or more temporally shapes in one cycle,^251,253,254^ Kohlmeyer and its co-workers studied a polymeric nanocomposite prepared by dispersing single-walled carbon nanotubes (SWNTs) within a Nafion matrix (SWNTs/Nafion_H+_). The as-prepared system stood out by its outstanding and versatile shape-memory property and by being able to self-repair.^255^ The authors settled on an optimum concentration of 0.5 wt % of SWNTs in nanocomposites, which were prepared by dispersing an amide functionalized SWNTs into an alcoholic solution of Nafion (5% w/w). Films were obtained by the *solvent-casting* technique using PTFE dishes. The outstanding shape-memory property of this material was evaluated by performing consecutive multiple-shape cycles using a flat strip as starting material. In a first cycle (Figure 28A), the first step consisted in the transition from a flat shape to a coiled configuration via IR laser irradiation (808 nm, 6 mW/mm^2^), which allow reaching of temperatures around 70–75 °C and fixing this new temporary shape by cooling down to room temperature. Then, the coiled sample was bent locally again but using a more powerful IR source (808 nm, 25 mW/mm^2^), allowing reaching of temperatures as high as 150 °C followed by cooling. The third temporary shape was achieved by maintaining the bent configuration but removing the coiled structure. This was achieved after heating the sample at 75 °C in an oven. Finally, the initial flat configuration was recovered by removing the localized bend via heating at 140–150 °C, again, with an IR source (808 nm, 25 mW/mm^2^). With this new flat configuration, authors performed a second multiple-shape cycle (Figure 28B) by stretching the sample at 100 °C. The stretched temporal shape was subjected to a bending process by heating locally with IR laser (140–150 °C) four different portions of the sample, which was fixed by cooling again at room temperature. Then, each folded portion were removed by irradiating selectively and independently different zones of the sample with IR light (140–150 °C), achieving a stretched flat sample. Finally, by heating at 120 °C with an IR lamp, the stretched flat sample recovered its initial configuration. With this complex experiment, the authors demonstrated the versatility and robustness of the material, revealing an outstanding actuation function. Additionally, the self-healing capability displayed by the system was successfully corroborated after observing the disappearance of razor cuts intentionally exerted on the material surface after being heating at 140–150 °C by IR irradiation (Figure 28C,D). Based on the authors explanation, a possible mechanism for the observed healing process could be related to a synergic effect created between hydrogen bonding interactions and the diffusion/re-entanglement of polymer chains. Both processes would be activated by the abrupt temperature increase during light irradiation. In this sense, the increased mobility of polymer chains at higher temperatures plus the reversible nature of hydrogen bonds coming from the acid groups of the polymer matrix would generate a propitious environment to inducing the self-healing process, showing healing efficiencies of about 100%. However, the authors were clear about the limitation of the healing process developed in this work when working with “deprotonate” Nafion (Nafion_Na+_), which is usually the most employed in applications.^256−259^ According to their experiments, by IR irradiation or direct heating, this polyelectrolyte could not mend the exerted damage. The authors argued that this issue could be related to a severe increase in the chain mobility restriction when this polymer adopts a charged state.

**Figure 29:**
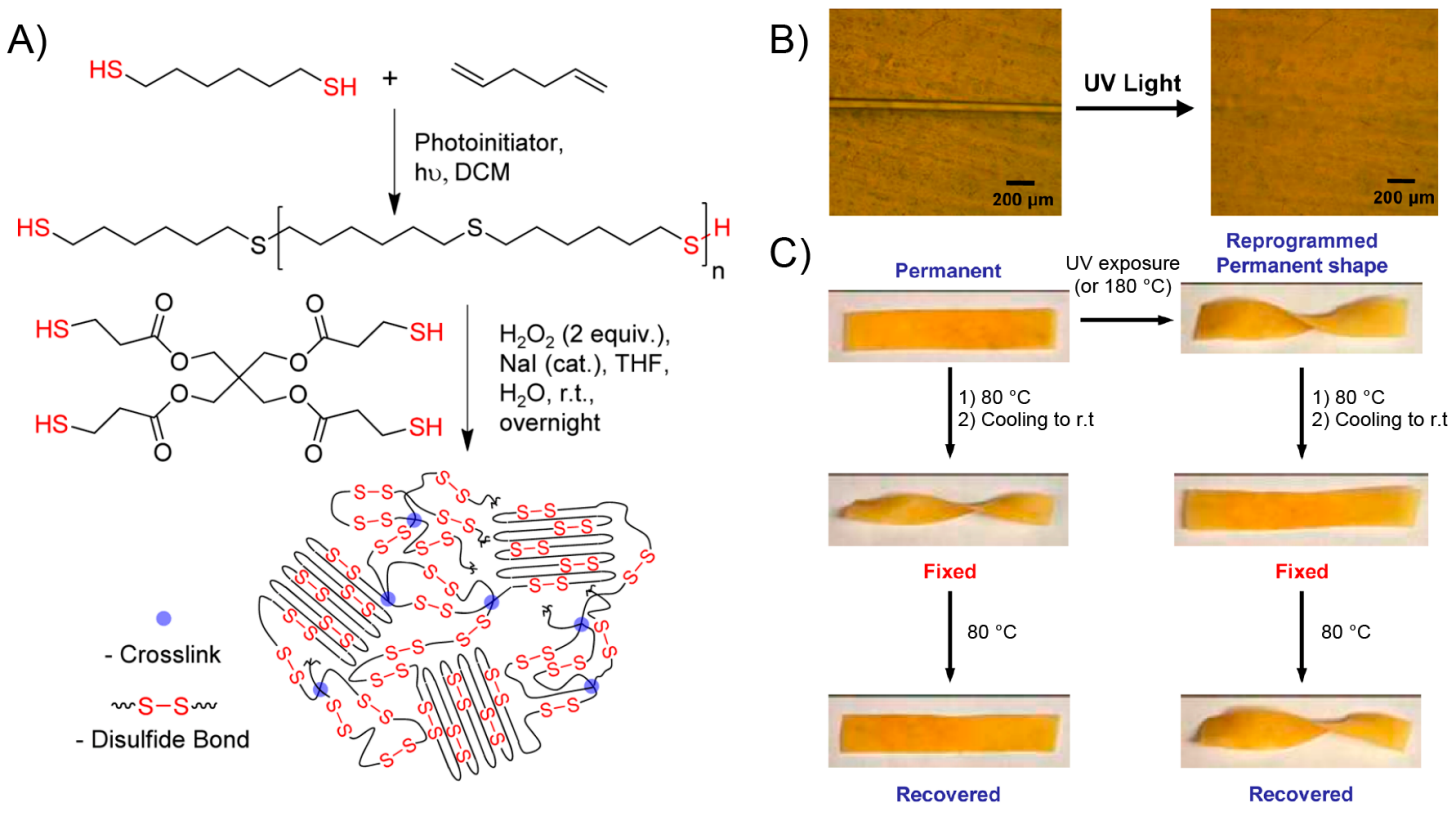
(A) Scheme of the synthetic route employed to fabricate the polydisulfide network. (B) Image showing healing of a scratched film under UV light exposure. (C) Pictures showing the reprogrammable shape-memory properties of semicrystalline polydisulfide network film. Adapted with permission from ref (260). Copyright 2013 American Chemical Society.

In 2013, Michal et al. propose a material which combines shape-memory and self-healing capabilities based on the preparation of a semicrystalline polydisulfide network.^260^ The shape-memory effect was triggered by heating the sample while the self-healing function was achieved under UV light irradiation. Inspired by the chemistry and reversibility of disulfide linkages,^261−263^ the authors decided to fabricate a polymer-based soft actuator with self-healable function. To achieve this, using 1,6-hexanedithiol and 1,5-hexadiene as monomer entities, they conducted the preparation of a bisthiol oligomer via photoinduced thiol–ene reaction. Importantly, they were able to manage to some extent the molecular weight of the obtained system by controlling the ratio between both reactants. Then, in a second step, the oxidative coupling between thiols moieties coming from the presynthesized oligomer and pentaerythritol tetrakis(3-mercaptopropionate) (cross-linking agent) was successfully promoted, achieving the fabrication of a semicrystalline, covalently cross-linked system (Figure 29A). Moreover, by varying the [oligomer]/[tetrathiol] ratio, the authors prepared a set of reticulated systems having different cross-linked densities. All the samples showed melting phenomena with *T*_m_ values in the range of 57 and 61 °C. In addition, by DMA analysis, the *T*_g_ of these systems was determined around −30 °C. Then, the cross-linked samples were disposed in film format to pursue the evaluation of their properties. The self-healing performance showed by these materials was evaluated by scratching the surface of a film with a razor blade. The depth of the razos cut was approximately 150 μm. After that, the film was exposed for 5 min to UV irradiation (320–390 nm, 2000 mW/cm^2^). In order to avoid the effect of the photothermal phenomenom during the healing process, the authors placed the film sample between an aluminum block and a glass slide, both acting as heating sinks. However, the visualization of samples under IR camera, showed that after 5 min of irradiation samples reached temperatures around 77 °C, which is slightly above the *T*_m_ of samples. Notwithstanding the above, after the irradiation process, the scratches were nearly detected under optical microscopy (Figure 29B). In addition, stress–strain tests were performed to calculate the healing efficiency of materials. Results showed that, without being exposed to UV light, scratched films broke easily under tensile testing. Conversely, after being irradiated, healed samples displayed a similar mechanical response than original films. All samples exhibited healing efficiencies above 98%, allowing them to be considered materials with an excellent self-healing property attributed to the dynamic rupture and reformation of disulfide bonds across the damage region, collaterally helped by the diffusion and re-entanglement of polymer chains in melted domains. On the other hand, the shape-memory function was evaluated under thermal stimuli. Two types of experiments were designed by the authors. The first one was carried out by first heating a flat strip of sample at 80 °C (well-above *T*_m_) and deforming it into a spiral configuration, then being cooled, maintaining the stress to fix the new temporary shape. When this sample was heated again at 80 °C the initial flat shape was completely recovered. The authors designed a second experiment after noting that when a flat sample was twisted under UV light, the material adopted this new configuration as permanent shape. This reprogramed permanent shaping process was also achieved by heating the system at 180 °C for prolonged times. Then, with a twisted initial configuration, the sample was reshaped into a flat conformation following the same heating/cooling protocol explained above. Thus, after being heated again at 80 °C, the flat sample recovered its initial spiral configuration (Figure 29C). Finally, the authors also evaluated the synergy existing between the thermal shape-memory function and the photohealing process to heal more severe type of damage, as for example, scratching a prestretched sample. They demonstrated that after heating the sample at 80 °C a scar was clearly detected and was erased only after the irradiation with UV light.

**Figure 30:**
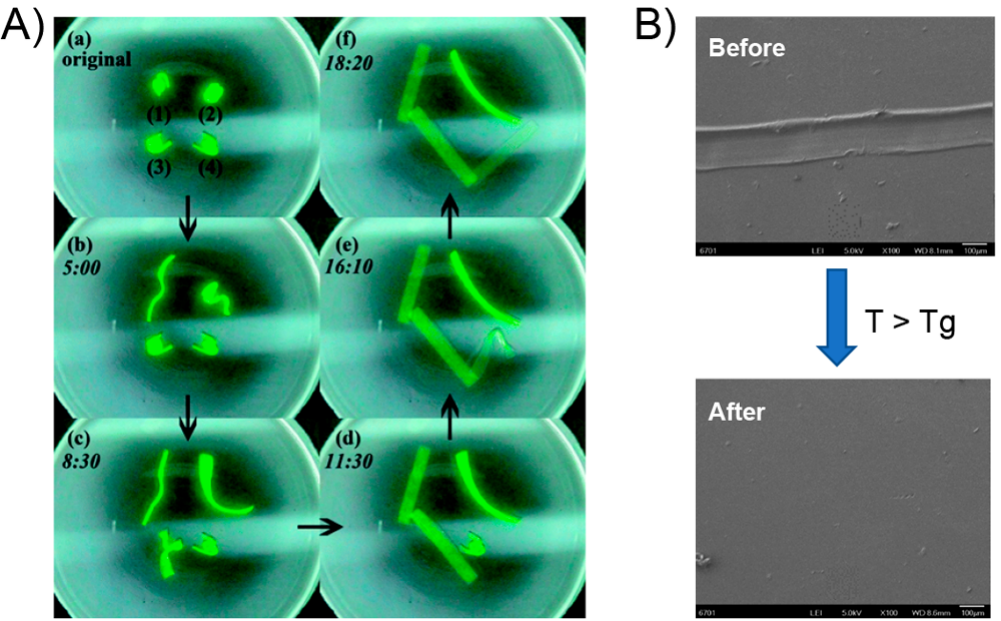
(A) Shape recovery of PVB-HDI samples in ethyl acetate. (B) SEM micrographs of scratched PVB-HDI-0.2 sample before (up) and after (bottom) the thermally driven healing process. Adapted with permission from ref (264). Copyright 2014 Royal Society of Chemistry.

In early 2014, Bai et al. carried out the preparation of a polymer gel by reacting poly(vinyl butyral) (PVB) with hexamethylene diisocyanate (HDI). The obtained material, showing a high cross-linking degree, stood out by presenting excellent shape-memory property and adequate self-healing efficiency.^264^ The synthesis of this macromolecular system was carried out in solution by conducting the cross-linking of PVB with HDI at 70 °C for 24 h. Samples were labeled as PVB-HDI-*n*, *n* being the amount (weight) of HDI using in the reaction. Based on the above, four samples named PVB-HDI-0.05, PVB-HDI-0.1, PVB-HDI-0.2, and PVB-HDI-0.3 were prepared and studied. As a first step in the characterization of samples, the authors evaluated the effect of HDI on the swelling property of the material as well as the gel content values. As expected, by increasing the amount of HDI employed in the reaction, the degree of swelling decrease from 1951 to 487% while the gel content increased from from 66.7 to 99.9%, demonstrating an enhanced cross-linking density in the material. Authors afford a good description of the mechanical and thermal properties of the obtained systems, mentioning that even when the system becomes brittle at higher cross-linking densities and exhibits low elongation at break values at room temperature, all samples could be elongated by more than 200% at 100 °C. Based on the above result, the authors designed the method to evaluate the shape-memory function of these materials by means of DMA. Samples were first heated at 100 °C and deformed into a stretch configuration which was fixed by rapidly cooling to room temperature. Then, the external force was removed and the shape recovery property was evaluated by reheating the sample at 100 °C. All samples showed excellent results where, for example, PVB-HDI-0.2 achieved values of *R*_f_ and *R*_r_ of 99.9 and 98.2%, respectively. The influence of the crooslinking density was also evaluated, showing the importance of the reticulation process on the shape-memory properties. In this sense, while pure PVB displayed poor shape-memory properties characterized by an extremely low *R*_r_ value of 6.2%, PVB-HDI-0.2 exhibited a value of 98.2%; however, this value calculated for PVB-HDI-0.3 revealed a slight decrease, indicating that an excess of cross-linking points within the matrix is detrimental. In addition, the cross-linking density also affected the speed of the thermal-actuation, revealing that higher recovery times are required for highly cross-linked samples. A strong point of this work was the evaluation of the shape-memory properties of these gels under solvent immersion conditions (solvent-induced shape-memory), being especially relevant in biomedical applications.^265^ Like in dry conditions, samples recovered their initial shapes after immersing (Figure 30A). However, opposite to the thermal-induced actuation, the speed of the solvent-induced recovery process increased with the cross-linking density. This was attributed to a more difficult solvent penetration due to the existence of a lower free volume in highly reticulated samples. In addition, the actuation motions were evaluated in different solvents, allowing the authors to establish interesting correlations between chemical structure, polarity, molar volume, and recovery time parameters, among others. Additionally, PVB-HDI-*n* samples also shown self-healing properties under heating conditions. This property was evaluated, first, by scratching the surface of a dry sample and then heating the system over 100 °C. After the heating process, SEM images did not reveal any presence of scratch or scars (Figure 30B). Moreover, tensile strength values of 40.7, 22.8, and 36.9 MPa were calculated for original, scratched, and healed samples, demonstrating that over 80% lost strength was recovered. Authors argued that the healing mechanism was based on the activation of polymer chain motions at temperatures above *T*_m_ and *T*_g_ where, in addition to diffusion and re-entanglement phenomena,^266^ the internal stress released helps to bring into contact the surfaces across the damaged region. This was confirmed by performing successfully the healing process at temperature slightly above *T*_g_ (70 °C). As a pending task, the evaluation remained unsolved of a possible solvent-driven healing capacity.

**Figure 31:**
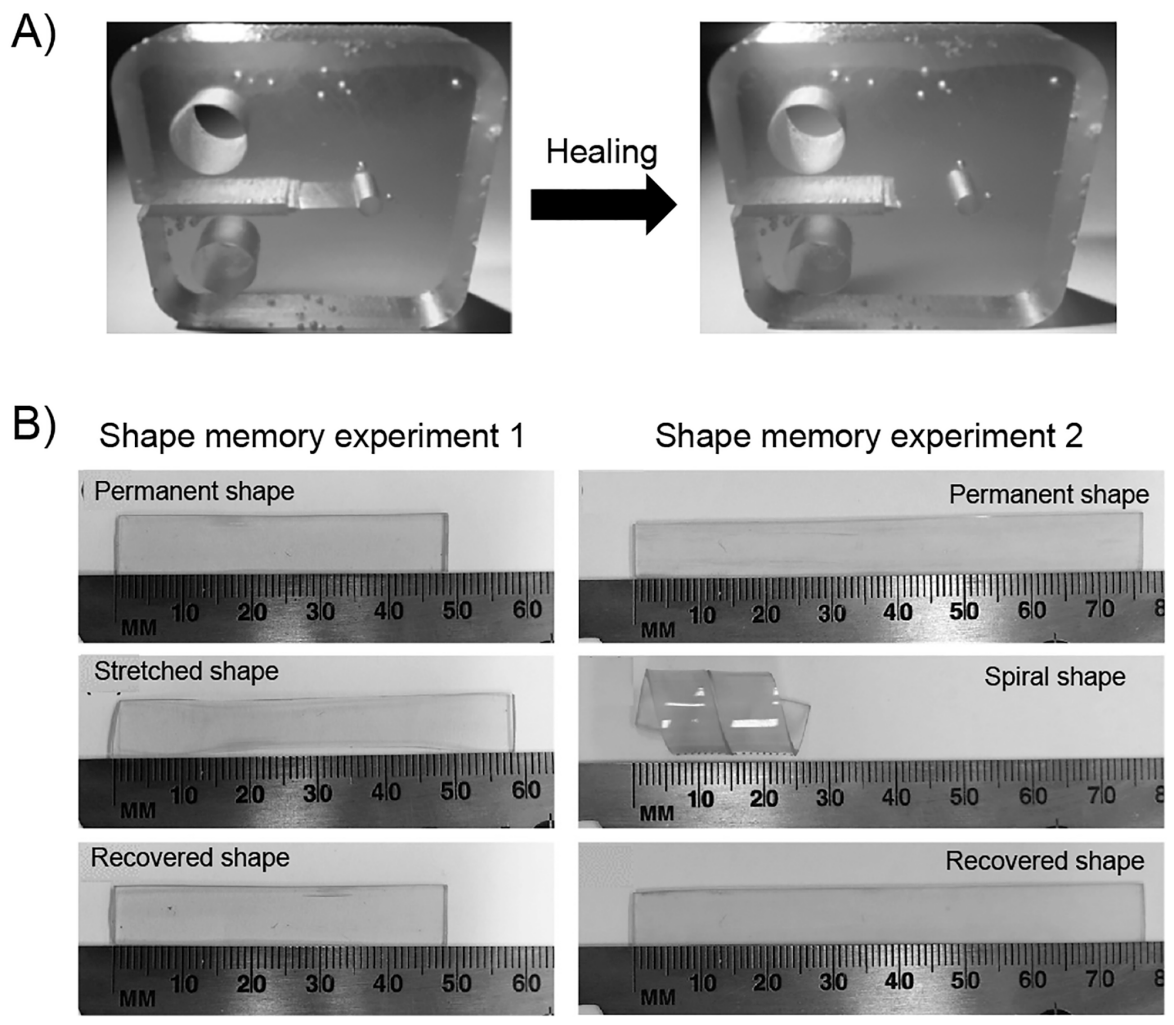
(A) Image of a specimen after cracking (left) and after the thermal healing process without the use of external forces (right). (B) Images of shape-memory experiments conducted using 1.5DA1T films showing their initial permanent configurations, their temporary shapes after deforming and fixing, and their thermally driven shape recovery process. Adapted with permission from ref (267). Copyright 2014 John Wiley and Sons.

Two months later, inspired by the reversible nature of DA reactions, Heo and Sodano carried out the synthesis of a reticulated polyurethane having in its chemical structure maleimide–furan DA adducts, allowing the preparation of a self-healable polymer material showing excellent results in terms of shape-memory function.^267^ In a first step, the authors carried out the preparation of the DS adduct by reacting furfuryl alcohol (FA) with *N*-(2-hydroxyethyl)-maleimide (HEM). Later, this diol adduct was employed in the synthesis of PUs by mixing it with triethanolamine (TEA) and HDI. Thereby, two different PUs (named 1DA1T and 1.5DA1T) were prepared by varying reactant ratios in the above formulations. Prior to the examination of PUs, the reversibility of the DA reaction performed by suitable monomers was successfully demonstrated using proton nuclear magnetic resonance (^1^H NMR). Then, by means of DSC measurements, the temperatures at which the DA and retro DA (r-DA) reactions take place in PUs were successfully determined. The DA and r-DA processes in 1DA1T were visualized at 97.3 and 131.6 °C, respectively, while for 1.5DA1T, the temperatures were very similar centering at 96.6 and 129.3 °C. These temperatures turned out the be slightly higher than previous reports,^265^ being ascribed to the high stability of the obtained polymer networks. In addition, 1DA1T and 1.5DA1T exhibited transitions assigned to *T*_g_ around 42.4 and 45.6 °C, respectively. With this information, the authors attempted to study the self-healing properties of both polymers. The healing process was carried out in an oven under nitrogen atmosphere. The fractured samples were first heated at 135 °C over 2.5 h and then incubated at this temperature for 2 h. Then, samples were cooled down to 90 °C over 1 h and kept at this temperature for 2 h to be finally cooled to 70 °C over 1 h and stabilized at this temperature for additional 2 h. The fractured samples were first heated at 135 °C over 2.5 h and then incubated at this temperature for 2 h. Then, samples were cooled down to 90 °C over 1 h and kept at this temperature for 2 h to be finally cooled to 70 °C over 1 h and stabilized at this temperature for an additional 2 h. As can be seen, samples were initially heated at temperatures above the r-DA reaction to induce the cleavage of unbroken linkages, allowing increasing of the possibility of healing the crack and then maintained at temperatures between DA and r-DA to ensure the reformation of DA adducts (Figure 31A). The self-healing efficiency was evaluated through fracture testing, measuring the fracture loads from both samples during three consecutive healing processes. In this regard, after the first, second, and third healing cycle, 1DA1T showed healing efficiencies of 79.76%, 69.30%, and 59.26%, while 1.5DA1T revealed values of 84.08%, 84.34%, and 75.89%, respectively. Overall, the healing efficiency measured after the first cycle is around 80–85%, being higher than other similar systems previously reported.^268,269^ On the other hand, healing efficiencies below 100% were attributed by authors, in part, to the limited efficiency of the recross-linking reaction of DA entities. In this regard, as was demonstrated by ^1^H NMR experiments, the cleaving process commanded by r-DA reactions was not complete in solution and, therefore, is expected to be less efficient in the solid-state due to diffusion and steric effects. However, the higher healing efficiencies measured for 1.5DA1T could be related to the higher amount of DA units present in its structure. After evaluating the healing property of these materials, the authors studied the shape-memory function triggered by thermal stimuli based on protocols previously reported.^270^ In a first instance, the shape-memory property of 1DA1T and 1.5DA1T was evaluated in terms of the shape recovery ability showed by these samples. To achieve this, a sample strip having an initial flat configuration as permanent shape was heated at 100 °C (above *T*_g_) for 5 min and deformed into a new temporary shape (stretched or spiral shape), which was fixed after cooling down the system to room temperature. Finally, samples were reheated at 100 °C, showing a complete recovery of their initial flat configurations showing an outstanding shape-memory function (Figure 31B). From the above, the authors argued that the healing process could also be supported by the shape recovery property shown by these PUs, where the emerged restoring force would help to close the crack.

**Figure 32:**
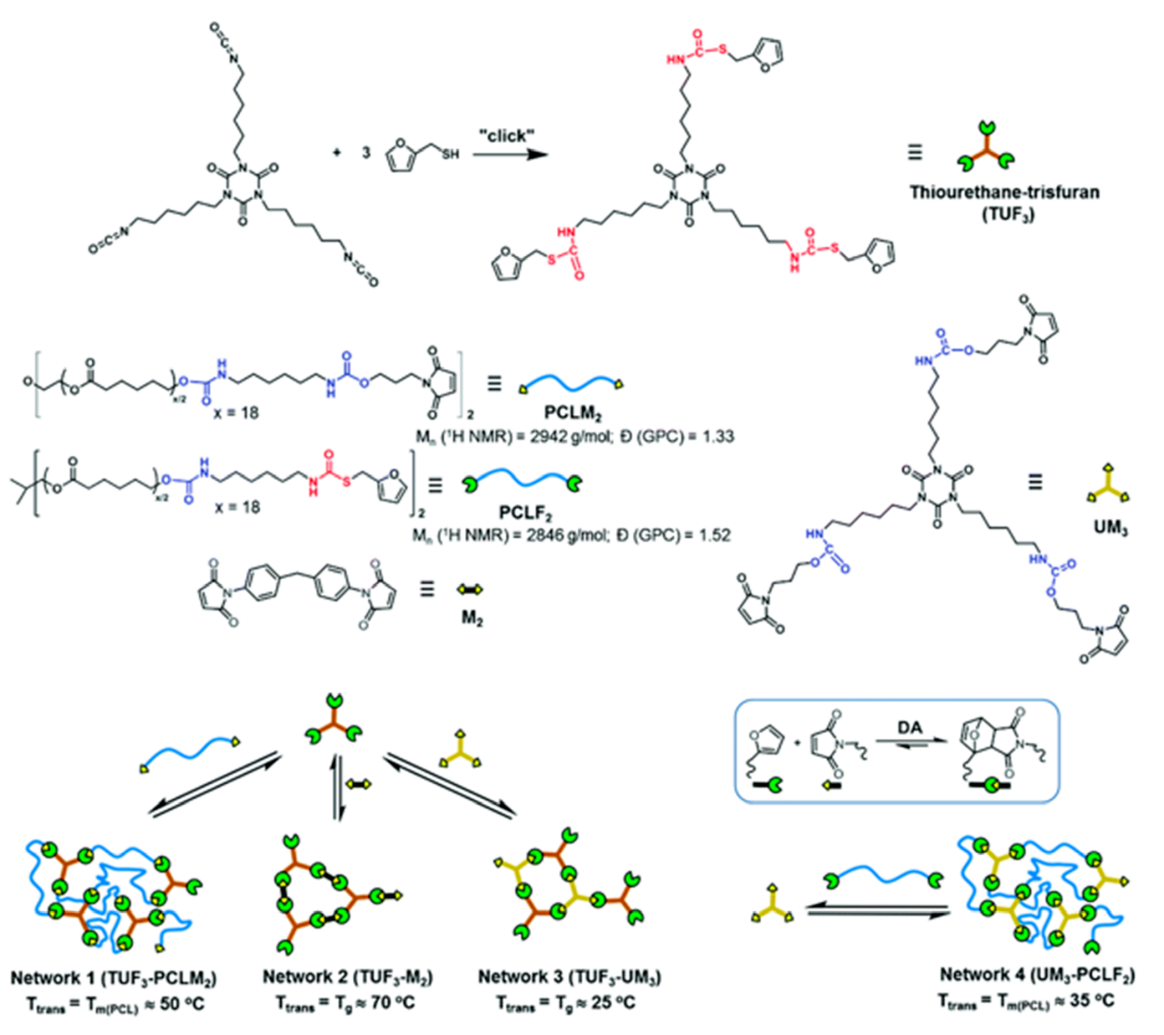
Chemical structures of the (thio)urethane multimaleimide and multifuran monomers and schematic depiction of the shape-memory networks reversibly cross-linked by DA reactions. Reproduced with permission from ref (271). Copyright 2015 Royal Society of Chemistry.

In 2015, Le-Thu and co-workers achieved the preparation of urethane–thiourethane macromolecular networks displaying a self-healing capability and actuation motions triggered by thermal stimuli.^271^ The reticulation process was achieved through the generation of DA adducts arising from the reaction between furans and maleimides entities.^272,273^ In this sense, a thiourethane-based structure having furans as terminal structures (TUF_3_) was prepared through the thiol-isocyanate “click” reaction,^274^ to be later cross-linked using a series of bis and trifunctional maleimides. These different cross-linked thiourethane networks were designed in order to achieve shape-memory switching temperatures within the temperature range where DA adducts formation take place (<100 °C).^275^ In this way, at one-single temperature the healing process, triggered by DA bond reformation, would be simultaneously supported by the shape-memory function of the system. Thereby, four different polymer networks were fabricated (Figure 32).

**Figure 33:**
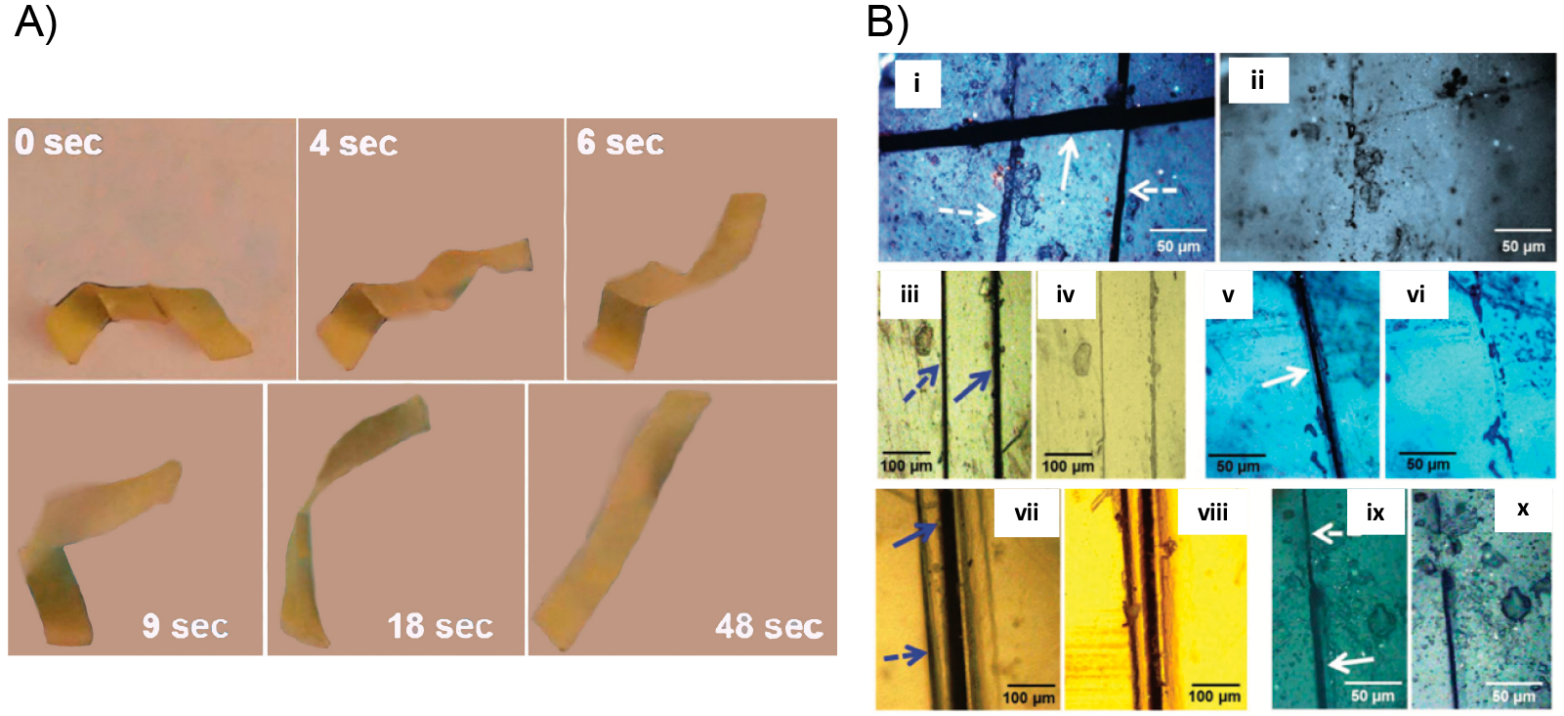
(A) Photographs showing sequential recovery from the temporary shape (spiral) to the permanent shape (strip) of network 3 at 60 °C. (B) Optical micrographs of scratches on the (thio)urethane network samples. Network 1 (i) before and (ii) after heating at 60 °C for 2 h. Network 3 (iii) before and (iv) after heating at 60 °C for 72 h. Network 3 (v) before and (vi) after heating at 120 °C for 10 min. Network 2 (vii) before and (viii) after heating at 70 °C for 72 h. Network 4 (ix) before and (x) after heating at 60 °C for 72 h. Solid arrows point to the wider scratches made with a scalpel blade, whereas the dashed arrows point to the narrower scratches made with a razor blade. Adapted with permission from ref (271). Copyright 2015 Royal Society of Chemistry.

Network 1 was prepared by reacting TUF_3_ with a bismaleimidic-terminated poly(ε-caprolactone) (TUF_3_-PCLM_2_), achieving a system which shape-memory function was based in melting–recrystallization phenomena. On the other hand, networks 2 and 3 were prepared by reacting TUF_3_ with a commercially available bifunctional maleimide (M_2_) and a urethane tris-maleimide derivative (UM_3_), respectively, affording amorphous materials whose shape-memory functions were induced by the glass transition phenomenon. By last, network 4 was constructed by seeking a similar structure to network 1 but placing furan structures at the end of PCL chains instead TUF_3_. Therefore, network 4 was prepared by cross-linking a bisfuranic-terminated PCL (PCLF_2_) with UM_3_. The cross-linked systems were prepared by mixing multimaleimide with multifurans substrates (1:1 furan-to-maleimide ratio) and dissolving them in tetrahydrofuran. The mixture was cast in a glass Petri dish at 40 °C for 48 h, followed by vacuum-dried at 60 °C for 24 h. Based on experimental evidence shown by the authors, the above conditions would be suitable to favor the formation of DA adducts between the monomeric species. Indeed, based on FTIR characterization, the high conversion values of 63.2 and 82% during the obtainment of the network 3 were achieved at room temperature after 3 and 12 h, respectively. Moreover, the cross-linking reaction at 30, 40, and 60 °C after 24 h showed a slight increment of the conversion values. Afterward, the thermal properties of these systems along with the studied of the DA and r-DA reactions was carried out using DSC measurements. All samples exhibited similar thermal transitions assigned to the reversible DA reaction. DSC thermograms displayed clear transitions assigned to the DA process (60–120 °C) followed by an endothermic process ascribed to the r-DA reaction (above 125 °C), demonstrating the reversibility of the DA reaction within these materials and, therefore, the cyclic behavior between cross-linking and cleaving processes. It is worth mentioning that the temperatures at which these transitions were observed are in good agreement with previous reports.^147,275,276^ Additionally, DSC measurements revealed the semicrystalline nature of network 1 and 4; however, for the latter, it seems that the inner structure of the system decreased crystalline degree and allow the detection of a small transition at 78 °C probably assigned to the *T*_g_ of a mixed phase between PCL and thiourethane portions. On the other hand, a mostly amorphous behavior was observed for networks 2 and 3. The authors determined that both processes, reversible DA adduct formation and the shape-memory function, were highly required to achieve an efficient and successful healing operation. The above due to the need to induce an intimate contact between the damaged surfaces to facilitate the reformation of bonds within the affected area through DA reactions. Therefore, before assessing the healing capability, the authors evaluated the shape-memory function of these samples by analyzing the shape recovery after being subjected to deformation stress. Samples were deformed into temporary shapes by heating them slightly above their *T*_m_ or *T*_g_ (85 °C for network 2, 60 °C for networks 1, 2, and 3). All samples could adopt complex temporary shapes (such as spirals), except network 2, which due to its higher rigidity only afforded simple configurations, such as U-shapes. Then, after being heated at the above-mentioned temperatures, samples recovered their initial configurations (Figure 33A). Tensile deformation tests were also used to achieve a more profound insight into the shape-memory function, allowing a better understanding of the observed thermally driven actuation motions. Finally, the self-healing ability of these networks was tested by scratching their surfaces and heating them at different temperatures, while the vanish of the damage was studied under optical microscopy (Figure 33B) and tensile tests. As the author mentioned, different results were obtained between the samples. In all cases, the temperature used in the healing process was mandatory to achieve good results. Overall, the temperature must be high enough to trigger both the shape-memory function and the reversible DA adducts reformation. Network 1 stood out by showing a suitable self-healing property under mild conditions and relatively short times. Conversely, due to the lack of an efficient shape-memory function, network 2 could not afford a complete healing process. Due to its higher cross-linking density, network 3 exhibited a high modulus material, showing a good healing process. In addition, this sample was used to demonstrate that even when higher temperatures trigger a better shape-memory property, if this temperature promotes de r-DA reaction, the healing process is notably suppressed. Surprisingly, even when networks 1 and 4 are very similar in terms of chemical structure, network 4 exhibited a low scratch-closing capacity, suggesting that the attachment of furans and maleimide into networks is an important parameter to be considered.

**Figure 34:**
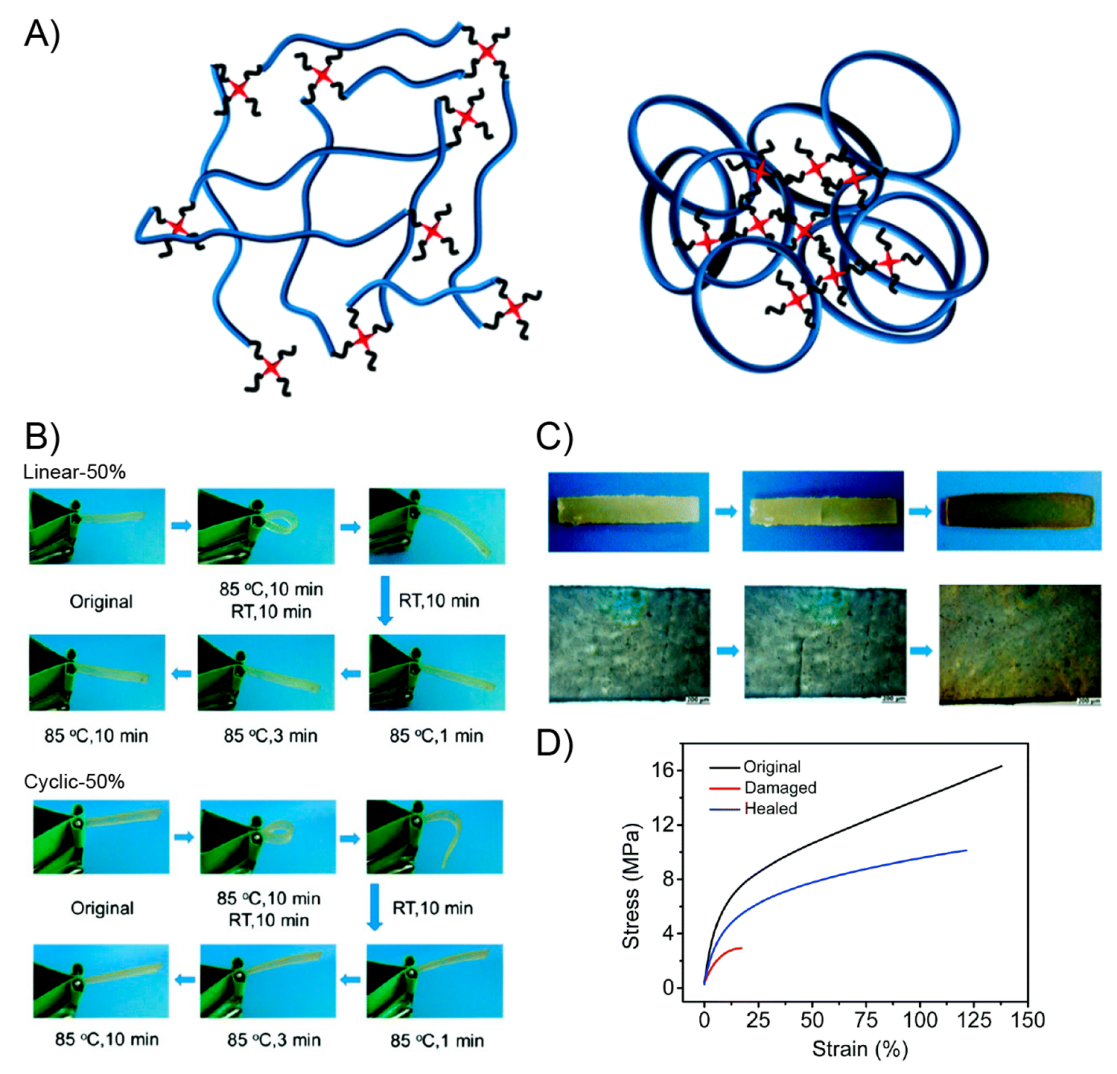
(A) Schematic representation of the network difference between linear (left) and cyclic (right) polymer-made PUs. (B) Pictures showing the shape-memory properties of linear-50% (up) and cyclic-50% (bottom) samples. (C) Digital and optical microcopy images of cyclic-50% sample during the thermally driven healing process. (D) Stress–strain curve for the original, damaged and healed cyclic-50% sample. Adapted with permission from ref (277). Copyright 2016 Royal Society of Chemistry.

By the end of 2016, Chen et al. designed an unprecedent example about the influence of the topology of polymers on the shape-memory properties and self-healing function of bulk materials.^277^ In order to achieve this, the authors performed successfully the preparation of a cyclic PCL polymer bearing two OH functional groups (c-PCL-2OH) and a linear PCL-diol (l-PCL-2OH), which were later used in the elaboration of highly cross-linked PU-based networks (Figure 34A). The synthesis of reticulated systems was carried out by mixing c-PCL-2OH or l-PCL-2OH with HDI (isocyanate source) and a tetra-ol entity (FM) obtained through protocols based on DA reactions. PU samples with different HDI compositions (35, 50, and 70 mol %) were prepared by using both types of PCL, being labeled as cyclic-X% or linear-X%. The thermal characterization of the obtained PU-based systems showed that the crystalline behavior of samples was highly affected by the amount and topology of PCL. Regarding the above, samples cyclic-50% and linear-50% exhibited a semicrystalline behavior supported by the clear observation of melting–crystallization phenomena by DSC measurements. However, for cyclic-70% and linear-70%, no evident melting–crystallization processes were observed. On the other hand, cyclic-50% displayed a higher *T*_m_ value accompanied by a lower Δ*H*_m_ than linear-50%. In addition, in all DSC thermograms, an endothermic peak was visualized around 130 °C, assigned to the retro DA reaction due to the incorporation of FM units in the material. This is of high relevance because these units would be involved in the self-healing process displayed by these materials. Due to the presence of PCL, all PU samples exhibited shape-memory process. This was attributed to the ability to fix the strain of the sample by crystallization and the subsequent recovery of the sample triggered by the elasticity gain at the rubbery state. In addition, it has already been reported that incorporating PCL into polymeric systems allows endowing them with adequate shape-memory function.^227,225,278−280^ As is shown in Figure 34B, sample strips were deformed into a “U” configuration, fixing them with the help of a binder clip. Then, the systems were heated above their *T*_m_ values for 10 min (85 °C), ensuring the free motions of the polymer chains. After completing the heating time, samples were cooled down to room temperature and equilibrated for 10 min. Afterward, one of the ends of the strip was removed from the binder clip (releasing the stress), maintaining the “U” temporary shape for 10 min. Finally, the deformed sample was heated up again at 85 °C, causing the recovery of its initial shape. It was demonstrated that PU systems prepared from c-PCL-2OH or l-PCL-2OH were able to recover their original shapes effectively, displaying excellent shape-memory properties. However, interesting differences were noted by changing the topology of the PCL component. Regarding the above, remarkable differences related to the ability to maintain the temporary shape (degree of fixing) were found (Figure 34B). In this sense, this property was increased by incorporating higher amounts of hard segments in the final PU material. In addition, for samples sharing the same HDI content (e.g., 50%), those incorporating cyclic PCL were able to retain in a better manner their temporary shapes. Aiming to achieve a more quantitative analysis, the fixing ratio (*R*_f_) and recovery ratio (*R*_r_) values for PUs were evaluated by DMA analysis. Surprisingly, over 5 consecutive cycles, linear-70%, cyclic-50%, and cyclic-70% exhibited fixing ratios values around 95%, while linear-35% and linear-50% revealed values below 80%. Therefore, as a trend, it can be considered that PU samples prepared using cyclic PCL would retain better temporary shapes than those containing linear PCL. Also, higher fixing ratios were obtained when the amount of hard segment (i.e., HDI) was increased. Another relevant result from this work was the unexpected diminishing of the fixing ratio while the PCL content rises. This result was discordant with previous works performed on PUs. Therefore, it seems that in this particular system, the fixing ratio was strongly dependent on the cross-linking density rather than the crystallizable portion. The unique behavior showed by PUs containing cyclic PCL was attributed by authors to the inherently different conformations that cyclic chains adopt within the reticulated system. In this sense, it could be possible that cyclic chains impose more positional restrictions,^281^ inducing the formation of more compact networks, traduced in higher fixing ratios. Based on the same arguments, the slightly lower *R*_r_ values registered for PUs based on cyclic PCL can be understood. By last, the self-healing performance displayed by these systems was studied. This property was envisioned after the incorporation of FM units into the PUs chemical structures because the dynamic nature of the covalent bonds formed between furan and maleimide would allow the occurrence of a reversible bond disruption–reformation process. In this sense, by ATR-FTIR the authors successfully demonstrated the reversibility of the DA adduct present in PUs and, consequently, their thermally driven self-healing capability. To test this, a razor blade crack was made on a cyclic-50% film. This film was put in an oven and heated at 130 °C for 4 h (promoting the r-DA reaction) and then stored at 60 °C for 48 h (promoting DA reaction), allowing the complete disappearance of the crack (Figure 34C). Finally, healing efficiencies were calculated by performing tensile stress–strain experiments using DMA (Figure 34D). The cyclic-50% damage sample experienced a dramatic reduction of its mechanical properties, being easily broken under mechanical stress; however, after the healing process, the mechanical strength was notably enhanced. It is worth noting that all PU samples showed healing property, where no evident difference was noted between systems prepared from cyclic or linear PCL.

**Figure 35:**
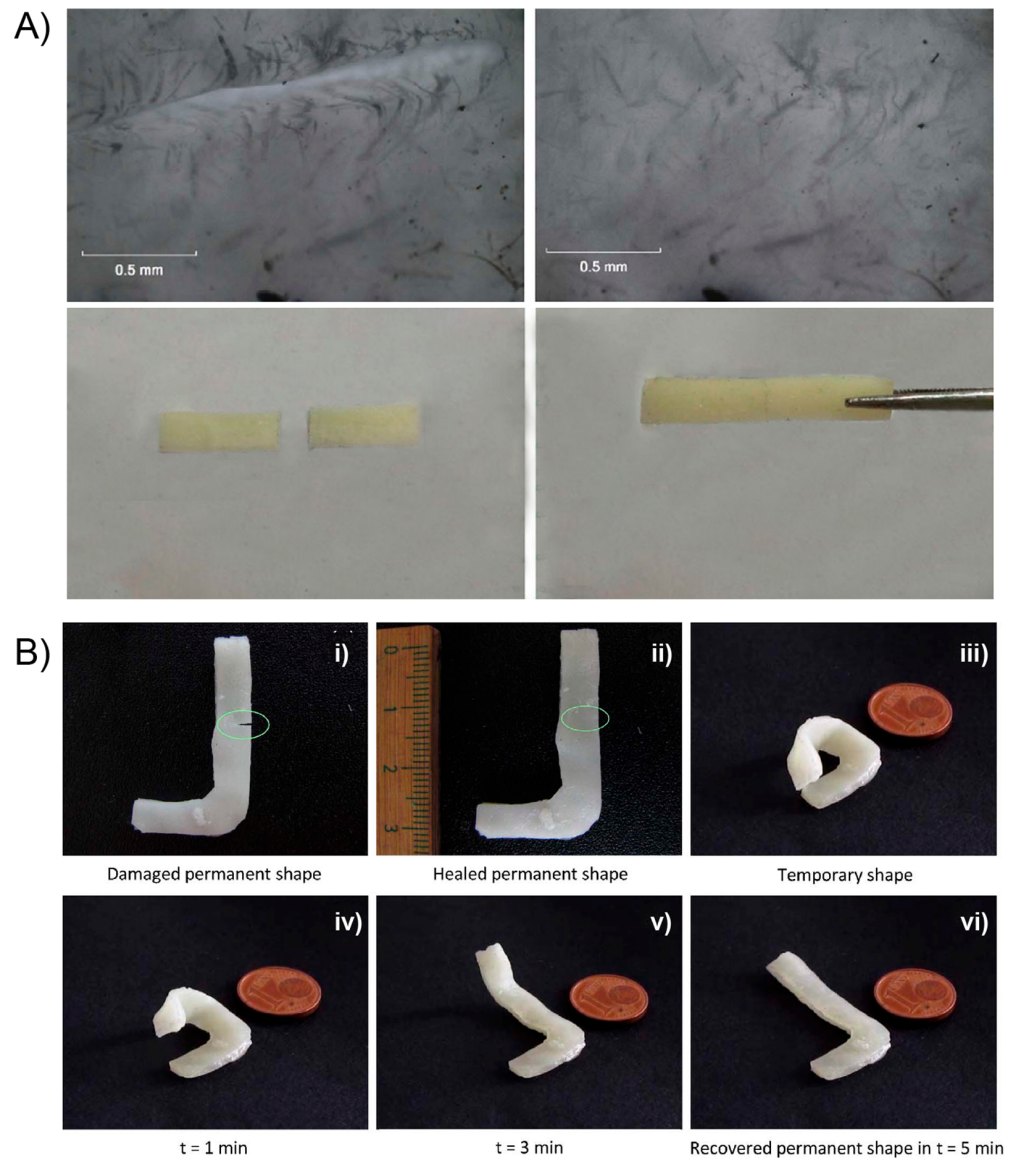
(A) Optical microscopy images (above) and digital photographs (bottom) of a printed PCLDMA-UPyMA sample before (left) and after (right) self-healing process. (B) Shape-memory effect of PCLDMA-UPyMA repaired samples. The specimen was cut (i) and repaired after a thermal treatment of 1 h at 80 °C (ii). The deformed object (iii) was heated at 70 °C to start and complete the recovery of the original shape (iv–vi). Adapted with permission from ref (282). Copyright 2018 Elsevier.

When a 3D printed material is endowed with the ability to adapt or change its shape through time is knowing as four-dimensional (4D) printing. Based on this concept, in 2018, Invernizzi et al. reported for the first time a 4D printed shape-memory material, also displaying thermally induced self-healing capability.^282^ The authors describe a polymeric 4D material prepared by digital light projection (DLP). This technique allows the production of materials with intricate designs in a fast manner. The foundations of the DLP process are based on the projection of an image over a platforms surface. The precise projection of the image is achieved by means of a digital micromirror device that guides the light toward a microchip, allowing the respective coordinates to be sent to print the desired material. On the other hand, the printable formulation used in this work corresponded to a mixture of end-modified PCL chains with methacrylate units (PCLDMA), a methacrylic monomer bearing UPy motifs (UPyMA), and a photoinitiator. Thus, during DLP a photoinduced cross-linking process via UV–visible light is triggered, inducing the formation of a reticulated polymer network named PCLDMA-UPyMA. The introduction of UPy structures was supported by previous publications in which it is demonstrated that endow the materials with an adequate self-healing property mediated by reversible hydrogen bonding.^283−286^ Rectangular printed samples were prepared and used for further thermal and mechanical characterization, as well as for the evaluation of the shape-memory property and self-healing function. In addition, for comparison purposes, the authors also carried out the preparation of photocured PCLDMA and PCLDMA-UPyMA samples by casting. The mechanical properties of the printed and cast samples were evaluated in terms of maximum tensile strength and elongation at break showing that, regardless of the tensile test speed, all samples exhibited similar tensile strength values, but the cast PCLDA-UPyMA displayed higher elongation at break values. This was ascribed to the presence of hydrogen bonding interactions within the structure. However, the above argument did not support the lower values achieved by the printed PCLDA-UPyMA, which also should count with the presence of these interactions. The authors defended this result based on previous reports that revealed that during the printing process some voids and defects can be incorporated into the material structure, affecting their mechanical strength.^287,288^ By aiming to evaluate the self-healing properties displayed by these materials, two types of injuries were exerted: surface and bulk damage. Regarding surface damage, deep scratches were induced over the printed PCLDMA-UPyMA sample, which was completely repaired after treating the sample at 80 °C for 1 h. On the other hand, under the same thermal-driven protocol, the bulk damage (which consisted of the bisection of the sample) was completely healed, achieving a perfect binding between both parts (Figure 35A). The curing process was also studied by analyzing the changes in the mechanical properties of materials. Healing efficiencies for printed samples, based on the recovery of tensile strength property, were around 10% higher than the ones obtained through casting. The better healing property of printed samples could be ascribed to their rougher interface observed at the damaged area that could be prompt a more efficient polymer chain interpenetration along with a higher amount of hydrogen bonds interactions. Additionally, printed PCLDMA-UPyMA exhibited outstanding shape-memory function characterized by *R*_f_ and *R*_r_ values of 99.8 and 98.6%, respectively, showing better results than other 3D printed materials.^289^ Seeking to contribute to the field of soft robotics, the authors printed a “L” shaped PCLDMA-UPyMA sample trying to replicate the index finger and the thumb (Figure 35B). This sample was first cut and successfully healed by thermal treatment. Then, the sample heated above *T*_m_ was deformed and cooled down to room temperature, inducing the recrystallization of PCL domains, allowing to fix the new temporary shape. Then, the sample was heated again above *T*_m_, allowing the recovery of the “L” shape. These thermally activated actuation motions are released during the melting of the crystalline phase and driven by entropic elasticity phenomena.^290−292^ More importantly, printed and repaired samples’ shape-memory and healing functions were as good as the original printed samples.

**Figure 36:**
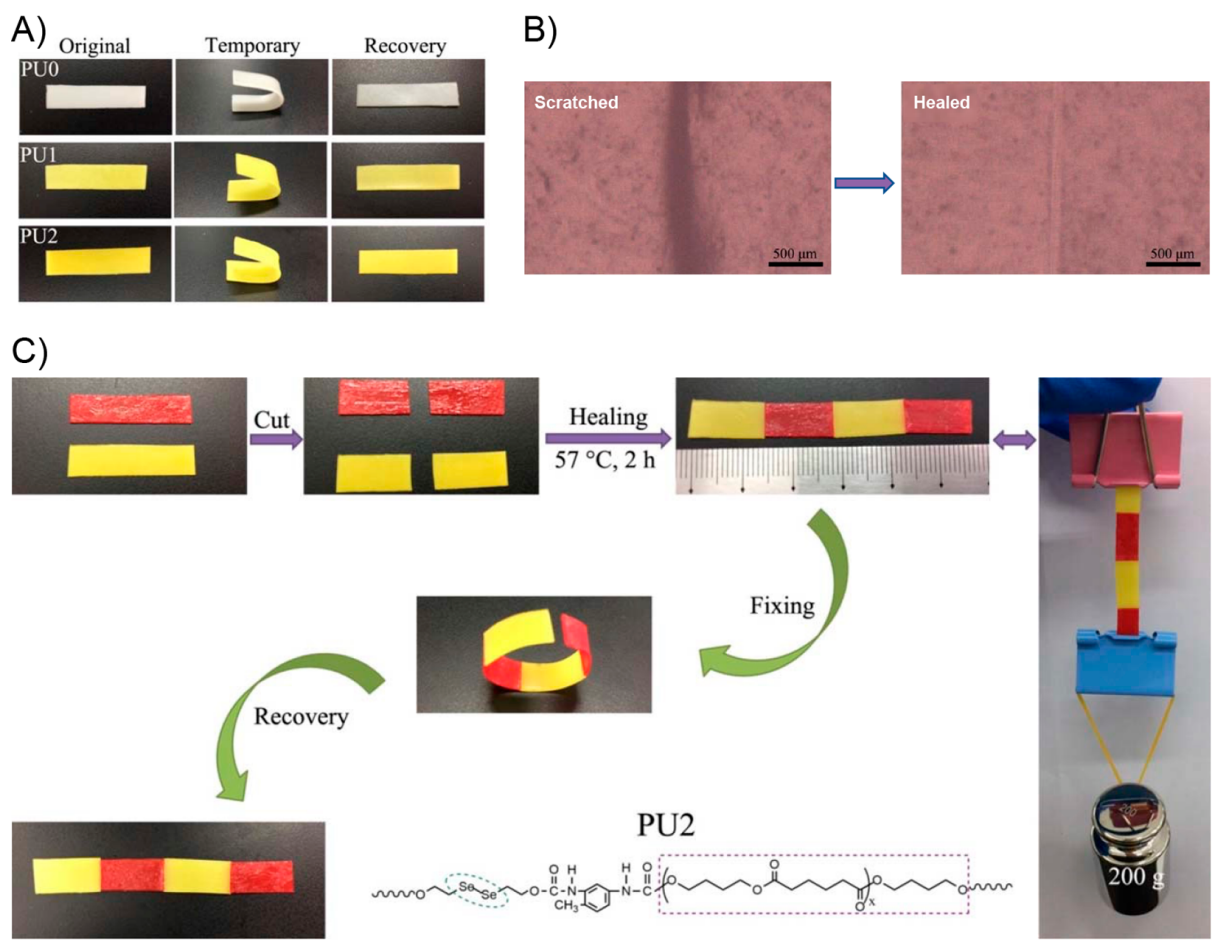
(A) Thermally driven shape-memory process of PU0, PU1, and PU2. (B) Representative SEM surface micrographs of PU2 before and after healing. (C) Photographs of consecutive thermal induced self-healing and shape-memory functions triggered in PU2, also showing the mechanical strength of this sample by lifting a 200 g weight. Adapted with permission from ref (293). Copyright 2018 John Wiley and Sons.

Du et al. studied the effect of incorporating diselenide linkages into PU matrices. This strategy allowed the preparation of self-healable polymer networks, showing shape-memory function.^293^ By following typical polymerization protocols, the authors conducted the synthesis of a series of PUs based on semicrystalline poly(butylene adipate) (PBA) and 2,4-toluene diisocyanate (TDI). However, the novelty of this work was the incorporation of two types of chain extenders; the commercially available BDO and DiSe, previously reported by the same group. Based on the above, three types of PUs were synthesized named PU0, PU1, and PU2. PU0 and PU2 were prepared by using BDO and DiSe, respectively, as chain extenders while during the synthesis of PU1 an equimolar amount of BDO:DiSe was employed. The chemical structure as well as their macromolecular nature was successfully studied by means of FTIR, NMR, XRD, and gel permeation chromatography (GPC) analysis. On the other hand, their thermal and mechanical properties were also evaluated. Regarding thermal properties, DSC measurements showed that all PUs revealed three thermal transitions that can be dominate importantly their shape-memory properties.^294^ These transitions were ascribed as glass transition temperature (*T*_g_), cold crystallization (*T*_c_), and melting temperature (*T*_m_). A more detailed analysis of the thermal properties ascribed to the semicrystalline structure of PUs would reveal an increase of the *T*_g_, *T*_c_, and *T*_m_ values as the amount of diselenide bonds also increased. Conversely, at higher amount of diselenide linkages lower crystallinity degrees were achieved. Then, the mechanical properties of these polymers were studied by DMA analysis. These results showed that for all PUs, large differences in their storage modulus were detected when measured above and below the transition temperature (*T*_g_), revealing potentially a suitable shape-memory function.^295^ Indeed, the authors found that the inclusion of diselenide bonds into PU structures allows decreasing of the ratio of the storage modulus calculated above and below the transition temperature, potentially affecting their shape-memory properties. The shape-memory function for these PU were studied using the bending test, aiming to calculate the *R*_f_ and *R*_r_ values. It is well-known that the shape fixing of semicrystalline polymer systems would be related to the crystallization of their soft segments, whereas the shape recovery function to the elasticity of samples.^296^ The calculated *R*_f_ values for PU0, PU1, and PU2 were 99.6%, 94.1%, and 95.3%, respectively, while the corresponding *R*_r_ values were 98.4%, 96.2%, and 96.7%, respectively. From these results, it can be concluded that all samples exhibited excellent shape-memory properties. However, results indicated that the presence of diselenide bonds slightly decrease both parameters and, thereby, the shape-memory function of samples, confirming the speculations conducted from DMA results. Based on the crystalline behavior showed by samples, while the decrease of *R*_f_ in PU1 and PU2 could be supported by their lower crystallinity degrees, the diminishing of their *R*_r_ values was not expected. The authors argued this result in terms of interaction restrictions between polymeric chains due to the presence of selenide entities, which facilitates the sliding process of macromolecular entities. All samples showed good reliability of the shape-memory function by keeping *R*_f_ and *R*_r_ values above 90% over 5 consecutive cycles. Figure 36A illustrates the shape-memory process carried out by PU0, PU1, and PU2 bulk samples. Each sample was remolded into a “U” temporary shape by heating them at 57 °C (*T* > *T*_m_), followed by cooling down the system at 0 °C for 5 min to fix the new configuration. Then, the reshaped samples were heating again at 57 °C during 10 min, showing a nearly complete shape recuperation. The dynamic exchangeable property of diselenide bond motivated the study of the self-healing properties of these PUs. To achieve this, healing efficiencies were calculated in terms of the recovery of tensile strength and elongation at break values, which were obtained using tensile testing measurements. Scratched samples showed a dramatic decrease of their mechanical properties when compared to nondamaged PUs, showing to be rapidly fractured under mechanical stress. Then, PU samples were subjected to a thermally driven healing process by heating them at 57 °C for 2 h, showing that, with exception of PU0, both tensile strength and elongation at break parameters were notably improved. In addition to the above, healing efficiencies were enhanced by increasing the content of DiSe structures within the polymer structure. In this sense, PU2 exhibited the better healing capacity characterized by healing efficiency values of 87.6% and 89.1% calculated from the recovery of the tensile strength and elongation at break values relative to the original sample. The healing mechanism proposed by the authors would be mainly driven by the melting and recrystallization process carried out by the soft segments of the material but also supported by the dynamic nature of diselenide bonds that, under thermal stimuli, are capable of performing reversible exchange reactions that would also promote the healing of the material.^297,298^ The authors also conducted a visual inspection of the healing process through SEM analysis (Figure 36B) corroborating the better healing ability of PU2. In this sense, PU2 allowed the complete closure of the crack, leaving a slight scar after the healing process, while PU1 achieved the narrowing of the scratch but not its complete disappearance. Contrary to the above, after the healing treatment, no evident changes between the damaged and healed PU0 samples were observed. On the other hand, the robustness of the PU2 sample was successfully demonstrated by inducing, in a consecutive manner, its healing and shape-memory function (Figure 36C). Furthermore, due to its excellent healing capacity, the PU2 sample could be reprocessed and subsequently lift a weight of 200 g.

**Figure 37:**
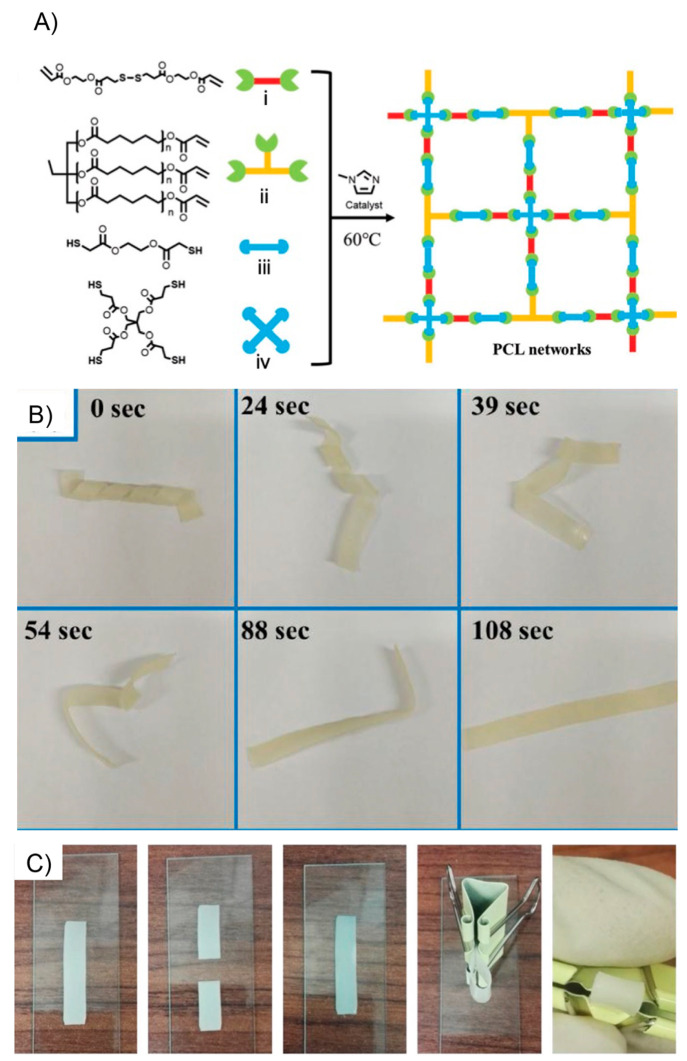
(A) Schematic representation of the synthesis of PCL networks. (B) Photographic sequence of the healing process. (C) Photographic sequence of shape recovery from a temporary shape to the permanent shape at 60 °C. Adapted with permission from ref (299). Copyright 2018 John Wiley and Sons.

Later in that year, Zhang et al. carried out the fabrication of a new PCL-based network reticulated by monomers bearing disulfide linkages. The material stood out by presenting multiple functional properties such as shape-memory, reprocessability, degradability, and more importantly, a fast self-healing under mild conditions.^299^ The authors focused their efforts on achieving higher healing efficiency values than those previously reported for other PCL-based systems. As strategy, they carried out the cross-linking process via thiol–ene “click” reactions between a branched PCL derivative (ii) and a novel disulfide monomer (i) (both containing acrylate entities as terminal structures) using, simultaneously, two different thiol-containing structures as cross-linking agents (iii and (iv) (Figure 37A). Different formulations were achieved by varying the molecular weith of PCL as well as the ratios between PCL and the disulfide monomer. The success of the reaction was confirmed by FTIR, where no visible signals for alkenes from acrylate groups and thiol functionalities were detected. On the other hand, the cross-linked structure of the obtained material was demonstrated by gel content values above 90%. DSC measurements allowed determination of what PCL networks exhibited a semicrystalline behavior characterized by *T*_m_ values in the range of 40–50 °C and a *T*_g_ centered at −15 °C. Therefore, the activation of polymer chains motions would be achieved by exposing samples at temperatures slightly above their *T*_m_ values (considered as mild conditions based on the authors appreciation). Initially, the healing capability of samples was tested by bisecting a specimen in two parts, followed by placing them in close contact at 60 °C for 1 h. After slowly cooling at room temperature, a one-piece material was obtained, displaying no visible marks on its surface. This sample was able to bend over 90° without showing crack reopening (Figure 37B). Using the mechanical properties of the original samples as reference, several healing efficiencies were calculated from the recovery of their mechanical properties. In this sense, PCL networks prepared in this work achieved healing efficiency values as high as 94% and 92% based on the recovery of their Young’s modulus and yield strength parameters, respectively, being higher than those reported for other self-healable PCL materials based on UPy units and DA adducts. In addition, the sample prepared without disulfide bond showed healing efficiency values of zero, demonstrating the importance of the introduction of this type of moiety into the polymer structure. It is well-known that the exchange reaction of disulfide bonds exhibits a high activity and takes place at moderate temperatures.^262,300^ Thereby, a remarkably enhancement for the healing efficiency values could be achieved by increasing the healing times or the healing temperatures. The authors also evaluated the effect of the PCL/disulfide monomer proportion and the effect of the molecular weight of PCL on the healing capacity of networks. Overall, the increment of the disulfide monomer up to 40 wt % in the formulation allows increase in an outstanding manner the healing efficiency reaching values as high as 99%, confirming the importance of these units in the process. Moreover, the increase of the PCL molecular weight also allowed the increment of the healing efficiencies but in a more conservative manner. Finally, the optimal composition to achieve a PCL network showing self-healing function along with good thermal and mechanical properties was using a 20% composition of disulfide monomer and a PCL with a molecular weight of 7000 g/mol. Afterward, the shape-memory property of this optimized sample was evaluated. The sample exhibited a good shape function being able to adopt complex temporary shapes and recovering from them under thermal stimuli (Figure 37C). Based on the above, an initially flat strip sample twisted into a spiral configuration during heating to 60 °C, successfully retaining the new temporary shape after rapidly cooling to room temperature. Then, after being reheated at 60 °C, the sample recovered its initial configuration in 108 s. A more quantitative perspective was given to the shape-memory evaluation by DMA analysis, where the authors calculated a shape fixing ratio and shape recovery ratio of 98% and 95%, respectively, denoting the outstanding shape-memory function delivered by the system. Interestingly, the authors devised an ingenious strategy to promote the degradation of these reticulate systems based on the reversible nature of disulfide bonds.

**Figure 38:**
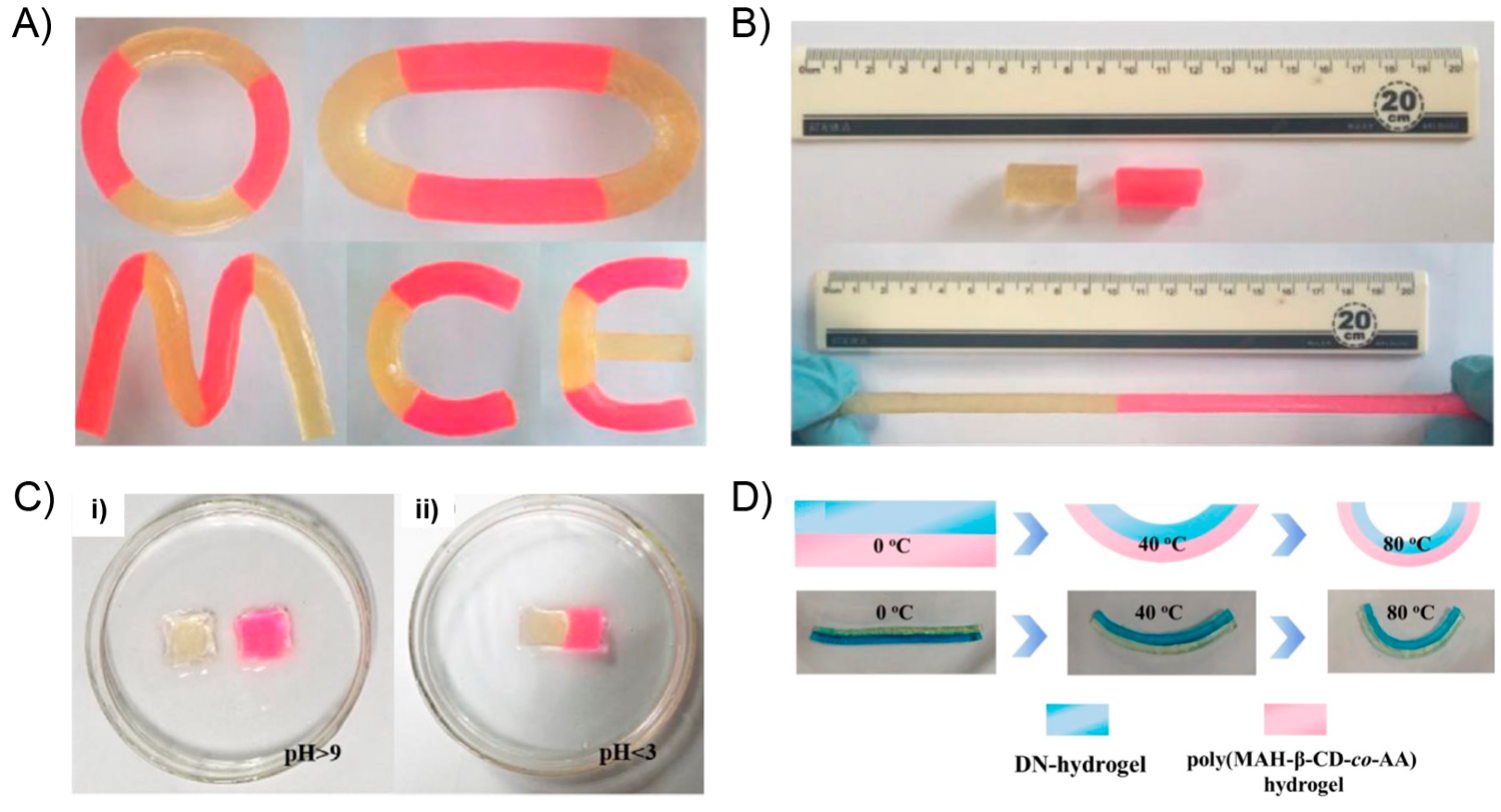
(A) Various shapes and letters obtained by bringing the freshly fractured surfaces into contact. (B) Self-healing and stretching behaviors of poly(MAH-β-CD-*co*-AA) hydrogels. (C) Deprotonated and protonated cylindrical hydrogels in (i) urea solution (pH > 9) and (ii) acidic solution (pH < 3). (D) Schematic representation and digital photos of the actuation motion observed for poly(MAH-β-CD-*co*-AA/DN bilayer hydrogel actuator in hot water (40 and 80 °C). Adapted with permission from ref (301). Copyright 2019 Elsevier.

Trying to contribute to the elaboration of new self-healable hydrogel systems with high mechanical strength, at the beginning of 2019, Li et al. presented for the first time the preparation of a self-healable hydrogel via frontal polymerization (FP).^301^ To achieve this, the authors first performed the synthesis of MAH-β-CD, a β-cyclodextrin derivate bearing a vinyl carboxylic acid group from maleic anhydride (MAH).^302^ This derivate was then mixed with acrylic acid (AA) and radically reticulated by using ammonium APS and *N*,*N*′-methylene(bis(acrylamide)) (MBAA) as initiator and cross-linking agent, respectively. It is worth noting to mention that the authors performed a complete and deep study seeking for the optimum conditions to perform the FP of the system. Finally, they managed to obtain a series of poly(MAH-β-CD-*co*-AA) hydrogels from different MAH-β-CD:AA ratios, ranging from 2:2 to 2:5. All hydrogels exhibited an adequate swelling property in water that was notably enhanced by increasing the amount of AA in the hydrogel structure. The above result was not only ascribed to the more hydrophilic nature of AA but also to the porous morphology exhibited by the obtained samples, where higher average pore sizes were visualized for samples having higher AA content. In addition, the swelling property was also tested at different pH values showing an enormous increase under alkaline conditions owing to the deprotonation of carboxylic acids increasing the hydrophilic behavior of the system. The mechanical properties of hydrogels were analyzed by strain–stress experiments, demonstrating a high mechanical strength property. In this sense, the authors ascribed the strength of the material to the presence of MAH-β-CD where, by decreasing the amount of this entity in the cross-linked structure the tensile strength of the material decreases while the elongation increased remarkably. On the other hand, by rheological experiments a reticulated chemical structure for all samples was successfully corroborated. The self-healing capability exhibited by these materials was evaluated first by cutting into two parts a hydrogel sample and then merging both freshly fractured surfaces. The healing process was allowed to proceed in the absence of any external stimuli, achieving successfully the obtaining of a one-piece material. Following the above process, hydrogel pieces displaying various complex shapes were prepared (Figure 38A). In addition, the effectiveness of the process was demonstrated based on the high stretchability shown by healed samples (Figure 38B). The authors argued that the healing mechanism was based on the multiple hydrogen-bonding interactions taking place between carbonyl and hydroxyl functional groups, abundantly present within the hydrogel structure. Aiming to confirm this hypothesis, they immersed healed samples into an alkaline solution observing the disassembly of the specimen due to the deprotonation of carboxylic groups and, consequently, the disruption of hydrogen bonds (Figure 38C).^303^ Conversely, samples successfully retained their structures after being immersed in acidic media. The healing efficiencies of samples were highly dependent on the AA content, going from 79.2% to 94.3%. Additionally, the optimum conditions for achieve a complete healing process were 24 h at room temperature. Finally, due to the current involvement of hydrogel actuator in fields such as biomedical and soft robotics,^304−308^ the authors devised a strategy to endow these hydrogels with an actuation property. The strategy consisted in the fabrication of bilayered hydrogels by incorporating *N*-isopropylacrylamide (NIPAM) monomers into poly(MAH-β-CD-*co*-AA) hydrogels and promoting their polymerization. A double network (DN) hydrogel was obtained, sustained by host–guest interactions between β-CD and NIPAM entities that allow the cross-linking between both networks.^309^ The bilayered hydrogel revealed thermally driven actuation identified as bending motions toward the DN hydrogel side (Figure 38D). The actuation property would arise from the asymmetric volume expansion existing between both reticulated layers, ascribed to a higher cross-linking density in the DN hydrogel. As authors mentioned, the strategy delivered in this work could be broadly applied in the fabrication of new and versatile soft actuators.

Also in 2019, Kong et al. described a self-healing and shape-memory polyimide (SHSMPI) based on poly(amic acid) (PAmA) upon scattering polystyrene into the matrix as healing agent.^310^ The optimum amount of PS was established in 8%, while higher content of SP caused the loss of the shape-memory property. This material exhibited quantitative *R*_f_ and *R*_r_ after 18 s at 243 °C, and self-repairing within 4 min after a crack.

Very recently, Meng et al. reported a bioinspired material which combines fluorescence color-tunable, shape-memory, and self-healing properties triggered by temperature.^311^ In addition, this material showed outstanding tensile properties with a breaking tensile value of 1600%. In this work, the fluorescence color change was given by the photophysical effect known as aggregation-induced emission (AIE), where the luminescence of aggregation-induced emission agents (AIEgens) is activated by the formation of aggregates. In first place, the authors prepared a shape-memory and self-healing polyurethane-based material by combining PTMG, as soft segment, and MDI, as hard segment. Subsequently, the obtained matrix was allowed to react with bis(2-hydroxyethyl) disulfide (HEDS) as chain extender, which would enable later the self-healing capacity through a temperature-triggered disulfide exchange process. In order to achieve changes in the fluorescence, they selected tetraphenyl ethylene (TPE-COOH) and (*E*)-4-(((2-hydroxynaphthalen-1-yl)methylene)amino) benzoic acid (HNMA) as AIEgens. These compounds were embedded in the polyurethane matrix, obtaining the materials labeled as P-H_1_T_0_, which only contains HNMA, and P-H_0_T_1_, which only contains TPE-COOH. To get several materials with different proportions, P-H_1_T_0_ and P-H_0_T_1_ were mixed in an appropriated mass ratio in DMF, and the solvent was subsequently evaporated allowing the formation of a film, which was then cut into small strips for further analyses. The thermal transition temperature (*T*_trans_) of the material obtained by DSC was ≈20 °C. The TPE-COOH and HNMA showed fluorescence values 2.75 and 2.52 times higher at −196 °C than that at 25 °C, respectively. Besides, the P-H_1_T_0_ and P-H_0_T_1_ at −196 °C showed values 7.14 and 9.68 higher than those at 25 °C, respectively. This behavior can be attributed to the movement of the AIEgens along the polymer matrix. When the temperature is below the *T*_trans_ (−20 °C), the polymer chains are in a glassy state, in which the AIEgens have less space to move, increasing the fluorescence. The shape-memory process results from the reversible phase transition between the hard and the soft segments due to dipole–dipole interactions, hydrogen bonding, or crystallization. If the material is heated above the *T*_trans_ (50 °C), it can be deformed into a temporal shape, which is set by cooling down below the *T*_trans_ (−20 °C). The material recovered its original shape by reheating at 50 °C (*R*_r_ = 83%, *R*_f_ = 96%). As mentioned, the self-healing process is dominated in this example by the disulfide exchange bonds associated with the end-chain HEDS. This process can take place at 20–70 °C, showing a modest healing efficiency of around 60%. Finally, the authors designed a reprocessing thermal scheme, which allows the soft actuator to transform it shape from 2D shape into 3D shape by taking advantage of the self-healing properties.

### 2.5 Self-Healing Electric Actuators

**Figure 39:**
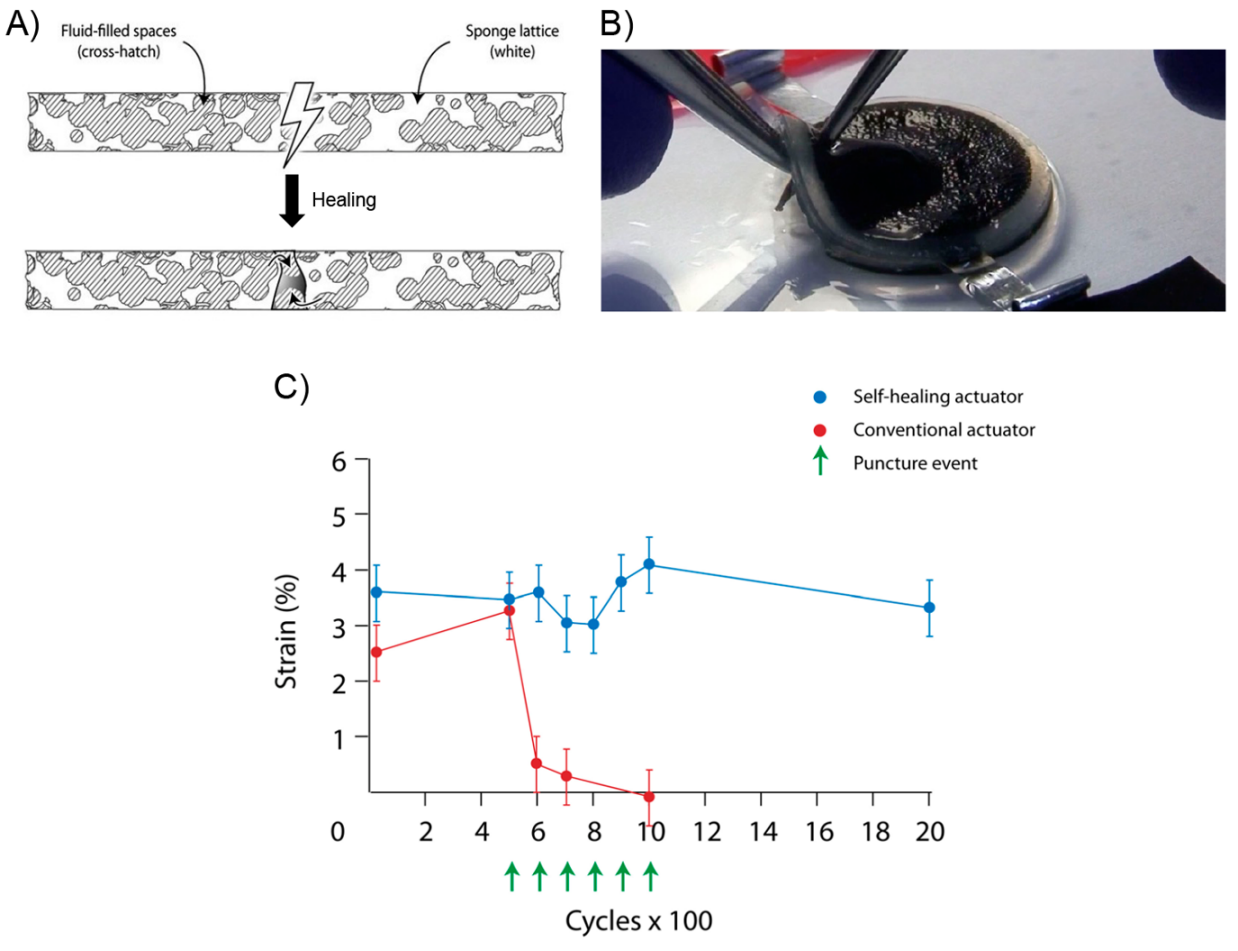
(A) Mechanism of self-healing in the dielectric and (B) graph showing self-healing efficiency. Adapted and modified with permission from ref (312). Copyright 2014 John Wiley and Sons.

One of the first reports about a self-healable electric actuator appeared in 2014 when Hunt et al. reported a two-phased compliant dielectric that is made up of a silicone sponge saturated with silicone oil.^312^ In the case of a dielectric breakdown, the oil from the sponge was able to flow back into the defects created thus healing the dielectric structure (Figure 39A). Furthermore, they demonstrated that a dielectric elastomer (DE) actuator can self-heal and continue to function with the same efficiency even after being damaged multiple times. DEs can be described as stretchable capacitors constructed by sandwiching a compliant dielectric between two stretchable electrodes. Under an applied voltage, generated electrostatic forces deform the DE, resulting in actuation motions. The dielectric elastomer actuator (DEA) was fabricated by embedding two flexible carbon grease circular electrodes, each 2 cm in diameter, in a layer of silicone, and a circular silicone sponge. The electrodes were then connected to opposite poles of a high voltage power supply set at 3.5 kV. The actuation was carried out at a frequency of 2 Hz for a total of 2000 cycles. The actuation motions were traduced into strain values considering the variation of area values displayed by the sample under voltage stimuli. To test the self-healability, the actuator was punctured at random points and at various intervals to simulate electromechanical breakdown of the dielectric layer (Figure 39B). To actuate a DE, high electric fields, often in the order of tens to hundreds of Megavolts/m,^313^ are applied across it in order to generate electrostatic forces enough to trigger the actuation. Such high electric field often results in dielectric breakdown,^314,315^ whereby the voltage is discharged suddenly between the electrodes, and therefore, across the material, resulting in heat generation and causing a breach in the dielectric. The self-healability of the actuator after a dielectric breakdown event was investigated by increasing the voltage gradually until the breakdown voltage, after which the voltage was reduced back immediately. It was found that as long as the voltage was reduced instantaneously upon reaching the breakdown point, the same area strain could be achieved afterward (Figure 39C). The authors differentiated between “self-clearing”, which results in loss of actuation performance due to loss of dielectric material, and “self-healing”, which is to repair/remove any defect formed such that the dielectric material is capable of retaining its structure and actuation performance.

**Figure 40:**
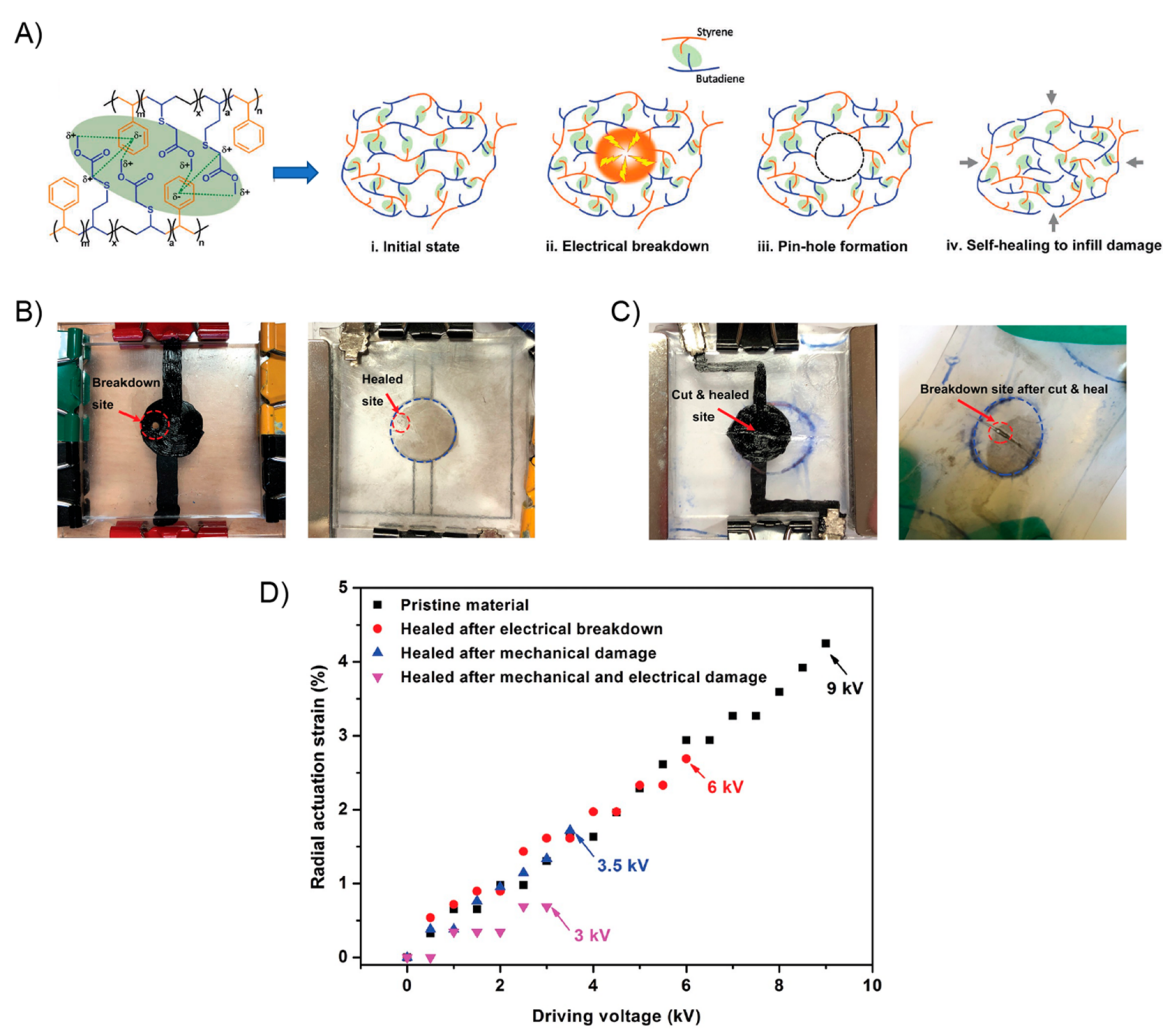
(A) (left) Healing mechanism with the δ+ proton adjacent to the ester interacting with the δ− aromatic center of styrene. (right) Scheme of the self-healing process after electrical breakdown where (i) initial state, (ii) electrical breakdown leading to vaporization of polymer, and (iii) formation of pinhole, (iv) healing and infilling of pinhole with application of pressure. (B) Actuator device with (left) pristine MGSBS elastomer after electrical breakdown with the thickness of 510 μm and (right) the corresponding healed breakdown site before coating the flexible carbon grease on both sides for the actuation strain test. (C) Actuator device (left) with self-healed MGSBS elastomer after mechanical damage via scalpel cutting and (right) the electrical breakdown site after application of a voltage of 9.25 kV (18.1 kV mm^–1^). (D) Radial actuation strain of the pristine elastomer and healed elastomer after electrical, mechanical, or mechanical and electrical damage. All actuation measurements were conducted with a 33% biaxial prestrain on the elastomer. Adapted and modified with permission from ref (316). Copyright 2019 John Wiley and Sons.

Around five year later, in 2019, the group of Zhang et al. was the first one in reporting self-healing of both electrical breakdown and mechanical damage in dielectric actuators composed of a thermoplastic methyl thioglycolate-modified styrene–butadiene–styrene (MGSBS) dielectric elastomer (DE) (Figure 40A).^316^ They investigated the self-healing performance of the DE at both the microstructural and device levels by analyzing the healing mechanism of pinholes and by characterizing the electrical properties before and after self-healing. Also, the actuation and actuation efficiency of the dielectric actuators after being subjected to electrical and mechanical damage was investigated. The mechanism of self-healing was evaluated by subjecting the dielectric elastomer to dielectric breakdown and mechanical damage and studying the healing process by a combination of microscopy, electrical, and actuation measurements at low and high electric fields. To investigate the dielectric actuation performance, all polymers were coated with carbon black grease to form a circular electrode region (diameter 15 mm) from the center (Figures 40B,C). DC voltages were increased slowly from 0 to 10 kV to drive the actuation, and the actuation behavior was further monitored by a camera to estimate the voltage-induced planar deformation. In this case, the intermolecular electrostatic interactions between the methyl thioglycolate-modified butadiene block and the styrene block of SBS results in a dynamic interchain interaction over the damaged region of the elastomer and promotes healing (Figure 40A).^317,318^ Moreover, the dielectric strength can be recovered by ∼67% of initial strength after dielectric breakdown, by ∼39% after mechanical damage and by ∼33% after simultaneous electrical and mechanical damage (Figure 40D). Also, the DE exhibits high permittivity (ε′ > 10), high dielectric strength (*E*_b_ ≈ 30 kV mm–1), low Young’s modulus of 2.9 MPa, and a large strain to failure of 600%.

**Figure 41:**
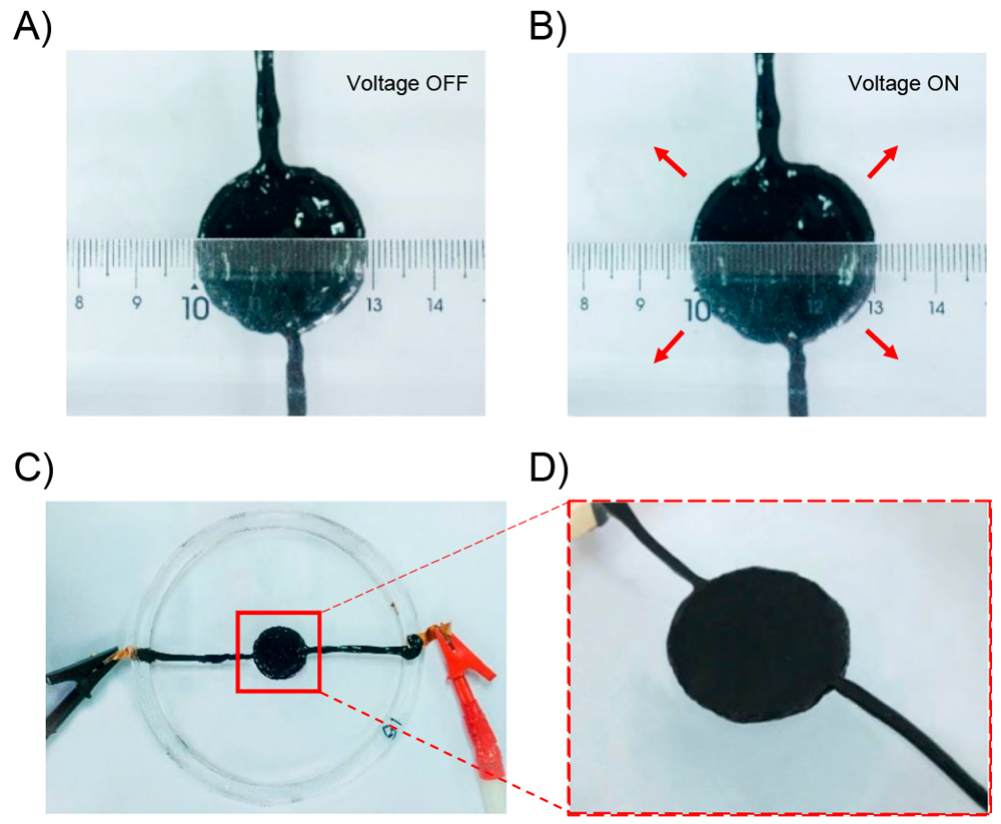
Off (A) and on (B) voltage-induced deformation of the active region of the DEA based on CNT/PVA hydrogel electrodes. (C) A photograph of circular DEA based on CNT/PVA hydrogel electrode. (D) A photograph showing the active area of the DEA. Adapted and modified with permission from ref (319). Copyright 2019 Royal Society of Chemistry.

The same year, Gao and collaborators fabricated a highly stretchable and self-healable ionic conductive electrode based on CNTs/PVA hydrogel to be used as compliant electrode in dielectric elastomer actuators (DEA).^319^ DEA are constructed by placing a soft dielectric polymer in between two compliant electrodes and are used to actuate soft robots.^79,320^ Note that the CNTs/PVA hydrogel electrode is highly stretchable (up to 200%) and conductive. The electrical conductivity of PVA modified by CNTs increases up to ∼0.71 S cm^–1^ compared to only PVA hydrogel. Upon subjecting the DEA to a high voltage, it reduces in thickness and expands in area due to the effect of Maxwell stress.^88^ In a CNTs/PVA hydrogel electrode containing DEA, the areal strain generated at an applied voltage of approximately 2 kV was more than 40%, which was almost two times larger than that based on pure PVA hydrogel electrode (Figure 41A,B). Moreover, the areal strain increases at higher applied voltages. Hydrogen bonding between the tetrafunctional borate ion and −OH group drives the spontaneous self-healing in the CNTs/PVA hydrogel electrodes. Experimentally, the CNT/PVA-based hydrogel electrode was fabricated as follows:^321^ To an aqueous solution of PVA and CNTs, sodium tetraborate was added to promote the cross-linking process. The DEA was constructed by inserting a prestretched (to a strain 200% × 200% in two directions) dielectric elastomer (i.e., VHB 4910) in between two CNTs/PVA hydrogels acting as electrodes. The thickness of the electrodes was 1 mm. The VHB was fixed to a rigid acrylic frame, while the two CNTs/PVA hydrogels electrodes were attached to the top and bottom surfaces of the elastomer. The active region of the DEA, with a diameter of approximately 3 cm, is shown in Figure 41A,B. The actuation was carried out using a high-voltage amplifier to actuate the DEAs. The real-time in-plane displacement of a thin paper tape, attached on the edge of electrode area of the DEAs, was measured continuously using a laser displacement sensor.

Areal strain (ε_area_) of the DEAs was calculated as follows:
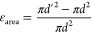
where *d* and *d*′ are the radii of the active region sandwiched by the electrodes at original and actuated state, respectively. On the other hand, the self-healing capability of CNTs/PVA hydrogel electrodes was demonstrated by performing a stretching test of the electrode after the healing process. Typically, two pieces of cut hydrogel were placed in contact with each other for approximately 1 min to self-heal. After that, the CNTs/PVA hydrogel could be stretched to the strain of ∼170% without having obvious structural fracture. This indicated that the CNTs/PVA hydrogel electrode was capable of large stretchability even after self-healing. Additionally, an electrical circuit containing a LED was connected to a power source using the CNTs/PVA hydrogel electrodes aiming to test the self-healing function. When one of the CNTs/PVA hydrogel electrode was cut, the LED turned off due to the open-circuit state. The LED was lit again when the two cut sections were kept in contact with each other, indicating self-healing without any assistance from some external stimuli.

**Figure 42:**
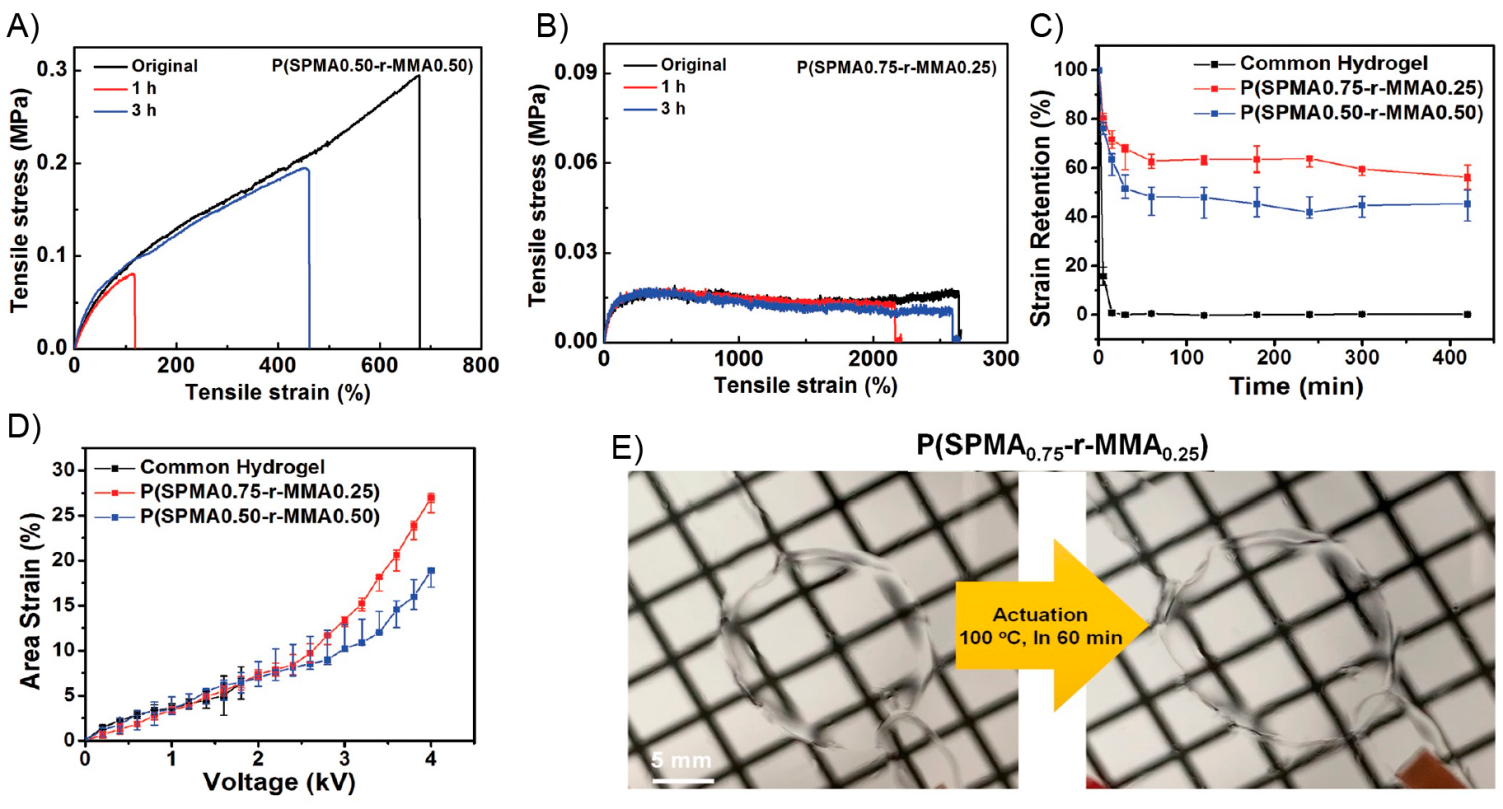
Tensile test at various healing times for (A) P(SPMA_0.75_-r-MMA_0.25_), and (B) P(SPMA_0.50_-r-MMA_0.50_). (C) Actuation efficiency of DEAs with P(SPMA_0.75_-r-MMA_0.25_), P(SPMA_0.50_-r-MMA_0.50_), and common hydrogel electrodes when subjected to a temperature of 100 °C for 420 min. DEAs were tested close to their breakdown voltages at which the DEAs with P(SPMA-r-MMA) electrodes were tested at 3.8 kV and DEAs with common hydrogel electrodes were tested at 2 kV (error bars with three devices). (D) Representative area strains of the DEA. (E) P(SPMA_0.75_-r-MMA_0.25_) electrodes before and after thermal aging for 60 min in the actuated state (voltage on). Adapted with permission from ref (324). Copyright 2019 John Wiley and Sons.

Stretchable electronics, which can recover its responsivity after severe wear and tear or any other mechanical damage, have attracted significant attention for the applications in biocompatible, wearable, and conformable devices.^322,323^ In this context, Lee and co-workers developed highly transparent, deformable, and self-healable thermal sensor gels P(SPMA-r-MMA) based on monomers (3-sulfopropyl methacrylate potassium salt (SPMA) and methyl methacrylate (MMA).^324^ Additionally, glycerol, as high boiling point solvent (290 °C) and showing excellent compatibility with the ionic chain, was used to prevent water evaporation from hydrogel and hence its stiffening.^325^ The ionic side chain in SPMA accounts for good conductivity, water solubility, and physical cross-linking to the polymers^326,327^ while the MMA imparts hydrophobicity, promoting polymer–polymer interactions. The self-healing capability of the thermal sensor was due to the strong electrostatic interactions in the ionic pendant group present in the gel structure. Electrostatic interactions promoted the reattachment of the cut gel surfaces when kept in contact with each other for 3 h, allowing the gel to be restretched adequately indicating recoverable self-healing properties. Tensile tests (stress–strain measurements) were performed to measure the break at elongation of the cut and healed gels in 1 and 3 h to account for the self-healing efficiency (Figure 42A,B). On the other hand, the MMA content in gels can be correlated to the mechanical properties, such as viscosity and elasticity. The dependence can be explained based on the strong polymer–polymer aggregation which resulted in physical cross-linking and entanglement within the ionic matrix. As a consequence of enhanced polymer–polymer aggregation, chain sliding was suppressed and the stretchability was decreased. To investigate the applicability of these P(SPMA-r-MMA) gels as ionic electrodes, a DEA composed of a dielectric elastomer sandwiched between two layers of ionic gels acting as conductors, was fabricated. Under an applied voltage, opposite charges begin to accumulate at the interfaces of the system producing columbic forces that compresses the dielectric elastomer along the thickness direction but expands in area. The actuation efficiency defined by the equation: strain retention = (*S*_t_ – *S*_0_)/*S*_0_ × 100%) was monitored at 100 °C over various time periods (Figure 42C), where *S*_0_ is the area strain at room temperature and *S*_t_ is the area strain at different thermal aging times at 100 °C. When aged at 100 °C for 15 min, DEAs with P(SPMA_0.75_-r-MMA_0.25_) and P(SPMA_0.50_-r-MMA_0.50_) electrodes showed an initial decrease in strain and then reached a plateau after 1 h while maintaining its transparent state throughout. These gels can find applicability as electrodes for high temperature applications, such as thermal sensors and soft robotics.^87^ It should be noted that the conductivities of PSPMA, P(SPMA_0.75_-r-MMA_0.25_), and P(SPMA_0.50_-r-MMA_0.50_) were found to be 9.8 × 10^–5^, 6.7 × 10^–4^, and 4.1 × 10^–4^ S cm^–1^, respectively. Among the three polymer gels, the P(SPMA_0.75_-r-MMA_0.25_) contains the right concentration of charge carriers, which does not disturb the movement of ions due to columbic interactions and shows the best conductivity compared to the other polymers. Also, the P(SPMA_0.75_-r-MMA_0.25_) gel showed high stretchability (2636% of break at elongation) and self-healing (98.3% in 3 h) properties. The degree of self-healing increases with increase in healing time, e.g., P(SPMA_0.75_-r-MMA_0.25_) gel shows 82.2% and 98.3% of healing efficiency in 1 and 3 h, respectively. At room temperature, P(SPMA_0.75_-r-MMA_0.25_) electrodes showed similar actuation strains as the control hydrogel used (Figure 42D), while the actuation area strain became higher when the strength of the electric field increased up to 4 kV. The absence of phase separation at high temperatures provided by the chemically bound ionic groups ensures the retention of the actuation efficiencies for P(SPMA_0.75_-r-MMA_0.25_), and P(SPMA_0.50_-r-MMA_0.50_) (62% and 46%, respectively, at 4 kV at 100 °C).

**Figure 43:**
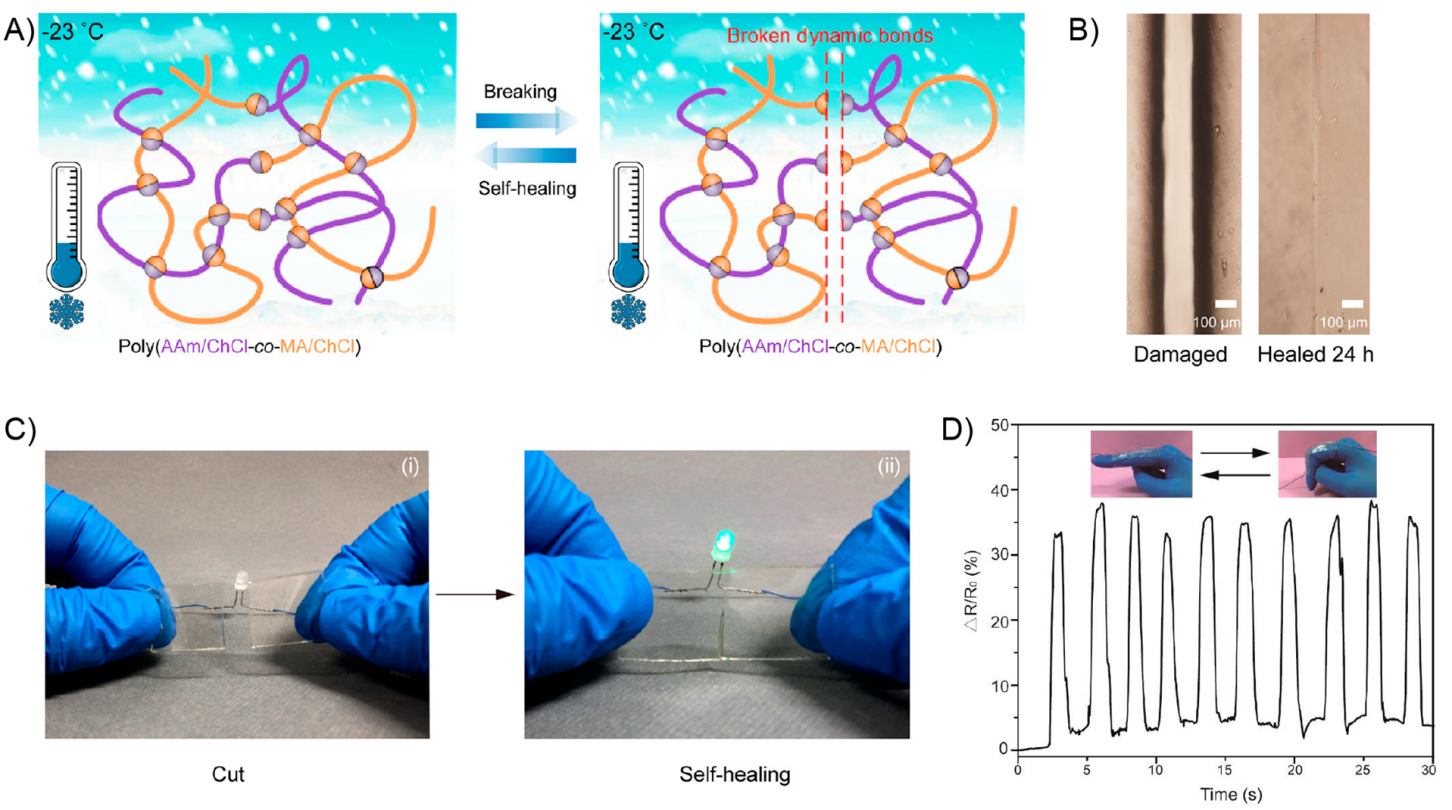
(A) Schematic representation of the healing process achieved in these autonomously self-healable, transparent, stretchable, and conducive elastomers. (B) Optical microscope images of a cut poly(AAm/ChCl-*co*-MA/ChCl) elastomer sample after healing at room temperature. C) Digital photographs of the healing process for a poly(AAm/ChCl-*co*-MA/ChCl) film in series with a LED. The LED could work once the two cut poly(AAm/ChCl-*co*-MA/ChCl) blocks were contacted together at room temperature. (D) Plots of resistance change of poly(AAm/ChCl-*co*-MA/ChCl) films as a function of time where a gentle motion of a finger could be monitor by upward and downward slopes of the relative resistance. Adapted with permission from ref (328). Copyright 2020 American Chemical Society.

Self-healable, transparent, conductive, and highly stretchable elastomers had been fabricated by Li et al. via a photoinitiated copolymerization of two polymerizable deep eutectic solvent (PDES) monomers–acrylic amide (AAm)/choline chloride (ChCl) and maleic acid (MA)/ChCl type PDESs.^328^ Acrylic acid (AA)/choline chloride (ChCl) type PDES based elastomer was previously found to be conductive, stretchable, and transparent but the challenge was self-repair due to lack of dynamic bonds.^329,330^ Moreover, conductive elastomers that can operate at extremely low temperature (−78 °C) but are unable to self-heal based on previous reports by Liu and co-workers.^331^ However, in this paper, dynamic hydrogen bonds between the building blocks of the poly(AAm/ChCl-*co*-MA/ChCl) system act as reversible cross-linking points, being able to spontaneously dissociate and reform imparting self-healing capability over a wide temperature range (−23 to 60 °C) (Figure 43A). In the field of stretchable electronics, transparency is very important.^332,333^ These elastomers demonstrate an ionic conductivity of 4.0 × 10^–4^ S cm^–1^, an average transmittance of 95.1%, high stretchability (strains up to 450%) at room temperature, and self-healing efficiency up to 94%. The elastomers were prepared by mixing two PDESs, AAm/ChCl and MA/ChCl, with MA/AAm at 1:1 mol ratio. Then, the PDES mixture was added to a cross-linker and a photoinitiator (1 mol % wrt comonomer), resulting in a transparent liquid precursor, which when cured under UV light for 5 min afford a transparent elastomer. To study the self-healing function of poly(AAm/ChCl-*co*-MA/ChCl) elastomers at temperatures below 0 °C, a film was cut into two pieces and then kept attached together below 0 °C to allow the cut heal. The self-healing capability was also studied quantitatively by calculating the self-healing efficiency calculated from the proportion of toughness restored relative to the original toughness. The self-healing studies demonstrated that the cut on the poly(AAm/ChCl-*co*-MA/ChCl) film had almost disappeared after healing for 24 h and only some minor scars remained. Figure 43B shows the optical microscope image of the damaged and the completely self-healed elastomer film after 24 h. Furthermore, it was observed that the increase in temperature and healing times resulted in a notable enhancement of the healing efficiency. Interestingly, when a damaged elastomer film was used in the elaboration of an electric circuit with a LED connected, the LED was lit immediately, indicating a rapid self-healing function and, therefore, the recovery of the electrical properties of the elastomeric film (Figure 43C). When integrated onto a volunteer’s finger or hindneck these autonomous self-healable and conductive elastomers could stretch and mimic the bending of human organs (Figure 43D). Moreover, the elastomers retained their ability to monitor the knee’s activities after being damaged by a knife. Although the work demonstrated that the reported conductive elastomers were able to self-repair over a wide temperature ranging from −23 to 60 °C, tensile tests revealed that the self-healing efficiencies are significantly lower at the lower end of the operating temperature as compared to that at the higher end.

Moved by the challenge of introducing self-healing features into soft dielectric actuators, Duan and collaborators described in 2020 an “all-polymer” self-healable actuator with electrical response based entirely on a polydimethylsiloxane/polyaniline system (PDMS-PANI*x*, being *x* the wt % of PANI content).^334^ In this sense, the authors demonstrated that by varying the amount of PANI, it was possible to tune the electrical properties from a material with insulating features at low PANI percentages (2.5 wt %) to a high conductivity system when the amount was increased up to 20 wt %. As a result, an integral actuator can be constructed employing the same class of materials as electrode and dielectric layers. Furthermore, due to the similar chemical nature shared between the electrodes and dielectric specimens, high compatibility was afforded during the device’s fabrication, being traduced in a system with good mechanical properties and an outstanding healing capability. Both, PDMS-PANI_20_ and PDMS-PANI_2.5_, were prepared through condensation reactions in which polydimethylsiloxane chains bearing anhydrides pendant groups reacted with bis-amino terminated polydimethylsiloxane chains and PANI doped with dodecylbenzenesulfonic acid. As authors mentioned, the basic requirements for the fabrication of a dielectric elastomer actuator are the presence of a dielectric layer with a high dielectric constant and low dielectric loss. In contrast, a material with high conductivity must be used as the electrode. Regarding the above, depending on the PANI content, samples with relevant differences not only in terms of dielectric property and electrical conductivity can be obtained but also with relevant variations in their stretchability and self-healing function. They conclude that the incorporation of a 2.5 wt % of PANI into PDMS allowed the preparation of a dielectric layer with a high dielectric constant (11.11 at 50 Hz), low dielectric loss (0.055 at 50 Hz), and adequate mechanical properties such as low Younǵs modulus (0.09 MPa) and high stretchability (3270%). They also found that a small increase of PANI (up to 5 wt %) elevates the dielectric constant but also induces an abrupt increase of the conductivity, indicating the formation of conductive channels across the sample.^335^ Indeed, higher contents of PANI notably enhanced the conductivity but at the expense of considerably sacrificing the stretchability of the material, therefore, as a conductive layer they decided to work with PDMS-PANI_20_, which stood out by a Young’s modulus of 0.47 MPa, a maximum stretchability of 450%, and a conductivity of 4.50 × 10^–5^ S/cm. The thermal properties of both materials revealed *T*_g_ values well below room temperature, ensuring a good fluidity of polymer chains at the normal working temperatures, and most importantly, allowing a good self-healing property. Initially, the authors tested the healing ability of these materials by scratching their surfaces. In the case of PDMS-PANI_2.5_, a complete healing process was achieved at room temperature, where no damage was remained after 6 h. However, PDMS-PANI_20_ showed a less efficient healability, in which, even after 48 h, traces of scar were still visible. The authors attributed this to a more restricted polymer chain mobility expected by the higher *T*_g_ value exhibited by this specimen. The outstanding self-healing capability of PDMS-PANI_2.5_ was also demonstrated by cutting a sample into two pieces and bringing them back together into contact for 1 h at room temperature, after which the healed sample was able to be stretched to 1000%. Overall, the calculated self-healing efficiency for PDMS-PANI_2.5_ was close to 98%, while for PDMS-PANI_20_ was lower than 85%. Notwithstanding the above, the healing ability of PDMS-PANI_20_ in terms of electrical conductivity recovery was enough, as was demonstrated by the authors after achieving to light a LED lamp a few minutes after starting the healing process of a damaged PDMS-PANI_20_ strip. The healing mechanism was attributed to a cooperation effect between the high mobility of polymer chains and the formation of a stable and reversible hydrogen bonding network that was corroborated by variable-temperature FTIR measurements.^336,337^ The remnant carboxylic acids coming from the hydrolysis of unreacted anhydrides, along with the amide entities, would be the main ones involved in the formation of this hydrogen bonding network. Fortunately, the self-healing property was successfully transferred once PDMS-PANI_20_ and PDMS-PANI_2.5_ were assembled into an actuator configuration. Indeed, SEM analysis revealed a highly interpenetrated interface between the dielectric and electrode layers, accusing good compatibility that should enhance the self-healing process across the whole system. As was expected, the assemble showed a proper actuation property under increasing electric fields, evidenced by the change of the area strain of a circular specimen. Regarding the above, actuated strain values above 7% were achieved after the application of electric field values higher than 15 V/μm. Finally, the authors evaluated the actuation performance of a sample healed during 48 h, showing that the material still exhibited actuation property after damage; however, it must be mentioned that the actuation capacity was notably diminished (1.62% at 15.8 V/μm). They also demonstrated that even after a breakdown failure, after several hours of healing the specimen still exhibited deformation motions. Moreover, the system showed an excellent cycling performance as after four consecutive cycles, no evident decay of the actuation stability was observed for both pristine and healed samples. This study can be considered pioneering because it achieved the preparation of the first dielectric soft actuator exhibiting integrally self-healing capability, as well as one of the first attempts to focus attention on the self-healing property of the electrode.

**Figure 44:**
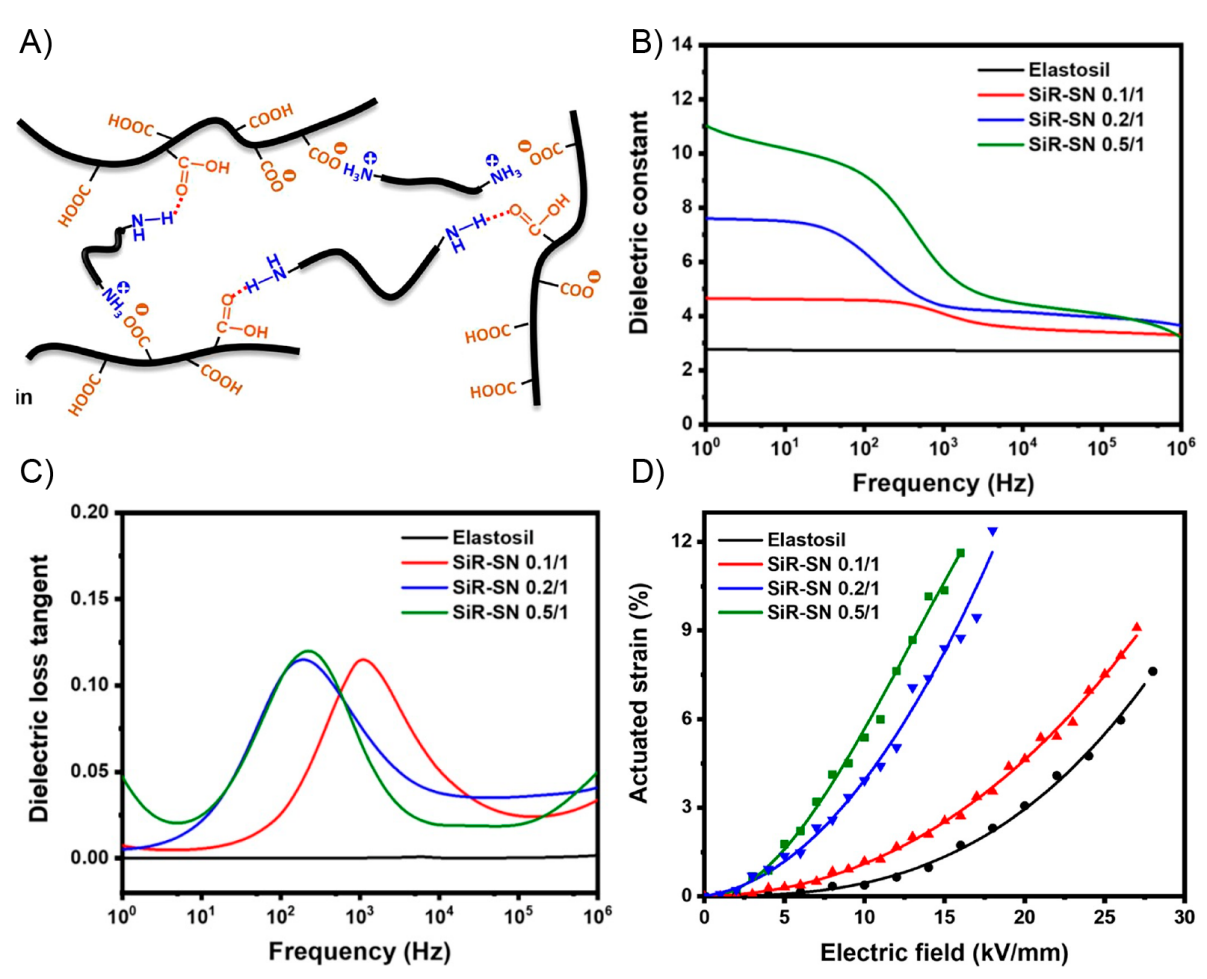
(A) Schematic showing the interactions leading to the formation of SiR-SN, (B) dielectric constant, (C) dielectric loss tangent versus frequency of reference Elastosil and SiR-SN, and (D) Actuated strain of reference Elastosil and SiR-SN. Adapted with permission from ref (338). Copyright 2020 Elsevier.

Sun and co-workers developed a self-healable silicon dielectric elastomer (SiR-SN) by blending a carboxyl terminated poly(methylvinylsiloxane) (PMS-*g*-COOH) and an amino terminated poly(dimethylsiloxane) (PDMS-NH_2_) into a supramolecular network (SN).^338^ The dielectric constant (ε′) of SiR-SN was high due to the polar PMS-*g*-COOH component and resulted in generation of high actuated strain under a low electric field, a property that is required for any dielectric elastomer to function as “artificial muscle”.^339,340^ Moreover, the noncovalent bonds such as hydrogen and ionic bonds between the two components contributes to the self-healing ability of the supramolecular network (Figure 44A). The DE films, SiR-SN, were prepared by mixing PDMS-NH_2_ with PMS-*g*-COOH through solution blending and casting the viscous white solution on a Teflon mold. After allowing solvent evaporation and film formation at room temperature for 3 days, the prepared elastomer film was kept in an oven at 60 °C for 72 h. The elastomeric network was formed as result of ionic bonds between COO^–^ and NH_3_^+^, which were generated by the deprotonation of COOH on the side chains of PMS-*g*-COOH and protonation of NH_2_ at the chain end of PDMS-NH_2_ and hydrogen bonds between the carbonyl groups (C=O) and the amino groups (NH_2_).^341,342^ The actuated strain of the DE film was measured by a circular strain test, whereby the film was fixed on a circle frame and the change in the pixel of the electrodes’ area divided by the original pixel area was calculated to determine the strain. The self-healing ability of the DE film was analyzed by determining the tensile properties of the thin films before and after self-healing. Moreover, actuated strain of the film after self-healing was also measured to study the self-healability. To perform the experiment, samples were cut into two halves and the fractured surfaces were attached together, kept in an oven for 5 h at 80 °C or 1 h at 100 °C, and retested for actuated strain. Due to the polar nature of SiR-SN, the ε′ decreases with increasing frequency and attains a constant value at higher frequencies. The ε′ at 10^3^ Hz was significantly enhanced from 4.1 for SiR-SN 0.1/1 to 5.5 for SiR-SN 0.5/1 because with the increase in PMS-*g*-COOH content in the DE films, the dipole content increases and thus the polarization of SiR-SN is enhanced (Figure 44B). It is important to note that compared with Elastosil, a commercial silicone with good actuation performance,^343^ both ε′ and dielectric loss tangent (tan δ) of SiR-SN was much higher (Figure 44C). It was observed that at 80 °C self-healing of SiR-SN is due to the reformation of hydrogen bonds, while at 100 °C, self-healing was due to the transition into ionic bonds from hydrogen bonds. Thus, the recovery of network structure and a change in network structure take place at 80 and 100 °C, respectively. Consequently, a self-healing efficiency of 115% in tensile strength and 100% in actuated strain (*S*_A_) at a given electric field was achieved after self-healing at 80 °C for 5 h, whereas an increase in elastic modulus and higher breakdown strength was achieved after self-healing at 100 °C. Regarding the actuation function of these samples, Figure 44D displays that for all samples, *S*_A_ values increase with the strength of the electric field because of the quadratic relationship existing between both parameters.^344^ The maximum *S*_A_ at given electric field increase as the content of PMS-*g*-COOH increased (Figure 44D). In this regard, a jump from 8.8% to 11.6% was observed for *S*_A_ values of SiR-SN 0.1/1 and SiR-SN 0.5/1, respectively. Moreover, similar trends for *S*_A_ were observed at low electrical fields (15 kV/mm), going from 2.5% for SiR-SN 0.1/1 to 10.7% for SiR-SN 0.5/1. This behavior was ascribed to the simultaneous increase in ε′ and elastic modulus in samples with higher content of PMS-*g*-COOH. The outstanding *S*_A_ values achieved by these samples were supported based on the fact that most silicone-based DE reported in literature shown values below 4% at 15 kV/mm.

At the beginning of 2022, Nie and co-workers came up with a facile and effective methodology through which a novel self-healable dielectric elastomer was prepared, exhibiting high stretchability, good dielectric properties, large actuation, and an outstanding self-repair ability against mechanical and electrical damage.^345^ The dielectric elastomeric system was achieved by blending poly(vinylidene fluoride-*co*-hexafluoropropylene) (P(VDF-HFP)) with increasing quantities (up to 20 wt %) of FS30, a well-known fluorosurfactant. The morphology, chemical structure and composition was successfully confirmed by means of SEM, FTIR, and elemental mapping. Surprisingly, and contrary to expectations, DSC analysis showed that *T*_g_ values for all materials fell around −20 °C despite the increasing amounts of FS30. Authors argued this in terms of the multiple and strong hydrogen bonds and dipole–dipole interactions due to the abundance of CF_3_ structures in the material, which were also responsible of the adequate dielectric properties exhibited by these systems. Regarding the last, all prepared samples showed dielectric constants values between 11.2 ± 0.3 and 10.7 ± 0.3 (at 1 kHz), being the highest value assigned to the sample with the lowest FS30 composition (6 wt %), while at the same time unnoticeable diminishing of dielectric breakdown values was observed. In addition, the homogeneous surfactant distribution achieved during specimen preparation endowed them with good mechanical properties. All films displayed high stretchability with elongation at break values above 2000% but a decrease of Young’s modulus as the surfactant composition increased. As a result, thanks to the incorporation of FS30, the sensitivity of the elastomer to electric fields, along with their electromechanical properties, improved notably. After confirming the suitable dielectric and mechanical properties displayed by these elastomeric materials, the authors attempted the preparation of an actuator system. Based on the above, a bending actuator was constructed by depositing a P(VDF-HFP)/FS30 sample coated on one side with a gold layer (acting as an electrode) over an inextensible PI film. This configuration imposes a restriction to the lateral expansion of the dielectric elastomeric layer, allowing the conversion of electrical energy into mechanical bending deformation. Then, by measuring the actuation performance in terms of angle deformation, the authors demonstrated that P(VDF-HFP)/FS30 systems achieved higher bending angles than an actuator fabricated solely with P(VDF-HFP). Specifically, the P(VDF-HFP)/FS30 based actuator reached values of bending angles of 13.8° and 29.5° at electric fields of 20 and 30 MV/m, respectively, representing an increase of 33% and 19% regarding the performance of the actuator without fluorosurfactant. By last, all samples exhibited a proper self-healing property that took place successfully at room temperature. As example, a surface damaged sample revealed only a small scar after 24 h of healing at room temperature, and authors also demonstrated that by applying pressure and also increasing the temperature over the affected zone, the healing process could be accelerated. Overall, after 24 h of healing, specimens nearly recovered their initial state, in which the healed sample was able to hold a weight 750 times its own weight. On the other hand, tensile tests showing that while pure P(VDF-HFP) was merely able to recover 17% of the initial elongation at break value, a sample containing FS30 surfactant reached values between 607% and 750%. The authors explain the outstanding self-healing property in terms of three consecutive procedures: contact, diffusion, and homogenization, which were assisted by the low *T*_g_ value and the existence of hydroxyl and CF_3_ structures favoring the existence of multiple and reversible hydrogen bonds and dipolar–dipolar interactions. Fortunately, the healing property was also able to fix electrical damage, showing that the healed samples still exhibited actuation motions in two different formats, bending and diaphragm actuator devices. In fact, despite the irreversible damage, the actuation motions were still comparable to that of the pristine sample. Moreover, the bending angle values revealed outstanding stability during cyclic actuation tests, achieving the recovery of the original values even after five dielectric breakdown cycles. This work represents a novel route to prepare reliable elastomer actuators with electrical responsiveness, providing a long service time and with potential application in fields such as wearable electronics and soft robotics.

Almost parallel to above-mentioned Nie’s work, Liu and co-workers prepared stretchable and self-healable ionic electroactive polymers (PAST-iEAPs) based on a cross-linked hydrogel composed of poly(acrylic acid/2-acrylamide-2-methylpropanesulfonic acid) (PAA/AMPS) and gelatinized cassava starch.^346^ The effect of different conductive substances, such as MWCNTs aqueous dispersion, reduced graphene oxide (rGO) dispersion, NaCl, and CaCl_2_ on the self-healability and the electromechanical performance had been studied. The functionalized PAST-iEAPs demonstrated excellent tensile, electrochemical, and electromechanical properties, as well as good self-healing ability and can be used in soft robots, flexible actuators, and medical devices. Because the actuation in electric field-responsive materials can be controlled remotely, they are generally used in soft robots and actuators.^347,348^ Previous literature reports suggested that PAA/AMPS hydrogels showed good mechanical and electrical response properties but weak self-healing capability, which made self-repair after being damaged impossible.^349,350^ The PAST-iEAPS were prepared by cross-linking a mixture of gelatinized cassava starch (CST), acrylic acid (AA), and 2-acrylamide-2-methyl-1-propanesulfonic acid (AMPS), which was neutralized with 20 wt % NaOH, using *N*-methylenebis(acrylamide) (MBA) as the cross-linking agent. To prepare the electrode surface layer, first, chitosan (CS) was dissolved in acetic acid (HAc). Then, MWCNT, rGO, and PANI were successively added to the solution along with a few drops of glycerol and stirred for 5 min. The resulting solution was coated on the surface of PAST-iEAPs. The hydroxyl groups in CST formed a large number of dynamic physical bonds in the matrix of PAST-iEAPs, resulting in a chemical–physical cross-linked double network structure.^351^ The electro-actuation of the PAST-iEAPs was controlled by the movement of ions under the action of the electric field, which resulted in a gradient difference of ion concentration. The free moving cations (e.g., H^+^, Na^+^) and anions (e.g., OH^–^) dissolved in polar solvents were produced by hydrolysis. When the electric field was applied on PAST-iEAPs, the cations and anions moved in the opposite direction of the cathode and anode, respectively, resulting in good electrical properties. The self-healing mechanism of PAST-iEAPs can be related to cassava starch. The numerous hydroxyl groups and heavily branched high-amylopectin starch chains at the cutting interface promote self-healing through rearrangement and reconstruction of noncovalent interactions such as hydrogen bonding, chain entanglement, and hydrophobic interaction.^352^ The modified PAST-iEAPs demonstrated good tensile strength; PAST-iEAPs functionalized with rGO had maximum tensile stress of 1.449 MPa, which was 2.5 times higher than the original PAST-iEAP, while the maximum tensile stress of PAST-iEAP functionalized with Ca^2+^ and MWCNT was about 1.8 times higher than the unmodified PAST-iEAPs. On the contrary, PAST-iEAP functionalized with NaCl demonstrated weaker strength, toughness, tensile properties, and low self-healing capability. Additionally, the modified PAST-iEAPs had an excellent electromechanical performance, especially, PAST-iEAPs functionalized with rGO and MWCNT could maintain good response speed in the first 30 s. Of these, PAST-iEAP functionalized with rGO had higher maximum output force of 38.326 mN, about 2.1 times higher than that of the unmodified PAST-iEAP. The PAST-iEAP functionalized with NaCl demonstrated maximum deflection angle of 128° at 10 V DC voltage, which is about 1.8 times higher than original PAST-iEAPs. The self-healing efficiency of the original PAST-iEAPs was about 92% after 24 h at room temperature. Furthermore, the self-healing efficiencies of PAST-iEAPs functionalized with rGO, CaCl_2_, and NaCl were 96.44%, 94.95%, and 85.45%, respectively.

In less than two months, Nie and collaborators contributed again with the preparation of a new dielectric elastomer actuator displaying a rapid self-healing capability at room temperature, but in this opportunity employing a much more challenging chemistry than in their previous contribution.^353^ In this sense, the dielectric elastomer was classified as a supramolecular system based on poly(urethane-urea) structures, where the hard segments were responsible for the dielectric and self-healing properties, while the soft segments endowed the material with high stretchability. As authors stated, the key to the outstanding performance showed by this system was the use of dynamic covalent chemistry tools, where, in addition to the already existing hydrogen bonds, they managed the incorporation of disulfide linkages. Thus, the complementarity between disulfide metathesis reactions and the multiple/reversible hydrogen bonds allowed the obtention of a material exhibiting a fast and efficient healing property. By means of FTIR, the authors corroborated the presence of hydrogen bonds between urea and urethane moieties, while through Raman spectroscopy they proved the correct incorporation of disulfide bonds. Moreover, wide-angle X-ray scattering (WAXD) and DSC analysis accused the formation of a complete amorphous material, offering a suitable environment to maintain the dynamic nature of the elastomeric network. Indeed, tan(δ) profiles acquired through DMA showed a glass transition temperature around −60.5 °C for the system having disulfide bonds, favoring a high chain mobility at room temperature that would also allow to increase the efficiency of the healing process. In terms of mechanical properties, the samples stood out by displaying a high stretchability of around 10 times their initial length (elongation = 1068%) and low Young’s modulus (1.76 MPa). The high stretchability was ascribed to the presence of PTMG structures acting as soft segments in the structure, along with multiple hydrogen bonding interactions. On the other hand, the low Young’s modulus was attributed to the S–S exchange reaction that reduces the capability of the material to resist mechanical deformation.^354^ Regarding the dielectric properties of the system, a high dielectric constant of 10.9 at 1 kHz was determined. This increased value was explained by author through two main contributions: (1) the high dipole mobility at room temperature due to the low *T*_g_ of the system and (2) an inherent increased polarity of the system related to the presence of highly polar urethane and urea functionalities.^355^ Then, the actuation performance was evaluated, first, by disposing the material into a circular actuator configuration, facilitating the measurement of the area strain increment as the electric field rose up to the electrical breakdown. It worth noting that the sample containing disulfide bonds exhibited a noticeably higher strain than a reference sample without this type of linkage (18.2% vs 4.6% at 60 MV/m), being around 4 times higher at the same external electrical field. Indeed, based on the authors comments, the actuation area strain displayed by this material notably surpasses those previously reported elastomeric actuators based on polyurethane systems.^356−361^ Then, they carried out the construction of a bending actuator following the same protocol as their previous report.^353^ Surprisingly, the elastomeric actuator having S–S linkages exhibited an improved actuation property characterized by an increase in bending angle value from 1.4° to 25.9° as the electric field increased from 10 MV/m to 30 MV/m. The performance observed in this material was comparable to other electroactive polymers, for example, those referred to as relaxor ferroelectrics.^362^ In terms of self-healing properties, specimens stood out by displaying a fast and excellent healing capability triggered without any external stimuli, ascribed to the multiple and reversible hydrogen bond formation, along with the metathesis disulfide reaction that allows the reformation of chemical bonds after damage. The healing process was first tested by cutting a sample into two halves and then bringing back together by applying a slight pressure. As a result, the healed sample could hold a structure 1500 times greater than its own weight. In addition, the OM and SEM analysis revealed that the initial damage ended as a slight cut trace. Tensile tests also allowed the evaluation of the healing property, showing that after 1 h of incubation at room temperature, the healed sample could be stretched up to 324 ± 56%, representing a healing efficiency of around 30%. Notwithstanding the above, when the healing time was increased to 3 h, the process reached an efficiency of 96%. Interestingly, the authors also evaluated the surfaces aging resistance and demonstrate that light irradiation promote the healing process by favoring the exchange of disulfide bonds. Last, they also corroborated that the healing property can be successfully transferred to an actuator device, where, after damaging the system and letting the healing process to proceed at room temperature for 20 min, they found that the actuation motion of the healed sample was completely recovered regarding the performance of the original sample. The authors propose this type of system to be potentially applied as electric-field-activated actuators in high voltage devices, such as “high-voltage alarm” as a switch apparatus.

To the best of our knowledge, the last report involving the development of a dielectric elastomer with self-healing properties belongs to Szczepanski and co-workers, which, starting from cyclic siloxanes bearing nitriles as dipolar pendant groups achieved the synthesis of silicone-based elastomers with high and modulable permittivity and self-healing capability, employing a simple one-step methodology through anionic ring-opening polymerization.^363^ At the end of the process, the equilibrated macromolecular network, it is composed of linear chains and cyclic compounds covalently attached, whose thermal and mechanical properties showed to be highly dependent on the cross-linking density, as well as the amount of dipolar structures. Regarding mechanical properties, tensile tests revealed that an increment of the cross-linker composition in the formulation led to a depletion of the elongation at break value, falling from 59 ± 3% to 41 ± 3%, while at the same time a rise of the Young’s modulus up to 202 ± 10 kPa was achieved. Aiming to improve the strain at break values, the authors carried out the preparation of these materials decreasing the composition of cyanopropyl groups, which was achieved by replacing 50% of the initial dipolar monomer content with nonpolar monomer. This strategy turned out to be surprisingly effective, where higher Young’s modulus values were achieved along with higher elongation at break. The authors attributed the improvement of both parameters to a better compatibility between the nonpolar cross-linker and nonpolar monomer included in the new formulation, promoting the formation of a more robust elastomeric network. Then, through temperature-dependent DMA measurements, the authors proved the thermoreversible nature of the “living” polymer network, showing that upon heating and subsequent cooling, the initial mechanical properties could be restored. In this regard, the self-healing capability showed by these materials and the possibility to be reprocessed opens new strategies to develop novel robust dielectric elastomer actuators. To test the self-healing property, samples were cut with a blade, and after 10 min of heating at 80 °C the original mechanical properties were effectively restored. As the authors explained, the healing property is attribute to the “living” nature of the network, represented by the active silanolate end groups in charge of the self-healing process, which takes place through reversible covalent bonds. Thus, heating the specimen promotes its partial depolymerization, becoming a viscous-like system that, thanks to the increased mobility of polymer chains, facilitates the action of the terminal groups and, thereby, of the self-healing process. Moreover, the authors carried out the reshaping of the healed elastomer three times, maintaining the mechanical properties only slightly affected. The resulting film was able to be stretched without showing glimpses of fracture in the healed area. In addition, the authors also demonstrated that the healing process take place in the material when it is in a relaxed state (without applying pressure) and also that a lower cross-linking density allows lowering the temperature necessary to promote self-healing. In terms of electric properties, all specimens showed high conductivity values and dielectric constants between 16.9 ± 0.3 and 18.1 ± 0.1 at 100 kHz for those samples having the highest amount of dipole entities. On the contrary, samples prepared with 50% of nonpolar monomer exhibited a lower dielectric constant of around 12.7 due to a diminishing of the dipolar density. In addition, the authors also notice that an increment on the number of dipoles restricts at some extent the mobility of chain segments, as was corroborated by the increase of *T*_g_ values. Finally, the authors achieved the fabrication of an actuator device using samples in film format. In this sense, one of the achieved systems displayed a lateral strain actuation of 3.8% at 5.2 V/μm (low voltage), which remained stable for over 100 cycles. Additionally, the actuator was able to self-repair after suffering electrical breakdown, showing almost an exact actuation property in the next cycle. However, under more severe damage caused by repetitive electrical breakdowns, specimens could not fully recover their initial actuation performance. Then, motivated by the good results, the authors carried out the fabrication of stack actuators. As a result, they successfully achieved the preparation of a device that, as actuation motion, showed an average thickness change of 56 ± 2 μm related with an actuation of 5.4 ± 0.2% at 3.2 V/μm considered as a very low electric field. Additionally, by preparing devices with a greater number of layers, a higher actuation property was achieved. However, it must be mentioned that this actuation showed a depletion after several cycles. Although, it must be mentioned that this actuation showed a strain depletion after several cycles. Notwithstanding the above, this work can be considered as the first report on stack actuators prepared from self-healable polysiloxanes presenting high permittivity as dielectric layers. The authors propose these systems as promising materials for artificial muscles, among other applications, declaring that the next step of this research would be printing these materials in new prototype devices.

### 2.6 Self-Healing pH, Mechanical, and Redox Actuators

**Figure 45:**
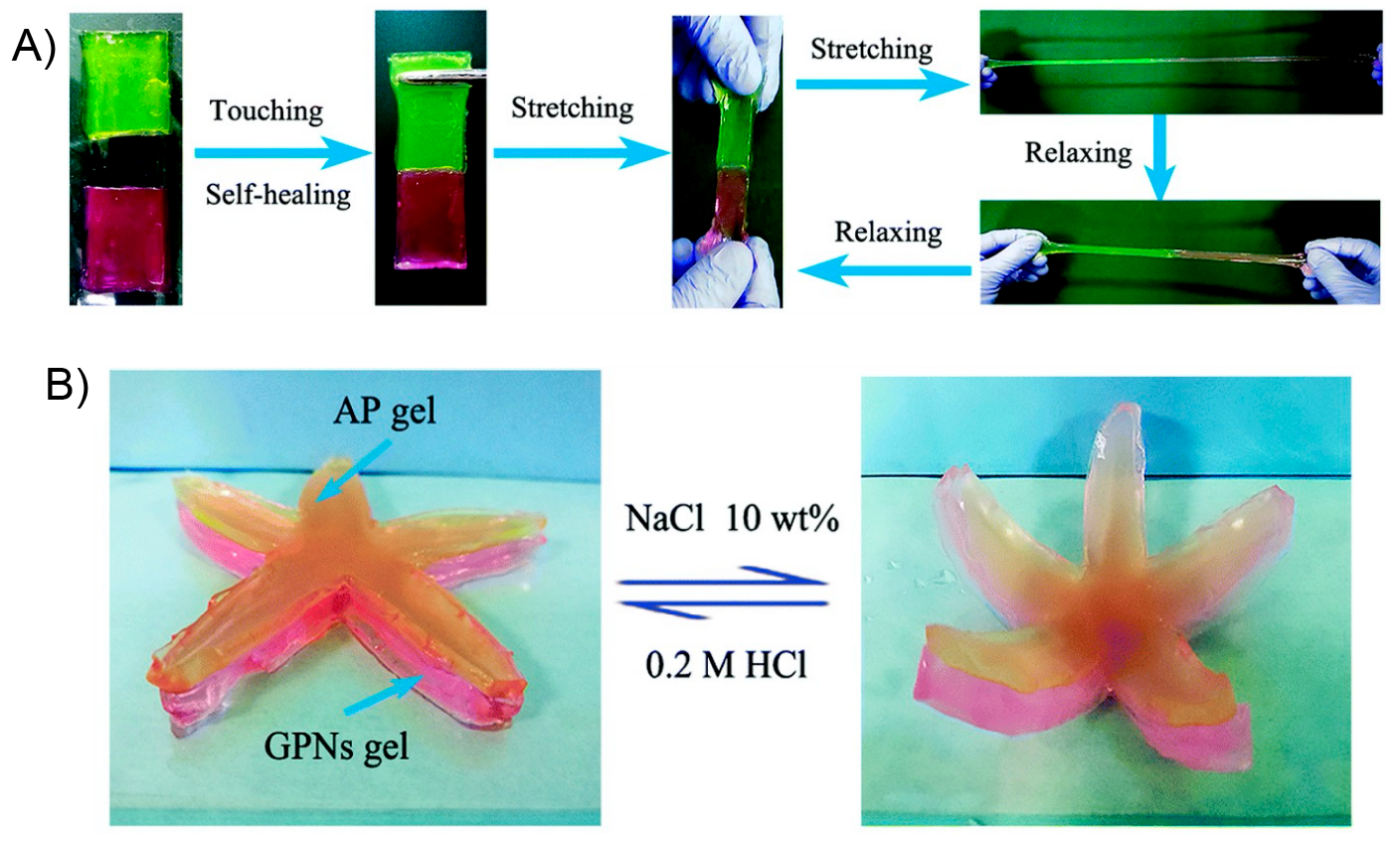
(A) Schematic illustration of the recoverable and self-healing process of the GPNs gel. After touching for a few minutes, the two pieces of the cutting hydrogels demonstrated fast healing and presented excellent tensile strength and restorability. (B) Photographs of the reversible actuation of the GPNs/NS bilayer hydrogel in NaCl and HCl solutions. Adapted and modified with permission from ref (364). Copyright 2020 Royal Society of Chemistry.

At the beginning of 2020, Yi Zhu and co-workers designed a smart gelatin/PAAm/clay ionic hydrogel (GPN gel) that achieved the synergistic characteristics of excellent mechanical properties, robust adhesion, excellent self-healing function, and ion-driven/pH-driven response to stimuli.^364^ GPN gels were designed by constructing a dual network with intercalated charged nanosheets. First, gelatin (1–5 wt %) was thermally dissolved in water, followed by the addition of acrylic amide (AAm, 20 wt %), *N*,*N*-methylenebis(acrylamide) (BIS) (0.1 wt % of AAm) as a cross-linker, a suspension of clay (0–20% by weight) and UV initiators (0.9% v/w of AAm). Finally, the resulting formulation was poured onto a template and subjected to UV radiation (λ = 365 nm, 96 W) for 20 min, achieving the formation of transparent gels. Based on the above, a dual network structure was built through the interpenetration of gelatin and PAAm chains. Moreover, Clay nanosheets were homogeneously dispersed within the gel network acting as multifunctional cross-linkers, connecting polymer chains through electrostatic interactions, which significantly increased the mechanical characteristics, adhesion, and other properties of the GPN hydrogel.^365−368^ The self-healing property of the gel was measured through a simple process, where two cut pieces were brought into close contact for a certain amount of time. The curing efficiency was evaluated in terms of the tensile strength values recovered from the gels after exposure to different healing times. The two cut pieces healed quickly and effectively, showing that the healed sample could be stretched to more than six times its original length and returned to its initial configuration in a short time when the force was removed (Figure 45A). Healing efficiencies of around 80% were achieved when samples were allowed to heal for 60 min. The authors attributed the healing mechanism to the efficient reformation of hydrogen bonds and re-entanglements between polymer chains, allowing the successful reparation of the network. The GPN gel exhibited multiple sensitive responses (including salt, pH, and stress ions) due to the negatively charged clay nanosheets and the ampholytic property of gelatin. Concerning the above, the GPN gels showed an adequate swelling property even in saline solutions, which would be attributed to the electrostatic interactions that occur between Na ions (from NaCl solution) and the negatively charged groups present in both clay and gelatin chemical structures, which contributed to the existence of a higher ionic concentration in the inner structure of the gel compared to the outer solution. Based on this, the authors designed a simple NaCl-sensitive actuator by fabricating a bilayered gel material using PAAm gel and agar (AP), inspired by previously reported results.^369−372^ The bilayer sample was molded into a flower shape to test their salt/pH-driven actuation function (Figure 45B). When this sample was immersed in a 10 wt % NaCl solution, the PAAm gel layer exhibited a fast swelling process. In contrast, the AP gel shrank rapidly due to the high ion concentration in the solution. Thus, the hydrogel flower folded toward the AP gel side, presenting a petal closing process. In contrast, the GPNs gel and the AP gel showed the opposite swelling tendency when the closed hydrogel flower was immersed in HCl solution, resulting in a return to the original state. This unique performance of the bilayer hydrogel would be benefited from the opposite swelling behavior presented by the PAAm and AP in the presence of salt ions and pH variations.

Regarding mechanical stimuli, Sitti and co-workers introduced high-strength synthetic proteins that self-heal almost instantaneously by applying local heating after micro- and macroscale mechanical damage.^373^ The authors optimized these materials in order to improve their hydrogen-bonded nanostructure and network morphology, affording programmable healing properties (i.e., 2–23 MPa strength after 1 s of healing) that exceed by several orders of magnitude those of other natural and synthetic soft materials. Experimentally, biosynthetic proteins with *n* = 4, 7, 11, and 25 tandem repetitions (TRs) of a squid-inspired building block (TRn4, TRn7, TRn11, and TRn25, with molecular weights of 15.8, 25.7, 39.4, and 84.6 kDa, respectively) were synthesized, covering peptide lengths usually found in native proteins. In these systems, the tandem repeat polypeptides self-assemble into supramolecular β-sheet-stabilized networks, where the network morphology arise from the amorphous phase composed of flexible chains. In this field, it is important to highlight that potential molecular defects of protein networks such as dangling ends or loops can degrade the material properties.^374^ Thus, in order to quantify these defects, a network parameter ε_eff_ = 1 – (β_c_/*n*), in which β_c_ is the β-sheet crystallite size (average of 4 β-strands) and *n* is the tandem repetition of the main building block, is used to define the effective strand density (e.g., effective connections between cross-linking points) from zero (defective network) to unity (perfect network).^375^ The results obtained by these authors demonstrated that the molecular defect density from “all-defective” networks (TRn4) to “close-to-perfect” networks (TRn25) could be tailored systematically by controlling tandem repetition accurately in the polypeptides. The practical importance of such control of the molecular defects is that the properties can be programmed by sequence design and yield protein materials with remarkable self-healing capacity.^376,377^ To visualize the self-healing process, protein-coated substrates were laser-micromachined to simulate a scratch defect pattern and were locally (half defect) and fully (complete defect) healed for 2 s sequentially. Importantly, after the short healing process, the damaged protein recovered its conformal surface coverage. In addition, the authors punctured a free-standing, flexible TRn11 protein film (50 μm thick) to create a hole defect that was subsequently healed in less than 1 s by local heating.^378^ Although the size of the damage area and viscoelasticity can be a limitation in intrinsic self-healing mechanisms, it can be overcome by patching with the incorporation of new protein material. Moreover, free-standing films of cut-damaged protein were found to heal also within 1 s, recovering their elastomeric properties (i.e., stretching deformations >200% strain and posthealing strength of up to 23 MPa).^122^ From a mechanistic point of view, owing to the noncovalent nature of the interactions involved in these protein networks, they can be repaired quickly after damage with physical cross-links through chain diffusion within the protein matrix and β-sheet nanostructures. Such chain diffusion and network repair are facilitated by the presence of water that acts as plasticizer. Indeed, hydration of the material decreases the glass transition temperature, which enables a stable rubbery state over a wide temperature range including room temperature.^379^ In terms of thermal stimuli, as temperature increases, so does the protein chain mobility,^380^ which softens the material and facilitates the fast diffusion of chain segments across the separated parts. Therefore, although healing occurs already at room temperature, temperature regulation offers in these materials a versatile control over the healing kinetics. In order to explore the use of self-healing bioinspired tandem repeat proteins in soft actuators, the authors built a pneumatic actuator^381,382^ consisting of two protein disc membranes joined together and connected to a circuit. In this case, when the actuator is pressurized, the soft TRn11 protein membranes deform and, in consequence, the actuator expands in volume, causing a deformation and a force output. Next, using the same protein soft actuator design, the authors succeeded in the fabrication of an artificial muscle capable of lifting repeatedly a dead weight at least 3000 times heavier than its own mass, displaying a specific work of 215 J kg^–1^, an average specific power of 488 W kg^–1^, and a thermodynamic efficiency of 62%, which exceed those of biological muscle and are comparable to other soft actuators.^383−385^ Last but not least, tandem repeat polypeptide actuators are also degraded at demand, taking advantage of the protein noncovalently cross-linked network and the fast dissolution of the protein actuator at acidic pH (i.e., acetic acid, 5% v/v), which breaks the β-sheet nanostructures.^386^

Very recently, Wang et al. reported a PEG-based polymer gel system prepared by UV-induced one-pot sequential polymerization and composed of a poly(hydroxyethyl methacrylate-*co*-acrylic acid) (P(HEMA-*co*-AAc)) network and small- to average-molecular-weight PEG.^387^ The use of macromolecular nonvolatile PEG as the liquid phase resulted in a PEG gel with exceptional physical properties, such as high stretchability (i.e., 6000% at 50 mm min^–1^) and toughness, transparency (95% for visible light), rapid self-healing (1 min), 3D printability, and long-term stability under ambient conditions as compared to the corresponding hydrogel generated using water or ethylene glycol as the liquid phase. Depending on the molecular weight and volume fraction of PEG, the tensile strength of PEG-based polymer gels varied from 0.22 to 41.3 MPa, fracture strain from 12% to 4336%, modulus from 0.08 to 352 MPa, and toughness from 2.89 to 56.23 MJ m^–3^. Importantly, these materials demonstrated self-healing capacity based on the diffusibility/mobility of small-molecular weight PEG molecules, which wrapped around the polymer network, the mobility of the polymer network, and the multiple weak C–H····O hydrogen bonding between the PEG and the polymer network, which acted as the dynamic cross-linkers. Each PEG molecule formed H-bonds at both ends to connect different sites of a single P(HEMA-*co*-AAc) chain (polymer–solvent–polymer cross-links) effectively introducing long-range correlations and enhancing compaction of the polymer coil. With this system, the authors fabricated a pneumatic PEG gel soft actuator, with a hollow structure with asymmetry above and below, through DLP 3D-printing technology. When the actuator was pressurized, the sawtooth part expanded and deformed toward the nonsawtooth side, causing a directed force that served as an actuator. The actuator bent under pressure (0.5 MPa) and recovered after the pressure was released. Because the pneumatic actuators are vulnerable to puncture damage, the self-healing properties of the 3D-printed actuator was investigated. The actuator lost deformation ability after cutting and complete separation because of the major leakage of the gas pressure applied. However, after a healing time of 10 min, the bending ability of the actuator with applied pressure was restored.

**Figure 46:**
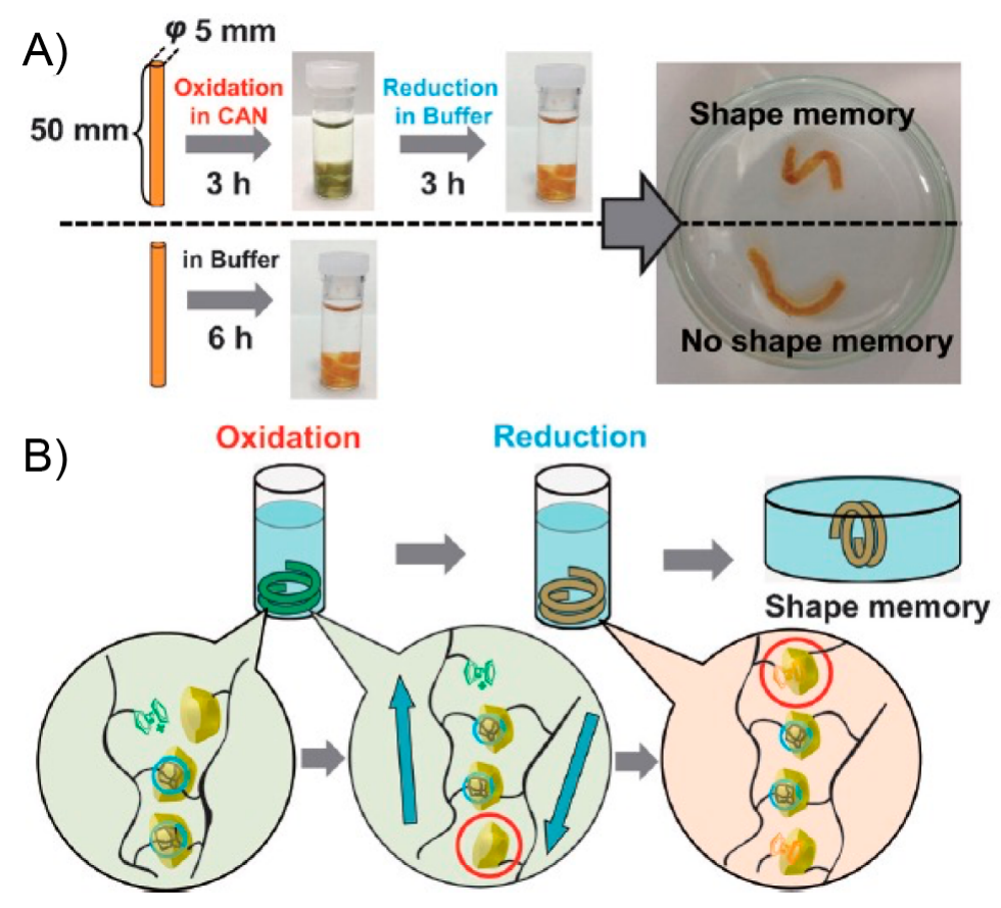
Shape-change experiment with the β-CD-Ad-Fc PAAm gel. First, a linear-cylindrical piece of β-CD-Ad-Fc PAAm gel (2,1,1) (diameter, 5 mm; length, 50 mm) was oxidized in an aqueous buffer containing CAN (25 mM) for 3 h, during which time it adopted a helical shape. Subsequently, the gel was reduced by immersion in the original buffer. When the gel was shaken in the buffer for 3 days, it retained its helical shape. This shape-memory effect did not occur without the oxidation–reduction cycle. (b) Schematic illustration of the shape-memory mechanism. Adapted with permission from ref (388). Copyright 2015 John Wiley and Sons.

Regarding redox-driven self-healing actuators, Miyamae et al. developed hydrogels by employing two different kinds of host–guest inclusion complexes of β-cyclodextrin (β-CD) with adamantane (Ad) and ferrocene (Fc) to bind polymers together to form a supramolecular hydrogel (β-CD-Ad-Fc gel).^388^ This gel showed self-healing ability when damaged and actuated to redox stimuli by expansion or contraction (Figure 46A). Even the β-CD-Ad-Fc gel showed a redox responsive shape-memory effect. The gel was prepared by conventional free-radical copolymerization, first β-CD–AAm and the guest monomers Ad–AAm and Fc–AAm were solvated by heating in a mixture of water and dimethyl sulfoxide (DMSO) (95:5, v/v). Then, the main-chain monomer (poly(acrylamide) (PAAm) and poly(*N*-isopropylacrylamide) (PNIPAAm)) selected to form the gel and water-soluble radical initiator VA-044 were added. Finally, the mixture was purged with argon and polymerized to give gels (*x*,*y*,*z*)). where *x*, *y*, and *z* represent the mol % of βCD–AAm, Ad–AAm, and Fc-AAm units. The authors investigated the self-healing properties resulting from the reversible noncovalent cross-links inside the gels. A monolith of β-CD-Ad-Fc PNIPAAm gel (6,3,3) was cut, and the two halves were then reattached together. After 3 h under wet conditions, it healed. In addition, to demonstrate the mechanism of self-healing, an aqueous solution of AdCANa (100 mm, 0.01 mL) was applied to one of the cut surfaces, no healing was observed. Indicates that the self-healing between cut surfaces is due to the reformation of host–guest inclusion complexes. Also, self-healing efficiency was estimated quantitatively by stress–strain measurements. The recovery ratio of the adhesive strength was determined by the amount of β-CD-Ad-Fc and the recovery time, and the best efficiency increased to 68% in the case of the β-CD-Ad-Fc PNIPAAm gel (10,5,5) with a healing time of 72 h. The redox responsive was demonstrated by a procedure in which first, a linear-cylindrical piece of β-CD-Ad-Fc PAAm gel (2,1,1) was oxidized in an aqueous buffer containing ceric ammonium nitrate (CAN) for 3 h, and during this time it adopted a helical shape. Afterward, the gel was reduced by immersion in the original buffer. When the gel was shaken in the buffer for 3 days, it retained its helical shape. This shape-memory effect did not occur without the oxidation–reduction cycle. Redox actuator mechanism is based in host–guest interaction that can be modulated by redox stimuli. The reduced form of Fc can be included in the cavity (host–guest), while the oxidized form cannot be included owing to its hydrophilic nature. The mechanism of self-healing properties resulting from the reversible noncovalent cross-links inside the gels (Figure 46B). Based on host–guest interactions between CDs and various guest moieties on polymer side chains. One is an inclusion complex between β-CD and adamantane (Ad) with an association constant (*K*) of 35 × 10^3^ M^–1^ forming a stable inclusion complex. The other is an inclusion complex between β-CD and Fc that form a relatively stable inclusion complex with a *K* value of 17 × 10^3^ M^–1^ with Fc in its reduced state.

## 3 Tabular Overview

In order to provide the readers with a practical and rapid guide of current technologies for the fabrication of polymeric soft actuators with self-healing capacity, we provide in Table 1 an overview of the main systems discussed in this review. Each entry is organized by the external stimuli employed, followed by main conditions for self-healing and for actuation, key mechanistic information for the driven-forces of both phenomena, and the most important features of the movement achieved during the actuation of the material.

**Table 1:**
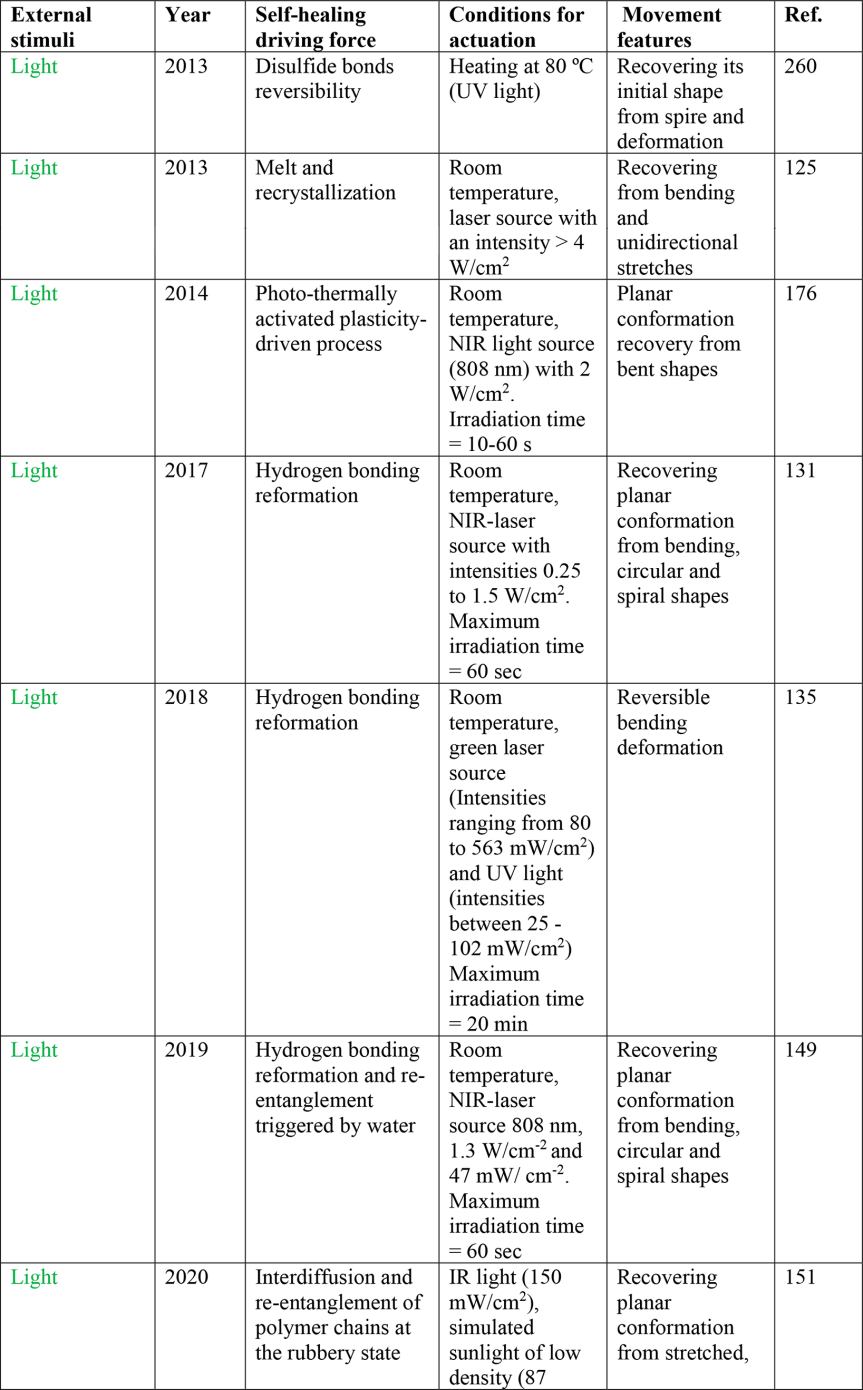

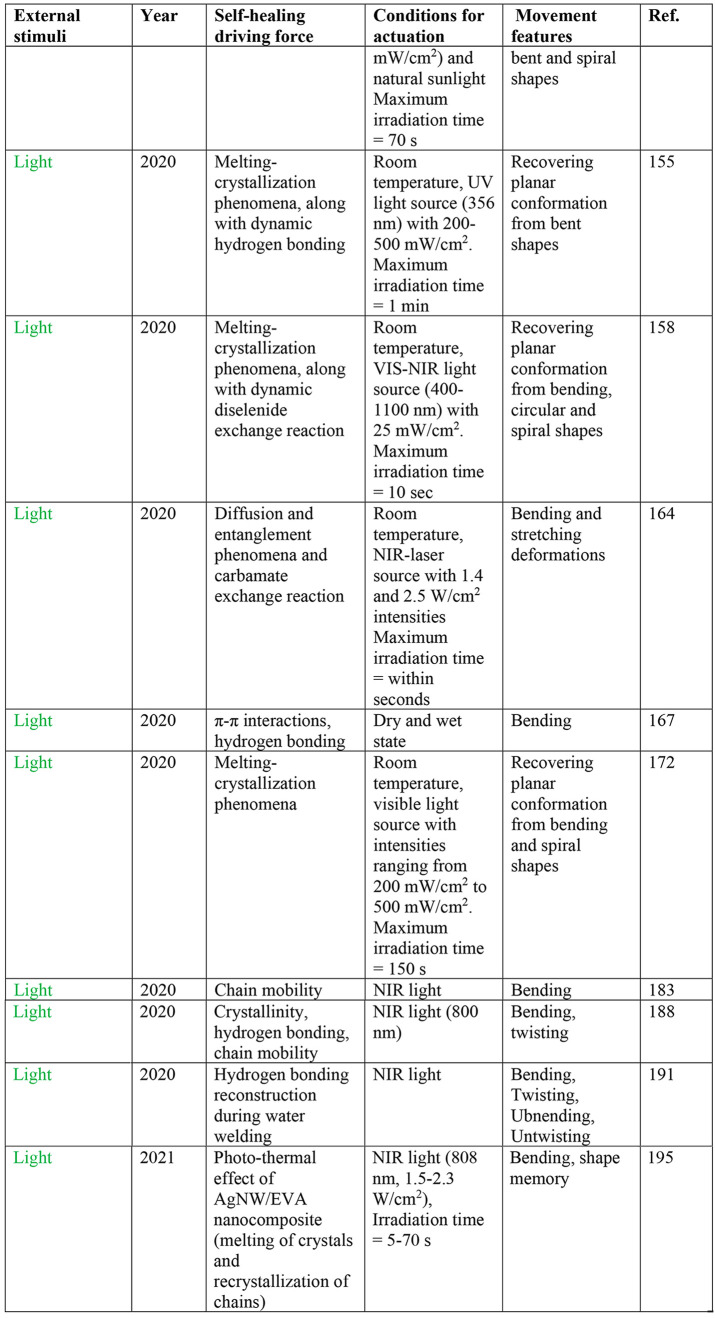

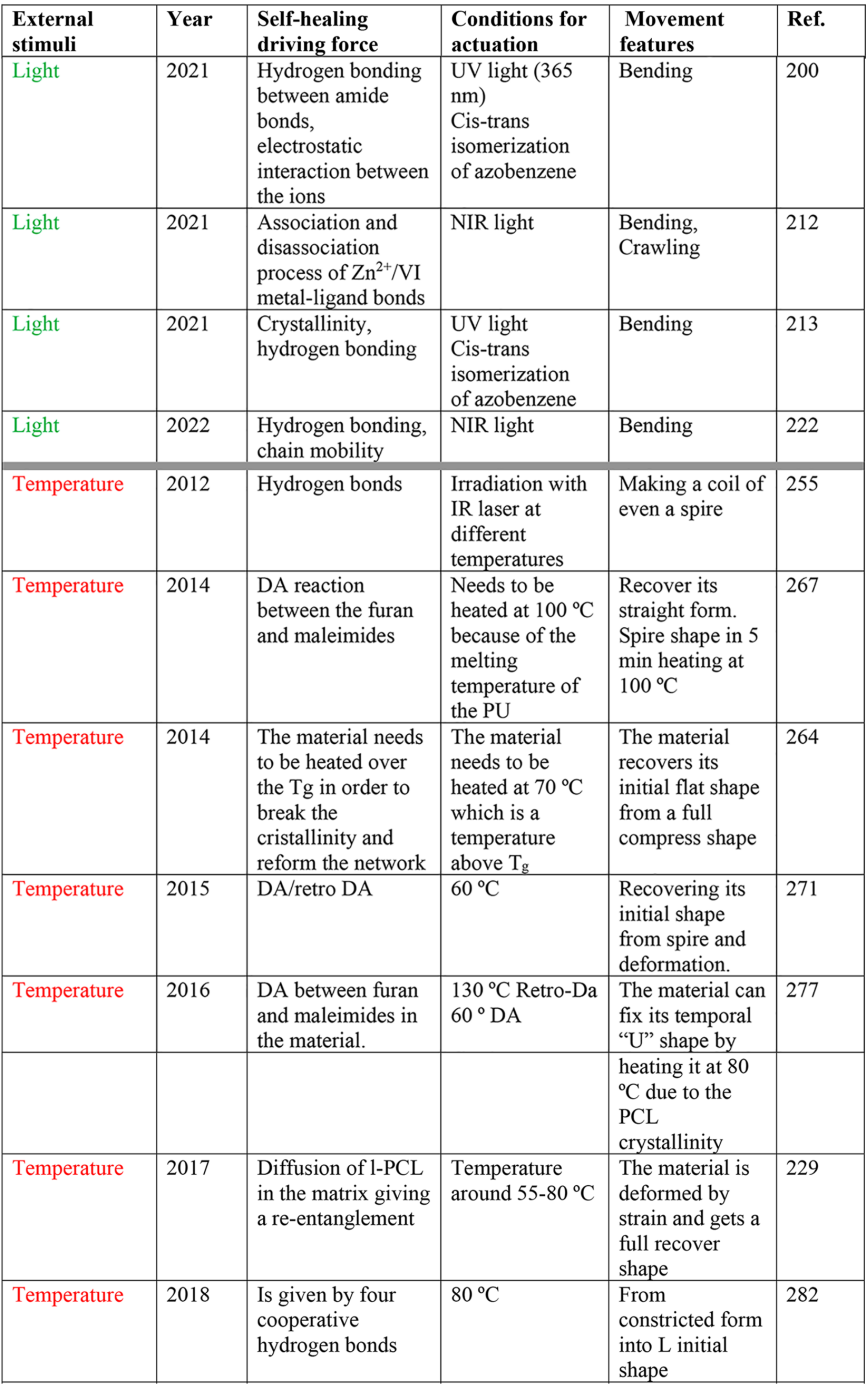

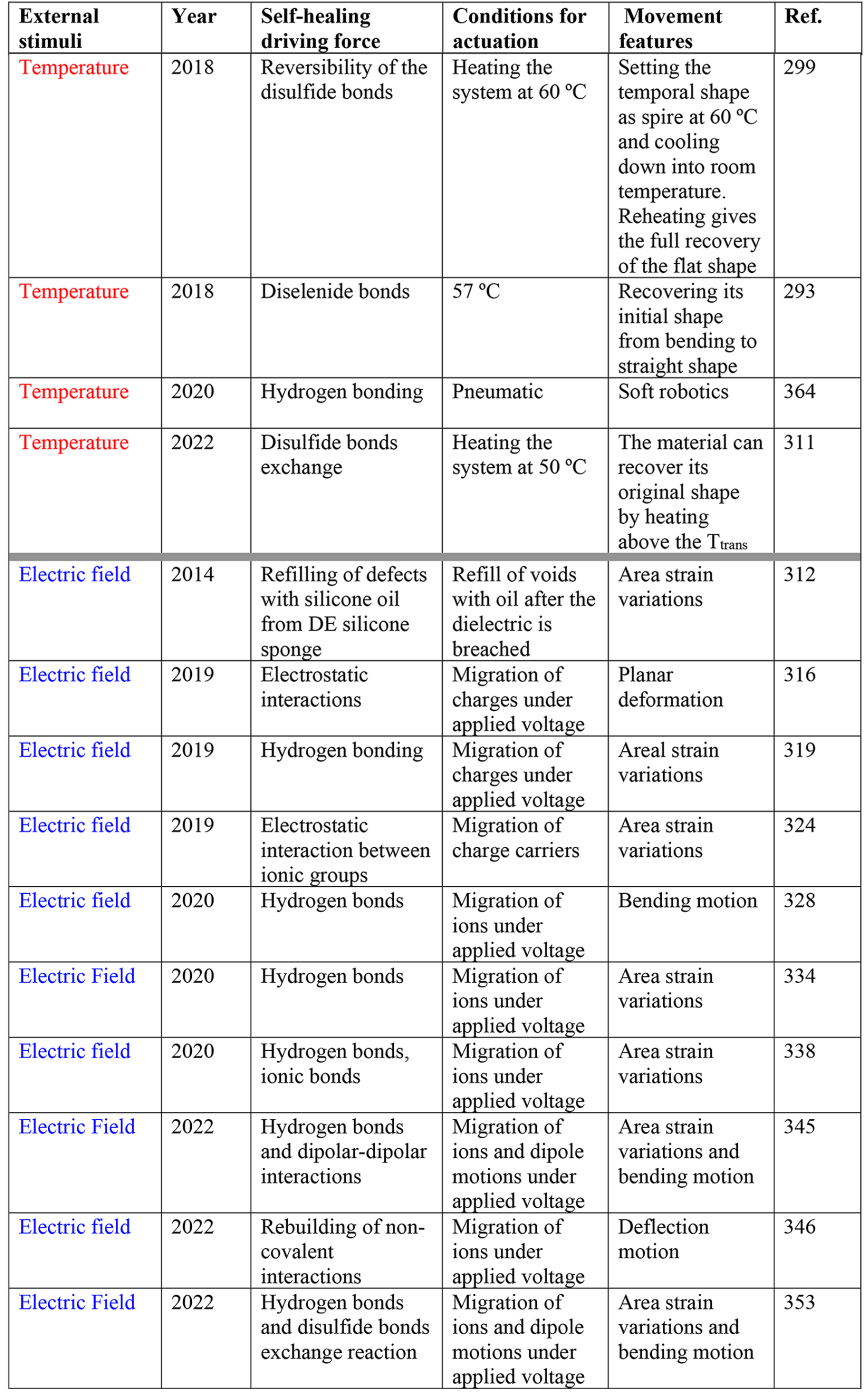

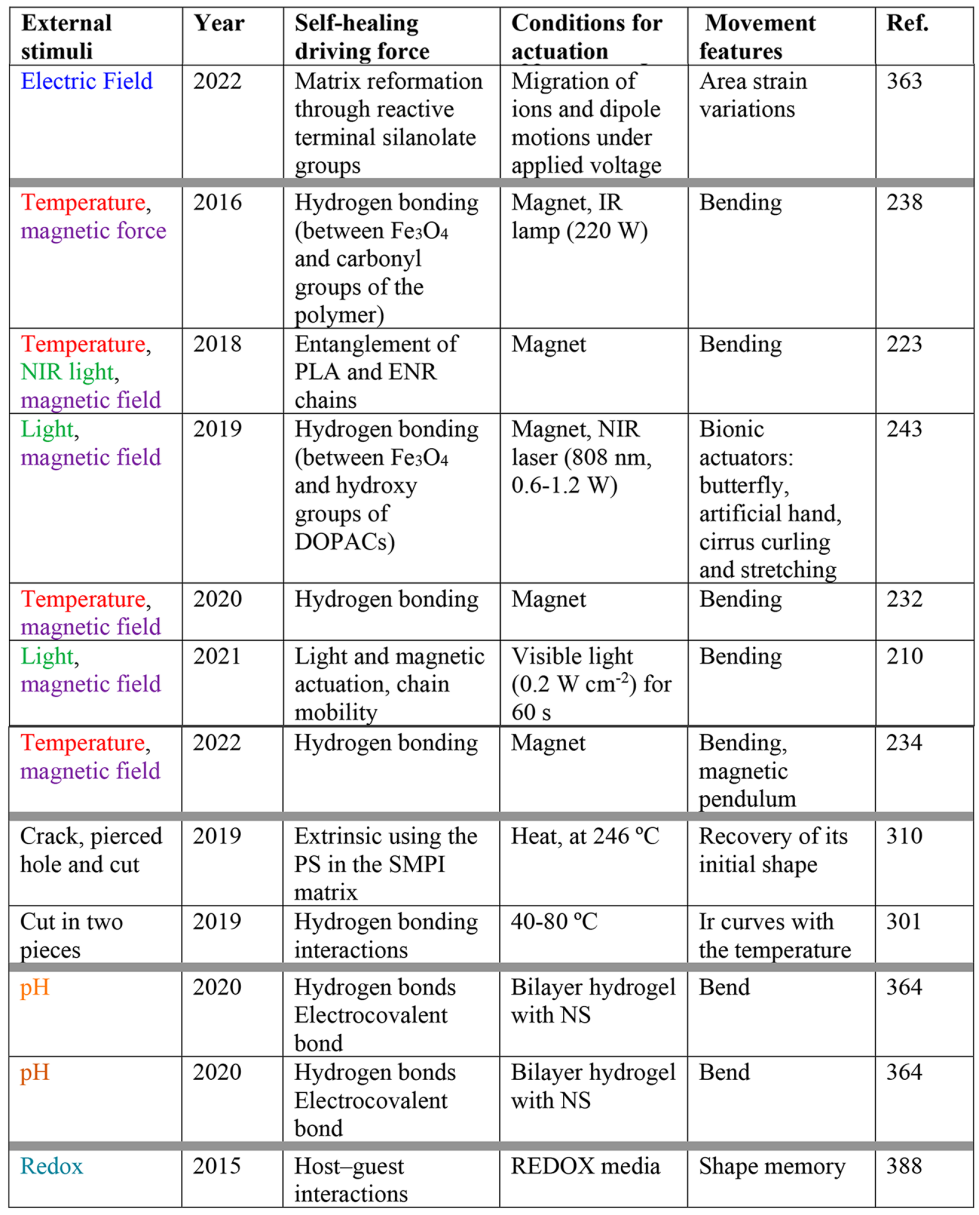
Summary of Self-Healing Polymeric Actuators Described in This Review with Indication of the External Stimuli Employed, Processes Involved in the Self-Healing Phenomenon, Specific Conditions for Actuation, and Type of Movement Achieved^a^^125,131,135,149,151,155,158,164,167,172,176,183,188,191,200,212,213,222,229,250,255,264,267,271,277,282,293,299,311,312,316,319,324,328,334,338,344,346,363,364,388^

a The examples are organized chronologically within each type of external stimulus.

## 4 Concluding Remarks

In conclusion, the amalgam between self-healing materials and polymeric actuators seems to be on the rise as it offers the possibility of designing soft actuators with self-repairing capabilities to continue functioning with unchanged efficiency after a damage. The scientific efforts made in this area seek to find new materials with longer lifespan at lower cost and with environmental friendliness and enhanced safety. A wide variety of chemistries regarding both extrinsic and intrinsic self-healing approaches have been developed by the research community over the last years, and some of them are being successfully implemented in the fabrication of self-healing polymeric soft actuators. In particular, reversible reactions (e.g., formation/rupture of disulfide and diselenide bonds DA reaction) and physical processes (e.g., host–guest interactions) are very promising tools for the synthesis of oriented polymers with high processability and self-repairing features. However, in many cases only small volumes of damage can be healed because material contact is required for healing, and cyclic chemical processes decrease the healing efficiency after repeated healing cycles. Polymeric soft actuators with self-healing and/or shape-memory capacity have the potential to revolutionize almost every aspect of our modern life, including healthcare, environmental remediation, energy management, entertainment, wearable devices, etc. In terms of external stimuli, light irradiation stands as one of the most versatile and appealing stimuli in this field nowadays. However, other stimuli such as thermal, electric/magnetic, especially combinations among them, together with the thickness control of the sample, constitute a promising strategy for tuning self-healing properties implemented into soft actuators and adapt them to the requirements of potential applications. The same external stimulus can trigger the self-healing and the actuation, although these two phenomena can also be activated individually and sequentially in some cases, in which nanoentities (e.g., nanoparticles, nanofibers, carbon-based nanomaterials) are included in the formulation of the final actuator.

Obviously, it is the final application of these soft actuators that will establish the requirements of, for instance, stiffness, impact resistance, adhesion strength, electrical conductivity, etc. This must be taken into consideration to avoid that the practical use of many of these materials remain elusive. With the numerous successful approaches described in this review, we believe that most materials fail at meeting those practical criteria as the formulation science to achieve the final product becomes more complex. In addition, it is vital in future innovative research to get deeper into the mechanisms of both self-healing and actuation when both properties are combined in a single material. For this, characterization techniques, such as electron microscopy, atomic force microscopy, IR spectroscopy, rheology, NMR, XRI and XPS, among others, take on a special value to understand the dynamic processes involved before, during, and after healing at the molecular level.

As can be confirmed during the reading of the present review article, an important number of the polymeric soft actuators discussed here can be classified as multicomponent systems (e.g., composites and polymer blends), and even so, it caught our attention the few times that the “compatibility” term was included by authors while describing their results. The search for compatibility between different components, aiming to fabricate more complex systems, has been from the very beginning a highly desirable property, while, at the same time, a challenging task within polymer and material science, among other fields. Increasing the compatibility of a multiphase system can be achieved by increasing the level (number and/or strength) of interactions between its components, causing a decrease in the stored interfacial energy within the system, which, thermodynamically, favors its stability. In this sense, reaching a good compatibility is not just preferred to attain an adequate phase-distribution between components but also to improve certain properties that are highly dependent on the chemical affinity existing inside the material, such as mechanical properties and, more relevant to the present topic, the self-healing capacity. Thus, we believe that essential advances in the next generation of self-healing soft actuators can be made by focusing efforts on finding new simple and scalable strategies that allow enhancing the degree of compatibility between their components, without neglecting the exploration of new chemical functionalities and their incorporation into novel formats of materials, pushing forward the development of more efficient systems.

Another constraint in this field is the limited fabrication techniques to scale up with precision the fabrication of self-healing soft actuators. Despite the development of the fabrication methods such as molding, 3D printing, and many thin-film fabrication and deposition techniques, their application to complex inner structures (e.g., hollow structures) and large-scale manufacturing remain challenging in many cases, mainly due to some inherent properties of soft materials such as nonlinear stress–strain relations, hysteresis, and large deformation profiles.

We must continue learning from biological organisms that produce at the least energetic and materials cost remarkable examples of controlled motion by assembling lower-level actuators into larger structures in a hierarchical manner. Thus, we certainly have a promising but also very challenging future in developing new soft actuators that can self-repair over many cycles while meeting also all the functional criteria for high-tech applications. These materials could change and improve many of their applications in critical fields for the human being such as biomedicine, energy, and environmental conservation.

## Abbreviations

1D: one-dimensional
^1^H NMR: proton nuclear magnetic resonance
2D: two-dimensional
3D: three-dimensional
4D: four-dimensional
AA: acrylic acid
AAm: acrylic amide
Ad: adamantane
AgNWs: silver nanowires
AIE: aggregation-induced emission
AIEgens: aggregation-induced emission agents
Am: acrylamide
AMF: alternating magnetic fields
AMPS: 2-acrylamide-2-methyl-1-propanesulfonic acid
AP: gel material using agar and poly(acrylamide) gel
APS: ammonium persulfate
AuNPs: Au nanoparticles
BA: *n*-butyl acrylate
BDO: 1,4-butanediol
BIS: *N*,*N*-methylenebis(acrylamide)
c-PCL-2OH: cyclic poly(ε-caprolactone) bearing two OH functional groups
CA: citric acid
CAN: ceric ammonium nitrate
ChCl: choline chloride
CNTs: carbon nanotubes
CNTs*x*-*g*-CP*y*: CNTs-*graft*-poly(tetrahydrofurfuryl methacrylate-*co*-lauryl acrylate-*co*-1-vinyl imidazole) nanocomposite
CPPC: chlorinated poly(propylene carbonate)
CS: chitosan
CST: cassava starch
CTA: chain-transfer agent
CuSNPs: copper sulfide nanoparticles
DA: Diels–Alder
DCP: dicumyl peroxide
DCPU: dynamic cross-linked polyurea
DE: dielectric elastomer
DEA: dielectric elastomer actuator
DiSe: di(1-hydroxyethylene)diselenide
DLP: digital light projection
DMA: dynamic mechanical analysis
DMAA: *N*,*N*-dimethylacrylamide
DMSO: dimethyl sulfoxide
DOPAC: 3,4-dihydroxyphenylacetic acid
DOPO: 9,10-dihydro-9-oxa-10-phosphaphenanthrene
dPTB: polyurethane copolymer containing 1,4-butanediol
dPTD: polyurethane copolymer containing di(1-hydroxyethylene)diselenide)
DSC: differential scanning calorimetry
EDS: energy dispersive X-ray spectroscopy
ENR: epoxidized natural rubber
EVA: poly(ethylene-*co*-vinyl acetate)
Fc: ferrocene
FM: tetrahydroxy functionalized cross-linker
FP: frontal polymerization
FTIR: Fourier transform infrared spectroscopy
GO: graphene oxide
GPC: gel permeation chromatography
GPN: ionic hydrogel maden of gelatin, PAAm, and clay
HAc: acetic acid
HDI: hexamethylene diisocyanate
HEDS: bis(2-hydroxyethyl) disulfide
HNMA: (*E*)-4-(((2-hydroxynaphthalen-1-yl)methylene)amino) benzoic acid
i-PCL: unmodified poly(ε-caprolactone)
iEAPs: ionic electroactive polymers
IPTS: isocyanatopropyltriethoxysilane
K: association constant
l-PCL-2OH: linear poly(ε-caprolactone)-diol
LED: light emitting diode
M_2_: bifunctional maleimide
MA: maleic acid
MAA: methacrylic acid
MAH: maleic anhydride
MBA: *N*-methylenebis(acrylamide)
MBAA: *N*,*N*′-methylene(bis(acrylamide))
MDI: 4,4-methylenediphenyl diisocyanate
mfGO: multifunctionalized graphene oxide
MGSBS: methyl thioglycolate-modified styrene–butadiene–styrene
MMA: methyl methacrylate
MNPs: magnetic nanoparticles
MWCNTs: multiwalled carbon nanotubes
n-PCL: poly(ε-caprolactone) diacrylate derivative
NIR: near-infrared
NPs: nanoparticles
OEGMA: oligo ethylene glycol methyl ether methacrylate
OM: optical microscopy
PA: phthalic anhydride
PAA: poly(acrylic acid)
PAA-GO: poly(acrylic acid)-grafted graphene oxide
PAA-u: poly(acrylic acid) graft copolymer bearing 2-ureido-4[1*H*]-pyrimidinone units
PAAm: poly(acrylamide)
PAmA: poly(amic acid)
PANI: polyaniline
PCCP: condensation polymer made of poly(ethylene glycol), polytetramethylene ether glycol, citric acid, and phthalic anhydride
PCL: poly(ε-caprolactone)
PCLDMA: end-modified PCL chains with methacrylate units
PCLF_2_: bisfuranic-terminated poly(ε-caprolactone)
PDA: polydopamine
PDAPs: polydopamine particles
PDES: polymerizable deep eutectic solvent
PDMS: poly(dimethylsiloxane)
PDMS-NH_2_: amino terminated poly(dimethylsiloxane)
PEG: poly(ethylene glycol)
PEI: poly(ethylenimine)
PEO: poly(ethylene oxide)
PEU: poly(ester-urea)
PHA: poly(hexylene-adipate)
P(HEMA-*co*-AAc): poly(hydroxyethyl methacrylate-*co*-acrylic acid)
PLA: polylactide
PMS-*g*-COOH: carboxyl terminated poly(methylvinylsiloxane)
PNIPAAm: poly(*N*-isopropylacrylamide)
POM: polarized optical microscopy
PTFE: polytetrafluoroethylene
PTMG: polytetramethylene ether glycol
PU: polyurethane
PVA: poly(vinyl alcohol)
PVB: poly(vinyl butyral)
P(VDF-HFP): poly(vinylidene fluoride-*co*-hexafluoropropylene)
r-DA: retro Diels–Alder
RAFT: reversible addition–fragmentation chain-transfer
R_f_: shape fixing ratio
RPSM: reversible plasticity shape memory
R_r_: shape recovery ratio
SEM: scanning electron microscopy
SHSMPI: shape-memory polyimide
SiR-SN: silicon dielectric elastomer with supramolecular network
SPMA: 3-sulfopropyl methacrylate
SWNTs: single-walled carbon nanotubes
*T*: temperature
t-Azo: 3,3′,5,5-azobenzenetetracarboxylic acid
TDI: 2,4-toluene diisocyanate
TEA: triethanolamine
TEM: transmission electron microscopy
TEMED: *N*,*N*,*N*′,*N*′-tetramethyldiamine
*T*_g_: glass transition temperature
*T*_m_: melting temperature
*T*_trans_: thermal transition temperature
TMA: thermomechanical analysis
TMEDA: *N*,*N*,*N*′,*N*′-tetramethylethylenediamine
TPE-COOH: tetraphenyl ethylene
TPU: thermoplastic polyurethane
TPVs: thermoplastic vulcanizates
TRs: tandem repetitions
TUF_3_: thiourethane compound containing three furan moieties
UM_3_: urethane tris-maleimide derivative
UPy: 2-ureido-4[1*H*]-pyrimidinone
UPyMA: methacrylic monomer bearing 2-ureido-4[1*H*]-pyrimidinone motifs
UV: ultraviolet
UV–vis: ultraviolet–visible
*WAXD*: wide-angle X-ray scattering
wt %: weight percent
XRD: X-ray diffraction
β-CD: β-cyclodextrin

## References

[ref1] Cremaldi J. C.; Bhushan B. Bioinspired Self-Healing Materials: Lessons from Nature. Beilstein J. Nanotechnol. 2018, 9, 907–935. 10.3762/bjnano.9.85. 29600152 PMC5870156

[ref2] Withers P. C. Comparative Animal Physiology; Thompson Learning: Boston, MA, 1992.

[ref3] Ruppert E. E.; Fox R. S.; Barnes R. D. Invertebrate Zoology: A Functional Evolutionary Approach, 7th ed.; Brooks/Cole: Pacific Grove, CA, 2004.

[ref4] Judd W. S.; Campbell C. S.; Kellog E. A.; Stevens P. F.; Donoghue M. J. Plant Systematics: A Phylogenic Approach, 3rd ed.; Sinauer Associates: Sunderland, MA, 2008.

[ref5] Mora C.; Tittensor D. P.; Adl S.; Simpson A. G. B.; Worm B. How Many Species Are There on Earth and in the Ocean?. PLoS Biol. 2011, 9, e1001127 10.1371/journal.pbio.1001127. 21886479 PMC3160336

[ref6] Teyssier J.; Saenko S. V.; van der Marel D.; Milinkovitch M. C. Photonic Crystals Cause Active Colour Change in Chameleons. Nat. Commun. 2015, 6, 6368 10.1038/ncomms7368. 25757068 PMC4366488

[ref7] Rinkevich B.; Müller W. E. G., Eds. Invertebrate Immunology; Springer: Berlin, 1996.

[ref8] Krautz R.; Arefin B.; Theopold U. Damage Signals in the Insect Immune Response. Front. Plant Sci. 2014, 5, 342 10.3389/fpls.2014.00342. 25071815 PMC4093659

[ref9] Bulla L. A. Jr.; Cheng T. C. Invertebrate Immune Responses; Plenum Press: New York, 1997.

[ref10] Moffett S. B. Nervous System Regeneration in the Invertebrates; Springer-Verlag: Berlin, 1996.

[ref11] Crockett J. C.; Rogers M. J.; Coxon F. P.; Hocking L. J.; Helfrich M. H. Bone Remodelling at a Glance. J. Cell Sci. 2011, 124, 991–998. 10.1242/jcs.063032. 21402872

[ref12] Hoebe K.; Janssen E.; Beutler B. The Interface Between Innate and Adaptive Immunity. Nat. Immunol. 2004, 5, 971–974. 10.1038/ni1004-971. 15454919

[ref13] Moffett S. B. Nervous System Regeneration in the Invertebrates; Springer-Verlag: Berlin, 1996.

[ref14] Schmidt C. E.; Leach J. B. Neural Tissue Engineering: Strategies for Repair and Regeneration. Annu. Rev. Biomed. Eng. 2003, 5, 293–347. 10.1146/annurev.bioeng.5.011303.120731. 14527315

[ref15] Hadjidakis D. J.; Androulakis I. I. Bone Remodeling. Ann. N.Y. Acad. Sci. 2006, 1092, 385–396. 10.1196/annals.1365.035. 17308163

[ref16] Hickman C. P. Jr.; Roberts L. S.; Keen S. L.; Larson A.; I’Anson H.; Eisenhour D. J. Integrated Principles of Zoology, 14th ed.; McGraw Hill: New York, 2008.

[ref17] Metcalfe C. R. Distribution of Latex in the Plant Kingdom. Econ. Bot. 1967, 21, 115–127. 10.1007/BF02897859.

[ref18] Nguyen A. V.; Soulika A. M. The Dynamics of the Skin’s Immune System. Int. J. Mol. Sci. 2019, 20, 1811 10.3390/ijms20081811. 31013709 PMC6515324

[ref19] Bekas D. G.; Tsirka K.; Baltzis D.; Paipetis A. S. Self-Healing Materials: A Review of Advances in Materials, Evaluation, Characterization and Monitoring Techniques. Compos. B Eng. 2016, 87, 92–119. 10.1016/j.compositesb.2015.09.057.

[ref20] White S. R.; Sottos N. R.; Geubelle P. H.; Moore J. S.; Kessler M. R.; Sriram S. R.; Brown E. N.; Viswanathan S. Autonomic Healing of Polymer Composites. Nature 2001, 409, 794–797. 10.1038/35057232. 11236987

[ref21] Wang S.; Urban M. W. Self-Healing Polymers. Nat. Rev. Mater. 2020, 5, 562–583. 10.1038/s41578-020-0202-4.

[ref22] Jayabalakrishnan D.; Muruga D. B. N.; Bhaskar K.; Pavan P.; Balaji K.; Rajakumar P. S.; Priya C.; Deepa R. A. B.; Sendilvelan S.; Prabhahar M. Self-Healing Materials–A Review. Mater. Today: Proc. 2021, 45, 7195–7199. 10.1016/j.matpr.2021.02.415.

[ref23] Wool R. P.; O’Connor K. M. A Theory Crack Healing in Polymers. J. Appl. Phys. 1981, 52, 5953–5963. 10.1063/1.328526.

[ref24] Yang Y.; Davydovich D.; Hornat C. C.; Liu X.; Urban M. W. Leaf-Inspired Self-Healing Polymers. Chem. 2018, 4, 1928–1936. 10.1016/j.chempr.2018.06.001.

[ref25] Chen Y.; Kushner A. M.; Williams G. A.; Guan Z. Multiphase Design of Autonomic Self-Healing Thermoplastic Elastomers. Nat. Chem. 2012, 4, 467–472. 10.1038/nchem.1314. 22614381

[ref26] Nji J.; Li G. A Biomimic Shape Memory Polymer Based Self-Healing Particulate Composite. Polymer 2010, 51, 6021–6029. 10.1016/j.polymer.2010.10.021.

[ref27] Hornat C. C.; Urban M. W. Shape Memory Effects in Self-Healing Polymers. Prog. Polym. Sci. 2020, 102, 101208 10.1016/j.progpolymsci.2020.101208.

[ref28] Corten C. C.; Urban M. W. Repairing Polymers Using Oscillating Magnetic Field. Adv. Mater. 2009, 21, 5011–5015. 10.1002/adma.200901940. 25377855

[ref29] Yang Y.; Urban M. W. Self-Repairable Polyurethane Networks by Atmospheric Carbon Dioxide and Water. Angew. Chem., Int. Ed. 2014, 53, 12142–12147. 10.1002/anie.201407978. 25220903

[ref30] Ying H.; Zhang Y.; Cheng J. Dynamic Urea Bond for the Design of Reversible and Self-Healing Polymers. Nat. Commun. 2014, 5, 3218 10.1038/ncomms4218. 24492620 PMC4438999

[ref31] Chen X.; Dam M. A.; Ono K.; Mal A.; Shen H.; Nutt S. R.; Sheran K.; Wudl F. A Thermally Re-Mendable Cross-Linked Polymeric Material. Science 2002, 295, 1698–1702. 10.1126/science.1065879. 11872836

[ref32] Ghosh B.; Urban M. W. Self-Repairing Oxetane-Substituted Chitosan Polyurethane Networks. Science 2009, 323, 1458–1460. 10.1126/science.1167391. 19286550

[ref33] Imato K.; Nishihara M.; Kanehara T.; Amamoto Y.; Takahara A.; Otsuka H. Self-Healing of Chemical Gels Cross-Linked by Diarylbibenzofuranone-Based Trigger-Free Dynamic Covalent Bonds at Room Temperature. Angew. Chem., Int. Ed. 2012, 51, 1138–1142. 10.1002/anie.201104069. 22052848

[ref34] Cordier P.; Tournilhac F.; Soulié-Ziakovic C.; Leibler L. Self-Healing and Thermoreversible Rubber from Supramolecular Assembly. Nature 2008, 451, 977–980. 10.1038/nature06669. 18288191

[ref35] Burnworth M.; Tang L.; Kumpfer J. R.; Duncan A. J.; Beyer F. L.; Fiore G. L.; Rowan S. J.; Weder C. Optically Healable Supramolecular Polymers. Nature 2011, 472, 334–337. 10.1038/nature09963. 21512571

[ref36] Nakahata M.; Takashima Y.; Yamaguchi H.; Harada A. Redox-Responsive Self-Healing Materials Formed from Host–Guest Polymers. Nat. Commun. 2011, 2, 511 10.1038/ncomms1521. 22027591 PMC3207205

[ref37] Li Z.; Yu R.; Guo B. Shape-Memory and Self-Healing Polymers Based on Dynamic Covalent Bonds and Dynamic Noncovalent Interactions: Synthesis, Mechanism, and Application. ACS Appl. Bio Mater. 2021, 4, 5926–5943. 10.1021/acsabm.1c00606. 35006922

[ref38] Kessler M. R.; Sottos N. R.; White S. R. Self-Healing Structural Composite Materials. Compos. Part A Appl. Sci. Manuf. 2003, 34, 743–753. 10.1016/S1359-835X(03)00138-6.

[ref39] Guan Q.; Dai Y.; Yang Y.; Bi X.; Wen Z.; Pan Y. Near-infrared Irradiation Induced Remote and Efficient Self-healable Triboelectric Nanogenerator for Potential Implantable Electronics. Nano Energy 2018, 51, 333–339. 10.1016/j.nanoen.2018.06.060.

[ref40] Taynton P.; Zhu C.; Loob S.; Shoemaker R.; Pritchard J.; Jin Y.; Zhang W. Re-healable Polyimine Thermosets: Polymer Composition and Moisture Sensitivity. Polym. Chem. 2016, 7, 7052–7056. 10.1039/C6PY01395C.

[ref41] Cromwell O. R.; Chung J.; Guan Z. Malleable and Self-Healing Covalent Polymer Networks through Tunable Dynamic Boronic Ester Bonds. J. Am. Chem. Soc. 2015, 137, 6492–6495. 10.1021/jacs.5b03551. 25945818

[ref42] Postiglione G.; Turri S.; Levi M. Effect of The Plasticizer on the Self-Healing Properties of a Polymer Coating Based on the Thermoreversible Diels–Alder Reaction. Prog. Org. Coat. 2015, 78, 526–531. 10.1016/j.porgcoat.2014.05.022.

[ref43] Park J. S.; Darlington T.; Starr A. F.; Takahashi K.; Riendeau J.; Hahn H. T. Multiple Healing Effect of Thermally Activated Self-Healing Composites Based on Diels–Alder Reaction. Compos. Sci. Technol. 2010, 70, 2154–2159. 10.1016/j.compscitech.2010.08.017.

[ref44] Kötteritzsch J.; Stumpf S.; Hoeppener S.; Vitz J.; Hager M. D.; Schubert U. S. One-Component Intrinsic Self-Healing Coatings Based on Reversible Crosslinking by Diels–Alder Cycloadditions. Macromol. Chem. Phys. 2013, 214, 1636–1649. 10.1002/macp.201200712.

[ref45] Zhang W.; Duchet J.; Gerard J. F. Self-Healable Interfaces Based on Thermo-Reversible Diels–Alder Reactions in Carbon Fiber Reinforced Composites. J. Colloid Interface Sci. 2014, 430, 61–68. 10.1016/j.jcis.2014.05.007. 24998055

[ref46] Lafont U.; Moreno-Belle C.; van Zeijl H.; van der Zwaag S. Self-Healing Thermally Conductive Adhesives. J. Intell. Mater. Syst. Struct. 2014, 25, 67–74. 10.1177/1045389X13498314.

[ref47] Kakuta T.; Takashima Y.; Nakahata M.; Otsubo M.; Yamaguchi H.; Harada A. Preorganized Hydrogel: Self-Healing Properties of Supramolecular Hydrogels Formed by Polymerization of Host-Guest-Monomers that Contain Cyclodextrins and Hydrophobic Guest Groups. Adv. Mater. 2013, 25, 2849–2853. 10.1002/adma.201205321. 23423947

[ref48] Yang Y.; Urban M. W. Self-healing Polymeric Materials. Chem. Soc. Rev. 2013, 42, 7446–7467. 10.1039/c3cs60109a. 23864042

[ref49] Roy N.; Buhler E.; Lehn J.-M. Double Dynamic Self-Healing Polymers: Supramolecular and Covalent Dynamic Polymers Based on the Bis-Iminocarbohydrazide Motif. Polym. Int. 2014, 63, 1400–1405. 10.1002/pi.4646.

[ref50] Zhu D. Y.; Rong M. Z.; Zhang M. Q. Self-healing Polymeric Materials Based on Microencapsulated Healing Agents: From Design to Preparation. Prog. Polym. Sci. 2015, 49, 175–220. 10.1016/j.progpolymsci.2015.07.002.

[ref51] Crall M. D.; Keller M. W. Targeted Self-Healing by Magnetically Guiding Microcapsules. ACS Appl. Mater. Interfaces 2017, 9, 6504–6511. 10.1021/acsami.7b00459. 28095672

[ref52] Yuan L.; Huang S.; Gu A.; Liang G.; Chen F.; Hu Y.; Nutt S. A Cyanate Ester/Microcapsule System with Low Cure Temperature and Self-Healing Capacity. Compos. Sci. Technol. 2013, 87, 111–117. 10.1016/j.compscitech.2013.08.005.

[ref53] González-García Y.; García S. J.; Hughes A. E.; Mol J. M. C. A Combined Redox-Competition and Negative-Feedback SECM Study of Self-Healing Anticorrosive Coatings. Electrochem. Commun. 2011, 13, 1094–1097. 10.1016/j.elecom.2011.07.009.

[ref54] García S. J.; Fischer H. R.; White P. A.; Mardel J.; González-García Y.; Mol J. M. C.; Hughes A. E. Self-Healing Anticorrosive Organic Coating Based on an Encapsulated Water Reactive Silyl Ester: Synthesis and Proof of Concept. Prog. Org. Coat. 2011, 70, 142–149. 10.1016/j.porgcoat.2010.11.021.

[ref55] Hollamby M. J.; Fix D.; Donch I.; Borisova D.; Mohwald H.; Shchukin D. Hybrid Polyester Coating Incorporating Functionalized Mesoporous Carriers for the Holistic Protection of Steel Surfaces. Adv. Mater. 2011, 23, 1361–1365. 10.1002/adma.201003035. 21400596

[ref56] Huang M.; Yang J. Long-Term Performance Of 1H, 1H′, 2H, 2H′-Perfluorooctyl Triethoxysilane (POTS) Microcapsule-Based Self-Healing Anticorrosive Coatings. J. Intell. Mater. Syst. Struct. 2014, 25, 98–106. 10.1177/1045389X13505785.

[ref57] Nesterova T.; Dam-Johansen K.; Kiil S. Synthesis of Durable Microcapsules for Self-Healing Anticorrosive Coatings: A Comparison of Selected Methods. Prog. Org. Coat. 2011, 70, 342–352. 10.1016/j.porgcoat.2010.09.032.

[ref58] Liu X.; Zhang H.; Wang J.; Wang Z.; Wang S. Preparation of Epoxy Microcapsule Based Self-Healing Coatings and Their Behavior. Surf. Coat. Technol. 2012, 206, 4976–4980. 10.1016/j.surfcoat.2012.05.133.

[ref59] Li Q.; Siddaramaiah; Kim N. H.; Hui D.; Lee J. H. Effects of Dual Component Microcapsules of Resin and Curing Agent on the Self-Healing Efficiency of Epoxy. Compos. Part B Eng. 2013, 55, 79–85. 10.1016/j.compositesb.2013.06.006.

[ref60] Zhu D. Y.; Rong M. Z.; Zhang M. Q. Preparation and Characterization of Multilayered Microcapsule-Like Microreactor for Self-Healing Polymers. Polymer 2013, 54, 4227–4236. 10.1016/j.polymer.2013.06.014.

[ref61] Toohey K. S.; Sottos N. R.; Lewis J. A.; Moore J. S.; White S. R. Self-Healing Materials with Microvascular Networks. Nat. Mater. 2007, 6, 581–585. 10.1038/nmat1934. 17558429

[ref62] Hamilton A. R.; Sottos N. R.; White S. R. Self-Healing of Internal Damage in Synthetic Vascular Materials. Adv. Mater. 2010, 22, 5159–5163. 10.1002/adma.201002561. 20872411

[ref63] Norris C. J.; Bond I. P.; Trask R. S. Interactions Between Propagating Cracks and Bioinspired Self-Healing Vascules Embedded in Glass Fibre Reinforced Composites. Compos. Sci. Technol. 2011, 71, 847–853. 10.1016/j.compscitech.2011.01.027.

[ref64] Peterson A. M.; Kotthapalli H.; Rahmathullah M. A. M.; Palmese G. R. Investigation of Interpenetrating Polymer Networks for Self-Healing Applications. Compos. Sci. Technol. 2012, 72, 330–336. 10.1016/j.compscitech.2011.11.022.

[ref65] Grunenfelder L. K.; Suksangpanya N.; Salinas C.; Milliron G.; Yaraghi N.; Herrera S.; Evans-Lutterodt K.; Nutt S. R.; Zavattieri P.; Kisailus D. Bio-Inspired Impact-Resistant Composites. Acta Biomater. 2014, 10, 3997–4008. 10.1016/j.actbio.2014.03.022. 24681369

[ref66] Esser-Kahn A. P.; Thakre P. R.; Dong H.; Patrick J. F.; Vlasko-Vlasov V. K.; Sottos N. R.; Moore J. S.; White S. R. Three-Dimensional Microvascular Fiber-Reinforced Composites. Adv. Mater. 2011, 23, 3654–3658. 10.1002/adma.201100933. 21766345

[ref67] Yang T.; Wang C. H.; Zhang J.; He S.; Mouritz A. P. Toughening and Self-Healing of Epoxy Matrix Laminates Using Mendable Polymer Stitching. Compos. Sci. Technol. 2012, 72, 1396–1401. 10.1016/j.compscitech.2012.05.012.

[ref68] Coppola A. M.; Thakre P. R.; Sottos N. R.; White S. R. Tensile Properties and Damage Evolution in Vascular 3D Woven Glass/Epoxy Composites. Compos. Part A Appl. Sci. Manuf. 2014, 59, 9–17. 10.1016/j.compositesa.2013.12.006.

[ref69] Hansen C. J.; White S. R.; Sottos N. R.; Lewis J. A. Accelerated Self-Healing Via Ternary Interpenetrating Microvascular Networks. Adv. Funct. Mater. 2011, 21, 4320–4326. 10.1002/adfm.201101553.

[ref70] Trask R. S.; Norris C. J.; Bond I. P. Stimuli-Triggered Self-Healing Functionality in Advanced Fibre-Reinforced Composites. J. Intell. Mater. Syst. Struct. 2014, 25, 87–97. 10.1177/1045389X13505006.

[ref71] Zainuddin S.; Arefin T.; Fahim A.; Hosur M. V.; Tyson J. D.; Kumar A.; Trovillion J.; Jeelani S. Recovery and Improvement in Low-Velocity Impact Properties of E-Glass/Epoxy Composites Through Novel Self-Healing Technique. Compos. Struct. 2014, 108, 277–286. 10.1016/j.compstruct.2013.09.023.

[ref72] Koralagundi Matt A.K.; Strong S.; ElGammal T.; Amano R. S. Development of Novel Self-Healing Polymer Composites for Use in Wind Turbine Blades. J. Energy Resour. Technol. 2015, 137, 051202 10.1115/1.4029912.

[ref73] Martins P.; Correia D. M.; Correia V.; Lanceros-Mendez S. Polymer-Based Actuators: Back to the Future. Phys. Chem. Chem. Phys. 2020, 22, 15163–15182. 10.1039/D0CP02436H. 32633288

[ref74] Ionov L. Polymeric Actuators. Langmuir 2015, 31, 5015–5024. 10.1021/la503407z. 25386998

[ref75] Kongahage D.; Foroughi J. Actuator Materials: Review on Recent Advances and Future Outlook for Smart Textiles. Fibers 2019, 7, 21 10.3390/fib7030021.

[ref76] Pelrine R.; Kornbluh R.; Pei Q. B.; Joseph J. High-speed Electrically Actuated Elastomers with Strain Greater Than 100%. Science 2000, 287, 836–839. 10.1126/science.287.5454.836. 10657293

[ref77] Rus D.; Tolley M. T. Design, Fabrication and Control of Soft Robots. Nature 2015, 521, 467–475. 10.1038/nature14543. 26017446

[ref78] Laschi C.; Cianchetti M. Soft Robotics: New Perspectives for Robot Bodyware and Control. Front. Bioeng. Biotechnol. 2014, 2, 3 10.3389/fbioe.2014.00003. 25022259 PMC4090912

[ref79] Kim S.; Laschi C.; Trimmer B. Soft Robotics: A Bioinspired Evolution in Robotics. Trends Biotechnol. 2013, 31, 287–294. 10.1016/j.tibtech.2013.03.002. 23582470

[ref80] Trimmer B. Soft Robots. Curr. Biol. 2013, 23, R639–R641. 10.1016/j.cub.2013.04.070. 23928077

[ref81] Trivedi D.; Rahn C. D.; Kier W. M.; Walker I. D. Soft Robotics: Biological Inspiration, State of the Art, and Future Research. Appl. Bionics Biomech. 2008, 5, 520417 10.1155/2008/520417.

[ref82] Wei S.; Ghosh T. K. Bioinspired Structures for Soft Actuators. Adv. Mater. Technol. 2022, 7, 2101521 10.1002/admt.202101521.

[ref83] Feinberg A. W. Biological Soft Robotics. Annu. Rev. Biomed. Eng. 2015, 17, 243–265. 10.1146/annurev-bioeng-071114-040632. 26643022

[ref84] Morris R. J.; Blyth M. How Water Flow, Geometry, and Material Properties Drive Plant Movements. J. Exp. Bot. 2019, 70, 3549–3560. 10.1093/jxb/erz167. 31112593

[ref85] Burgert I.; Fratzl P. Actuation Systems in Plants as Prototypes for Bioinspired Devices.. Philos. Trans. R. Soc., A 2009, 367, 1541–1547. 10.1098/rsta.2009.0003. 19324722

[ref86] Dawson C.; Vincent J. F. V.; Rocca A.-M. How Pine Cones Open. Nature 1997, 390, 668 10.1038/37745.

[ref87] Hines L.; Petersen K.; Lum G. Z.; Sitti M. Soft Actuators for Small-Scale Robotics. Adv. Mater. 2017, 29, 1603483 10.1002/adma.201603483. 28032926

[ref88] Chen D.; Pei Q. Electronic Muscles and Skins: A Review of Soft Sensors and Actuators. Chem. Rev. 2017, 117, 11239–11268. 10.1021/acs.chemrev.7b00019. 28816043

[ref89] Gagnier C. E. Functional Design of Aircraft Electric Actuator Equipment. Trans. Am. Inst. Electr. Eng. 1944, 63, 813–815. 10.1109/T-AIEE.1944.5058803.

[ref90] Miriyev A.; Stack K.; Lipson H. Soft Material for Soft Actuators. Nat. Commun. 2017, 8, 596 10.1038/s41467-017-00685-3. 28928384 PMC5605691

[ref91] Zhang Y.; Ionov L. Actuating Porous Polyimide Films. ACS Appl. Mater. Interfaces 2014, 6, 10072–10077. 10.1021/am502492u. 24903283

[ref92] Martins P.; Lopes A. C.; Lanceros-Mendez S. Electroactive Phases of Poly(Vinylidene Fluoride): Determination, Processing and Applications. Prog. Polym. Sci. 2014, 39, 683–706. 10.1016/j.progpolymsci.2013.07.006.

[ref93] Shen Z.; Chen F.; Zhu X.; Yong K.-T.; Gu G. Stimuli-Responsive Functional Materials for Soft Robotics. J. Mater. Chem. B 2020, 8, 8972–8991. 10.1039/D0TB01585G. 32901646

[ref94] Huang W. M.; Ding Z.; Wang C. C.; Wei J.; Zhao Y.; Purnawali H. Shape Memory Materials. Mater. Today 2010, 13, 54–61. 10.1016/S1369-7021(10)70128-0.

[ref95] Ratna D.; Karger-Kocsis J. Recent Advances in Shape Memory Polymers and Composites: A Review. J. Mater. Sci. 2008, 43, 254–269. 10.1007/s10853-007-2176-7.

[ref96] Laschi C.; Cianchetti M.; Mazzolai B.; Margheri L.; Follador M.; Dario P. Soft Robot Arm Inspired by the Octopus. Adv. Robot. 2012, 26, 709–727. 10.1163/156855312X626343.

[ref97] Shang J.; Le X.; Zhang J.; Chen T.; Theato P. Trends in Polymeric Shape Memory Hydrogels and Hydrogel Actuators. Polym. Chem. 2019, 10, 1036–1055. 10.1039/C8PY01286E.

[ref98] Saint Martin L. B.; Mendes R. U.; Cavalca K. L. Electromagnetic Actuators for Controlling Flexible Cantilever Beams. Struct. Control Health Monit. 2018, 25, e2043 10.1002/stc.2043.

[ref99] Kim K. J.; Shahinpoor M. A Novel Method of Manufacturing Three-Dimensional Ionic Polymer–Metal Composites (Ipmcs) Biomimetic Sensors, Actuators and Artificial Muscles. Polymer 2002, 43, 797–802. 10.1016/S0032-3861(01)00648-6.

[ref100] Cianchetti M.; Mattoli V.; Mazzolai B.; Laschi C.; Dario P. A New Design Methodology of Electrostrictive Actuators for Bio-Inspired Robotics. Sens. Actuators B Chem. 2009, 142, 288–297. 10.1016/j.snb.2009.08.039.

[ref101] O’Halloran A.; O’Malley F.; McHugh P. A Review on Dielectric Elastomer Actuators, Technology, Applications, and Challenges. J. Appl. Phys. 2008, 104, 071101 10.1063/1.2981642.

[ref102] Mirfakhrai T.; Madden J. D. W.; Baughman R. H. Polymer Artificial Muscles. Mater. Today 2007, 10, 30–38. 10.1016/S1369-7021(07)70048-2.

[ref103] Galantini F.; Carpi F.; Gallone G. Effects of Plasticization of a Soft Silicone for Dielectric Elastomer Actuation. Smart Mater. Struct. 2013, 22, 104020 10.1088/0964-1726/22/10/104020.

[ref104] Shahinpoor M.; Kim K. J. Ionic Polymer-Metal Composites: I. Fundamentals. Smart Mater. Struct. 2001, 10, 819–833. 10.1088/0964-1726/10/4/327.

[ref105] Risse S.; Kussmaul B.; Krüger H.; Kofod G. A Versatile Method for Enhancement of Electromechanical Sensitivity of Silicone Elastomers. RSC Adv. 2012, 2, 9029–9035. 10.1039/c2ra21541a.

[ref106] La T.-G.; Lau G.-K. Very High Dielectric Strength for Dielectric Elastomer Actuators in Liquid Dielectric Immersion. Appl. Phys. Lett. 2013, 102, 192905 10.1063/1.4806976.

[ref107] Madsen F. B.; Yu L.; Skov A. L. Self-Healing, High-Permittivity Silicone Dielectric Elastomer. ACS Macro Lett. 2016, 5, 1196–1200. 10.1021/acsmacrolett.6b00662. 35614744

[ref108] Hajiesmaili E.; Khare E.; Chortos A.; Lewis J.; Clarke D. R. Voltage-Controlled Morphing of Dielectric Elastomer Circular Sheets into Conical Surfaces. Extreme Mech. Lett. 2019, 30, 100504 10.1016/j.eml.2019.100504.

[ref109] Chortos A.; Hajiesmaili E.; Morales J.; Clarke D. R.; Lewis J. A. 3D Printing of Interdigitated Dielectric Elastomer Actuators. Adv. Funct. Mater. 2020, 30, 1907375 10.1002/adfm.201907375.

[ref110] Kim H.; Lee J. A.; Ambulo C. P.; Lee H. B.; Kim S. H.; Naik V. V.; Haines C. S.; Aliev A.; Ovalle-Robles E. R.; Baughman R. H.; Ware T. H. Intelligently Actuating Liquid Crystal Elastomer-Carbon Nanotube Composites. Adv. Funct. Mater. 2019, 29, 1905063 10.1002/adfm.201905063.

[ref111] Liu L.; Liu M.-H.; Deng L.-L.; Lin B.-P.; Yang H. Near-Infrared Chromophore Functionalized Soft Actuator with Ultrafast Photoresponsive Speed and Superior Mechanical Property. J. Am. Chem. Soc. 2017, 139, 11333–11336. 10.1021/jacs.7b06410. 28786668

[ref112] Donovan B. R.; Matavulj V. M.; Ahn S.-K.; Guin T.; White T. J. All-Optical Control of Shape. Adv. Mater. 2019, 31, 1805750 10.1002/adma.201805750. 30417450

[ref113] Iamsaard S.; Aßhoff S. J.; Matt B.; Kudernac T.; Cornelissen J. J. L. M.; Fletcher S. P.; Katsonis N. Conversion of Light into Macroscopic Helical Motion. Nat. Chem. 2014, 6, 229–235. 10.1038/nchem.1859. 24557138

[ref114] Yu C.; Duan Z.; Yuan P.; Li Y.; Su Y.; Zhang X.; Pan Y.; Dai L. L.; Nuzzo R. G.; Huang Y.; et al. Electronically Programmable, Reversible Shape Change in Two- and Three-Dimensional Hydrogel Structures. Adv. Mater. 2013, 25, 1541–1546. 10.1002/adma.201204180. 23255239

[ref115] Liang S.; Tu Y.; Chen Q.; Jia W.; Wang W.; Zhang L. Microscopic Hollow Hydrogel Springs, Necklaces and Ladders: A Tubular Robot as a Potential Vascular Scavenger. Mater. Horiz. 2019, 6, 2135–2142. 10.1039/C9MH00793H.

[ref116] Xiang C.; Wang Z.; Yang C.; Yao X.; Wang Y.; Suo Z. Stretchable and Fatigue-Resistant Materials. Mater. Today 2020, 34, 7–16. 10.1016/j.mattod.2019.08.009.

[ref117] Larson C.; Peele B.; Li S.; Robinson S.; Totaro M.; Beccai L.; Mazzolai B.; Shepherd R. Highly Stretchable Electroluminescent Skin for Optical Signaling and Tactile Sensing. Science 2016, 351, 1071–1074. 10.1126/science.aac5082. 26941316

[ref118] Qin H.; Zhang T.; Li N.; Cong H. P.; Yu S. H. Anisotropic and Self-Healing Hydrogels with Multi-Responsive Actuating Capability. Nat. Commun. 2019, 10, 2202 10.1038/s41467-019-10243-8. 31101823 PMC6525195

[ref119] Tarazón R. L. Robotics in Micro-manufacturing and Micro-robotics, Micromanufacturing Engineering and Technology. Micro Nano Technol. 2015, 661–674. 10.1016/B978-0-323-31149-6.00028-1.

[ref120] Kongahage D.; Foroughi J. Actuator Materials: Review on Recent Advances and Future Outlook for Smart Textiles. Fibers 2019, 7, 21 10.3390/fib7030021.

[ref121] Huang Y.; Yu Q.; Su C.; Jiang J.; Chen N.; Shao H. Light-Responsive Soft Actuators: Mechanism, Materials. Fabrication, and Applications. Actuators 2021, 10, 298 10.3390/act10110298.

[ref122] Roels E.; Terryn S.; Iida F.; Bosman A. W.; Norvez S.; Clemens F.; Van Assche G.; Vanderborght B.; Brancart J. Processing of Self-Healing Polymers for Soft Robotics. Adv. Mater. 2022, 34, 2104798 10.1002/adma.202104798. 34610181

[ref123] Kim S.-M.; Jeon H.; Shin S.-H.; Park S.-A; Jegal J.; Hwang S. Y.; Oh D. X.; Park J. Superior Toughness and Fast Self-Healing at Room Temperature Engineered by Transparent Elastomers. Adv. Mater. 2018, 30, 1705145 10.1002/adma.201705145. 29131415

[ref124] Ge Q. A.; Sakhaei H.; Lee H.; Dunn C. K.; Fang N. X.; Dunn M. L. Multimaterial 4D Printing with Tailorable Shape Memory Polymers. Sci. Rep. 2016, 6, 31110 10.1038/srep31110. 27499417 PMC4976324

[ref125] Zhang H.; Zhao Y. Polymers with Dual Light-Triggered Functions of Shape Memory and Healing Using Gold Nanoparticles. ACS Appl. Mater. Interfaces 2013, 5, 13069–13075. 10.1021/am404087q. 24308556

[ref126] Lim D. K.; Barhoumi A.; Wylie R. G.; Reznor G.; Langer R. S.; Kohane D. S. Enhanced Photothermal Effect of Plasmonic Nanoparticles Coated with Reduced Graphene Oxide. Nano Letters. 2013, 13, 4075–4079. 10.1021/nl4014315. 23899267

[ref127] Lukianova-Hleb E. Y.; Volkov A. N.; Wu X.; Lapotko D. O. Transient Enhancement and Spectral Narrowing of the Photothermal Effect of Plasmonic Nanoparticles Under Pulsed Excitation. Adv. Mater. 2013, 25, 772–776. 10.1002/adma.201204083. 23161793 PMC3772718

[ref128] Yang C.; Sui H.; Li X.; Han J.; Luo X.; Zhang H.; Sun H.; Sun H.; Zhou Y.; Yang B. Gold Nanoparticle Superstructures with Enhanced Photothermal Effect. CrystEngComm. 2013, 15, 3490–3497. 10.1039/c3ce26975b.

[ref129] Chen J.; Ye Z.; Yang F.; Yin Y. Plasmonic Nanostructures for Photothermal Conversion. Small Sci. 2021, 1, 2000055–2000072. 10.1002/smsc.202000055. PMC11935886 40212468

[ref130] Wang X.; Zhao J.; Chen M.; Ma L.; Zhao X.; Dang Z. M.; Wang Z. Improved Self-Healing of Polyethylene/Carbon Black Nanocomposites by their Shape Memory Effect. J. Phys. Chem. B 2013, 117, 1467–1474. 10.1021/jp3098796. 23301766

[ref131] Yang L.; Wang Z.; Fei G.; Xia H. Polydopamine Particles Reinforced Poly (Vinyl Alcohol) Hydrogel with NIR Light Triggered Shape Memory and Self-Healing Capability. Macromol. Rapid Commun. 2017, 38, 1700421–1700429. 10.1002/marc.201700421. 29027284

[ref132] Gao Y.; Wu X.; Zhou L.; Su Y.; Dong C. M. A Sweet Polydopamine Nanoplatform For Synergistic Combination Of Targeted Chemo-Photothermal Therapy. Macromol. Rapid Commun. 2015, 36, 916–922. 10.1002/marc.201500090. 25833346

[ref133] Liu Y.; Ai K.; Liu J.; Deng M.; He Y.; Lu L. Dopamine-Melanin Colloidal Nanospheres: An Efficient Near-Infrared Photothermal Therapeutic Agent for in Vivo Cancer Therapy. Adv. Mater. 2013, 25, 1353–1359. 10.1002/adma.201204683. 23280690

[ref134] Ai K.; Liu Y.; Ruan C.; Lu L.; Lu G. Sp2 C-Dominant N-Doped Carbon Sub-Micrometer Spheres with a Tunable Size: A Versatile Platform for Highly Efficient Oxygen-Reduction Catalysts. Adv. Mater. 2013, 25, 998–1003. 10.1002/adma.201203923. 23239109

[ref135] Si Q.; Feng Y.; Yang W.; Fu L.; Yan Q.; Dong L.; Long P.; Feng W. Controllable and Stable Deformation of a Self-Healing Photo-Responsive Supramolecular Assembly for an Optically Actuated Manipulator Arm. ACS Appl. Mater. Interfaces 2018, 10, 29909–29917. 10.1021/acsami.8b08025. 30047262

[ref136] Kondo M.; Yu Y.; Ikeda T. How Does the Initial Alignment of Mesogens Affect the Photoinduced Bending Behavior of Liquid-Crystalline Elastomers?. Angew. Chem., Int. Ed. 2006, 45, 1378–1382. 10.1002/anie.200503684. 16425331

[ref137] Chan W. W.; Lo S. F.; Zhou Z.; Yu W. Y. Rh-Catalyzed Intermolecular Carbenoid Functionalization of Aromatic C–H Bonds by α-Diazomalonates. J. Am. Chem. Soc. 2012, 134, 13565–13568. 10.1021/ja305771y. 22860697

[ref138] Samanta S.; Beharry A. A.; Sadovski O.; McCormick T. M.; Babalhavaeji A.; Tropepe V.; Woolley G. A. Photoswitching Azo Compounds in Vivo with Red Light. J. Am. Chem. Soc. 2013, 135, 9777–9784. 10.1021/ja402220t. 23750583

[ref139] Robertus J.; Reker S. F.; Pijper T. C.; Deuzeman A.; Browne W. R.; Feringa B. L. Kinetic Analysis of the Thermal Isomerisation Pathways in an Asymmetric Double Azobenzene Switch. Phys. Chem. Chem. Phys. 2012, 14, 4374–4382. 10.1039/c2cp23756c. 22354349

[ref140] Hu Y.; Li Z.; Lan T.; Chen W. Photoactuators for Direct Optical-To-Mechanical Energy Conversion: From Nanocomponent Assembly to Macroscopic Deformation. Adv. Mater. 2016, 28, 10548–10556. 10.1002/adma.201602685. 27604650

[ref141] Wani O. M.; Zeng H.; Priimagi A. A Light-Driven Artificial Flytrap. Nat. Commun. 2017, 8, 15546 10.1038/ncomms15546. 28534872 PMC5457518

[ref142] Xiong Y.; Zhang L.; Weis P.; Naumov P.; Wu S. A Solar Actuator Based on Hydrogen-Bonded Azopolymers for Electricity Generation. J. Mater. Chem. A 2018, 6, 3361–3366. 10.1039/C7TA11139H.

[ref143] Zhang Y.; Ma Y.; Sun J. Reversible Actuation of Polyelectrolyte Films: Expansion-Induced Mechanical Force Enables Cis–Trans Isomerization of Azobenzenes. Langmuir. 2013, 29, 14919–14925. 10.1021/la403019z. 24215493

[ref144] Lee K. M.; Wang D. H.; Koerner H.; Vaia R. A.; Tan L. S.; White T. J. Enhancement of Photogenerated Mechanical Force in Azobenzene-Functionalized Polyimides. Angew. Chem., Int. Ed. 2012, 51, 4117–4121. 10.1002/anie.201200726. 22407969

[ref145] Koshima H.; Ojima N.; Uchimoto H. Mechanical Motion of Azobenzene Crystals Upon Photoirradiation. J. Am. Chem. Soc. 2009, 131, 6890–6891. 10.1021/ja8098596. 19453188

[ref146] Wu W.; Yao L.; Yang T.; Yin R.; Li F.; Yu Y. NIR-Light-Induced Deformation of Cross-Linked Liquid-Crystal Polymers Using Upconversion Nanophosphors. J. Am. Chem. Soc. 2011, 133, 15810–15813. 10.1021/ja2043276. 21913658

[ref147] Wang D. H.; Wie J. J.; Lee K. M.; White T. J.; Tan L. S. Impact of Backbone Rigidity on the Photomechanical Response of Glassy, Azobenzene-Functionalized Polyimides. Macromolecules 2014, 47, 659–667. 10.1021/ma402178z.

[ref148] Vapaavuori J.; Laventure A.; Bazuin C. G.; Lebel O.; Pellerin C. Submolecular Plasticization Induced by Photons in Azobenzene Materials. J. Am. Chem. Soc. 2015, 137, 13510–13517. 10.1021/jacs.5b06611. 26439981

[ref149] Li T.; Li Y.; Wang X.; Li X.; Sun J. Thermally and Near-Infrared Light-Induced Shape Memory Polymers Capable of Healing Mechanical Damage and Fatigued Shape Memory Function. ACS Appl. Mater. Interfaces 2019, 11, 9470–9477. 10.1021/acsami.8b21970. 30735026

[ref150] Yanagisawa Y.; Nan Y.; Okuro K.; Aida T. Mechanically Robust, Readily Repairable Polymers Via Tailored Noncovalent Cross-Linking. Science 2018, 359, 72–76. 10.1126/science.aam7588. 29242235

[ref151] Cui X.; Chen J.; Zhu Y.; Jiang W. Natural Sunlight-Actuated Shape Memory Materials with Reversible Shape Change and Self-Healing Abilities Based on Carbon Nanotubes Filled Conductive Polymer Composites. Chem. Eng. J. 2020, 382, 122823–122834. 10.1016/j.cej.2019.122823.

[ref152] Wang M.; Sayed S. M.; Guo L. X.; Lin B. P.; Zhang X. Q.; Sun Y.; Yang H. Multi-Stimuli Responsive Carbon Nanotube Incorporated Polysiloxane Azobenzene Liquid Crystalline Elastomer Composites. Macromolecules 2016, 49, 663–671. 10.1021/acs.macromol.5b02388.

[ref153] Han B.; Zhang Y. L.; Chen Q. D.; Sun H. B. Carbon-Based Photothermal Actuators. Adv. Funct. Mater. 2018, 28, 1802235–1802258. 10.1002/adfm.201802235.

[ref154] Zhou P.; Chen L.; Yao L.; Weng M.; Zhang W. Humidity-and Light-Driven Actuators Based on Carbon Nanotube-Coated Paper and Polymer Composite. Nanoscale. 2018, 10, 8422–8427. 10.1039/C7NR09580E. 29637961

[ref155] Yan J.; Li M.; Wang Z.; Chen C.; Ma C.; Yang G. Highly Tough, Multi-Stimuli-Responsive, and Fast Self-Healing Supramolecular Networks Toward Strain Sensor Application. Chem. Eng. J. 2020, 389, 123468–123480. 10.1016/j.cej.2019.123468.

[ref156] Biyani M. V.; Foster E. J.; Weder C. Light-Healable Supramolecular Nanocomposites Based on Modified Cellulose Nanocrystals. ACS Macro Lett. 2013, 2, 236–240. 10.1021/mz400059w. 35581888

[ref157] Balkenende D. W.; Monnier C. A.; Fiore G. L.; Weder C. Optically Responsive Supramolecular Polymer Glasses. Nat. Commun. 2016, 7, 10995 10.1038/ncomms10995. 26983805 PMC4800438

[ref158] Du W.; Jin Y.; Lai S.; Shi L.; Shen Y.; Yang H. Multifunctional Light-Responsive Graphene-Based Polyurethane Composites with Shape Memory, Self-Healing, and Flame Retardancy Properties. Compos. Part A Appl. Sci. Manuf. 2020, 128, 105686–105697. 10.1016/j.compositesa.2019.105686.

[ref159] Jin Y.; Huang G.; Han D.; Song P.; Tang W.; Bao J.; Li R.; Liu Y. Functionalizing Graphene Decorated with Phosphorus-Nitrogen Containing Dendrimer for High-Performance Polymer Nanocomposites. Compos. Part A Appl. Sci. Manuf. 2016, 86, 9–18. 10.1016/j.compositesa.2016.03.030.

[ref160] Wang X.; Song L.; Yang H.; Xing W.; Kandola B.; Hu Y. Simultaneous Reduction and Surface Functionalization of Graphene Oxide with POSS for Reducing Fire Hazards in Epoxy Composites. J. Mater. Chem. 2012, 22, 22037–22043. 10.1039/c2jm35479a.

[ref161] Shi Y.; Yu B.; Zheng Y.; Yang J.; Duan Z.; Hu Y. Design of Reduced Graphene Oxide Decorated with DOPO-Phosphanomidate for Enhanced Fire Safety of Epoxy Resin. J. Colloid Interface Sci. 2018, 521, 160–171. 10.1016/j.jcis.2018.02.054. 29567604

[ref162] Ji S.; Cao W.; Yu Y.; Xu H. Dynamic Diselenide Bonds: Exchange Reaction Induced by Visible Light Without Catalysis. Angew. Chem., Int. Ed. 2014, 53, 6781–6785. 10.1002/anie.201403442. 24842614

[ref163] Xia J.; Ji S.; Xu H. Diselenide Covalent Chemistry at the Interface: Stabilizing an Asymmetric Diselenide-Containing Polymer Via Micelle Formation. Polym. Chem. 2016, 7, 6708–6713. 10.1039/C6PY01610C.

[ref164] Bai Y.; Zhang J.; Wen D.; Gong P.; Liu J.; Ju J.; Chen X. A Reconfigurable, Self-Healing and Near Infrared Light Responsive Thermoset Shape Memory Polymer. Compos. Sci. Technol. 2020, 187, 107940–107948. 10.1016/j.compscitech.2019.107940.

[ref165] Chen X.; Li L.; Jin K.; Torkelson J. M. Reprocessable Polyhydroxyurethane Networks Exhibiting Full Property Recovery and Concurrent Associative and Dissociative Dynamic Chemistry Via Transcarbamoylation and Reversible Cyclic Carbonate Aminolysis. Polym. Chem. 2017, 8, 6349–6355. 10.1039/C7PY01160A.

[ref166] Sheppard D. T.; Jin K.; Hamachi L. S.; Dean W.; Fortman D. J.; Ellison C. J.; Dichtel W. R. Reprocessing Postconsumer Polyurethane Foam Using Carbamate Exchange Catalysis and Twin-Screw Extrusion. ACS Cent. Sci. 2020, 6, 921–927. 10.1021/acscentsci.0c00083. 32607439 PMC7318067

[ref167] Jiang Z.; Tan M. L.; Taheri M.; Yan Q.; Tsuzuki T.; Gardiner M. G.; Diggle B.; Connal L. A. Strong, Self-Healable, and Recyclable Visible-Light-Responsive Hydrogel Actuators. Angew. Chem., Int. Ed. 2020, 59, 7049–7056. 10.1002/anie.201916058. 32167650

[ref168] Song P. N.; Hong J. L. Highly-Stretchable, Self-Healable Random Copolymers for Loading Large Amounts of Multiwall Carbon Nanotubes (Mwcnts) for the Preparation of Stretchable and Healable Electric Sensors. J. Mater. Chem. C 2019, 7, 13161–13175. 10.1039/C9TC03735G.

[ref169] Bai J.; Shi Z.; Yin J.; Tian M.; Qu R. Shape Reconfiguration of a Biomimetic Elastic Membrane with a Switchable Janus Structure. Adv. Funct. Mater. 2018, 28, 1800939–1800948. 10.1002/adfm.201800939.

[ref170] Takashima Y.; Hatanaka S.; Otsubo M.; Nakahata M.; Kakuta T.; Hashidzume A.; Yamaguchi H.; Harada A. Expansion–Contraction of Photoresponsive Artificial Muscle Regulated by Host–Guest Interactions. Nat. Commun. 2012, 3, 1270 10.1038/ncomms2280. 23232400 PMC3535346

[ref171] Iwaso K.; Takashima Y.; Harada A. Fast Response Dry-Type Artificial Molecular Muscles with [c2] Daisy Chains. Nat. Chem. 2016, 8, 625–632. 10.1038/nchem.2513. 27219709

[ref172] Chen Y.; Zhao X.; Luo C.; Shao Y.; Yang M. B.; Yin B. A Facile Fabrication of Shape Memory Polymer Nanocomposites with Fast Light-Response and Self-Healing Performance. Compos. Part A Appl. Sci. Manuf. 2020, 135, 105931–105940. 10.1016/j.compositesa.2020.105931.

[ref173] Kim Y. H.; Wool R. P. A Theory of Healing at a Polymer-Polymer Interface. Macromolecules 1983, 16, 1115–1120. 10.1021/ma00241a013.

[ref174] Wei H.; Yao Y.; Liu Y.; Leng J. A Dual-Functional Polymeric System Combining Shape Memory with Self-Healing Properties. Compos. B. Eng. 2015, 83, 7–13. 10.1016/j.compositesb.2015.08.019.

[ref175] Jing X.; Mi H. Y.; Huang H. X.; Turng L. S. Shape Memory Thermoplastic Polyurethane (TPU)/Poly (ε-Caprolactone)(PCL) Blends as Self-Knotting Sutures. J. Mech. Behav. Biomed. Mater. 2016, 64, 94–103. 10.1016/j.jmbbm.2016.07.023. 27490212

[ref176] Bongiovanni Abel S.; Molina M. A.; Rivarola C. R.; Kogan M. J.; Barbero C. A. Smart Polyaniline Nanoparticles with Thermal and Photothermal Sensitivity. Nanotechnology 2014, 25, 495602 10.1088/0957-4484/25/49/495602. 25407569

[ref177] Dong Y.; Geng C.; Liu C.; Gao J.; Zhou Q. Near-Infrared Light Photothermally Induced Shape Memory and Self-Healing Effects of Epoxy Resin Coating with Polyaniline Nanofibers. Synth. Met. 2020, 266, 116417–116428. 10.1016/j.synthmet.2020.116417.

[ref178] Zhou J.; Lu Z.; Zhu X.; Wang X.; Liao Y.; Ma Z.; Li F. NIR Photothermal Therapy Using Polyaniline Nanoparticles. Biomaterials 2013, 34, 9584–9592. 10.1016/j.biomaterials.2013.08.075. 24044996

[ref179] Chen Y.; Li C.; Hou Z.; Huang S.; Liu B.; He F.; Luo L.; Lin J. Polyaniline Electrospinning Composite Fibers for Orthotopic Photothermal Treatment of Tumors in Vivo. New J. Chem. 2015, 39, 4987–4993. 10.1039/C5NJ00327J.

[ref180] Jiang B. P.; Zhang L.; Zhu Y.; Shen X. C.; Ji S. C.; Tan X. Y.; Cheng L.; Liang H. Water-Soluble Hyaluronic Acid–Hybridized Polyaniline Nanoparticles for Effectively Targeted Photothermal Therapy. J. Mater. Chem. B 2015, 3, 3767–3776. 10.1039/C4TB01738B. 32262851

[ref181] Weng S.; Zhou J.; Lin Z. Preparation of One-Dimensional (1D) Polyaniline–Polypyrrole Coaxial Nanofibers and their Application in Gas Sensor. Synth. Met. 2010, 160, 1136–1142. 10.1016/j.synthmet.2010.02.037.

[ref182] Scotto J.; Florit M. I.; Posadas D. About the Species Formed During the Electrochemical Half Oxidation of Polyaniline: Polaron-Bipolaron Equilibrium. Electrochim. Acta 2018, 268, 187–194. 10.1016/j.electacta.2018.02.066.

[ref183] Li M.; Fu S.; Lucia L. A.; Wang Y. Ultra-Efficient Photo-Triggerable Healing and Shape-Memory Nanocomposite Materials Doped with Copper Sulfide Nanoparticles. Compos. Sci. Technol. 2020, 199, 108371–108379. 10.1016/j.compscitech.2020.108371.

[ref184] Zhang L.; Gao S.; Zhang F.; Yang K.; Ma Q.; Zhu L. Activatable Hyaluronic Acid Nanoparticle as a Theranostic Agent for Optical/Photoacoustic Image-Guided Photothermal Therapy. ACS Nano 2014, 8, 12250–12258. 10.1021/nn506130t. 25402600

[ref185] Wang S.; Riedinger A.; Li H.; Fu C.; Liu H.; Li L.; Liu T.; Tan L.; Barthel M. J.; Pugliese G.; et al. Plasmonic Copper Sulfide Nanocrystals Exhibiting Near-Infrared Photothermal and Photodynamic Therapeutic Effects. ACS Nano 2015, 9, 1788–1800. 10.1021/nn506687t. 25603353

[ref186] Zhang H.; Fortin D.; Xia H.; Zhao Y. Fast Optical Healing of Crystalline Polymers Enabled by Gold Nanoparticles. Macromol. Rapid Commun. 2013, 34, 1742–1746. 10.1002/marc.201300640. 24249088

[ref187] Huang L.; Yi N.; Wu Y.; Zhang Y.; Zhang Q.; Huang Y.; Ma Y.; Chen Y. Multichannel and Repeatable Self-Healing of Mechanical Enhanced Graphene-Thermoplastic Polyurethane Composites. Adv. Mater. 2013, 25, 2224–2228. 10.1002/adma.201204768. 23417742

[ref188] Qiu X.; Guo Q.; Wang Y.; Huang X.; Cao J.; Zheng Z.; Zhang X. Self-Healing and Reconfigurable Actuators Based on Synergistically Cross-Linked Supramolecular Elastomer. ACS Appl. Mater. Interfaces 2020, 12, 41981–41990. 10.1021/acsami.0c11708. 32835472

[ref189] Chen L.; Weng M.; Zhou P.; Zhang L.; Huang Z.; Zhang W. Multi-Responsive Actuators Based on a Graphene Oxide Composite: Intelligent Robot and Bioinspired Applications. Nanoscale 2017, 9, 9825–9833. 10.1039/C7NR01913K. 28585961

[ref190] Amjadi M.; Sitti M. Self-Sensing Paper Actuators Based on Graphite-Carbon Nanotube Hybrid Films. Adv. Sci. 2018, 5, 1800239–1800247. 10.1002/advs.201800239. PMC6051221 30027053

[ref191] Weng M.; Xiao Y.; Yao L.; Zhang W.; Zhou P.; Chen L. Programmable and Self-Healing Light-Driven Actuators through Synergetic Use of Water-Shaping and -Welding Methods. ACS Appl. Mater. Interfaces 2020, 12, 55125–55133. 10.1021/acsami.0c14380. 33253523

[ref192] Cheng H.; Huang Y.; Cheng Q.; Shi G.; Jiang L.; Qu L. Self-Healing Graphene Oxide Based Functional Architectures Triggered by Moisture. Adv. Funct. Mater. 2017, 27, 1703096–1703104. 10.1002/adfm.201703096.

[ref193] Luo C.; Yeh C. N.; Baltazar J. M. L.; Tsai C. L.; Huang J. A Cut-and-Paste Approach to 3D Graphene-Oxide-Based Architectures. Adv. Mater. 2018, 30, 1706229–1706235. 10.1002/adma.201706229. 29517826

[ref194] Mao J.; Chen Z.; Han D.; Ma J.; Zhang Y.; Sun H. Nacre-Inspired Moisture-Responsive Graphene Actuators with Robustness and Self-Healing Properties. Nanoscale 2019, 11, 20614–20619. 10.1039/C9NR06579B. 31641724

[ref195] Liu M.; Zhu S.; Huang Y.; Lin Z.; Liu W.; Yang L.; Ge D. A Self-Healing Composite Actuator for Multifunctional Soft Robot Via Photo-Welding. Compos. B. Eng. 2021, 214, 108748 10.1016/j.compositesb.2021.108748.

[ref196] Calisti M. Soft Robotics in Underwater Legged Locomotion: From Octopus-Inspired Solutions to Running Robots. In Soft Robotics: Trends, Applications and Challenges; Springer, 2017; pp 31–36.

[ref197] Qiang Y. X.; Zhu C. H.; Wu Y. P.; Cui S.; Liu Y. Bio-Inspired Semi-Transparent Silver Nanowire Conductor Based on a Vein Network with Excellent Electromechanical and Photothermal Properties. RSC Adv. 2018, 8, 23066–23076. 10.1039/C8RA02064G. 35540127 PMC9081629

[ref198] Li Q.; Chen L.; Xu H.; Liu Z.; Wei H. Photothermal Modulation of Propagating Surface Plasmons on Silver Nanowires. ACS Photonics 2019, 6, 2133–2140. 10.1021/acsphotonics.9b00711.

[ref199] Guan Q.; Picken S. J.; Sheiko S. S.; Dingemans T. J. High-Temperature Shape Memory Behavior of Novel All-Aromatic (AB) n-Multiblock Copoly(Ester Imide)S. Macromolecules 2017, 50, 3903–3910. 10.1021/acs.macromol.7b00569.

[ref200] Shen W.; Du B.; Liu J.; Zhuo H.; Yang C.; Chen S. A Facile Approach for the Preparation of Liquid Crystalline Polyurethane for Light-Responsive Actuator Films with Self-Healing Performance. Mater. Chem. Front. 2021, 5, 3192–3200. 10.1039/D0QM01084G.

[ref201] Jeong H. M.; Kim B. K.; Choi Y. J. Synthesis and Properties of Thermotropic Liquid Crystalline Polyurethane Elastomers. Polymer 2000, 41, 1849–1855. 10.1016/S0032-3861(99)00334-1.

[ref202] Wen Z.; Zhang T.; Hui Y.; Wang W.; Yang K.; Zhou Q.; Wang Y. Elaborate Fabrication Of Well-Defined Side-Chain Liquid Crystalline Polyurethane Networks With Triple-Shape Memory Capacity. J. Mater. Chem. A 2015, 3, 13435–13444. 10.1039/C5TA02537K.

[ref203] Niezgoda I.; Pociecha D.; Galewski Z. Monotropic or Enantiotropic Mesophases? Liquid-Crystalline and Solid State Polymorphism 4-Chloro-1, 3-Phenylene Bis-[4-(4-Alkyloxyphenylazo) Benzoates. Thermochim. Acta 2014, 587, 59–66. 10.1016/j.tca.2014.04.024.

[ref204] Merino E.; Ribagorda M. Control Over Molecular Motion Using the Cis–Trans Photoisomerization of the Azo Group. Beilstein J. Org. Chem. 2012, 8, 1071–1090. 10.3762/bjoc.8.119. 23019434 PMC3458724

[ref205] Yu H.; Ikeda T. Photocontrollable Liquid-Crystalline Actuators. Adv. Mater. 2011, 23, 2149–2180. 10.1002/adma.201100131. 21484890

[ref206] Wu Y.; Demachi Y.; Tsutsumi O.; Kanazawa A.; Shiono T.; Ikeda T. Photoinduced Alignment of Polymer Liquid Crystals Containing Azobenzene Moieties in the Side Chain. 2. Effect of Spacer Length of The Azobenzene Unit on Alignment Behavior. Macromolecules 1998, 31 (4), 1104–1108. 10.1021/ma971035v.

[ref207] Li Z.; Zhang X.; Wang S.; Yang Y.; Qin B.; Wang K.; Xie T.; Wei Y.; Ji Y. Polydopamine Coated Shape Memory Polymer: Enabling Light Triggered Shape Recovery, Light Controlled Shape Reprogramming and Surface Functionalization. Chem. Sci. 2016, 7, 4741–4747. 10.1039/C6SC00584E. 30155125 PMC6014076

[ref208] Dreyer D. R.; Miller D. J.; Freeman B. D.; Paul D. R.; Bielawski C. W. Elucidating the Structure of Poly (Dopamine). Langmuir 2012, 28, 6428–6435. 10.1021/la204831b. 22475082

[ref209] Wei Y.; Qi X.; He S.; Deng S.; Liu D.; Fu Q. Gradient Polydopamine Coating: A Simple and General Strategy Toward Multishape Memory Effects. ACS Appl. Mater. Interfaces 2018, 10, 32922–32934. 10.1021/acsami.8b13134. 30168310

[ref210] Chen Y.; Zhao X.; Li Y.; Jin Z.-Y.; Yang Y.; Yang M.-B.; Yin B. Light- And Magnetic-Responsive Synergy-Controlled Reconfiguration of Polymer Nanocomposites with Shape Memory Assisted Self-Healing Performance for Soft Robotics. J. Mater. Chem. C 2021, 9, 5515–5527. 10.1039/D1TC00468A.

[ref211] Wang J.; Liu H.; Liu Y.; Chu C.; Yang Y.; Zeng Y.; Zhang W.; Liu G. Eumelanin–Fe_3_O_4_ Hybrid Nanoparticles for Enhanced MR/PA Imaging-Assisted Local Photothermolysis. Biomater. Sci. 2018, 6, 586–595. 10.1039/C8BM00003D. 29389007

[ref212] Wang F.; Wang W.; Zhang C.; Tang J.; Zeng X.; Wan X. Scalable Manufactured Bio-Based Polymer Nanocomposite with Instantaneous Near-Infrared Light-Actuated Targeted Shape Memory and Remote-Controlled Accurate Self-Healing. Compos. Part B 2021, 219, 108927–108939. 10.1016/j.compositesb.2021.108927.

[ref213] Wang L.; Zhou Y.; Ma S.; Zhang H. Reprocessable and Healable Room Temperature Photoactuators Based on a Main-Chain Azobenzene Liquid Crystalline Poly(Ester-Urea). J. Mater. Chem. C 2021, 9, 13255–13265. 10.1039/D1TC03064G.

[ref214] Lahikainen M.; Zeng H.; Priimagi A. Reconfigurable Photoactuator through Synergistic Use of Photochemical and Photothermal Effects. Nat. Commun. 2018, 9, 4148 10.1038/s41467-018-06647-7. 30297774 PMC6175871

[ref215] Oscurato S. L.; Salvatore M.; Maddalena P.; Ambrosio A. From Nanoscopic to Macroscopic Photo-Driven Motion in Azobenzene-Containing Materials. Nanophotonics 2018, 7, 1387–1422. 10.1515/nanoph-2018-0040.

[ref216] Chen M.; Liang S.; Liu C.; Liu Y.; Wu S. Reconfigurable and Recyclable Photoactuators Based on Azobenzene-Containing Polymers. Front. Chem. 2020, 8, 706 10.3389/fchem.2020.00706. 32974276 PMC7471039

[ref217] Cheng Z.; Ma S.; Zhang Y.; Huang S.; Chen Y.; Yu H. Photomechanical Motion of Liquid-Crystalline Fibers Bending Away from a Light Source. Macromolecules 2017, 50, 8317–8324. 10.1021/acs.macromol.7b01741.

[ref218] Yoshino T.; Kondo M.; Mamiya J. I.; Kinoshita M.; Yu Y.; Ikeda T. Three-Dimensional Photomobility of Crosslinked Azobenzene Liquid-Crystalline Polymer Fibers. Adv. Mater. 2010, 22, 1361–1363. 10.1002/adma.200902879. 20437482

[ref219] Fang L.; Han G.; Zhang J.; Zhang H.; Zhang H. Synthesis of Well-Defined Easily Crosslinkable Azobenzene Side-Chain Liquid Crystalline Polymers Via Reversible Addition–Fragmentation Chain Transfer Polymerization and Photomechanical Properties of Their Post-Crosslinked Fibers. Eur. Polym. J. 2015, 69, 592–604. 10.1016/j.eurpolymj.2015.01.001.

[ref220] Fang L.; Zhang H.; Li Z.; Zhang Y.; Zhang Y.; Zhang H. Synthesis of Reactive Azobenzene Main-Chain Liquid Crystalline Polymers Via Michael Addition Polymerization and Photomechanical Effects of their Supramolecular Hydrogen-Bonded Fibers. Macromolecules 2013, 46, 7650–7660. 10.1021/ma401655k.

[ref221] Yu Y.; Nakano M.; Ikeda T. Photoinduced Bending and Unbending Behavior of Liquid-Crystalline Gels and Elastomers. Pure Appl. Chem. 2004, 76, 1467–1477. 10.1351/pac200476071467.

[ref222] Zhou Z.; Wang X.; Yu H.; Yu C.; Zhang F. Dynamic Cross-Linked Polyurea/Polydopamine Nanocomposites for Photoresponsive Self-Healing and Photoactuation. Macromolecules 2022, 55, 2193–2201. 10.1021/acs.macromol.1c02534.

[ref223] Huang J.; Cao L.; Yuan D.; Chen Y. Design of Novel Self-Healing Thermoplastic Vulcanizates Utilizing Thermal/Magnetic/Light-Triggered Shape Memory Effects. ACS Appl. Mater. Interfaces 2018, 10, 40996–41002. 10.1021/acsami.8b18212. 30456940

[ref224] Habault D.; Zhang H.; Zhao Y. Light-Triggered Self-Healing and Shape-Memory Polymers. Chem. Soc. Rev. 2013, 42, 7244–7256. 10.1039/c3cs35489j. 23440057

[ref225] Rodriguez E.; Luo X.; Mather P. Linear/Network Poly(ε-caprolactone) Blends Exhibiting Shape Memory Assisted Self-Healing (SMASH). ACS Appl. Mater. Interfaces 2011, 3, 152–161. 10.1021/am101012c. 21250636

[ref226] Lu C.; Liu Y.; Liu X.; Wang C.; Wang J.; Chu F. Sustainable Multiple- and Multistimulus-Shape-Memory and Self-Healing Elastomers with Semi-interpenetrating Network Derived from Biomass via Bulk Radical Polymerization. ACS Sustainable Chem. Eng. 2018, 6, 6527–6535. 10.1021/acssuschemeng.8b00329.

[ref227] Luo X.; Mather P. Shape Memory Assisted Self-Healing Coating. ACS Macro Lett. 2013, 2, 152–156. 10.1021/mz400017x. 35581778

[ref228] Li G.; Zhang H.; Fortin D.; Xia H.; Zhao Y. Poly(vinyl alcohol)-Poly(ethylene glycol) Double-Network Hydrogel: A General Approach to Shape Memory and Self-Healing Functionalities. Langmuir 2015, 31, 11709–11716. 10.1021/acs.langmuir.5b03474. 26442631

[ref229] Cao L.; Yuan D.; Xu C.; Chen Y. Biobased, Self-Healable, High Strength Rubber with Tunicate Cellulose Nanocrystals. Nanoscale 2017, 9, 15696–15706. 10.1039/C7NR05011A. 28994438

[ref230] Xu C.; Cui R.; Fu L.; Lin B. Recyclable and Heat-Healable Epoxidized Natural Rubber/Bentonite Composites. Compos. Sci. Technol. 2018, 167, 421–430. 10.1016/j.compscitech.2018.08.027.

[ref231] Xu C.; Zheng Z.; Wu W.; Wang Z.; Fu L. Dynamically Vulcanized PP/EPDM Blends with Balanced Stiffness and Toughness via in-Situ Compatibilization Of MAA and Excess Zno Nanoparticles: Preparation, Structure and Properties. Compos. Part B 2019, 160, 147–157. 10.1016/j.compositesb.2018.10.014.

[ref232] Muradyan H.; Mozhdehi D.; Guan Z. Self-Healing Magnetic Nanocomposites with Robust Mechanical Properties and High Magnetic Actuation Potential Prepared from Commodity Monomers via Graft-from Approach. Polym. Chem. 2020, 11, 1292–1297. 10.1039/C9PY01700C.

[ref233] Kumar S. K.; Jouault N.; Benicewicz B.; Neely T. Nanocomposites with polymer grafted nanoparticles. Macromolecules 2013, 46, 3199–3214. 10.1021/ma4001385.

[ref234] Tao H.-Q.; Yue D.-W.; Li C.-H. A Fast Self-Healing Magnetic Nanocomposite for Magnetic Actuators. Macromol. Mater. Eng. 2022, 307, 2100649 10.1002/mame.202100649.

[ref235] Yue D. W.; Wang H. Q.; Tao H. Q.; Zheng P.; Li C. H.; Zuo J. L. A Fast and Room-Temperature Self-Healing Thermal Conductive Polymer Composite. Chin. J. Polym. Sci. 2021, 39, 1328–1336. 10.1007/s10118-021-2620-1.

[ref236] Lai J. C.; Li L.; Wang D. P.; Zhang M. H.; Mo S. R.; Wang X.; Zeng K. Y.; Li C. H.; Jiang Q.; You X. Z.; et al. A Rigid and Healable Polymer Cross-Linked by Weak But Abundant Zn (II)-Carboxylate Interactions. Nat. Commun. 2018, 9, 2725 10.1038/s41467-018-05285-3. 30006515 PMC6045665

[ref237] Yang Y.; He J.; Li Q.; Gao L.; Hu J.; Zeng R.; Qin J.; Wang S. X.; Wang Q. Self-Healing of Electrical Damage in Polymers Using Superparamagnetic Nanoparticles. Nat. Nanotechnol. 2019, 14, 151–155. 10.1038/s41565-018-0327-4. 30598524

[ref238] Feng X. Q.; Zhang G. Z.; Bai Q. M.; Jiang H. Y.; Xu B.; Li H. J. High Strength Self-Healing Magnetic Elastomers with Shape Memory Effect. Macromol. Mater. Eng. 2016, 301, 125–132. 10.1002/mame.201500226.

[ref239] He Z.; Satarkar W. N.; Xie T.; Cheng Y. T.; Hilt J. Z. Remote Controlled Multishape Polymer Nanocomposites with Selective Radiofrequency Actuations. Adv. Mater. 2011, 23, 3192–3196. 10.1002/adma.201100646. 21638345

[ref240] You J.; Fu H.; Dong W.; Zhao L.; Cao X.; Li Y. Shape Memory Performance of Thermoplastic Polyvinylidene Fluoride/Acrylic Copolymer Blends Physically Cross-Linked by Tiny Crystals. ACS Appl. Mater. Interfaces 2012, 4, 4825–4831. 10.1021/am301161s. 22897334

[ref241] Gupta S.; Zhang Q.; Emrick T.; Balazs A. C.; Russell T. P. Entropy-Driven Segregation of Nanoparticles to Cracks in Multilayered Composite Polymer Structures. Nat. Mater. 2006, 5, 229–233. 10.1038/nmat1582.

[ref242] Lee J. Y.; Buxton G. A.; Balazs A. C. Using Nanoparticles to Create Self-Healing Composites. J. Chem. Phys. 2004, 121, 5531–5540. 10.1063/1.1784432. 15352848

[ref243] Wang Y.; Guo Q.; Su G.; Cao J.; Liu J.; Zhang X. Hierarchically Structured Self-Healing Actuators with Superfast Light- and Magnetic-Response. Adv. Funct. Mater. 2019, 29, 1906198–1906206. 10.1002/adfm.201906198.

[ref244] Liu X.; Su G.; Guo Q.; Lu C.; Zhou T.; Zhou C.; Zhang X. Hierarchically Structured Self-Healing Sensors with Tunable Positive/Negative Piezoresistivity. Adv. Funct. Mater. 2018, 28, 1706658–1706668. 10.1002/adfm.201706658.

[ref245] Cao J.; Lu C.; Zhuang J.; Liu M.; Zhang X.; Yu Y.; Tao Q. Multiple Hydrogen Bonfing Enables the Self-Healing of Sensors for Human-Machine Interactions. Angew. Chem., Int. Ed. 2017, 56, 8795–8800. 10.1002/anie.201704217. 28544097

[ref246] Liu J.; Guo Q.; Mao S.; Chen Z.; Zhang X.; Yang Y.; Zhang X. Templated synthesis of a 1D Ag nanohybrid in the solid state and its organized network for strain-sensing applications. J. Mater. Chem. C 2018, 6, 10730–10738. 10.1039/C8TC02720J.

[ref247] Yang S.; Cao C.; Sun Y.; Huang P.; Wei F.; Song W. Nanoscale Magnetic Bars for Heterogeneous Catalysis in Microscopic Systems. Angew. Chem., Int. Ed. 2015, 54, 2661–2664. 10.1002/anie.201410360. 25604535

[ref248] Ge R.; Li X.; Lin M.; Wang D.; Li S.; Liu S.; Tang Q.; Liu Y.; Jiang J.; Liu L.; et al. Fe_3_O_4_@polydopamine Composite Theranostic Superparticles Employing Preassembled Fe_3_O_4_ Nanoparticles as the Core. ACS Appl. Mater. Interfaces 2016, 8, 22942–22952. 10.1021/acsami.6b07997. 27560801

[ref249] Yu S.; Li G.; Liu R.; Ma D.; Xue W. Dendritic Fe_3_O_4_@Poly(dopamine)@PAMAM Nanocomposites as Controllable No-releasing Material: A Synergistic Photothermal and No Antibacterial Study. Adv. Funct. Mater. 2018, 28, 1707440–1707454. 10.1002/adfm.201707440.

[ref250] Yin X.; Zhang Y.; Lin P.; Liu Y.; Wang Z.; Yu B.; Zhou F.; Xue Q. Highly efficient thermogenesis from Fe3O4 nanoparticles for thermoplastic material repair both in air and underwater. J. Mater. Chem. A 2017, 5, 1221–1232. 10.1039/C6TA09227F.

[ref251] Mather P. T.; Luo X.; Rousseau I. A. Shape Memory Polymer Research. Annu. Rev. Mater. Res. 2009, 39, 445–471. 10.1146/annurev-matsci-082908-145419.

[ref252] Lee K. M.; Knight P. T.; Chung T.; Mather P. T. Polycaprolactone–POSS Chemical/Physical Double Networks. Macromolecules 2008, 41, 4730–4738. 10.1021/ma800586b.

[ref253] Leng J.; Lu H.; Liu Y.; Huang W. M.; Du S. Shape-Memory Polymers—A Class of Novel Smart Materials. MRS Bull. 2009, 34, 848–855. 10.1557/mrs2009.235.

[ref254] Xie T. Tunable Polymer Multi-Shape Memory Effect. Nature. 2010, 464, 267–270. 10.1038/nature08863. 20220846

[ref255] Kohlmeyer R. R.; Lor M.; Chen J. Remote, Local, and Chemical Programming of Healeable Multishape Memory Polymer Nanocomposite. Nano Lett. 2012, 12, 2757–2762. 10.1021/nl2044875. 22546074

[ref256] Lee J.; Seo J.; Han K.; Kim H. Preparation of Low Pt Loading Electrodes on Nafion (Na^+^)-Bonded Carbon Layer with Galvanostatic Pulses for PEMFC Application. J. Power Sources 2006, 163, 349–356. 10.1016/j.jpowsour.2006.09.018.

[ref257] Okada T.; Mo̷ller-Holst S.; Gorseth O.; Kjelstrup S. Transport and Equilibrium Properties of Nafion® Membranes with H^+^ and Na^+^ Ions. J. Electroanal. Chem. 1998, 442, 137–145. 10.1016/S0022-0728(97)00499-3.

[ref258] Hongsirikarn K.; Goodwin J. G. Jr; Greenway S.; Creager S. Effect of Cations (Na^+^, Ca^2+^, Fe^3+^) on the Conductivity of a Nafion Membrane. J. Power Sources 2010, 195, 7213–7220. 10.1016/j.jpowsour.2010.05.005.

[ref259] Voropaeva D. Y.; Novikova S. A.; Kulova T. L.; Yaroslavtsev A. B. Solvation and Sodium Conductivity of Nonaqueous Polymer Electrolytes Based on Nafion-117 Membranes and Polar Aprotic Solvents. Solid State Ion. 2018, 324, 28–32. 10.1016/j.ssi.2018.06.002.

[ref260] Michal B. T.; Jaye C. A.; Spencer E. J.; Rowan S. J. Inherently Photohealable and Termal Shape-Memory Polydisulfide Networks. ACS Macro Lett. 2013, 2, 694–699. 10.1021/mz400318m. 35606954

[ref261] Nair D. P.; Cramer N. B.; Gaipa J. C.; McBride M. K.; Matherly E. M.; McLeod R. R.; Shandas R.; Bowman C. N. Two-Stage Reactive Polymer Network Forming Systems. Adv. Funct. Mater. 2012, 22, 1502–1510. 10.1002/adfm.201102742. PMC3392718 22798700

[ref262] Canadell J.; Goossens H.; Klumperman B. Self-Healing Materials Based on Disulfide Links. Macromolecules 2011, 44, 2536–2541. 10.1021/ma2001492.

[ref263] Fairbanks B. D.; Singh S. P.; Bowman C. N.; Anseth K. S. Photodegradable, Photoadaptable Hydrogels Via Radical-Mediated Disulfide Fragmentation Reaction. Macromolecules 2011, 44, 2444–2450. 10.1021/ma200202w. 21512614 PMC3079292

[ref264] Bai Y.; Chen Y.; Wang Q.; Wang T. Poly (Vinyl Butyral) Based Polymer Networks with Dual-Responsive Shape Memory and Self-Healing Properties. J. Mater. Chem. 2014, 2, 9169–9177. 10.1039/c4ta00856a.

[ref265] Qiu Y.; Park K. Responsive Polymeric Delivery Systems. Adv. Drug Delivery Rev. 2001, 53, 321–339. 10.1016/S0169-409X(01)00203-4. 11744175

[ref266] Yang B.; Zhang H.; Peng H.; Xu Y.; Wu B.; Weng W.; Li L. Self-Healing Metallo-Supramolecular Polymers from a Ligand Macromolecule Synthesized Via Copper-Catalyzed Azide–Alkyne Cycloaddition and Thiol–Ene Double “Click” Reactions. Polym. Chem. 2014, 5, 1945–1953. 10.1039/C3PY00975K.

[ref267] Heo Y.; Sodano H. A. Self-Healing Polyurethanes with Shape Recovery. Adv. Funct. Mater. 2014, 24, 5261–5268. 10.1002/adfm.201400299.

[ref268] Chen X.; Wudl F.; Mal A. K.; Shen H.; Nutt S. R. New Thermally Remendable Highly Cross-Linked Polymeric Materials. Macromolecules 2003, 36, 1802–1807. 10.1021/ma0210675.

[ref269] Murphy E. B.; Bolanos E.; Schaffner-Hamann C.; Wudl F.; Nutt S. R.; Auad M. L. Synthesis and Characterization of a Single-Component Thermally Remendable Polymer Network: Staudinger and Stille Revisited. Macromolecules 2008, 41, 5203–5209. 10.1021/ma800432g.

[ref270] Wilson T. S.; Bearinger J. P.; Herberg J. L.; Marion J. E. III; Wright W. J.; Evans C. L.; Maitland D. J. Shape Memory Polymers Based on Uniform Aliphatic Urethane Networks. J. Appl. Polym. Sci. 2007, 106, 540–551. 10.1002/app.26593.

[ref271] Nguyen L.-T. T.; Truong T. T.; Nguyen H. T.; Le L.; Nguyen V. Q.; van Le T.; Luu A. T. Heleable Shape Memory (Thio)Urethane Thermosets. Polym. Chem. 2015, 6, 3143–3154. 10.1039/C5PY00126A.

[ref272] Rivero G.; Nguyen L. T. T.; Hillewaere X. K.; Du Prez F. E. One-Pot Thermo-Remendable Shape Memory Polyurethanes. Macromolecules 2014, 47, 2010–2018. 10.1021/ma402471c.

[ref273] Lu X.; Fei G.; Xia H.; Zhao Y. Ultrasound Healable Shape Memory Dynamic Polymers. J. Mater. Chem. A 2014, 2, 16051–16060. 10.1039/C4TA02726D.

[ref274] Hensarling R. M.; Rahane S. B.; LeBlanc A. P.; Sparks B. J.; White E. M.; Locklin J.; Patton D. L. Thiol–Isocyanate “Click” Reactions: Rapid Development of Functional Polymeric Surfaces. Polym. Chem. 2011, 2, 88–90. 10.1039/C0PY00292E.

[ref275] Nielsen C.; Weizman H.; Nemat-Nasser S. Thermally Reversible Cross-Links in a Healable Polymer: Estimating the Quantity, Rate of Formation, and Effect on Viscosity. Polymer 2014, 55, 632–641. 10.1016/j.polymer.2013.12.030.

[ref276] Nielsen C.; Weizman H.; Nemat-Nasser S. Thermally Reversible Cross-Links in a Healable Polymer: Estimating the Quantity, Rate of Formation, and Effect on Viscosity. Polymer 2014, 55, 632–641. 10.1016/j.polymer.2013.12.030.

[ref277] Chen W.; Zhou Y.; Li Y.; Sun J.; Pan X.; Yu Q.; Zhou N.; Zhang Z.; Zhu X. Shape-Memory and Self-Healing Polyurethanes Based on Cyclic Poly(Ε -Caprolactone). Polym. Chem. 2016, 7, 6789–6797. 10.1039/C6PY01638C.

[ref278] Wang L.; Yang X.; Chen H.; Gong T.; Li W.; Yang G.; Zhou S. Design of Triple-Shape Memory Polyurethane with Photo-Cross-Linking of Cinnamon Groups. ACS Appl. Mater. Interfaces 2013, 5, 10520–10528. 10.1021/am402091m. 24080202

[ref279] Yang X.; Wang L.; Wang W.; Chen H.; Yang G.; Zhou S. Triple Shape Memory Effect of Star-Shaped Polyurethane. ACS Appl. Mater. Interfaces 2014, 6, 6545–6554. 10.1021/am5001344. 24617646

[ref280] Lendlein A.; Kelch S. Shape-Memory Polymers. Angew. Chem., Int. Ed. 2002, 41, 2034–2057. 10.1002/1521-3773(20020617)41:12<2034::AID-ANIE2034>3.0.CO;2-M. 19746597

[ref281] Zhang K.; Lackey M. A.; Cui J.; Tew G. N. Gels Based on Cyclic Polymers. J. Am. Chem. Soc. 2011, 133, 4140–4148. 10.1021/ja111391z. 21351775

[ref282] Invernizzi M.; Turri S.; Levi M.; Suriano R. 4D Printed Thermally Activated Self-Healing and Shape-Memory Polycaprolactone-Based Polymers. Eur. Polym. J. 2018, 101, 169–176. 10.1016/j.eurpolymj.2018.02.023.

[ref283] Wei M.; Zhan M.; Yu D.; Xie H.; He M.; Yang K.; Wang Y. Novel Poly (Tetramethylene Ether) Glycol and Poly (ε-Caprolactone) Based Dynamic Network Via Quadruple Hydrogen Bonding with Triple-Shape Effect and Self-Healing Capacity. ACS Appl. Mater. Interfaces 2015, 7, 2585–2596. 10.1021/am507575z. 25558885

[ref284] Van Beek D. J. M.; Spiering A. J. H.; Peters G. W.; Te Nijenhuis K.; Sijbesma R. P. Unidirectional Dimerization and Stacking of Ureidopyrimidinone End Groups in Polycaprolactone Supramolecular Polymers. Macromolecules 2007, 40, 8464–8475. 10.1021/ma0712394.

[ref285] Voorhaar L.; Hoogenboom R. Supramolecular Polymer Networks: Hydrogels and Bulk Materials. Chem. Soc. Rev. 2016, 45, 4013–4031. 10.1039/C6CS00130K. 27206244

[ref286] Sijbesma R. P.; Beijer F. H.; Brunsveld L.; Folmer B. J.; Hirschberg J. K.; Lange R. F.; Lowe J. K. L.; Meijer E. W. Reversible Polymers Formed from Self-Complementary Monomers Using Quadruple Hydrogen Bonding. Science 1997, 278, 1601–1604. 10.1126/science.278.5343.1601. 9374454

[ref287] Torrado A. R.; Roberson D. A. Failure Analysis and Anisotropy Evaluation of 3D-Printed Tensile Test Specimens of Different Geometries and Print Raster Patterns. J. Fail. Anal. Prev. 2016, 16, 154–164. 10.1007/s11668-016-0067-4.

[ref288] Torrado Perez A. R.; Roberson D. A.; Wicker R. B. Fracture Surface Analysis of 3D-Printed Tensile Specimens of Novel ABS-Based Materials. J. Fail. Anal. Prev. 2014, 14, 343–353. 10.1007/s11668-014-9803-9.

[ref289] Zarek M.; Layani M.; Cooperstein I.; Sachyani E.; Cohn D.; Magdassi S. 3D Printing of Shape Memory Polymers for Flexible Electronic Devices. Adv. Mater. 2016, 28, 4449–4454. 10.1002/adma.201503132. 26402320

[ref290] Wang R.; Shen Y.; Qian D.; Sun J.; Zhou X.; Wang W.; Liu Z. Tensile and Torsional Elastomer Fiber Artificial Muscle by Entropic Elasticity with Thermo-Piezoresistive Sensing of Strain and Rotation by a Single Electric Signal. Mater. Horiz. 2020, 7, 3305–3315. 10.1039/D0MH01003K.

[ref291] Porath L. E.; Evans C. M. Importance of Broad Temperature Windows and Multiple Rheological Approaches for Probing Viscoelasticity and Entropic Elasticity in Vitrimers. Macromolecules 2021, 54, 4782–4791. 10.1021/acs.macromol.0c02800.

[ref292] Chen P.; Lin Y.; Zhao J.; Meng L.; Wang D.; Chen W.; Li L. Reconstructing the Mechanical Response of Polybutadiene Rubber Based on Micro-Structural Evolution in Strain-Temperature Space: Entropic Elasticity and Strain-Induced Crystallization as the Bridges. Soft Matter 2020, 16, 447–455. 10.1039/C9SM02029B. 31803885

[ref293] Du W.; Jin Y.; Pan J.; Fan W.; Lai S.; Sun X. Thermal Induced Shape-Memory and Self-Healing of Segmented Polyurethane Containing Diselenide Bonds. J. Appl. Polym. Sci. 2018, 135, 46326–46336. 10.1002/app.46326.

[ref294] Chang R.; Shan G.; Bao Y.; Pan P. Enhancement of Crystallizability and Control of Mechanical and Shape-Memory Properties for Amorphous Enantiopure Supramolecular Copolymers Via Stereocomplexation. Macromolecules 2015, 48, 7872–7881. 10.1021/acs.macromol.5b01986.

[ref295] Kim B. K.; Lee S. Y.; Xu M. Polyurethanes Having Shape Memory Effects. Polymer 1996, 37, 5781–5793. 10.1016/S0032-3861(96)00442-9.

[ref296] Xue L.; Dai S.; Li Z. Synthesis and Characterization of Elastic Star Shape-Memory Polymers as Self-Expandable Drug-Eluting Stents. J. Mater. Chem. 2012, 22, 7403–7411. 10.1039/c2jm15918j.

[ref297] An X.; Aguirresarobe R. H.; Irusta L.; Ruipérez F.; Matxain J. M.; Pan X.; Aramburu N.; Mecerreyes D.; Sardon H.; Zhu J. Aromatic Diselenide Crosslinkers to Enhance the Reprocessability and Self-Healing of Polyurethane Thermosets. Polym. Chem. 2017, 8, 3641–3646. 10.1039/C7PY00448F.

[ref298] Ji S.; Fan F.; Sun C.; Yu Y.; Xu H. Visible Light-Induced Plasticity of Shape Memory Polymers. ACS Appl. Mater. Interfaces 2017, 9, 33169–33175. 10.1021/acsami.7b11188. 28882033

[ref299] Zhang L.; Qiu T.; Zhu Z.; Guo L.; Li X. Self-Healing Polycaprolactone Networks Through Thermo-Induced Reversible Disulfide Bond. Macromol. Rapid Commun. 2018, 39, 1800121–1800126. 10.1002/marc.201800121. 30040138

[ref300] Matxain J. M.; Asua J. M.; Ruipérez F. Design of New Disulfide-Based Organic Compounds for the Improvement of Self-Healing Materials. Phys. Chem. Chem. Phys. 2016, 18, 1758–1770. 10.1039/C5CP06660C. 26675660

[ref301] Li Q.; Liu J.-D.; Liu S.-S.; Wang C.-F.; Chen S. Frontal Polymerization-Oriented Self-Healing Hydrogels and Applications toward Temperature-Triggered Actuators. Ind. Eng. Chem. Res. 2019, 58, 3885–3892. 10.1021/acs.iecr.8b05369.

[ref302] Liu Y. Y.; Fan X. D. Synthesis and Characterization of Ph-and Temperature-Sensitive Hydrogel of N-Isopropylacrylamide/Cyclodextrin Based Copolymer. Polymer 2002, 43, 4997–5003. 10.1016/S0032-3861(02)00350-6.

[ref303] McQueen-Mason S.; Cosgrove D. J. Disruption of Hydrogen Bonding Between Plant Cell Wall Polymers by Proteins that Induce Wall Extension. Proc. Natl. Acad. Sci. U. S. A. 1994, 91, 6574–6578. 10.1073/pnas.91.14.6574. 11607483 PMC44245

[ref304] Jiang S.; Liu F.; Lerch A.; Ionov L.; Agarwal S. Unusual and Superfast Temperature-Triggered Actuators. Adv. Mater. 2015, 27, 4865–4870. 10.1002/adma.201502133. 26186175

[ref305] Zhao L.; Huang J.; Zhang Y.; Wang T.; Sun W.; Tong Z. Programmable and Bidirectional Bending of Soft Actuators Based on Janus Structure with Sticky Tough PAA-Clay Hydrogel. ACS Appl. Mater. Interfaces 2017, 9, 11866–11873. 10.1021/acsami.7b00138. 28290198

[ref306] Yao C.; Liu Z.; Yang C.; Wang W.; Ju X. J.; Xie R.; Chu L. Y. Poly (N-Isopropylacrylamide)-Clay Nanocomposite Hydrogels with Responsive Bending Property as Temperature-Controlled Manipulators. Adv. Funct. Mater. 2015, 25, 2980–2991. 10.1002/adfm.201500420.

[ref307] Ma C.; Li T.; Zhao Q.; Yang X.; Wu J.; Luo Y.; Xie T. Supramolecular Lego Assembly Towards Three-Dimensional Multi-Responsive Hydrogels. Adv. Mater. 2014, 26, 5665–5669. 10.1002/adma.201402026. 24975743

[ref308] Zhang F.; Fan J.; Zhang P.; Liu M.; Meng J.; Jiang L.; Wang S. A Monolithic Hydro/Organo Macro Copolymer Actuator Synthesized Via Interfacial Copolymerization. Npg Asia Mater. 2017, 9, e380–e380. 10.1038/am.2017.61.

[ref309] Sanna D.; Alzari V.; Nuvoli D.; Nuvoli L.; Rassu M.; Sanna V.; Mariani A. β-Cyclodextrin-Based Supramolecular Poly (N-Isopropylacrylamide) Hydrogels Prepared by Frontal Polymerization. Carbohydr. Polym. 2017, 166, 249–255. 10.1016/j.carbpol.2017.02.099. 28385230

[ref310] Kong D.; Li J.; Guo A.; Zhang X.; Xiao X. Self-Healing High Temperature Shape Memory Polymer. Eur. Polym. J. 2019, 120, 109279–109289. 10.1016/j.eurpolymj.2019.109279.

[ref311] Meng H.; Yang X.; Wang Y.; Wang C.; Ye W.; Ma F.; Han T.; Qi J.; Wang C. Bio-inspired fluorescence color-tunable soft actuators with a self-healing and reconfigurable nature. Mater. Today Commun. 2022, 24, 100855–100865. 10.1016/j.mtchem.2022.100855.

[ref312] Hunt S.; McKay T. G.; Anderson I. A. A Self-Healing Dielectric Elastomer Actuator. Appl. Phys. Lett. 2014, 104, 113701–113704. 10.1063/1.4869294.

[ref313] Pelrine R. E.; Kornbluh R. D.; Joseph J. P. Electrostriction of Polymer Dielectrics with Compliant Electrodes as a Means of Actuation. Sens. Actuator A. Phys. 1998, 64, 77–85. 10.1016/S0924-4247(97)01657-9.

[ref314] Plante J. S.; Dubowsky S. On the Properties of Dielectric Elastomer Actuators and their Design Implications. Smart Mater. Struct. 2007, 16, S227–S247. 10.1088/0964-1726/16/2/S05.

[ref315] Koh S. J. A.; Zhao X.; Suo Z. Maximal Energy That Can Be Converted by A Dielectric Elastomer Generator. Appl. Phys. Lett. 2009, 94, 262902–262905. 10.1063/1.3167773.

[ref316] Zhang Y.; Ellingford C.; Zhang R.; Roscow J.; Hopkins M.; Keogh P.; McNally T.; Bowen C.; Wan C. Electrical and Mechanical Self-Healing in High-Performance Dielectric Elastomer Actuator Materials. Adv. Funct. Mater. 2019, 29, 1808431–1808444. 10.1002/adfm.201808431.

[ref317] Ellingford C.; Zhang R.; Wemyss A. M.; Bowen C.; McNally T.; Figiel Ł.; Wan C. Intrinsic Tuning of Poly(styrene–butadiene–styrene)-Based Self-Healing Dielectric Elastomer Actuators with Enhanced Electromechanical Properties. ACS Appl. Mater. Interfaces 2018, 10, 38438–38448. 10.1021/acsami.8b13785. 30360080

[ref318] Kalista S. J.; Ward T. C.; Oyetunji Z. Self-Healing of Poly(Ethylene-co-Methacrylic Acid) Copolymers Following Projectile Puncture. Mech. Adv. Mater. Struct. 2007, 14, 391–397. 10.1080/15376490701298819.

[ref319] Gao Y.; Fang X.; Tran D.; Ju K.; Qian B.; Li J. Dielectric Elastomer Actuators Based on Stretchable and Self-Healable Hydrogel Electrodes. R. Soc. Open Sci. 2019, 6, 182145–182154. 10.1098/rsos.182145. 31598224 PMC6731732

[ref320] Chen X.; Jiang T.; Yao Y.; Xu L.; Zhao Z.; Wang Z. L. Stimulating Acrylic Elastomers by a Triboelectric Nanogenerator – Toward Selfpowered Electronic Skin and Artificial Muscle. Adv. Funct. Mater. 2016, 26, 4906–4913. 10.1002/adfm.201600624.

[ref321] Cai G.; Wang J.; Qian K.; Chen J.; Li S.; Lee P. S. Extremely Stretchable Strain Sensors Based on Conductive Self-Healing Dynamic Crosslinks Hydrogels for Human-Motion Detection. Adv. Sci. 2017, 4, 1600190–1600197. 10.1002/advs.201600190. PMC5323873 28251045

[ref322] Kang J.; Tok J. B.-H.; Bao Z. Self-Healing Soft Electronics. Nat. Electron. 2019, 2, 144–150. 10.1038/s41928-019-0235-0.

[ref323] Ariga K.; Nishikawa M.; Mori T.; Takeya J.; Shrestha L. K.; Hill J. P. Self-Assembly as a Key Player for Materials Nanoarchitectonics. J. P. Hill, Sci. Technol. Sci. Technol. Adv. Mater. 2019, 20, 51–95. 10.1080/14686996.2018.1553108. 30787960 PMC6374972

[ref324] Lee J.; Tan M. W. M.; Parida K.; Thangavel G.; Park S. A.; Park T.; Lee P. S. Water-Processable, Stretchable, Self-Healable, Thermally Stable, and Transparent Ionic Conductors for Actuators and Sensors. Adv. Mater. 2020, 32, 1906679–1906689. 10.1002/adma.201906679. 31858638

[ref325] Yang Y.; Guan L.; Li X.; Gao Z.; Ren X.; Gao G. Conductive Organohydrogels with Ultrastretchability, Antifreezing, Self-Healing, and Adhesive Properties for Motion Detection and Signal Transmission. ACS Appl. Mater. Interfaces 2019, 11, 3428–3437. 10.1021/acsami.8b17440. 30592212

[ref326] Das A.; Sallat A.; Böhme F.; Suckow M.; Basu D.; Wießner S.; Stöckelhuber K. W.; Voit B.; Heinrich G. Ionic Modification Turns Commercial Rubber into a Self-Healing Material. ACS Appl. Mater. Interfaces 2015, 7, 20623–20630. 10.1021/acsami.5b05041. 26332010

[ref327] Duan C.; Zhang K.; Zhong C.; Huang F.; Cao Y. Recent Advances in Water/Alcohol-Soluble Π-Conjugated Materials: New Materials and Growing Applications in Solar Cells. Chem. Soc. Rev. 2013, 42, 9071–9104. 10.1039/c3cs60200a. 23995779

[ref328] Li R.; Fan T.; Chen G.; Zhang K.; Su B.; Tian J.; He M. Autonomous Self-Healing, Anti-freezing, and Transparent Conductive Elastomers. Chem. Mater. 2020, 32, 874–881. 10.1021/acs.chemmater.9b04592.

[ref329] Wang M.; Li R.; Chen G.; Zhou S.; Feng X.; Chen Y.; He M.; Liu D.; Song T.; Qi H. Highly Stretchable, Transparent, and Conductive Wood Fabricated by in Situ Photopolymerization with Polymerizable Deep Eutectic Solvents. ACS Appl. Mater. Interfaces 2019, 11, 14313–14321. 10.1021/acsami.9b00728. 30915834

[ref330] Ren’ai L.; Zhang K.; Chen G.; Su B.; Tian J.; He M.; Lu F. Green Polymerizable Deep Eutectic Solvent (PDES) Type Conductive Paper for Origami 3D Circuits. Chem. Commun. 2018, 54, 2304–2307. 10.1039/C7CC09209A. 29445790

[ref331] Gao H.; Zhao Z.; Cai Y.; Zhou J.; Hua W.; Chen L.; Wang L.; Zhang J.; Han D.; Liu M.; Jiang L. Adaptive and Freeze-Tolerant Heteronetwork Organohydrogels with Enhanced Mechanical Stability Over a Wide Temperature Range. Nat. Commun. 2017, 8, 15911 10.1038/ncomms15911. 28639615 PMC5489716

[ref332] Koo J. H.; Kim D. C.; Shim H. J.; Kim T. H.; Kim D. H. Flexible and Stretchable Smart Display: Materials, Fabrication, Device Design, and System Integration. Adv. Funct. Mater. 2018, 28, 1801834–1801857. 10.1002/adfm.201801834.

[ref333] Li S.; Peele B. N.; Larson C. M.; Zhao H.; Shepherd R. F. A Stretchable Multicolor Display and Touch Interface Using Photopatterning and Transfer Printing. Adv. Mater. 2016, 28, 9770–9775. 10.1002/adma.201603408. 27717071

[ref334] Duan L.; Lai J.-C.; Li C.-H.; Zuo J.-L. A Dielectric Elastomer Actuator That Can Self-Heal Integrally. ACS Appl. Mater. Interfaces 2020, 12, 44137–44146. 10.1021/acsami.0c11697. 32926620

[ref335] Stoyanov H.; Kollosche M.; Risse S.; Waché R.; Kofod G. Soft Conductive Elastomer Materials for Stretchable Electronics and Voltage Controlled Artificial Muscles. Adv. Mater. 2013, 25, 578–583. 10.1002/adma.201202728. 23090668

[ref336] Wang Y.; Liu X.; Li S.; Li T.; Song Y.; Li Z.; Zhang W.; Sun J. Transparent, Healable Elastomers with High Mechanical Strength and Elasticity Derived from Hydrogen-Bonded Polymer Complexes. ACS Appl. Mater. Interfaces 2017, 9, 29120–29129. 10.1021/acsami.7b08636. 28795571

[ref337] Zhang Q.; Shi C.-Y.; Qu D.-H.; Long Y.-T.; Feringa B. L.; Tian H. Exploring a Naturally Tailored Small Molecule for Stretchable, Self-Healing, and Adhesive Supramolecular Polymers. Sci. Adv. 2018, 4, eaat8192 10.1126/sciadv.aat8192. 30062126 PMC6063538

[ref338] Sun H.; Liu X.; Liu S.; Yu B.; Ning N.; Tian M.; Zhang L. Silicone Dielectric Elastomer with Improved Actuated Strain at Low Electric Field and High Self-Healing Efficiency by Constructing Supramolecular Network. Chem. Eng. J. 2020, 384, 123242–123254. 10.1016/j.cej.2019.123242.

[ref339] Yang D.; Huang S.; Ruan M.; Li S.; Wu Y.; Guo W.; Zhang L. Improved Electromechanical Properties of Silicone Dielectric Elastomer Composites by Tuning Molecular Flexibility. Compos. Sci. Technol. 2018, 155, 160–168. 10.1016/j.compscitech.2017.12.010.

[ref340] Tian M.; Ma Q.; Li X.; Zhang L.; Nishi T.; Ning N. High Performance Dielectric Composites by Latex Compounding of Graphene Oxide-Encapsulated Carbon Nanosphere Hybrids with XNBR. J. Mater. Chem. A 2014, 2, 11144–11154. 10.1039/C4TA01600A.

[ref341] Pal A.; Basit H.; Sen S.; Aswal V. K.; Bhattacharya S. Structure and Properties of Two Component Hydrogels Comprising Lithocholic Acid and Organic Amines. J. Mater. Chem. 2009, 19, 4325–4334. 10.1039/b903407b.

[ref342] Hardy J. G.; Hirst A. R.; Smith D. K.; Brennan C.; Ashworth I. Controlling the Materials Properties and Nanostructure of a Single-Component Dendritic Gel by Adding a Second Component. Chem. Commun. 2005, 385–387. 10.1039/b413629b. 15645046

[ref343] Sun H.; Liu X.; Yu B.; Feng Z.; Ning N.; Hu G.-H.; Tian M.; Zhang L. Simultaneously Improved Dielectric and Mechanical Properties of Silicone Elastomer by Designing a Dual Crosslinking Network. Polym. Chem. 2019, 10, 633–645. 10.1039/C8PY01763H.

[ref344] Ning N.; Wang Z.; Yao Y.; Zhang L.; Tian M. Enhanced electromechanical performance of bio-based gelatin/glycerin dielectric elastomer by cellulose nanocrystals. Carbohydrate polymers. 2015, 130, 262–267. 10.1016/j.carbpol.2015.03.083. 26076625

[ref345] Nie R.-P.; Tang W.-B.; Li Y.; Jia L.-C.; Xu L.; Huang H.-D.; Lei J.; Li Z.-M. Surfactant-Assisted Fabrication of Room-Temperature Self-Healable Dielectric Elastomer Toward Actuation Application. Compos. B Eng. 2022, 234, 109655 10.1016/j.compositesb.2022.109655.

[ref346] Liu X.; Xu H.; Li Y.; Jing M.; Wang W.; Li Z.; Zhang P.; Sun Z. A Stretchable and Self-Healing Ionic Artificial Muscle Modified by Conductive Substances. Appl. Phys. A: Mater. Sci. Process. 2022, 128, 116–127. 10.1007/s00339-021-05245-7.

[ref347] Shi Q.; Liu H.; Tang D.; Li Y.; Li X.; Xu F. Bioactuators based on stimulus-responsive hydrogels and their emerging biomedical applications. NPG Asia Mater. 2019, 11, 64 10.1038/s41427-019-0165-3.

[ref348] Yoon C. Advances in Biomimetic Stimuli Responsive Soft Grippers. Nano Converg. 2019, 6, 20 10.1186/s40580-019-0191-4. 31257552 PMC6599812

[ref349] Wang S.; Guo X.; Guo P.; Guan S.; Fu H.; Cui W.; Ao Y. Tunable Mechanical and Self-Healing Poly (Acrylic Acid-Co-Stearyl Methacrylate) Hydrogels Induced by Soaking Methods. Coll. Surf. A Physicochem. Eng. Asp. 2021, 624, 126755–126764. 10.1016/j.colsurfa.2021.126755.

[ref350] Yang C.; Liu Z.; Chen C.; Shi K.; Zhang L.; Ju X.; Wang W.; Xie R.; Chu L. Reduced Graphene Oxide-Containing Smart Hydrogels with Excellent Electro-Response and Mechanical Properties for Soft Actuators. ACS Appl. Mater. Interfaces 2017, 9, 15758–15767. 10.1021/acsami.7b01710. 28425695

[ref351] Sanchez T.; Salcedo E.; Ceballos H.; Dufour D.; Mafla G.; Morante N.; Calle F.; Perez J. C.; Debouck D.; Jaramillo G.; et al. Screening of Starch Quality Traits in Cassava (Manihotesculenta Crantz). Starch/Staerke 2009, 61, 12–19. 10.1002/star.200800058.

[ref352] Shang X.; Wang Q.; Li J.; Zhang G.; Zhang J.; Liu P.; Wang L. Double-Network Hydrogels with Superior Self-Healing Properties Using Starch Reinforcing Strategy. Carbohy. Polym. 2021, 257, 117626–117636. 10.1016/j.carbpol.2021.117626. 33541652

[ref353] Nie R.-P.; Lin H.; Li Y.; Huang H.-D.; Yan D.-X.; Dai K.; Lei J.; Li Z.-M. Dynamic Chemical Bonds Design Strategy for Fabricating Fast Room-Temperature Healable Dielectric Elastomer with Significantly Improved Actuation Performance. Chem. Eng. J. 2022, 439, 135683 10.1016/j.cej.2022.135683.

[ref354] Xiang Z.; Chu C.; Xie H.; Xiang T.; Zhou S. Multifunctional Thermoplastic Polyurea Based on the Synergy of Dynamic Disulfide Bonds and Hydrogen Bond Cross-Links. ACS Appl. Mater. Interfaces 2021, 13, 1463–1473. 10.1021/acsami.0c18396. 33382585

[ref355] Tan M.-W.-M.; Thangavel G.; Lee P.-S. Rugged Soft Robots using Tough, Stretchable, and Self-Healable Adhesive Elastomers. Adv. Funct. Mater. 2021, 31, 2103097 10.1002/adfm.202103097.

[ref356] Xiang D.; He J.; Cui T.; Liu L.; Shi Q.-S.; Ma L.-C.; Liang Y. Multiphase Structure and Electromechanical Behaviors of Aliphatic Polyurethane Elastomers. Macromolecules 2018, 51, 6369–6379. 10.1021/acs.macromol.8b01171.

[ref357] Yao J.; Liu X.; Sun H.; Liu S.; Jiang Y.; Yu B.; Ning N.; Tian M.; Zhang L. Thermoplastic Polyurethane Dielectric Elastomers with High Actuated Strain and Good Mechanical Strength by Introducing Ester Group Grafted Polymethylvinylsiloxane. Ind. Eng. Chem. Res. 2021, 60, 4883–4891. 10.1021/acs.iecr.1c00362.

[ref358] Renard C.; Wang D.; Han P.; Xiong S.; Wen Y.; Dang Z. M.. Remarkably Improved Electromechanical Actuation of Polyurethane Enabled by Blending with Silicone Rubber. RSC Adv. 2017, 7, 22900–22908. 10.1039/C7RA03274A.

[ref359] Ning N.; Qin H.; Wang M.; Sun H.; Tian M.; Zhang L. Improved Dielectric and Actuated Performance of Thermoplastic Polyurethane by Blending with XNBR as Macromolecular Dielectrics. Polymer 2019, 179, 121646 10.1016/j.polymer.2019.121646.

[ref360] Caspari P.; Nüesch F.-A.; Opris D.-M. Synthesis of Solvent-Free Processable and On-Demand Cross-Linkable Dielectric Elastomers for Actuators. J. Mater. Chem. C 2019, 7, 12139–12150. 10.1039/C9TC03391B.

[ref361] Gallone G.; Galantini F.; Carpi F. Perspectives for New Dielectric Elastomers with Improved Electromechanical Actuation Performance: Composites versus Blends. Polym. Int. 2010, 59, 400–406. 10.1002/pi.2765.

[ref362] Zhang Z.; Wang X.; Tan S.; Wang Q. Superior Electrostrictive Strain Achieved Under Low Electric Fields in Relaxor Ferroelectric Polymers. J. Mater. Chem. A 2019, 7, 5201–5208. 10.1039/C8TA11938D.

[ref363] Von Szczepanski J.; Danner P.-M.; Opris D.-M. Self-Healable, Self-Repairable, and Recyclable Electrically Responsive Artificial Muscles. Adv. Sci. 2022, 9, 2202153 10.1002/advs.202202153. PMC9353453 35657031

[ref364] Zhu Y.; Lin L.; Chen Y.; Song Y.; Lu W.; Guo Y. A Self-Healing, Robust Adhesion, Multiple Stimuli-Response Hydrogel for Flexible Sensors. Soft Matter 2020, 16, 2238–2248. 10.1039/C9SM02303H. 32025677

[ref365] Chen W. P.; Hao D. Z.; Hao W. J.; Guo X. L.; Jiang L. Hydrogel with Ultrafast Self-Healing Property Both in Air and Underwater. ACS Appl. Mater. Interfaces 2018, 10, 1258–1265. 10.1021/acsami.7b17118. 29235838

[ref366] Li C.; Mu C.; Lin W.; Ngai T. Gelatin Effects on the Physicochemical and Hemocompatible Properties of Gelatin/PAAm/Laponite Nanocomposite Hydrogels. ACS Appl. Mater. Interfaces 2015, 7, 18732–18741. 10.1021/acsami.5b05287. 26202134

[ref367] Zhai X.; Ma Y.; Hou C.; Gao F.; Zhang Y.; Ruan C.; Pan H.; Lu W. W.; Liu W. 3D-Printed High Strength Bioactive Supramolecular Polymer/Clay Nanocomposite Hydrogel Scaffold for Bone Regeneration. ACS Biomater. Sci. Eng. 2017, 3, 1109–1118. 10.1021/acsbiomaterials.7b00224. 33429585

[ref368] Haraguchi K.; Takehisa T.; Ebato M. Control of Cell Cultivation and Cell Sheet Detachment on the Surface of Polymer/Clay Nanocomposite Hydrogels. Biomacromolecules 2006, 7, 3267–3275. 10.1021/bm060549b. 17096560

[ref369] Chen Q.; Zhu L.; Chen H.; Yan H.; Huang L.; Yang J.; Zheng J. A Novel Design Strategy for Fully Physically Linked Double Network Hydrogels with Tough, Fatigue Resistant, and Self-Healing Properties. Adv. Funct. Mater. 2015, 25, 1598–1607. 10.1002/adfm.201404357.

[ref370] Gao G.; Wang Z.; Xu D.; Wang L.; Xu T.; Zhang H.; Chen J.; Fu J. Snap-Buckling Motivated Controllable Jumping of Thermo-Responsive Hydrogel Bilayers. ACS Appl. Mater. Interfaces 2018, 10, 41724–41731. 10.1021/acsami.8b16402. 30387979

[ref371] Li X.; Wang H.; Li D.; Long S.; Zhang G.; Wu Z. Dual Ionically Cross-linked Double-Network Hydrogels with High Strength, Toughness, Swelling Resistance, and Improved 3D Printing Processability. ACS Appl. Mater. Interfaces 2018, 10, 31198–31207. 10.1021/acsami.8b13038. 30148345

[ref372] Yu H. C.; Li C. Y.; Du M.; Song Y.; Wu Z. L.; Zheng Q. Improved Toughness and Stability of κ-Carrageenan/Polyacrylamide Double-Network Hydrogels by Dual Cross-Linking of the First Network. Macromolecules 2019, 52, 629–638. 10.1021/acs.macromol.8b02269.

[ref373] Pena-Francesch A.; Jung H.; Demirel M. C.; Sitti M. Biosynthetic Self-Healing Materials for Soft Machines. Nat. Mater. 2020, 19, 1230–1235. 10.1038/s41563-020-0736-2. 32719508 PMC7610468

[ref374] Zhong M.; Wang R.; Kawamoto K.; Olsen B. D.; Johnson J. A. Quantifying the Impact of Molecular Defects on Polymer Network Elasticity. Science 2016, 353, 1264–1268. 10.1126/science.aag0184. 27634530

[ref375] Pena-Francesch A.; Jung H.; Segad M.; Colby R. H.; Allen B. D.; Demirel M. C. Mechanical Properties of Tandem-Repeat Proteins are Governed by Network Defects. ACS Biomater. Sci. Eng. 2018, 4, 884–891. 10.1021/acsbiomaterials.7b00830. 33418772

[ref376] Sariola V.; Pena-Francesch A.; Jung H.; Çetinkaya M.; Pacheco C.; Sitti M.; Demirel M. C. Segmented Molecular Design of Self-Healing Proteinaceous Materials. Sci. Rep. 2015, 5, 13482–13491. 10.1038/srep13482. 26323335 PMC4555047

[ref377] Ding D.; Guerette P. A.; Fu J.; Zhang S.; Irvine A.; Miserez I. From Soft Self-Healing Gels to Stiff Films in Suckerin-Based Materials Through Modulation of Crosslink Density and β-Sheet Content. Adv. Mater. 2015, 27, 3953–3961. 10.1002/adma.201500280. 26011516

[ref378] Blaiszik B. J.; Kramer S. L. B.; Olugebefola S. C.; Moore J. S.; Sottos N. R.; White S. R. Self-Healing Polymers and Composites. Annu. Rev. Mater. Res. 2010, 40, 179–211. 10.1146/annurev-matsci-070909-104532.

[ref379] Bier J. M.; Verbeek C. J. R.; Lay M. C. Thermal Transitions and Structural Relaxations in Protein-Based Thermoplastics. Macromol. Mater. Eng. 2014, 299, 524–539. 10.1002/mame.201300248.

[ref380] Tomko J. A.; Pena-Francesch A.; Jung H.; Tyagi M.; Allen B. D.; Demirel M. C.; Hopkins P. E. Tunable Thermal Transport and Reversible Thermal Conductivity Switching in topologically networked bio-inspired materials. Nat. Nanotechnol. 2018, 13, 959–964. 10.1038/s41565-018-0227-7. 30104620

[ref381] Ilievski F.; Mazzeo A. D.; Shepherd R. F.; Chen X.; Whitesides G. M. Soft Robotics for Chemists. Angew. Chem., Int. Ed. 2011, 50, 1890–1895. 10.1002/anie.201006464. 21328664

[ref382] Song S.; Drotlef D.-M.; Majidi C.; Sitti M. Controllable Load Sharing for Soft Adhesive Interfaces on Three-Dimensional Surfaces. Proc. Natl. Acad. Sci. 2017, 114, E4344–E4353. 10.1073/pnas.1620344114. 28507143 PMC5465890

[ref383] Madden J. D. W.; Vandesteeg N. A.; Anquetil P. A.; Madden P. G. A.; Takshi A.; Pytel R. Z.; Lafontaine S. R.; Wieringa P. A.; Hunter I. W. Artificial Muscle Technology: Physical Principles and Naval Prospects. IEEE J. Ocean. Eng. 2004, 29, 706–728. 10.1109/JOE.2004.833135.

[ref384] Miriyev A.; Stack K.; Lipson H. Soft Material for Soft Actuators. Nat. Commun. 2017, 8, 596 10.1038/s41467-017-00685-3. 28928384 PMC5605691

[ref385] Rich S. I.; Wood R. J.; Majidi C. Untethered Soft Robotics. Nat. Electron. 2018, 1, 102–112. 10.1038/s41928-018-0024-1.

[ref386] Pena-Francesch A.; Giltinan J.; Sitti M. Multifunctional and Biodegradable Self-Propelled Protein Motors. Nat. Commun. 2019, 10, 3188 10.1038/s41467-019-11141-9. 31320630 PMC6639312

[ref387] Wang Z.; Cui H.; Liu M.; Grage S. L.; Hoffmann M.; Sedghamiz E.; Wenzel W.; Levkin P. A. Tough, Transparent, 3D-Printable, and Self-Healing Poly(ethylene glycol)-Gel (PEGgel). Adv. Mater. 2022, 34, 2107791 10.1002/adma.202107791. 34854140

[ref388] Miyamae K.; Nakahata M.; Takashima Y.; Harada A. Self-Healing, Expansion–Contraction, and Shape-Memory Properties of a Preorganized Supramolecular Hydrogel through Host–Guest Interactions. Angew. Chem., Int. Ed. 2015, 54, 8984–8987. 10.1002/anie.201502957. 26080301

